# Highly Diastereoselective Multicomponent Synthesis of Spirocyclopropyl Oxindoles Enabled by Rare-Earth Metal Salts

**DOI:** 10.1021/acs.orglett.3c00772

**Published:** 2023-04-26

**Authors:** Matteo A. Tallarida, Fabrizio Olivito, Claudio D. Navo, Vincenzo Algieri, Antonio Jiritano, Paola Costanzo, Ana Poveda, Maria J. Moure, Jesús Jiménez-Barbero, Loredana Maiuolo, Gonzalo Jiménez-Osés, Antonio De Nino

**Affiliations:** † Department of Chemistry and Chemical Technologies, University of Calabria, Via P. Bucci, Cubo 12C, 87036 Rende, Italy; ‡ Center for Cooperative Research in Biosciences (CIC bioGUNE), Basque Research and Technology Alliance (BRTA), Bizkaia Technology Park, Building 800, 48160 Derio, Spain; § Ikerbasque, Basque Foundation for Science, 48013 Bilbao, Spain; ∥ Department of Organic Chemistry II, Faculty of Science & Technology, University of the Basque Country, Leioa 48940, Bizkaia, Spain; ⊥ Centro de Investigacion Biomedica En Red de Enfermedades Respiratorias, 28029 Madrid, Spain

## Abstract

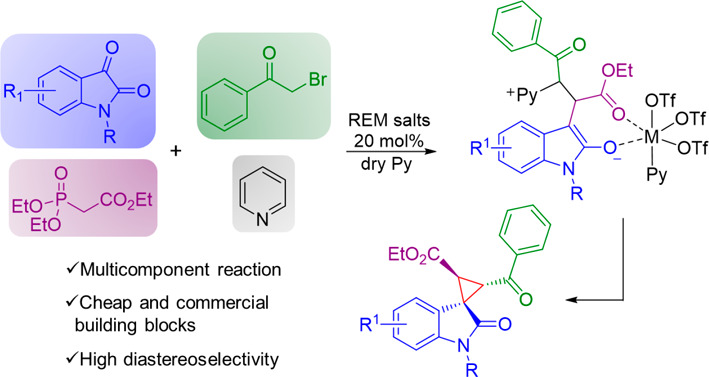

The synthesis of polysubstituted spirocyclopropyl oxindoles using a series of rare-earth metal (REM) salts is reported. REMs, in particular Sc(OTf)_3_, allowed access to the target compounds by a multicomponent reaction with high diastereoselectivity (≤94:6:0:0). Density functional theory calculations on the model reaction are consistent with the observed selectivity and revealed that the special coordinating capabilities and the oxophilicity of the metal are key factors in inducing the formation of one main diastereoisomer.

Rare-earth metals (REMs) constitute a large family of heavy metals, including 17 lanthanoid elements, yttrium, and scandium. REMs are characterized by special physicochemical properties and are widely employed in many synthetic and engineering applications. They are particularly useful in organic synthesis due to their Lewis acid properties, versatility, and low toxicity.^1^ For this reason, REMs have been employed, mostly as halogen or triflate salts, for many chemical transformations,^2,3^ particularly in recent years.^4^ Multicomponent reactions (MCRs) make up a class of one-pot synthetic approaches involving three or more reactants to obtain products with high atom economy, particularly useful in medicinal chemistry.^5,6^ MCRs also proved to be very useful for the preparation of oxindole-containing small molecules,^7−10^ a scaffold that is relevant in drug discovery.^11,12^ In this context, spirocyclopropyl oxindoles comprise a cyclopropyl moiety fused to position C3 of an oxindole core (Figure 1).

**Figure 1:**
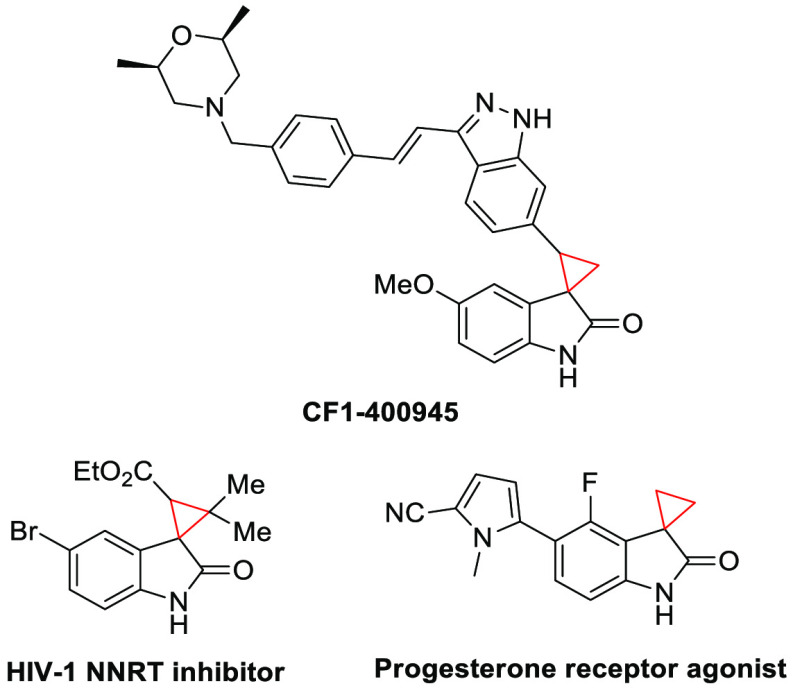
Representative bioactive spirocyclopropyl oxindoles.

Such compounds have attracted more attention over the past several years, mainly due to the broad spectrum of biological activities they display.^13−16^ Also, they pose a synthetic challenge associated with their high ring strain energy (∼27 kcal mol^–1^) (Figure 1, red moiety), particularly if substitution at multiple positions of the ring is needed.

Spirocyclopropyl oxindoles have been synthesized using 3-halooxindole,^17^ 3-diazooxindoles,^18^ oxindole,^19^ and 3-alkylideneoxindoles as starting reagents (Scheme 1).

**Scheme 1:**
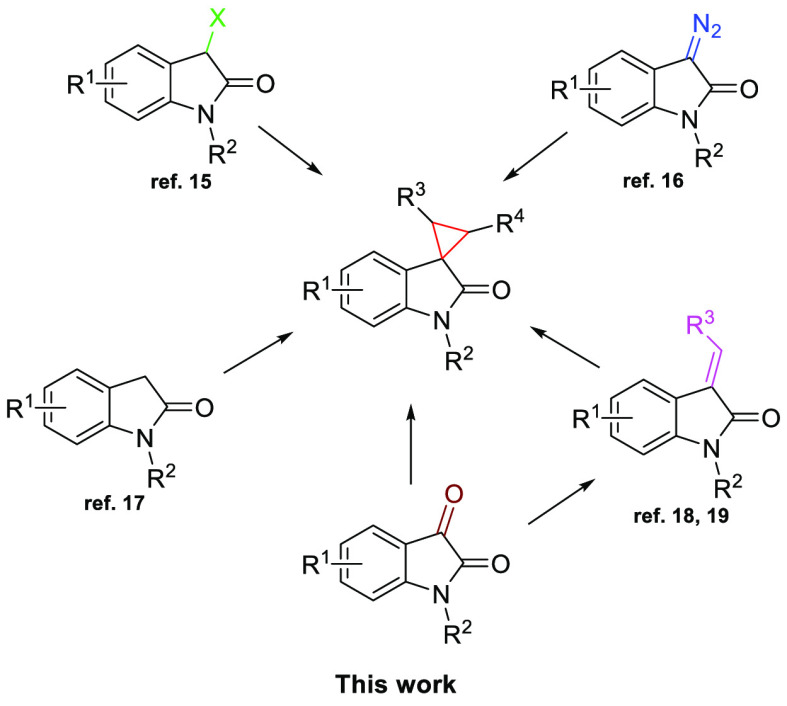
Common Precursors for the Synthesis of Spirocyclopropyl Oxindoles

With regard to 3-alkylideneoxindoles, the Feng group has made remarkable contributions in the past several years by using metal-activated sulfoxonium^20^ or cyclic sulfur^21^ ylides in the presence of chiral ligands.^22^

In this context, a limited number of synthetic procedures starting from commercially available isatin derivatives have been reported.^23^ Hence, in this work, we describe a highly diastereoselective multicomponent synthesis of spirocyclopropyl oxindoles starting from simple, inexpensive reagents in the presence of nonchiral REM salts.

Given our experience on the stereoselective synthesis of spirooxindole derivatives,^24,25^ we explored a four-component reaction entailing *N*-methylisatin **1a**, triethyl phosphono-acetate **2**, 2-bromoacetophenone **3**, and pyridine (Py) **4** in the presence of potassium carbonate. Pyridine was used as both a reagent and a solvent (Table 1). The optimal reaction temperature was 70 °C, because preliminary assays at 25 and 50 °C gave poor yields (22% and 58%, respectively). Protected *N*-methylisatin was chosen to avoid the formation of undesired byproducts under basic conditions.

**Table 1:**
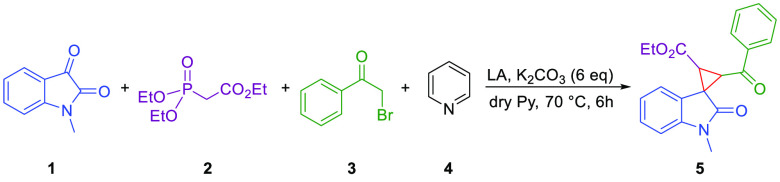
Optimization of the Reaction Conditions^a^

entry	Lewis acid	dr^d^	yield (%)
1^b^	–	–	–
2^c^	Sc(OTf)_3_	92:8:0:0	63
3	Sc(OTf)_3_	92:8:0:0	95
4	Er(OTf)_3_	89:11:0:0	88
5	Yb(OTf)_3_	90:10:0:0	90
6	Ho(OTf)_3_	88:12:0:0	81
7	Ce(OTf)_3_	90:10:0:0	79
8	La(OTf)_3_	91:9:0:0	75
9^e^	Sc(OTf)_3_	91:9:0:0	90
10^f^	ScBr_3_	89:11:0:0	93

a Reaction conditions: **1a** (0.1 mmol), **2** (0.1 mmol), **3** (0.25 mmol), **4** (0.35 mmol), Lewis acid (LA, 20 mol %), dry Py as the solvent (2 mL), 70 °C, N_2_ atmosphere.

b Reaction performed in the absence of a Lewis acid and under prolonged heating.

c With 10 mol % Lewis acid.

d dr calculated by GC/MS.

e Gram-scale reaction (see the Supporting Information for details).

f Reaction using ScBr_3_ (20 mol %) as a catalyst.

As one can see (Table 1, entry 1), in the absence of a Lewis acid, no product was obtained after 24 h. Rare-earth metal salts were then explored as catalysts for this reaction, considering their well-known oxophilicity^26^ and efficient activation of carbonyls.^27^ The use of triflate as a counterion derives from their tendency to maintain the ion pair in organic solvents such as pyridine. In fact, it is known that in the absence of water, rare-earth metals predominantly preserve their nondissociated nature.^28^ This property of the catalyst has mechanistic implications, because the metal normally expands its coordination sphere and changes its geometry upon reagent binding. For this reason, it was necessary to work in the strict absence of water that could saturate the metal-coordinating sphere, blocking its Lewis acid capability. In fact, preliminary tests in the presence of water did not lead to any product. Therefore, we tested Sc(OTf)_3_, Er(OTf)_3_, Yb(OTf)_3_, Ho(OTf)_3_, Ce(OTf)_3_, and La(OTf)_3_ as catalysts (Table 1, entries 2–8). With 10% Sc(OTf)_3_, spiro derivative **5** was obtained in a moderate yield (Table 1, entry 2), and when the amount of catalyst was doubled, the reaction yield was excellent (Table 1, entry 3). Fittingly, other rare-earth triflates led to similar or slightly lower yields and selectivities (Table 1, entries 4–8). It is noteworthy that in all cases the reaction is highly diastereoselective toward one particular isomer [dr = 92:8:0:0 (Table 1, entry 3)]. We tentatively attributed the observed diastereoselectivity to the special coordinating capabilities of REMs in the presence of the three O-donor carbonyls present in the substrates (see the computational study below). Other organic solvents were tested (Table 2). According to the observed results, aprotic and polar/coordinating solvents such as DMF and ACN are viable alternatives to pyridine in the presence of Sc(OTf)_3_ as a Lewis acid.

**Table 2:** Screening of the Model Reaction in Various Solvents^a^

entry	solvent	yield (%)
1	DMF	94
2	EtOH	–
3	DCE	73
4	ACN	86

a Reaction conditions: **1a** (0.1 mmol), **2** (0.1 mmol), **3** (0.25 mmol), **4** (0.35 mmol), Sc(OTf)_3_ (20 mol %), dry solvent (2 mL) 70 °C, N_2_ atmosphere. DMF = *N*,*N*-dimethylformamide. DCE = 1,2-dichloroethane. ACN = acetonitrile. It is noteworthy that the presence of solid potassium carbonate as a base in dry pyridine did not affect the global yield when the reaction was performed at a gram scale (Table 1, entry 9). Finally, the reaction performed equally well with ScBr_3_ as a catalyst (Table 1, entry 10). Using the optimized conditions [20% Sc(OTf)_3_], the protocol was extended to substrates **1b**–**l**, starting from commercially available isatins subjected to N-alkylation^29^ (Table 3).

**Table 3:**
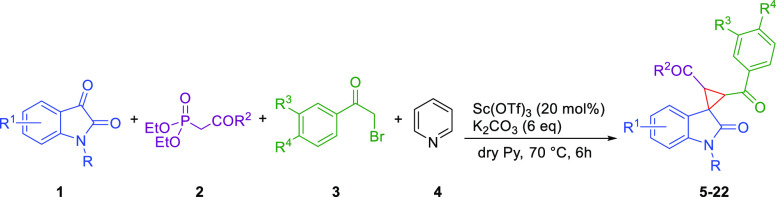

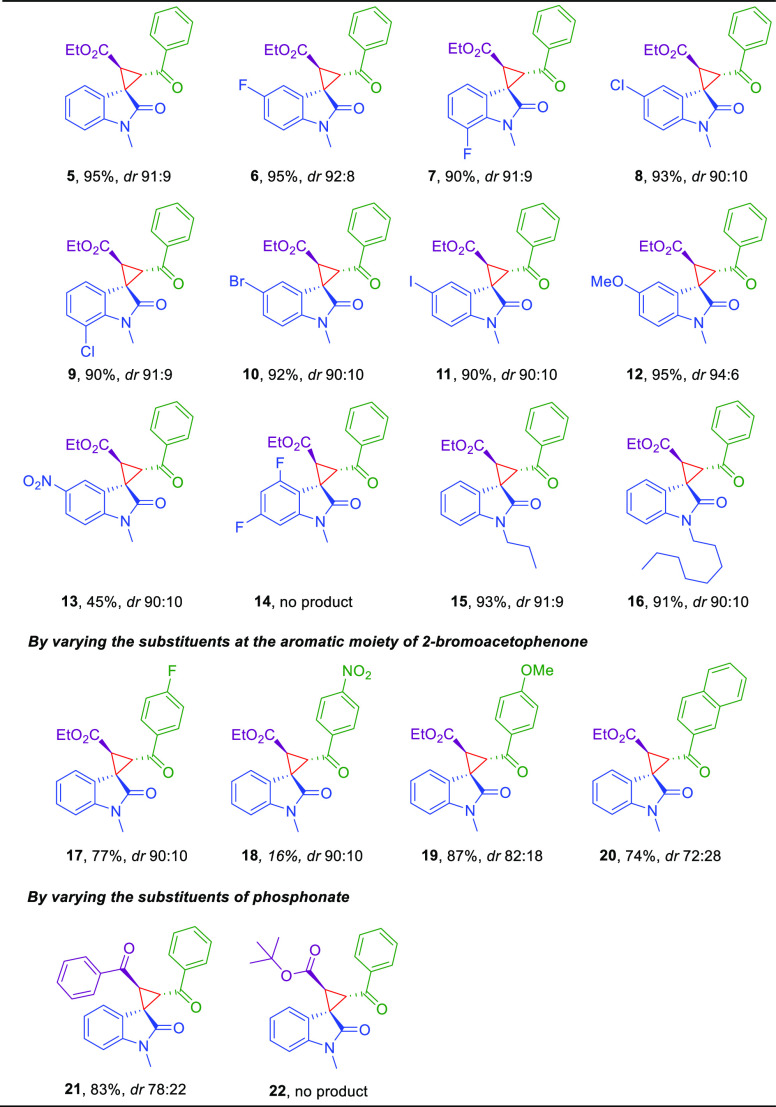
Scope of the Diastereoselective Multicomponent Reaction^a^–^c^

a Reaction conditions: **1** (0.1 mmol), **2** (0.1 mmol), **3** (0.25 mmol), **4** (0.35 mmol), Sc(OTf)_3_ (20 mol %), dry Py as the solvent (2 mL), 70 °C, N_2_ atmosphere. The yield refers to isolated products, and dr values were determined by ^1^H NMR analysis of the crude reaction mixture.

b Only one arbitrary enantiomer of the racemic mixture of the major diastereoisomer is shown.

c The relative stereochemistry was determined by NOE-based NMR experiments (see the Supporting Information).

The influence of electron-withdrawing and electron-donating substituents at different positions of the isatin benzene ring was investigated. As one can see from the results summarized in Table 2, no significant variations in yield or stereoselectivity were observed for compounds **6**–**12**; for derivative **13**, the presence of a nitro group drastically reduced the reaction yield, while the two fluorine atoms prevented the formation of product **14**. Consequently, the reaction tolerates electron-donating and moderately electron-withdrawing groups at the isatin core, while highly deactivated systems are poorly or not reactive. Moreover, we synthesized compounds with *N*-alkyl chains of different lengths with the aim of tuning the lipophilicity of the final product for possible biological applications, being able to isolate **15** and **16** in similarly high yields and diastereoselectivity. Then, we expanded the reaction scope by changing the substituents at the aromatic ring of 2-bromoacetophenone **3** (Table 2, entries 17–20), affording a generally good tolerance to diverse functional groups except for the nitro group (**18**, 16% yield), probably due to the strongly deactivating effect on the intermediate pyridinium ylide. Then, we used other phosphonate derivatives **2** (Table 2, entries 21 and 22), affording a good yield for compound **21** and no formation of product **22**, probably due to the steric hindrance of the *tert*-butyl group. Finally, we wanted to investigate a possible application for our reaction by choosing propachlor **23** as a surrogate of the 2-bromoacetophenone (Scheme 2). Propachlor is a well-known herbicide,^30^ and today, much effort is being spent to develop new herbicides,^31^ in particular new spiro pesticide derivatives.^32^ Therefore, the new spirocyclopropyl derivative **24** may represent a novel substrate with herbicidal properties.

**Scheme 2:**
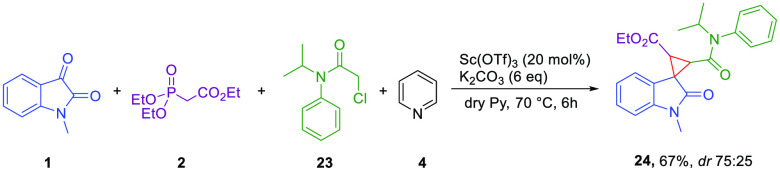
Spirocyclopropyl Derivative **24** with Propachlor **23**

On the basis of the pioneering work done by Bencivenni and Bartoli on Michael addition-initiated annulations,^33^ a plausible mechanism for our multicomponent reaction is proposed (Scheme 3).

**Scheme 3:**
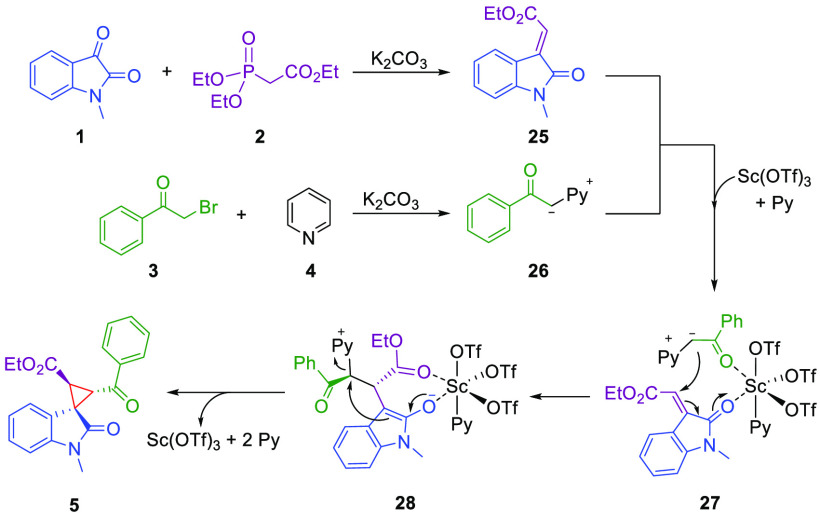
Proposed Reaction Mechanism

Such a mechanism involves the concomitant formation of oxoindolinylidene **25** through a Horner–Wadsworth–Emmons reaction between *N*-methylisatin **1** and phosphonate **2** and of pyridinium ylide **26** from bromoacetophenone **3** and pyridine **4** (Scheme 3).^29,33,34^^1^H NMR experiments confirmed the formation of only the *E* isomer of **25**, as expected (see the Supporting Information). In agreement with recent findings by Boyle et al.,^35^ we hypothesized the formation of an octahedral Sc(OTf)_3_Py_3_ complex after the addition of Sc(OTf)_3_ to the reaction mixture. Then intermediates **25** and **26** coordinate to the metal through their carbonyl groups by displacing two pyridine molecules and forming complex **27**, which undergoes intramolecular Michael addition. Finally, enolate **28** intramolecularly displaces the pyridinium moiety to form the cyclopropane ring in product **5**. DFT calculations were performed to validate the proposed mechanism and shed light into the origins of the observed stereoselectivity by using ωB97X-D as a density functional, 6-31G(d,p) as the basis set, LanL2DZ as the effective core potential for Sc and Br atoms, and PCM for the solvent modeling. For this purpose, the minimum energy pathways for three diastereomeric spirocyclopropyl oxindoles (**5a**, 1*R*,2*S*,3*S*; **5b**, 1*R*,2*R*,3*R*; **5c**, 1*R*,2*R*,3*S*) were calculated (Figure 2 and the Supporting Information). All pathways start from the octahedral scandium complex formed by three bromides, one pyridine molecule, and intermediates **25** and **26** coordinated through their carbonyls [initial complex (**IC**)]. Bromide anions were used as ligands considering the good yields obtained with ScBr_3_ as a catalyst (Table 1, entry 10). Calculations corroborated the high affinity of Sc for water, which enforces the use of dry pyridine as a solvent to prevent catalyst poisoning (see the Supporting Information). The first calculated step was the stereoselective Michael addition of the coordinated ylide to the oxoindolinylidene with relatively low activation barriers (Δ*G*^⧧^ = 18.1 kcal mol^–1^ for **TS1**_**RR**_, and Δ*G*^⧧^ = 23.6 kcal mol^–1^ for **TS1**_**RS**_) leading to thermoneutral enolates (Δ*G* ∼ 0 kcal mol^–1^ for **Int1b** and **Int1c**). A slightly more stable intermediate is generated from **Int1b** by decoordination of the ketone (green) and coordination of the ester (purple) groups (Δ*G* = −4.5 kcal mol^–1^ for **Int1a**).

**Figure 2:**
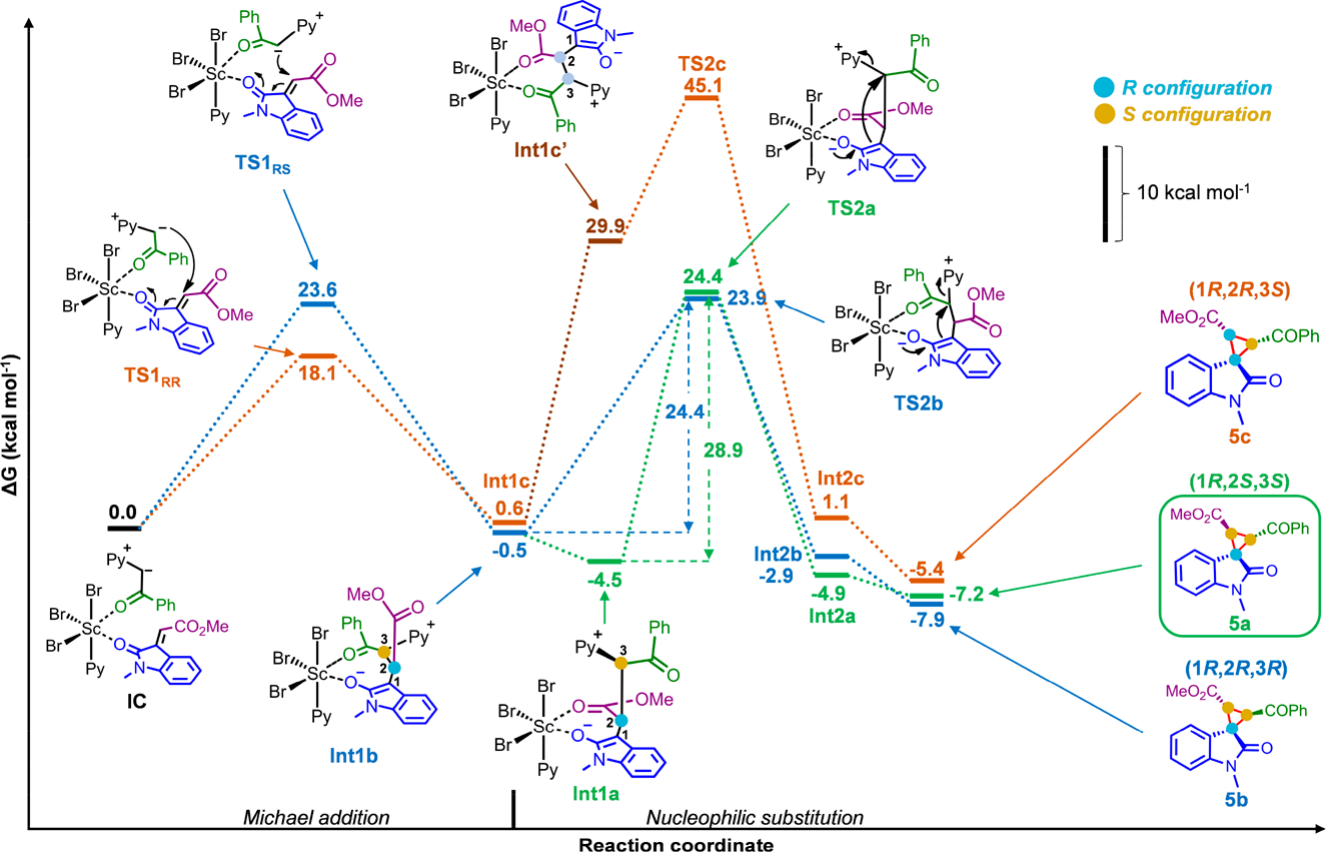
Minimum energy reaction pathway calculated with the PCM_pyridine_/ωB97X-D/6-31G(d,p) and LanL2DZ effective core potential for Sc and Br atoms. The chemical structure of relevant stationary points is depicted.

The subsequent diastereoselective ring-closing step takes place from enolates **Int1a–c**, where atom C1 undergoes a nucleophilic attack to atom C3 with the simultaneous displacement of pyridine (**TS2a-c**). Importantly, for this reaction to take place, the pyridinium leaving group and the nucleophilic enolate must be in an antiperiplanar conformation. These two reacting groups are in the right orientation in intermediates **Int1a** and **Int1b**, thus being able to undergo substitution directly with affordable intrinsic activation barriers (Δ*G*^⧧^ = 28.9 kcal mol^–1^ for **TS2a**, and Δ*G*^⧧^ = 24.4 kcal mol^–1^ for **TS2b**) to give *trans*-cyclopropane complexes **Int2a** and **Int2b**. Decoordination from scandium leads to the thermodynamically stable and experimentally observed products **5a** (1*R*,2*S*,3*S*) and **5b** (1*R*,2*R*,3*R*), respectively. On the contrary, **Int1c** cannot undergo the substitution directly, and a carbonyl group exchange implying a very unfavorable decoordination of the enolate (blue) (Δ*G* ∼ 30 kcal mol^–1^ for **Int1c′**) must take place before nucleophilic substitution, which as a consequence has a prohibitively high activation barrier (Δ*G*^⧧^ ∼ 45 kcal mol^–1^ for **TS2c**), to give *cis*-cyclopropane complex **Int2c**; this very unfavorable calculated pathway explains why product **5c** (1*R*,2*R*,3*S*), which is also less thermodynamically stable than both *trans* isomers, is not obtained experimentally. No reaction pathway toward stereoisomer **5d** (1*R*,2*S*,3*R*), also experimentally unobserved, was calculated. The formation of this stereoisomer would require the simultaneous coordination of the three carbonyl groups (i.e., isatin enolate, aryl ketone, and ester) to the metal center, which in turn would require olefin to have a *Z* configuration. Because olefin **25** is formed exclusively as an *E* isomer under our reaction conditions (see the Supporting Information), formation of compound **5d** was excluded from our calculations.

The lower activation energy of **TS2b**, which is rate-determining and ultimately responsible for the high diastereoselectivity observed experimentally under kinetic conditions, can be attributed to the higher electrophilicity of C3 upon coordination of the phenyl ketone. Hence, the metal center exerts a templating effect by coordinating the reacting fragments in a productive and energetically favored orientation, increasing both reactivity and stereoselectivity in the key ring-closing step.

In summary, REM triflate salts, particularly scandium triflate, allowed the development of a new, simple route for the multicomponent synthesis of disubstituted spirocyclopropyl oxindole derivatives from isatins in excellent yields and very high diastereoselectivity toward one specific *trans* isomer. DFT calculations support the proposed reaction mechanism and provide an explanation for such selectivity. This newly developed protocol is proposed as a valuable entry to diastereopure spirooxindolic compounds with biological potential.

## Data Availability

The data underlying this study are available in the published article and its Supporting Information.

## Supporting Information Available

Experimental details and characterization data of compounds, reaction optimization, and DFT calculations (PDF)
