# Supplementary material for: Highly Diastereoselective Multicomponent Synthesis of Spirocyclopropyl Oxindoles Enabled by Rare-Earth Metal Salts
Source: Org Lett. 2023 Apr 26;25(17):3001–6. doi: 10.1021/acs.orglett.3c00772 (PMC10167684; doi:10.1021/acs.orglett.3c00772)
Supplement: Supplementary file 1 — ol3c00772_si_001.pdf [file ol3c00772_si_001.pdf]

Supporting information

Highly Diastereoselective Multicomponent Synthesis of Spirocyclopropyl Oxindoles Enabled by Rare-Earth Metal Salts

Matteo A. Tallarida,* Fabrizio Olivito, Claudio D. Navo, Vincenzo Algieri, Antonio Jiritano, Paola Costanzo, Ana Poveda, Maria J. Moure, Jesús Jiménez-Barbero, Loredana Maiuolo, Gonzalo Jiménez-Osés,* and Antonio De Nino.*

Matteo A. Tallarida – *Department of Chemistry and Chemical Technologies, University of Calabria, Via P. Bucci, Cubo 12C, 87036, Rende, Italy. Center for Cooperative Research in Biosciences (CIC bioGUNE), Basque Research and Technology Alliance (BRTA), Bizkaia Technology Park, Building 800, 48160 Derio, Spain.* Email: matteoa.tallarida@unical.it

Gonzalo Jiménez-Osés – *Center for Cooperative Research in Biosciences (CIC bioGUNE), Basque Research and Technology Alliance (BRTA), Bizkaia Technology Park, Building 800, 48160 Derio, Spain – Ikerbasque, Basque Foundation for Science, 48013 Bilbao, Spain.* Email: gjoses@cicbiogune.es

Antonio De Nino – *Department of Chemistry and Chemical Technologies, University of Calabria, Via P. Bucci, Cubo 12C, 87036, Rende, Italy*. Email: *denino@unical.it*

Fabrizio Olivito, Vincenzo Algieri, Antonio Jiritano, Paola Costanzo, Loredana Maiuolo – *Department of Chemistry and Chemical Technologies, University of Calabria, Via P. Bucci, Cubo 12C, 87036, Rende, Italy.*

Claudio D. Navo, Ana Poveda, Maria J. Moure – *Center for Cooperative Research in Biosciences (CIC bioGUNE), Basque Research and Technology Alliance (BRTA), Bizkaia Technology Park, Building 800, 48160 Derio, Spain.*

Jesús Jiménez-Barbero – *Center for Cooperative Research in Biosciences (CIC bioGUNE), Basque Research and Technology Alliance (BRTA), Bizkaia Technology Park, Building 800, 48160 Derio, Spain. – Ikerbasque, Basque Foundation for Science, 48013 Bilbao, Spain. – Department of Organic Chemistry II, Faculty of Science & Technology, University of the Basque Country, Leioa 48940, Bizkaia, Spain. – Centro de Investigacion Biomedica En Red de Enfermedades Respiratorias, 28029 Madrid, Spain.*

Table of Contents

**Section**		**Pages**
1	General information	S3
2	Reagents	S4
3	General procedure for the synthesis of spirocyclopropyl oxindoles	S5
4	Characterization of the isolated products	S6
5	Computational studies	S13
6	^{1}H and ^{13}C NMR spectra of compound **25**	S29
7	GC chromatograms and MS spectra of the model reaction crude using different REM triflate salts	S31
8	HR-MS spectra of the isolated products	S34
9	^{1}H NMR spectra of reaction crudes (optimized conditions)	S39
10	^{1}H, ^{13}C, NOE and NOESY NMR spectra of the isolated products	S55
11	References	S103

1. General information

Commercial reagents (2.2 and 2.3 of *Reagents* section) were purchased from Merck or Thermo Fischer and used without any purification. Solvents were purified and dried through classical procedures. Reactions were monitored by TLC using silica plates 60-F264 commercially available form Merck (Darmstadt, Germany). Flash chromatography separations were carried out with silica gel columns. ^{1}H, ^{13}C, and NOESY nuclear magnetic resonance (NMR) spectra were recorded on a Bruker NMR spectrometer AVANCE 500 MHz Wide Bore (operating at 500 and 126 MHz, respectively), a Bruker NMR spectrometer 400 MHz (operating at 400 and 101 MHz, respectively) or a Bruker ACP 300 Mhz (operating at 300 and 75 MHz, respectively) in $CDCl_3$ or DMSO-d_6 using tetramethylsilane (TMS) as the internal standard. Chemical shifts are given in parts per million and coupling constants in Hertz. The signal splitting patterns were described as s = singlet, d = doublet, t = triplet, q = quartet, p = pentuplet, dd = doublet of doublet, dt = doublet of triplet, td = triplet of doublet, tt = triplet of triplet, ddd = doublet of doublet of doublet, m = multiplet, qd = quadruplet of doublet and ddq = doublet of doublet of doublet. Structural assignments were made with additional information from gCOSY, gHSQC, and gHMBC experiments.
HR-MS spectra were acquired with a Bruker Compact Q-TOF instrument (Bruker, Billerica, MA, USA).
Ultra-performance liquid chromatography (UPLC, AQUITY, Waters Inc., Manchester, UK) with a 1x100 mm, 1.7 µM BEH-C18 column (Waters Inc.) was used to analyze the samples for mass injection. The column was thermostated at 40 °C. The mobile phases consisted of A: 99.9%(v/v) water, 0.1%(v/v) formic acid (FA) and B: 99.9%(v/v) ACN and 0.1%(v/v) FA. The gradient was as follows: from 95%A to 1%A in 4 minutes, stable at 1%A for 0.8 minutes, back to 95%A in 0.2 minutes. Total run time per sample was 7 minutes, the flow was set to 140 µL/min and the injection volume was 1 µL. Exact masses were determined on a SYNAPT G2S Time-of-Flight instrument (Waters Inc.) in positive ionization mode with an electrospray ionization source. The instrument was operated with a capillary voltage of 1 kV, sampling cone voltage of 25 V, source offset of 80 V, source temperature of 120 °C, desolvation temperature of 450 °C, cone gas flow of 5 L/h, desolvation gas flow of 600 L/h and nebulizer pressure of 6 Bar. In order to guarantee accurate mass, the instrument was tuned to a resolution of 21.000 for m/z 556.2771 (Leu-Enk) and calibrated for the mass range between 50 and 1200 Da. In order to correct for mass drift during analysis, every 1.8 minutes a lock mass was recorded (Leu-Enk) and spectral peaks were corrected automatically for changes in this lock mass. Scan time was set to 0.2 scans/s. Results were obtained from MS/MS experiments on the expected m/z of the analyte ($[M+H]^+$). Collision energy was applied in the trap with a ramp from 5V to 30V to obtain fragmentation data from the specified pseudomolecular ion.
All structure formula reported in this ESI with a specific stereochemistry are only the representation of one arbitrary enantiomer of the racemic mixture of the major diastereoisomer.
Known compounds are referenced.

2. Reagents

2.1 N-alkyl isatin derivatives

Isatin derivatives **1a-1k** are known compounds and were synthesized according to the literature procecedure.[1–7] Compound **1l** has been synthesized using the same procedure as for compound **1k**.

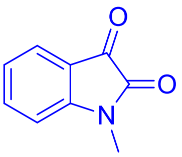

1a[1,2,6]

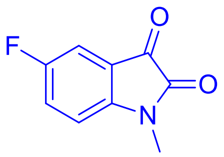

1b[6,7]

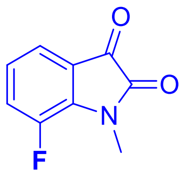

1c[6]

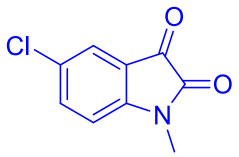

1d[1,7]

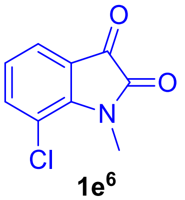

1e[6]

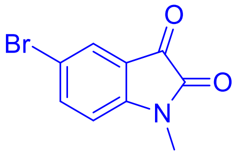

1f[1,6,7]

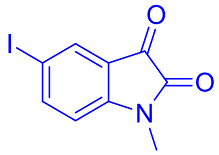

1g[1,6]

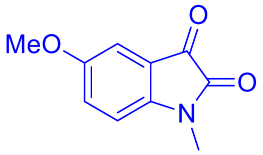

1h[6,7]

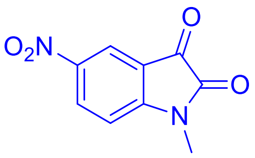

1i[2,6]

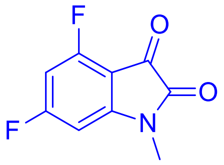

1j[5]

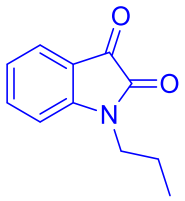

1k[7]

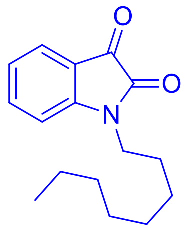

1l[7]

2.2 Phosphonate derivatives

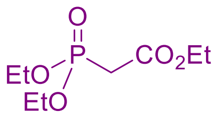

2a

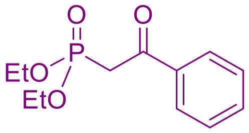

2b

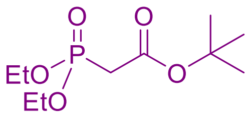

2c

2.3 2-Bromoacetophenone derivatives

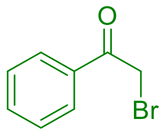

3a

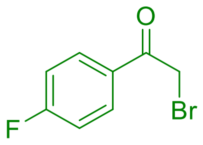

3b

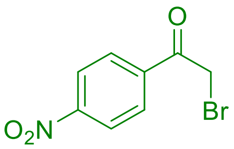

3c

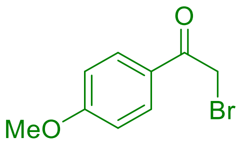

3d

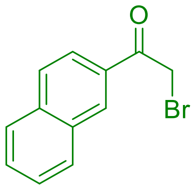

3e

3. General procedure for the synthesis of spirocyclopropyl oxindoles

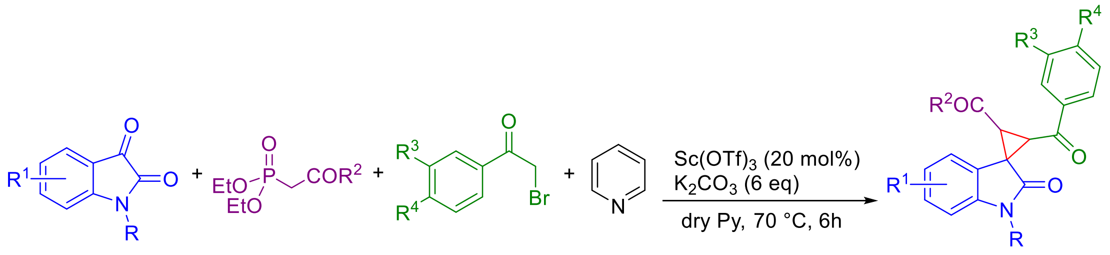

1 **2** **3** **4** **5-22**

In a 10 mL three-necked round-bottomed flask, equipped with bubble condenser and magnetic stir bar, a mixture of the chosen *N*-alkylisatin derivative **1** (1 eq), phosphonate **2** (1 eq), 2-bromoacetophenone derivative **3** (2.5 eq), *dry* pyridine **4** (3.5 eq), Sc(OTf)$_3$ (20 mol%), and K_2CO_3 (6 eq) was placed. Then, other 5 mL of dry pyridine were added as solvent. The reaction was warmed at 70 °C for 6 h by an oil bath. The crude was evaporated under vacuum through an azeotrope made up by adding toluene to the reaction mixture (3x5 mL). The residues of toluene were evaporated in the same fashion, using an azeotrope made up by adding ethanol to the mixture (3x5 mL). The crude was then extracted through a liquid/liquid separation, using ethyl acetate (EtOAc), water, and brine, and purified on a flash silica gel column by using EtOAc/hexane (1:4 v/v) to obtain the desired product (**5-22**).

3.1 Gram scale procedure

In a 250 mL three-necked round-bottomed flask, equipped with bubble condenser and magnetic stir bar, a mixture of **1a** (1 g, 6.2 mmol, 1 eq), **2a** (1.23 mL, 6.2 mmol, 1 eq,), **3a** (3.09 g, 15.5 mmol, 2.5 eq), **4** (1.75 mL, 21.7 mmol, 3.5 eq), Sc(OTf)$_3$ (0.61 g, 1.24 mmol, 20 mol%), K_2CO_3 (5.14 g, 37.2 mmol, 6 eq), and *dry* Py (1.72 mL, 21.7 mmol, 3.5 eq) were placed. Then, other 100 mL of dry pyridine were added as solvent. The reaction was warmed at 70 °C for 6 h by an oil bath. The crude was evaporated under vacuum through an azeotrope prepared by adding toluene to the reaction mixture (3x50 mL). The residues of toluene were evaporated in the same fashion, using an azeotrope prepared by adding ethanol to the mixture (3x20 mL). The crude was then extracted through a liquid/liquid separation, using ethyl acetate (EtOAc), water, and brine and purified on a flash silica gel column by using EtOAc/hexane (1:4 v/v) to obtain 2.40 gr of desired product (**5**) in 90% yield.

3.2 Procedure for the synthesis of spirocyclopropyl derivative with propachlor 24

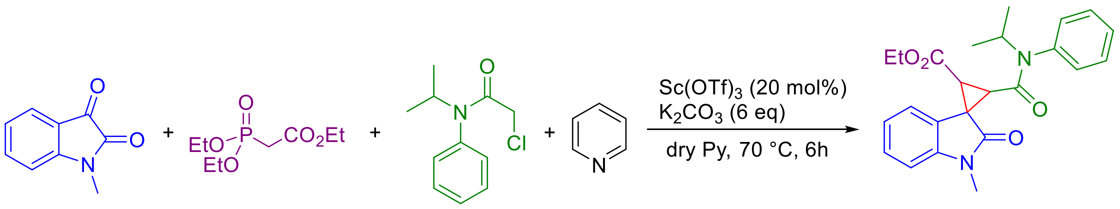

1a **2a** **23** **4** **24**

In a 10 mL three-necked round-bottomed flask, equipped with bubble condenser and magnetic stir bar, a mixture of **1a** (1 eq), **2a** (1 eq), propachlor **23** (1 eq), **4** (3.5 eq), Sc(OTf)$_3$ (20 mol%), and K_2CO_3 (6 eq) were placed. The reaction was warmed at 70 °C for 6 h by an oil bath. The crude was evaporated under vacuum through an azeotrope made up by adding toluene to the reaction mixture (3x5 mL). The residues of toluene were evaporated in the same fashion, using an azeotrope made up by adding ethanol to the mixture (3x5 mL). The crude was then extracted through a liquid/liquid separation, using ethyl acetate (EtOAc), water, and brine, and purified on a flash silica gel column by using EtOAc/hexane (1:4 v/v) to obtain 0.17 gr of desired product (**24**) in 67% yield.

4. Characterization of the isolated diastereoisomers

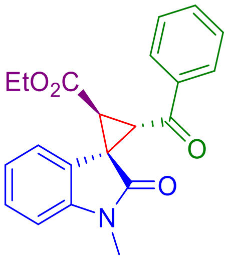

5a

Ethyl-3-benzoyl-1'-methyl-2'-oxospiro[cyclopropane-1,3'-indoline]-2-carboxylate 5a (major diastereoisomer): obtained using *N*-methylisatin **1a**, triethyl phosphonoacetate **2a**, and 2-bromoacetophenone **3a**. Purified by normal phase cromatography (EtOAc:Hexane, 1:4 v/v) to yield **5a** as major isomer (33 mg, 95%, **5a**:**5b**: 91:9 *dr*), as red solid. ^{1}H NMR (400 MHz, $CDCl_3$) δ 7.95 – 7.93 (m, 2H), 7.60 – 7.51 (m, 1H), 7.43 (t, *J* = 7.7 Hz, 2H), 7.25 (dd, *J* = 7.7, 1.2 Hz, 1H), 7.19 (dd, *J* = 7.6, 0.7 Hz, 1H), 6.99 (td, *J* = 7.6, 1.0 Hz, 1H), 6.87 (d, *J* = 7.8 Hz, 1H), 4.35 – 4.24 (m, 2H), 4.23 (d, *J* = 7.7 Hz, 1H), 3.63 (d, *J* = 7.7 Hz, 1H), 3.32 (s, 3H), 1.31 (t, *J* = 7.1 Hz, 3H). ^{13}C NMR (126 MHz, $CDCl_3$) δ 191.7, 171.8, 166.2, 144.1, 136.6, 133.8, 128.7, 128.6, 128.4, 124.2, 122.6, 122.5, 108.2, 61.7, 39.6, 38.9, 35.0, 26.8, 14.2. HRMS (UPLC-TOF) *m/z*: $[M+H]^+$ Calcd for $C_{21}H_{20}NO_4$: 350.1387; Found: 350.1390, RT=3.37.

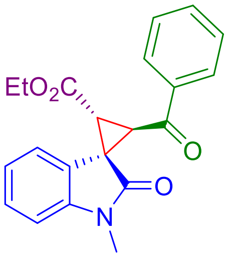

5b

Ethyl-3-benzoyl-1'-methyl-2'-oxospiro[cyclopropane-1,3'-indoline]-2-carboxylate 5b (minor isomer): ^{1}H NMR (400 MHz, $CDCl_3$) δ 7.79 (dd, *J* = 8.4, 1.3 Hz, 2H), 7.62 – 7.48 (m, 2H), 7.45 – 7.34 (m, 3H), 7.15 (td, *J* = 7.7, 1.1 Hz, 1H), 6.94 (d, *J* = 7.8 Hz, 1H), 4.37 – 4.08 (m, 2H), 3.85 (d, *J* = 7.8 Hz, 1H), 3.51 (d, *J* = 7.8 Hz, 1H), 3.16 (s, 3H), 1.26 (t, *J* = 7.1 Hz, 3H). ^{13}C NMR (126 MHz, $CDCl_3$) δ δ 189.9, 171.0, 167.7, 144.6, 136.1, 133.6, 128.7, 128.7, 128.4, 124.1, 122.7, 122.6, 108.3, 61.7, 39.6, 39.0, 35.2, 26.6, and 14.1. HRMS (UPLC-TOF) *m/z*: $[M+H]^+$ Calcd for $C_{21}H_{20}NO_4$: 350.1387; Found: 350.1390, RT=3.34.

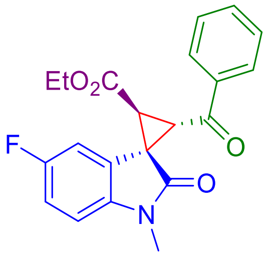

6

Ethyl-3-benzoyl-5'-fluoro-1'-methyl-2'-oxospiro[cyclopropane-1,3'-indoline]-2-carboxylate 6 (major diastereoisomer): obtained using 5-fluoro-*N*-methylisatin **1b**, triethyl phosphonoacetate **2a**, and 2-bromoacetophenone **3a**. Purified by normal phase cromatography (EtOAc:Hexane, 1:4 v/v) to yield **6** as major isomer (35 mg, 95%, 90:10 *dr*), as brown oil. ^{1}H NMR (400 MHz, $CDCl_3$) δ 7.96 (dd, *J* = 8.5, 1.3 Hz, 2H), 7.73 – 7.51 (m, 1H), 7.53 – 7.39 (m, 2H), 7.07 – 6.89 (m, 2H), 6.78 (dd, *J* = 8.5, 4.2 Hz, 1H), 4.40 – 4.15 (m, 3H), 3.60 (d, *J* = 7.8 Hz, 1H), 3.31 (s, 3H), 1.31 (t, *J* = 7.1 Hz, 3H). ^{13}C NMR (126 MHz, $CDCl_3$) δ 191.5, 171.5, 165.9, 160.0, 158.1, 140.1, 136.5, 134.0, 128.7 (d, J = 23.2 Hz), 125.8 (d, J = 9.4 Hz), 114.7 (d, J = 23.9 Hz), 111.0 (d, J = 26.6 Hz), 108.6 (d, J = 8.5 Hz), 61.8, 38.9, 35.4, 29.3, 26.9, 14.2. HRMS (UPLC-TOF) *m/z*: $[M+H]^+$ Calcd for $C_{21}H_{19}FNO_4$: 368.1298; Found: 368.1299, RT=3.44.

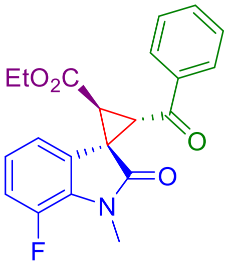

7

Ethyl-3-benzoyl-7'-fluoro-1'-methyl-2'-oxospiro[cyclopropane-1,3'-indoline]-2-carboxylate 7 (major diastereoisomer): obtained using 7-fluoro-*N*-methylisatin **1c**, triethyl phosphonoacetate **2a**, and 2-bromoacetophenone **3a**. Purified by normal phase cromatography (EtOAc:Hexane, 1:4 v/v) to yield **7** as major isomer (33 mg, 90%, 91:9 *dr*), as brown oil. ^{1}H NMR (400 MHz, $CDCl_3$) δ 8.16 – 7.73 (m, 2H), 7.67 – 7.51 (m, 1H), 7.52 – 7.39 (m, 2H), 7.09 – 6.81 (m, 3H), 4.28 (qt, *J* = 7.1, 3.5 Hz, 2H), 4.22 (d, *J* = 7.8 Hz, 1H), 3.61 (d, *J* = 7.8 Hz, 1H), 3.54 (d, *J* = 2.8 Hz, 3H), 1.32 (t, *J* = 7.1 Hz, 3H). ^{13}C NMR (126 MHz, $CDCl_3$) δ 191.4, 171.5, 165.9, 148.8, 146.8, 136.5, 134.0, 128.8, 128.6, 127.0 (d, J = 3.5 Hz), 123.1 (d, J = 6.5 Hz), 118.3 (d, J = 3.3 Hz), 116.4 (d, J = 19.0 Hz), 61.8, 39.4, 35.4, 29.4, 29.4, 14.2. HRMS (UPLC-TOF) *m/z*: $[M+H]^+$ Calcd for $C_{21}H_{19}FNO_4$: 368.1293; Found: 368.1295, RT=3.51.

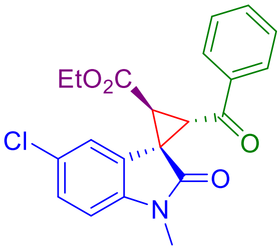

8

Ethyl-3-benzoyl-5'-chloro-1'-methyl-2'-oxospiro[cyclopropane-1,3'-indoline]-2-carboxylate 8 (major diastereoisomer): obtained using 5-chloro-*N*-methylisatin **1d**, triethyl phosphonoacetate **2a**, and 2-bromoacetophenone **3a**. Purified by normal phase cromatography (EtOAc:Hexane, 1:4 v/v) to yield **8** as major isomer (36 mg, 93%, 90:10 *dr*), as brown oil. ^{1}H NMR (400 MHz, $CDCl_3$) δ 8.07 – 7.84 (m, 2H), 7.67 – 7.52 (m, 1H), 7.51 – 7.41 (m, 2H), 7.27 – 7.20 (m, 2H), 6.79 (dd, *J* = 8.1, 0.6 Hz, 1H), 4.36 – 4.14 (m, 3H), 3.61 (d, *J* = 7.8 Hz, 1H), 3.30 (s, 3H), 1.31 (t, *J* = 7.1 Hz, 3H). ^{13}C NMR (101 MHz, $CDCl_3$) δ 191.4, 171.4, 165.9, 142.7, 136.5, 134.0, 128.8, 128.7, 128.3, 128.2, 125.9, 123.1, 109.1, 61.9, 39.4, 39.0, 35.4, 26.9, 14.2. HRMS (UPLC-TOF) *m/z*: $[M+H]^+$ Calcd for $C_{21}H_{19}ClNO_4$: 384.0997; Found: 384.0999, RT=3.62.

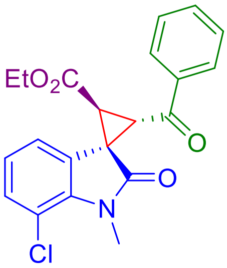

9

Ethyl-3-benzoyl-7'-chloro-1'-methyl-2'-oxospiro[cyclopropane-1,3'-indoline]-2-carboxylate 9 (major diastereoisomer): obtained using 7-chloro-*N*-methylisatin **1e**, triethyl phosphonoacetate **2a**, and 2-bromoacetophenone **3a**. Purified by normal phase cromatography (EtOAc:Hexane, 1:4 v/v) to yield **9** as major isomer (34 mg, 90%, 91:9 *dr*), as brown oil. ^{1}H NMR (400 MHz, $CDCl_3$) δ 7.92 (dd, *J* = 8.5, 1.3 Hz, 2H), 7.68 – 7.51 (m, 1H), 7.48 – 7.40 (m, 2H), 7.17 (dd, *J* = 8.2, 1.2 Hz, 1H), 7.05 (dd, *J* = 7.6, 1.2 Hz, 1H), 6.88 (dd, *J* = 8.2, 7.6 Hz, 1H), 4.27 (qd, *J* = 7.1, 3.1 Hz, 2H), 4.22 (d, *J* = 7.9 Hz, 1H), 3.69 (s, 3H), 3.60 (d, *J* = 7.9 Hz, 1H), 1.31 (t, *J* = 7.1 Hz, 3H). ^{13}C NMR (101 MHz, $CDCl_3$) δ 191.3, 172.1, 165.9, 139.9, 136.4, 134.0, 130.7, 128.8, 128.6, 126.9, 123.3, 120.8, 115.8, 61.9, 39.7, 39.1, 35.4, 30.3, 14.2. HRMS (UPLC-TOF) *m/z*: $[M+H]^+$ Calcd for $C_{21}H_{19}ClNO_4$: 384.0997; Found: 384.0995, RT=3.70.

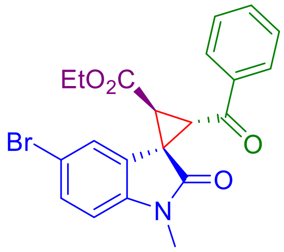

10

Ethyl-3-benzoyl-5'-bromo-1'-methyl-2'-oxospiro[cyclopropane-1,3'-indoline]-2-carboxylate 10 (major diastereoisomer): obtained using 5-bromo-*N*-methylisatin **1f**, triethyl phosphonoacetate **2a**, and 2-bromoacetophenone **3a**. Purified by normal phase cromatography (EtOAc:Hexane, 1:4 v/v) to yield **10** as major isomer (38 mg, 92%, 90:10 *dr*), as brown oil. ^{1}H NMR (400 MHz, $CDCl_3$) δ 7.98 – 7.94 (m, 2H), 7.62 – 7.55 (m, 1H), 7.51 – 7.43 (m, 2H), 7.40 (dd, J = 8.3, 2.0 Hz, 1H), 7.36 (d, J = 2.0 Hz, 1H), 6.74 (d, J = 8.3 Hz, 1H), 4.33 – 4.21 (m, 3H), 3.61 (d, J = 7.7 Hz, 1H), 3.30 (s, 3H), 1.31 (t, J = 7.2 Hz, 3H). ^{13}C NMR (126 MHz, $CDCl_3$) δ 191.4, 171.3, 165.9, 143.2, 136.5, 134.0, 131.3, 128.8, 128.7, 126.2, 125.9, 115.4, 109.6, 61.9, 39.0, 35.5, 29.3, 26.9, 14.1. HRMS (UPLC-TOF) *m/z*: $[M+H]^+$ Calcd for $C_{21}H_{19}BrNO_4$: 428.0492; Found: 428.0503, RT=3.65.

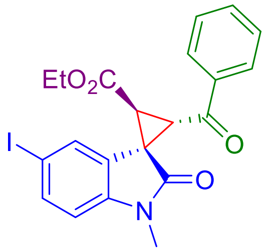

11

Ethyl-3-benzoyl-5'-iodo-1'-methyl-2'-oxospiro[cyclopropane-1,3'-indoline]-2-carboxylate 11 (major diastereoisomer): obtained using 5-iodo-*N*-methylisatin **1g**, triethyl phosphonoacetate **2a**, and 2-bromoacetophenone **3a**. Purified by normal phase cromatography (EtOAc:Hexane, 1:4 v/v) to yield **11** as major isomer (43 mg, 90%, 90:10 *dr*), as brown oil. ^{1}H NMR (400 MHz, $CDCl_3$) δ 8.00 – 7.90 (m, 2H), 7.58 (ddd, J = 7.2, 6.1, 1.5 Hz, 2H), 7.51 (d, J = 1.7 Hz, 1H), 7.46 (t, J = 7.7 Hz, 2H), 6.64 (d, J = 8.2 Hz, 1H), 4.35 – 4.15 (m, 3H), 3.59 (d, J = 7.8 Hz, 1H), 3.29 (s, 3H), 1.31 (t, J = 7.1 Hz, 3H). ^{13}C NMR (126 MHz, $CDCl_3$) δ 191.4, 171.1, 165.9, 143.8, 137.2, 136.5, 134.0, 131.3, 128.8, 128.6, 126.5, 110.2, 85.2, 61.9, 39.0, 35.4, 29.7, 26.8, 14.1. HRMS (UPLC-TOF) *m/z*: $[M+H]^+$ Calcd for $C_{21}H_{19}INO_4$: 476.0353; Found: 476.0356, RT=3.71.

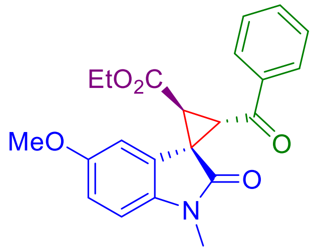

12

Ethyl-3-benzoyl-5'-methoxy-1'-methyl-2'-oxospiro[cyclopropane-1,3'-indoline]-2-carboxylate 12 (major diastereoisomer): obtained using 5-methoxy-*N*-methylisatin **1h**, triethyl phosphonoacetate **2a**, and 2-bromoacetophenone **3a**. Purified by normal phase cromatography (EtOAc:Hexane, 1:4 v/v) to yield **12** as major isomer (36 mg, 95%, 94:6 *dr*), as brown oil. ^{1}H NMR (400 MHz, $CDCl_3$) δ 7.95 (dd, J = 8.5, 1.3 Hz, 2H), 7.62 – 7.51 (m, 1H), 7.47 – 7.40 (m, 2H), 6.86 – 6.71 (m, 3H), 4.38 – 4.24 (m, 2H), 4.22 (d, J = 7.7 Hz, 1H), 3.76 (s, 3H), 3.59 (d, J = 7.8 Hz, 1H), 3.29 (s, 3H), 1.31 (t, J = 7.2 Hz, 3H). ^{13}C NMR (126 MHz, $CDCl_3$) δ 191.7, 171.5, 166.2, 155.9, 137.7, 136.7, 133.8, 128.7, 128.6, 125.4, 113.4, 109.5, 108.6, 61.7, 55.9, 39.8, 38.9, 35.1, 26.9, 14.2. HRMS (UPLC-TOF) *m/z*: $[M+H]^+$ Calcd for $C_{22}H_{22}NO_5$: 380.1492; Found: 380.1493, RT=3.29.

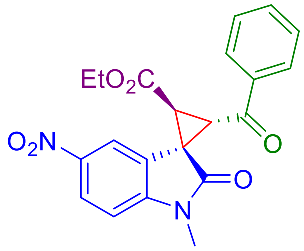

13

Ethyl-3-benzoyl-1'-methyl-5'-nitro-2'-oxospiro[cyclopropane-1,3'-indoline]-2-carboxylate 13 (major diastereoisomer): obtained using 5-nitro-*N*-methylisatin **1i**, triethyl phosphonoacetate **2a**, and 2-bromoacetophenone **3a**. Purified by normal phase cromatography (EtOAc:Hexane, 1:4 v/v) to yield **13** as major isomer (18 mg, 45%, 90:10 *dr*), as pale yellow solid. ^{1}H NMR (500 MHz, $CDCl_3$) δ 8.50 (d, *J* = 2.3 Hz, 1H), 8.37 (dd, *J* = 8.7, 2.3 Hz, 1H), 7.83 – 7.73 (m, 2H), 7.60 – 7.51 (m, 1H), 7.43 – 7.35 (m, 2H), 7.02 (d, *J* = 8.7 Hz, 1H), 4.33 – 4.16 (m, 2H), 3.99 (d, *J* = 8.0 Hz, 1H), 3.55 (d, *J* = 8.0 Hz, 1H), 3.23 (s, 3H), 1.29 (t, *J* = 7.1 Hz, 3H). ^{13}C NMR (126 MHz, $CDCl_3$) δ 188.8, 171.2, 167.0, 149.8, 143.4, 135.6, 133.9, 128.9, 128.3, 125.7, 124.9, 118.9, 107.9, 62.2, 40.1, 38.2, 36.0, 27.1, 14.1. HRMS (ESI-QTOF) *m/z*: $[M+H]^+$ Calcd for $C_{21}H_{19}N_2O_6$: 395.1238; Found: 395.1251.

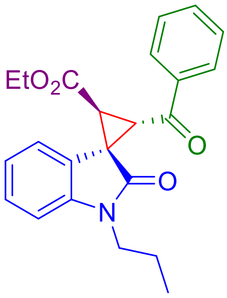

15

Ethyl-3-benzoyl-2'-oxo-1'-propylspiro[cyclopropane-1,3'-indoline]-2-carboxylate 15 (major diastereoisomer): obtained using *N*-propylisatin **1k**, triethyl phosphonoacetate **2a**, and 2-bromoacetophenone **3a**. Purified by normal phase cromatography (EtOAc:Hexane, 1:4 v/v) to yield **15** (35 mg, 93%, 91:9 *dr*), as red solid. ^{1}H NMR (500 MHz, $CDCl_3$) δ 7.90 (dd, *J* = 8.5, 1.3 Hz, 2H), 7.55 – 7.46 (m, 1H), 7.42 – 7.35 (m, 2H), 7.21 (td, *J* = 7.7, 1.2 Hz, 1H), 7.16 – 7.12 (m, 1H), 6.93 (td, *J* = 7.7, 1.0 Hz, 1H), 6.86 (d, *J* = 7.9 Hz, 1H), 4.24 (qd, *J* = 7.2, 1.7 Hz, 2H), 4.19 (d, *J* = 7.7 Hz, 1H), 3.78 (dd, *J* = 7.6, 6.6 Hz, 2H), 3.61 (d, *J* = 7.7 Hz, 1H), 1.74 (h, *J* = 7.3 Hz, 2H), 1.27 (t, *J* = 7.1 Hz, 3H), 0.96 (t, *J* = 7.4 Hz, 3H). ^{13}C NMR (126 MHz, $CDCl_3$) δ 190.7, 170.8, 165.2, 142.5, 135.6, 132.8, 127.7, 127.5, 127.2, 123.3, 121.4, 121.3, 107.5, 60.6, 41.0, 38.4, 38.0, 33.9, 19.8, 13.1, 10.2. HRMS (ESI-QTOF) *m/z*: $[M+H]^+$ Calcd for $C_{23}H_{24}NO_4$: 378.1700; Found: 378.1708.

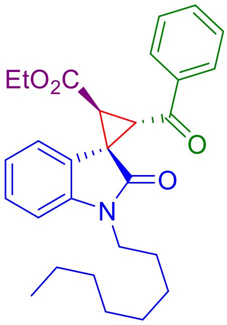

16

Ethyl-3-benzoyl-1'-octyl-2'-oxospiro[cyclopropane-1,3'-indoline]-2-carboxylate 16 (major diastereoisomer): obtained using *N*-octylisatin **1l**, triethyl phosphonoacetate **2a**, and 2-bromoacetophenone **3a**. Purified by normal phase cromatography (EtOAc:Hexane, 1:4 v/v) to yield **16** (41 mg, 91%, 90:10 *dr*), as

red solid. ^{1}H NMR (500 MHz, $CDCl_3$) δ 7.91 (dd, *J* = 8.5, 1.2 Hz, 2H), 7.56 – 7.50 (m, 1H), 7.43 – 7.37 (m, 2H), 7.23 (td, *J* = 7.7, 1.2 Hz, 1H), 7.17 – 7.14 (m, 1H), 6.95 (td, *J* = 7.6, 1.0 Hz, 1H), 6.87 (d, *J* = 7.7 Hz, 1H), 4.30 – 4.22 (m, 2H), 4.20 (d, *J* = 7.7 Hz, 1H), 3.87 – 3.74 (m, 2H), 3.62 (d, *J* = 7.7 Hz, 1H), 1.71 (p, *J* = 7.3 Hz, 2H), 1.45 – 1.18 (m, 13H), 0.97 – 0.82 (m, 3H). ^{13}C NMR (126 MHz, $CDCl_3$) δ 190.7, 170.6, 165.2, 142.5, 135.6, 132.8, 127.7, 127.5, 127.2, 123.3, 121.5, 121.3, 107.5, 60.6, 39.5, 38.4, 38.0, 33.8, 30.8, 28.21, 28.18, 26.5, 25.8, 21.6, 13.1, 13.1. HRMS (ESI-QTOF) *m/z*: $[M+H]^+$ Calcd for $C_{28}H_{34}NO_4$: 448.2482; Found: 448.2505.

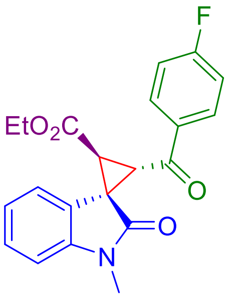

17

Ethyl-3-(4-fluorobenzoyl)-1'-methyl-2'-oxospiro[cyclopropane-1,3'-indoline]-2-carboxylate 17 (major diastereoisomer): obtained using *N*-methyilisatin **1a**, triethyl phosphonoacetate **2a**, and 2-bromo-3′-fluoroacetophenone **3a**. Purified by normal phase cromatography (EtOAc:Hexane, 1:4 v/v) to yield **17** as major isomer (33 mg, 77%, 90:10 *dr*), as brown solid. ^{1}H NMR (300 MHz, $CDCl_3$) δ 8.00 – 7.94 (m, 2H), 7.27 (td, *J* = 7.7, 1.2 Hz, 1H), 7.16 (dd, *J* = 7.6, 1.3 Hz, 1H), 7.12 – 7.07 (m, 2H), 6.99 (td, *J* = 7.7, 1.0 Hz, 1H), 6.88 (d, *J* = 7.9 Hz, 1H), 4.33-4.21 (m, 2H), 4.17 (d, *J* = 7.7 Hz, 1H), 3.61 (d, *J* = 7.7 Hz, 1H), 3.32 (s, 3H), 1.30 (t, *J* = 7.1 Hz, 3H). ^{13}C NMR (101 MHz, MeOD) δ 210.8, 190.1, 171.7, 166.1, 144.1, 131.3, 131.2, 128.5, 122.6, 122.4, 116.1, 115.9, 108.3, 61.8, 39.5, 38.8, 35.0, 26.8, 14.2. HRMS (UPLC-TOF) *m/z*: $[M+H]^+$ Calcd for $C_{21}H_{19}FNO_4$: 368.1298; Found: 368.1302, RT=3.43.

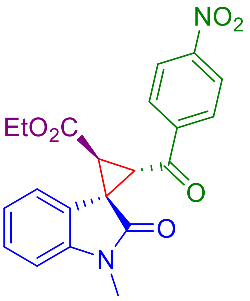

18

Ethyl-1'-methyl-3-(4-nitrobenzoyl)-2'-oxospiro[cyclopropane-1,3'-indoline]-2-carboxylate 18 (major diastereoisomer): obtained using *N*-methyilisatin **1a**, triethyl phosphonoacetate **2a**, and 2-bromo-3′-nitroacetophenone **3b**. Purified by normal phase cromatography (EtOAc:Hexane, 1:4 v/v) to yield **18** as major isomer (6 mg, 16%, 90:10 *dr*), as brown solid. ^{1}H NMR (500 MHz, $CDCl_3$) δ 8.25 – 8.20 (m, 2H), 7.94 – 7.90 (m, 2H), 7.55 (ddd, *J* = 7.5, 1.3, 0.6 Hz, 1H), 7.42 (td, *J* = 7.8, 1.2 Hz, 1H), 7.16 (td, *J* = 7.7, 1.0 Hz, 1H), 6.95 (dd, *J* = 7.8, 0.8 Hz, 1H), 4.28-4.12 (m, 2H), 3.83 (d, *J* = 7.7 Hz, 1H), 3.48 (d, *J* = 7.8 Hz, 1H), 3.15 (s, 3H), 2.18 (s, 3H). ^{13}C NMR (126 MHz, $CDCl_3$) δ 188.9, 170.8, 167.2, 150.5, 144.4, 140.2, 129.3, 129.1, 124.0, 123.4, 122.9, 122.7, 108.6, 61.9, 39.2, 35.1, 29.6, 26.7, 14.1. HRMS (UPLC-TOF) *m/z*: $[M+H]^+$ Calcd for $C_{21}H_{19}N_2O_6$: 395.1243; Found: 395.1246, RT=3.37.

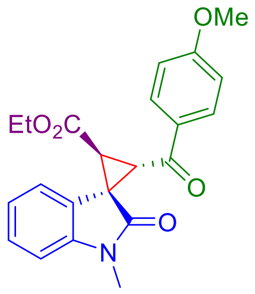

19

Ethyl-3-(4-methoxybenzoyl)-1'-methyl-2'-oxospiro[cyclopropane-1,3'-indoline]-2-carboxylate 19 (major diastereoisomer): obtained using *N*-methyilisatin **1a**, triethyl phosphonoacetate **2a**, and 2-bromo-3′-methoxyacetophenone **3c**. Purified by normal phase cromatography (EtOAc:Hexane, 1:4 v/v) to yield **19** as major isomer (33 mg, 87%, 82:18 *dr*), as white solid. ^{1}H NMR (500 MHz, $CDCl_3$) δ 7.94 – 7.89 (m, 2H), 7.23 (td, *J* = 7.8, 1.2 Hz, 1H), 7.17 (ddd, *J* = 7.7, 1.2, 0.6 Hz, 1H), 6.96 (td, *J* = 7.7, 1.0 Hz, 1H), 6.89 – 6.81 (m, 3H), 4.30 – 4.19 (m, 2H), 4.17 (d, *J* = 7.7 Hz, 1H), 3.82 (s, 3H), 3.60 (d, *J* = 7.8 Hz, 1H), 3.30 (s, 3H), 1.29 (t, *J* = 7.1 Hz, 3H). ^{13}C NMR (126 MHz, $CDCl_3$) δ 189.8, 171.8, 166.3, 164.1, 144.0, 130.9, 129.7, 128.2, 124.3, 122.5, 113.9, 108.1, 61.6, 55.4, 39.3, 38.7, 34.9, 26.7, 14.1, 10.3. HRMS (UPLC-TOF) *m/z*: $[M+H]^+$ Calcd for $C_{22}H_{22}NO_5$: 380.1498; Found: 380.1508, 3.35.

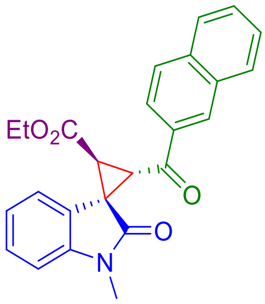

20

Ethyl-2-(2-naphthoyl)-1'-methyl-2'-oxospiro[cyclopropane-1,3'-indoline]-3-carboxylate 20 (major diastereoisomer): obtained using *N*-methyilisatin **1a**, triethyl phosphonoacetate **2a**, and 2-bromo-1-(naphthalen-2-yl)ethan-1-one **3d**. Purified by normal phase cromatography (EtOAc:Hexane, 1:4 v/v) to yield **20** as major isomer (30 mg, 74%, 72:28 *dr*), as red solid. ^{1}H NMR (500 MHz, $CDCl_3$) δ 8.20 (t, *J* = 1.2 Hz, 1H), 7.90 (dd, *J* = 8.6, 1.8 Hz, 1H), 7.82 (ddd, *J* = 8.9, 5.1, 1.0 Hz, 2H), 7.70 (ddq, *J* = 8.2, 1.2, 0.6 Hz, 1H), 7.63 (ddd, *J* = 7.6, 1.3, 0.6 Hz, 1H), 7.57 (ddd, *J* = 8.2, 6.9, 1.3 Hz, 1H), 7.48 (ddd, *J* = 8.2, 6.9, 1.2 Hz, 1H), 7.42 (td, *J* = 7.8, 1.2 Hz, 1H), 7.18 (td, *J* = 7.7, 1.0 Hz, 1H), 6.94 (dt, *J* = 7.7, 0.8 Hz, 1H), 4.33 – 4.10 (m, 2H), 3.97 (d, *J* = 7.9 Hz, 1H), 3.56 (d, *J* = 7.8 Hz, 1H), 3.10 (s, 3H), 1.27 (td, *J* = 7.1, 1.5 Hz, 3H). ^{13}C NMR (126 MHz, $CDCl_3$) δ 189.9, 171.0, 167.7, 144.6, 135.8, 133.4, 132.3, 130.3, 129.6, 128.7, 128.6, 127.7, 126.7, 124.1, 123.8, 122.7, 108.3, 61.7, 39.9, 39.1, 35.2, 26.6, 14.1. HRMS (UPLC-TOF) *m/z*: $[M+H]^+$ Calcd for $C_{25}H_{22}NO_4$: 400.1549; Found: 400.1548, RT=3.72.

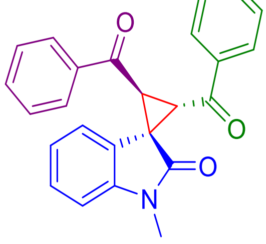

21

(1'-methyl-2'-oxospiro[cyclopropane-1,3'-indoline]-2,3-diyl)bis(phenylmethanone) 21 (major diastereoisomer): obtained using *N*-methyilisatin **1a**, diethyl (2-oxo-2-phenylethyl)phosphonate **2a**, and 2-bromoacetophenone **3a**. Purified by normal phase cromatography (EtOAc:Hexane, 1:4 v/v) to yield **21** as major isomer (32 mg, 83%, 78:22 *dr*), as red solid. ^{1}H NMR (500 MHz, $CDCl_3$) δ 8.04 – 7.94 (m, 2H), 7.89

– 7.78 (m, 2H), 7.58 – 7.49 (m, 2H), 7.47 – 7.41 (m, 2H), 7.41 – 7.35 (m, 3H), 7.32 (td, *J* = 7.8, 1.2 Hz, 1H), 7.07 (td, *J* = 7.7, 1.0 Hz, 1H), 6.93 – 6.85 (m, 1H), 4.46 (d, *J* = 7.9 Hz, 1H), 4.19 (d, *J* = 7.9 Hz, 1H), 3.19 (s, 3H). ^{13}C NMR (126 MHz, $CDCl_3$) δ 192.2, 190.3, 171.2, 144.2, 136.7, 136.1, 133.8, 133.5, 128.7, 128.6, 128.5, 128.4, 123.9, 122.8, 122.4, 108.3, 40.9, 39.5, 38.6, 26.7, 14.2. HRMS (UPLC-TOF) *m/z*: $[M+H]^+$ Calcd for $C_{25}H_{20}NO_3$: 382.1443; Found: 382.1445, RT=3.55.

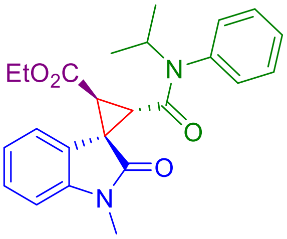

24

Ethyl-3-(isopropyl(phenyl)carbamoyl)-1'-methyl-2'-oxospiro[cyclopropane-1,3'-indoline]-2-carboxylate 24 (major diastereoisomer): obtained using *N*-methyilisatin **1a**, triethyl phosphonoacetate **2a**, and 2-chloro-*N*-isopropyl-*N*-phenylacetamide (propachlor) **23**. Purified by normal phase cromatography (EtOAc:Hexane, 1:4 v/v) to yield **24** as major isomer (27 mg, 67%, 75:25 *dr*), as white solid. ^{1}H NMR (500 MHz, $CDCl_3$) δ 7.36 (td, *J* = 7.7, 1.2 Hz, 2H), 7.29 – 7.26 (m, 1H), 7.20 (dd, *J* = 7.6, 1.2 Hz, 2H), 7.06 (td, *J* = 7.6, 1.0 Hz, 1H), 6.97 (sb, 1H), 6.89 (d, *J* = 7.8 Hz 1H), 5.73 (d, *J* = 6.4 Hz, 1H), 4.89 (hept, *J* = 6.7 Hz, 1H), 4.12 (qd, *J* = 7.1, 2.3 Hz, 2H), 3.39 (d, *J* = 7.7 Hz, 1H), 3.08 (s, 3H), 2.76 (d, *J* = 7.7, Hz, 1H), 1.19 (t, *J* = 7.1 Hz, 3H), 0.99 (d, *J* = 6.9 Hz, 3H), 0.84 (d, *J* = 6.7 Hz, 3H). ^{13}C NMR (126 MHz, $CDCl_3$) δ 171.1, 166.3, 164.2, 144.2, 136.9, 130.3, 129.7, 129.3, 128.5, 128.3, 128.1, 125.1, 122.2, 121.3, 107.8, 61.3, 46.8, 38.3, 36.7, 34.4, 26.4, 21.0, 20.6, 14.0. HRMS (UPLC-TOF) *m/z*: $[M+H]^+$ Calcd for $C_{24}H_{27}N_2O_4$: 407.1971; Found: 407.1978, RT=3.40.

5. Computational studies

Full geometry optimizations and transition structure (TS) searches were carried out with Gaussian 16[8] using the ωB97X-D hybrid functional,[9] 6-31G(d,p) basis set for C, N, O, and H, and LANL2DZ[10] effective core potential for Sc and Br atoms with ultrafine integration grids. Bulk solvent effects in pyridine were considered implicitly through the IEF-PCM polarizable continuum model.[11] The possibility of different conformations was considered for all structures. All stationary points were characterized by a frequency analysis performed at the same level used in the geometry optimizations from which thermal corrections were obtained at 343.15 K. The quasiharmonic approximation reported by Truhlar *et al.*[12] and Head-Gordon *et al.*[13] were used to replace the harmonic oscillator approximation for the calculation of the vibrational contribution to entropy and enthalpy, respectively. Scaled frequencies were not considered. Mass-weighted intrinsic reaction coordinate (IRC) calculations were carried out by using the Hratchian and Schlegel scheme[14] to ensure that the TSs indeed connected the appropriate reactants and products. Gibbs free energies (ΔG) were used for the discussion on the relative stabilities of the considered structures. The lowest energy conformer for each calculated stationary point (**Figure S1 and S2**) was considered in the discussion; all the computed structures can be obtained from authors upon request. Electronic energies, entropies, enthalpies, Gibbs free energies, and lowest frequencies of the calculated structures are summarized in **Table S1 and S3**. Cartesian coordinates of the lowest energy structures calculated with PCM(pyridine)/ ωB97X-D/6-31G(d,p)+LANL2DZ(Sc,Br) are shown in **Table S2 and S4**.

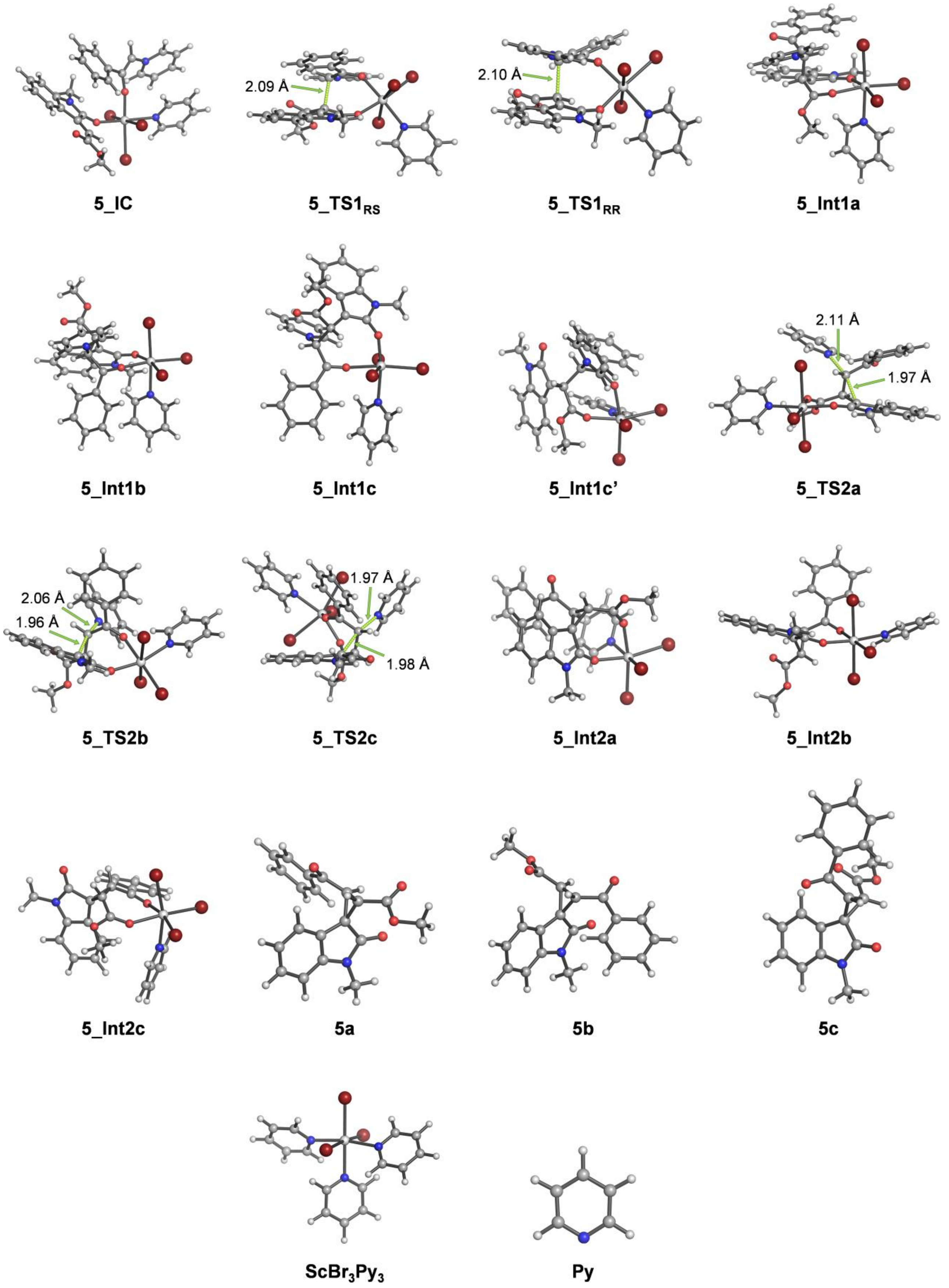

Figure S1. Lowest energy geometries for the reactants, transition states, intermediates and products for the reaction pathways calculated with PCM(pyridine)/ωB97X-D/6-31G(d,p)+LANL2DZ(Sc,Br).

Table S1. Energies, entropies, and lowest frequencies of the lowest energy calculated structures shown in **Figure S1**.[a]

Structure	E_{elec} (Hartree)	E_{elec} + ZPE (Hartree)	H (Hartree)	S (cal mol^{-1} K^{-1})	G (Hartree)	Lowest freq (cm^{-1})	# imag. freq
**5_IC**	–1710.481414	0.523066	–1709.915627	278.8	–1710.054398	11.7	0
**5_TS1$_{RS}$**	–1710.446428	0.523312	–1709.881123	273.9	–1710.016854	–522.1	1
**5_TS1$_{RR}$**	–1710.456122	0.523733	–1709.890398	268.9	–1710.025620	–478.0	1
**5_Int1a**	–1710.493585	0.526045	–1709.925608	271.9	–1710.061497	14.5	0
**5_Int1b**	–1710.487575	0.526188	–1709.919452	272.5	–1710.055204	14.9	0
**5_Int1c**	–1710.485007	0.525749	–1709.917496	275.1	–1710.053392	8.8	0
**5_Int1c'**	–1710.440462	0.526314	–1709.872002	265.5	–1710.006671	24.8	0
**5_TS2a**	–1710.442804	0.522058	–1709.878656	277.5	–1710.015566	–522.0	1
**5_TS2b**	–1710.441536	0.521387	–1709.878339	282.6	–1710.016284	–554.2	1
**5_TS2c**	–1710.411052	0.522524	–1709.846268	269.9	–1709.982475	–451.7	1
**5_Int2a**	–1462.260401	0.431409	–1461.792033	248.3	–1461.915361	13.8	0
**5_Int2b**	–1462.256426	0.431223	–1461.788476	251.8	–1461.912294	11.1	0
**5_Int2c**	–1462.250759	0.431232	–1461.782668	246.2	–1461.905864	19.7	0
**5a**	–1127.708759	0.333948	–1127.350081	172.0	–1127.437990	14.7	0
**5b**	–1127.710687	0.334278	–1127.351582	170.2	–1127.439120	22.0	0
**5c**	–1127.706756	0.334286	–1127.347652	170.0	–1127.435187	18.8	0
**Py**	–248.207338	0.089931	–248.110851	71.2	–248.149811	391.4	0
**$ScBr_3Py_3$**	–830.986690	0.279524	–830.682479	185.7	–830.777670	31.6	0

[a]Energy values calculated at the PCM(pyridine)/ωB97X-D/6-31G(d,p) level. 1 Hartree = 627.51 kcal mol^{-1}. Thermal corrections at 343.15 K.

Table S2. Cartesian coordinates of the lowest energy structures calculated with PCM(pyridine)/M06-2X/6-31G(d,p)+LANL2DZ(Sc,Br).

Structure **5_IC**

```
N -2.334300 1.182400 -1.688300
N 3.057100 2.747400 0.951900
N 3.101900 -1.077100 0.341400
C -4.314200 0.552500 -0.684300
C -3.673300 1.554100 -1.430300
C -4.295000 2.726400 -1.807400
C -5.623200 2.896900 -1.407000
C -6.281900 1.919700 -0.664100
C -5.636000 0.737700 -0.295900
C -3.318700 -0.501100 -0.462900
C -2.074100 -0.002400 -1.130600
C -1.379900 2.014900 -2.393200
C -3.256900 -1.685900 0.163000
C -4.340400 -2.339500 0.932700
C -4.877600 -4.282900 2.129000
C 0.699100 2.108500 0.868300
C 1.667300 3.004300 1.183300
C -0.722600 2.462700 1.137700
C -1.213200 3.742500 0.860600
C -1.580100 1.498800 1.678300
C -2.544400 4.054200 1.125200
C -2.905100 1.819800 1.952800
C -3.391100 3.095900 1.675100
C 3.475200 2.232500 -0.225300
C 3.948500 3.039600 1.930100
C 4.812200 1.964200 -0.442400
C 5.295100 2.815600 1.747500
C 5.739500 2.258700 0.549200
C 4.089400 -1.618000 -0.389300
C 3.395900 -0.608300 1.566700
C 5.395500 -1.707200 0.071100
C 4.672100 -0.666100 2.103000
C 5.693300 -1.225000 1.340400
O -0.974100 -0.594200 -1.164000
O -5.437200 -1.877200 1.174100
O -3.933700 -3.537800 1.352400
O 0.945400 0.926100 0.364400
Sc 0.949800 -0.849200 -0.442900
Br 0.082200 -1.948100 1.891900
Br 1.069300 -3.314700 -1.551200
Br 2.024200 0.187400 -2.759300
H -3.776600 3.490100 -2.375400
H -6.144700 3.808200 -1.680100
H -7.312300 2.077900 -0.364800
H -6.137600 -0.021400 0.288500
H -1.217200 2.937200 -1.829800
H -0.440600 1.470500 -2.488900
H -1.765900 2.251600 -3.386800
H -2.312200 -2.220900 0.133400
H -4.379400 -5.217400 2.377700
H -5.138200 -3.734600 3.036500
H -5.781900 -4.473200 1.547700
H 1.458100 3.934200 1.690500
H -0.559000 4.485900 0.415000
H -1.202000 0.501600 1.885200
H -2.922100 5.043600 0.888500
H -3.564800 1.064400 2.367700
H -4.432700 3.333400 1.864600
H 2.715900 2.035300 -0.970300
H 3.533300 3.441600 2.844700
H 5.104300 1.532000 -1.391000
H 5.981600 3.061800 2.547000
H 6.793400 2.059900 0.394500
H 3.809300 -1.985600 -1.369900
H 2.570200 -0.179200 2.123100
H 6.156900 -2.150300 -0.559000
H 4.856000 -0.275100 3.096200
H 6.704100 -1.282200 1.729500
```

Structure **5_TS1**$_{\mathbf{RS}}$

```
N -1.385600 -2.645600 -1.022100
N -1.355700 2.947000 1.077800
N 3.254700 -0.881800 -1.315900
C -3.270600 -1.358600 -1.113200
C -2.777000 -2.678600 -1.138900
C -3.595100 -3.794600 -1.234100
C -4.968400 -3.576800 -1.302200
C -5.485800 -2.277700 -1.277400
C -4.652200 -1.166000 -1.184200
C -0.976700 -1.361700 -0.918600
C -2.108500 -0.487100 -0.979100
C -1.934200 0.898300 -0.821600
C -1.824300 1.582400 1.150700
C -0.528800 -3.808400 -0.915500
C -1.023100 0.515200 1.652400
C -1.703300 -0.701900 2.123100
C -0.920800 -1.717000 2.694200
C -3.080600 -0.916900 1.948000
C -1.495700 -2.923900 3.061300
C -3.648800 -2.131700 2.305100
C -2.859000 -3.136700 2.857500
C -3.018800 1.837700 -1.258300
C -3.498400 3.636300 -2.685500
C -2.307100 3.902200 0.876700
C -0.056600 3.310500 1.196100
C -1.976000 5.231000 0.770800
C 0.316200 4.638800 1.104700
C -0.640200 5.616700 0.880900
C 4.566000 -0.613200 -1.200600
C 2.839400 -1.606900 -2.368100
C 5.500800 -1.055000 -2.124400
C 3.710300 -2.086500 -3.333600
C 5.067200 -1.806100 -3.211300
O 0.265200 -1.057000 -0.791400
O 0.243500 0.518000 1.551300
O -4.151600 1.860900 -0.820300
O -2.556000 2.682800 -2.171000
Sc 1.731200 -0.157000 0.216600
Br 3.422600 1.294400 1.739600
Br 2.413100 -2.438100 1.472400
Br 1.555500 1.961100 -1.509900
```

H -3.182100 -4.797600 -1.248300
H -5.641200 -4.424900 -1.374400
H -6.559900 -2.130700 -1.329200
H -5.070100 -0.168100 -1.149300
H -0.544800 -4.381100 -1.846000
H 0.485700 -3.472100 -0.703100
H -0.870900 -4.440300 -0.092100
H 0.141100 -1.556800 2.831200
H -3.716500 -0.171700 1.485700
H -0.878600 -3.702100 3.496900
H -4.705300 -2.299000 2.127900
H -3.305900 -4.087700 3.128800
H -0.934000 1.263800 -1.049000
H -4.339600 3.121500 -3.151800
H -3.863300 4.278000 -1.881100
H -2.948600 4.220100 -3.419700
H -2.862200 1.577400 1.449000
H -3.328500 3.552100 0.808300
H 0.656200 2.524000 1.382700
H -2.765900 5.953100 0.609800
H 1.366400 4.879500 1.206400
H -0.358800 6.659800 0.800500
H 4.858100 -0.024800 -0.337300
H 1.773600 -1.797600 -2.421800
H 6.547100 -0.810900 -1.986300
H 3.325200 -2.667300 -4.163000
H 5.774400 -2.166700 -3.950400

Structure **5_TS1$_{RR}$**
N -1.280900 -2.679700 -0.896600
N -2.606100 0.607400 1.633900
N 3.133800 -1.630100 -0.915800
C -3.240100 -1.499100 -0.852000
C -2.672800 -2.789600 -0.830600
C -3.426300 -3.950400 -0.738800
C -4.809800 -3.810300 -0.662600
C -5.399000 -2.543500 -0.680400
C -4.629100 -1.386300 -0.775600
C -0.934700 -1.374500 -0.936600
C -2.122600 -0.560900 -0.938800
C -2.008800 0.831800 -1.039700
C -1.963700 1.644400 0.899100
C -0.361200 -3.798500 -0.926000
C -0.556200 1.905500 0.959900
C -0.080800 3.280900 0.708100
C 1.198100 3.620800 1.167700
C -0.850600 4.242100 0.037100
C 1.690200 4.904600 0.976300
C -0.343600 5.517100 -0.172000
C 0.922800 5.853100 0.304200
C -3.138100 1.693000 -1.509000
C -3.673200 3.530600 -2.868400
C -3.939400 0.754900 1.881800
C -1.988000 -0.557200 1.943500
C -4.669900 -0.257800 2.448000
C -2.691900 -1.602500 2.516900
C -4.047700 -1.469100 2.760600
C 4.178700 -2.213300 -0.304300
C 2.873000 -1.963800 -2.192400
C 4.992100 -3.140200 -0.938000
C 3.637000 -2.881900 -2.895200
C 4.717600 -3.482400 -2.257400
O 0.281500 -0.988600 -0.985300
O 0.290400 0.985000 1.153200
O -4.294000 1.610300 -1.148000
O -2.691500 2.615800 -2.358000
Sc 1.731500 -0.181500 0.164600
Br 2.092000 1.598100 -1.793500
Br 3.761400 0.776800 1.642900
Br 1.282900 -2.156100 1.978900
H -2.956900 -4.928100 -0.723900
H -5.432800 -4.695400 -0.587300
H -6.479000 -2.456100 -0.618200
H -5.095700 -0.410500 -0.781400
H -0.571800 -4.427800 -1.794300
H 0.653100 -3.409000 -0.990000
H -0.456900 -4.387500 -0.011500
H 1.800400 2.873400 1.672100
H -1.825900 3.998200 -0.366900
H 2.677800 5.161100 1.343900
H -0.936800 6.248700 -0.709700
H 1.312200 6.853500 0.145900
H -1.037000 1.183600 -1.378600
H -3.126500 4.209400 -3.518800
H -4.436000 2.989400 -3.430200
H -4.142800 4.077300 -2.048500
H -2.587300 2.527100 0.890200
H -4.379500 1.697100 1.588000
H -0.924800 -0.610700 1.774200
H -5.725800 -0.101700 2.625400
H -2.150300 -2.510700 2.749400
H -4.616800 -2.283900 3.191700
H 4.356700 -1.916100 0.722700
H 2.018800 -1.476200 -2.647400
H 5.822000 -3.581400 -0.399600
H 3.384200 -3.116000 -3.922100
H 5.335100 -4.204800 -2.780200

Structure **5_Int1a**
N -1.522900 2.637600 -0.071300
N -1.530600 -3.141300 -0.296200
N 2.667200 1.351400 -1.339000
C -2.802700 1.339800 -1.412900
C -2.715900 2.593400 -0.764900
C -3.728500 3.545100 -0.842500
C -4.859300 3.219600 -1.582200
C -4.968300 1.978700 -2.229200
C -3.950400 1.036500 -2.153000
C -0.876000 1.435300 -0.227800
C -1.613500 0.610400 -1.067800
C -1.403600 -0.855500 -1.302400
C -1.761100 -1.699100 -0.046200
C -1.075600 3.721900 0.773300
C -3.233000 -1.445600 0.358600
C -3.541400 -0.295800 1.239700
C -4.829800 0.248200 1.145800

C -2.605100 0.281600 2.104400
C -5.167200 1.372900 1.882800
C -2.954600 1.398900 2.854900
C -4.227100 1.951900 2.735600
C 0.027600 -1.128800 -1.711100
C 1.511900 -1.663100 -3.451600
C -2.186600 -3.778400 -1.293600
C -0.644100 -3.799700 0.485400
C -1.965700 -5.119100 -1.529600
C -0.392900 -5.140100 0.284800
C -1.060300 -5.813800 -0.733600
C 3.971400 1.607500 -1.536000
C 1.791900 1.778000 -2.266000
C 4.442400 2.279300 -2.654100
C 2.180200 2.449600 -3.414600
C 3.532800 2.705600 -3.615000
O 0.258500 1.222800 0.379700
O -4.101900 -2.149200 -0.122200
O 0.985300 -1.071900 -0.937100
O 0.166900 -1.431200 -2.971300
Sc 1.924600 0.238500 0.545800
Br 0.943900 -1.236000 2.624600
Br 3.127300 2.084700 2.006700
Br 4.046100 -1.414800 0.281800
H -3.644400 4.499400 -0.332600
H -5.671200 3.935800 -1.658200
H -5.865000 1.751700 -2.797300
H -4.055100 0.076500 -2.651200
H -1.784100 3.877500 1.592600
H -0.102600 3.457900 1.187200
H -0.983400 4.645000 0.195000
H -5.541000 -0.201200 0.462400
H -1.605000 -0.125500 2.214100
H -6.156700 1.806300 1.786600
H -2.225800 1.840900 3.525800
H -4.488400 2.835200 3.309700
H -2.039500 -1.160800 -2.137300
H 2.096300 -0.747100 -3.362200
H 1.976000 -2.460900 -2.872100
H 1.394100 -1.948500 -4.493200
H -1.079800 -1.420100 0.758300
H -2.884900 -3.194900 -1.872900
H -0.148300 -3.213100 1.252800
H -2.501600 -5.604000 -2.334800
H 0.324400 -5.638700 0.923300
H -0.873800 -6.866900 -0.908200
H 4.648600 1.252200 -0.768300
H 0.742500 1.572400 -2.083100
H 5.505200 2.458400 -2.762300
H 1.430800 2.764500 -4.130400
H 3.870400 3.229600 -4.502500

Structure **5_Int1b**
N 0.707000 2.417400 -1.963200
N 2.273900 -2.002200 1.548500
N -2.499400 1.434300 0.362300
C 2.747300 1.783900 -1.205100
C 1.993000 2.863300 -1.725400
C 2.537600 4.130200 -1.916800
C 3.869000 4.318500 -1.562200
C 4.632900 3.265800 -1.035700
C 4.087900 2.000100 -0.856200
C 0.626900 1.086500 -1.601400
C 1.864500 0.651000 -1.148600
C 2.165400 -0.733200 -0.649700
C 2.132700 -0.682700 0.879000
C -0.368400 3.200200 -2.532600
C 0.804200 -0.045400 1.312200
C 0.759400 1.038100 2.291000
C -0.396900 1.159300 3.078500
C 1.796900 1.975400 2.404200
C -0.502500 2.199800 3.987700
C 1.664900 3.033700 3.293100
C 0.524100 3.139300 4.088400
C 3.516000 -1.244200 -1.124700
C 4.779300 -1.863400 -2.994800
C 3.269600 -2.173700 2.445800
C 1.420400 -3.006600 1.249600
C 3.431100 -3.380600 3.089900
C 1.546900 -4.233300 1.867500
C 2.559600 -4.427700 2.800900
C -1.638700 2.456500 0.486900
C -3.752000 1.608400 0.818600
C -1.981900 3.670800 1.060700
C -4.177700 2.788900 1.407500
C -3.277300 3.842100 1.532900
O -0.515600 0.458700 -1.649400
O -0.232900 -0.526400 0.848600
O 4.461500 -1.442800 -0.387900
O 3.531700 -1.429800 -2.434700
Sc -1.832100 -0.562200 -0.617500
Br -0.925700 -2.917300 -1.628800
Br -3.843100 -0.276200 -2.287700
Br -3.150500 -1.815300 1.383000
H 1.943900 4.942400 -2.323400
H 4.322500 5.295400 -1.694500
H 5.669700 3.442800 -0.767400
H 4.695000 1.193400 -0.455900
H -1.261600 2.577000 -2.576800
H -0.575600 4.076900 -1.912000
H -0.109800 3.528300 -3.542900
H -1.193300 0.429900 2.967000
H 2.674300 1.919900 1.767400
H -1.387500 2.289100 4.607500
H 2.451000 3.776600 3.366400
H 0.433500 3.962100 4.789900
H 1.393600 -1.411100 -1.032100
H 4.601900 -1.958200 -4.063600
H 5.556000 -1.121900 -2.799100
H 5.072900 -2.823800 -2.567800
H 2.972500 -0.085700 1.227100
H 3.919000 -1.326500 2.617800
H 0.651100 -2.807800 0.510700
H 4.235400 -3.491700 3.805100
H 0.850700 -5.020200 1.609000
H 2.672100 -5.385300 3.295500

H -0.632800 2.294900 0.121800
H -4.422900 0.765100 0.704900
H -1.237400 4.453800 1.139500
H -5.198900 2.873200 1.758600
H -3.580900 4.777200 1.990800

Structure **5_Int1c**
N -2.125800 -2.681100 0.077600
N -2.217800 1.829700 0.873400
N 3.423300 -0.108900 0.650900
C -3.530600 -0.919000 -0.162000
C -3.410400 -2.242600 0.328900
C -4.461900 -2.907200 0.954100
C -5.668200 -2.228000 1.085100
C -5.815600 -0.922200 0.594600
C -4.762600 -0.264500 -0.029800
C -1.428600 -1.667900 -0.538600
C -2.246500 -0.562000 -0.707400
C -1.833300 0.721600 -1.352700
C -1.429900 1.873000 -0.377600
C -1.587900 -3.972200 0.444200
C 0.074000 1.862300 -0.072000
C 0.814200 3.121300 0.009400
C 2.197900 3.091900 -0.227300
C 0.183300 4.333900 0.328300
C 2.938800 4.260500 -0.140300
C 0.935400 5.494600 0.435600
C 2.309600 5.458000 0.198600
C -2.934000 1.243100 -2.274600
C -4.241700 0.753600 -4.152400
C -3.289700 2.648800 0.993500
C -1.936100 0.903200 1.815600
C -4.111100 2.566100 2.094200
C -2.739900 0.780100 2.931700
C -3.841700 1.613200 3.075600
C 4.611300 -0.200100 0.028800
C 3.327400 0.706600 1.714400
C 5.727700 0.516000 0.434000
C 4.393500 1.458500 2.183500
C 5.617500 1.364900 1.530200
O -0.174100 -1.828400 -0.868600
O 0.644800 0.780900 0.053900
O -3.524400 2.295100 -2.118700
O -3.176900 0.395600 -3.260400
Sc 1.557500 -1.185800 -0.269800
Br 0.769700 -1.599600 2.311400
Br 2.923800 -3.412200 -0.550800
Br 2.201800 -0.066500 -2.621600
H -4.343100 -3.919300 1.328000
H -6.506700 -2.717400 1.570200
H -6.770400 -0.416700 0.702000
H -4.901400 0.743800 -0.408400
H -1.579900 -4.090900 1.531000
H -2.183900 -4.771400 -0.005100
H -0.564500 -4.036300 0.076400
H 2.666700 2.157600 -0.513400
H -0.882200 4.376700 0.526800
H 4.004900 4.238800 -0.336600
H 0.452100 6.428100 0.700400
H 2.891700 6.370600 0.273100
H -0.960900 0.518900 -1.985900
H -4.292500 -0.050600 -4.882800
H -5.181000 0.830700 -3.601500
H -4.023900 1.704200 -4.642100
H -1.673600 2.819400 -0.855900
H -3.469600 3.324900 0.168500
H -1.066300 0.274900 1.663500
H -4.960400 3.232300 2.166300
H -2.491600 0.027300 3.668400
H -4.486200 1.525500 3.942300
H 4.655000 -0.868000 -0.824200
H 2.356300 0.750500 2.193900
H 6.660300 0.406400 -0.105900
H 4.257600 2.105200 3.041800
H 6.470900 1.941900 1.869600

Structure **5_Int1c’**
N 4.722100 0.248600 -0.631300
N 0.423300 3.408300 0.597100
N -0.501000 -2.169600 0.537600
C 2.865100 -0.393900 -1.780500
C 4.206400 -0.740500 -1.442300
C 4.818100 -1.903700 -1.890400
C 4.087100 -2.757800 -2.717900
C 2.780100 -2.430900 -3.087000
C 2.168500 -1.263400 -2.633200
C 3.758600 1.235500 -0.413500
C 2.580400 0.829400 -1.083200
C 1.362800 1.713700 -1.020600
C 1.075000 2.080100 0.480100
C 6.031000 0.255000 -0.030600
C 0.374200 1.025400 1.334200
C 1.103400 0.273000 2.341800
C 0.368400 -0.637900 3.129800
C 2.502200 0.345900 2.480300
C 1.022100 -1.482300 4.006800
C 3.147400 -0.516300 3.356200
C 2.414900 -1.432000 4.108100
C 0.216800 1.110600 -1.799500
C -0.934000 1.051600 -3.849800
C 1.238000 4.451100 0.888300
C -0.889100 3.599700 0.349100
C 0.735500 5.731500 0.968000
C -1.435900 4.864500 0.420400
C -0.621300 5.946100 0.737800
C 0.802500 -2.039100 0.243100
C -0.886200 -3.277800 1.200400
C 1.761300 -2.976500 0.592300
C 0.008300 -4.256500 1.598100
C 1.359100 -4.105400 1.293000
O 3.973000 2.249600 0.314600
O -0.829400 0.791500 1.121700
O -0.480400 0.163400 -1.410900
O 0.071200 1.629000 -2.979500
Sc -2.000800 -0.558100 -0.099200
Br -3.511900 1.480000 -0.939600

Br -3.488600 -1.065900 1.987200
Br -2.881100 -2.283200 -1.819100
H 5.839000 -2.139300 -1.604600
H 4.541200 -3.674500 -3.080500
H 2.223000 -3.098300 -3.737700
H 1.145200 -1.062900 -2.932900
H 6.817900 0.249700 -0.791800
H 6.110600 1.166800 0.562500
H 6.168700 -0.613500 0.623000
H -0.710800 -0.679000 3.027800
H 3.099400 1.053400 1.913300
H 0.455300 -2.187100 4.604400
H 4.226600 -0.473500 3.450500
H 2.929300 -2.106100 4.785300
H 1.570100 2.683200 -1.492800
H -0.872100 1.628100 -4.768200
H -0.699400 0.001800 -4.029200
H -1.914300 1.146300 -3.382300
H 2.081100 2.249700 0.862000
H 2.285900 4.211600 1.025500
H -1.485500 2.731300 0.101800
H 1.407600 6.545700 1.204900
H -2.492800 4.983900 0.220500
H -1.035200 6.945800 0.796400
H 1.097900 -1.148400 -0.289400
H -1.943600 -3.364400 1.419000
H 2.792600 -2.804600 0.304900
H -0.355300 -5.123100 2.136700
H 2.079300 -4.858800 1.592800

Structure **5_TS2a**
N -2.214200 -2.324800 0.409100
N 3.753400 0.170100 -0.094600
C -3.115300 -0.674100 1.685800
C -3.346900 -1.942700 1.125700
C -4.540800 -2.628200 1.295400
C -5.527300 -2.007600 2.058400
C -5.314900 -0.747700 2.628500
C -4.112000 -0.069500 2.448700
C -1.275400 -1.348500 0.479700
C -1.797700 -0.258500 1.239800
C -1.203100 1.111900 1.443300
C -1.643600 1.317500 0.060800
C -2.083700 -3.544500 -0.361500
C -3.113300 1.505700 -0.211600
C -3.743000 0.641100 -1.240300
C -5.140500 0.556300 -1.231600
C -3.005900 -0.156600 -2.125700
C -5.793300 -0.321000 -2.085700
C -3.664700 -1.027600 -2.986100
C -5.055100 -1.116300 -2.961800
C 0.280500 1.243300 1.639200
C 2.028500 2.022500 2.991800
C 3.819300 1.498000 -0.294100
C 4.884200 -0.484600 0.220700
C 5.000600 2.212900 -0.181900
C 6.107200 0.156100 0.348700
C 6.167900 1.530200 0.146200
O -0.148200 -1.474600 -0.112100
O -3.754500 2.270700 0.489000
O 1.106600 0.876100 0.801200
O 0.612700 1.837300 2.743600
Sc 1.758300 -0.944300 -0.202700
Br 1.263900 0.255900 -2.567600
Br 2.178300 -1.820200 2.304200
Br 2.768800 -3.093900 -1.312600
H -4.702800 -3.603500 0.849700
H -6.475400 -2.511900 2.212100
H -6.101200 -0.289800 3.219300
H -3.963700 0.913400 2.882600
H -2.807600 -3.543600 -1.181000
H -1.074400 -3.587800 -0.768100
H -2.254400 -4.411900 0.279500
H -5.695200 1.166300 -0.527500
H -1.921800 -0.113200 -2.163300
H -6.875400 -0.393100 -2.064600
H -3.089600 -1.641200 -3.671200
H -5.564000 -1.807200 -3.626100
H -1.755000 1.679200 2.190600
H 2.525800 1.052100 2.981800
H 2.445700 2.674900 2.224000
H 2.083100 2.488000 3.971700
H -0.983700 0.943100 -0.714200
H 2.889400 1.992300 -0.550900
H 4.792400 -1.554200 0.373700
H 4.999400 3.282600 -0.352100
H 6.989100 -0.418300 0.604300
H 7.109100 2.060800 0.241000
N -1.009600 3.286800 -0.348100
C -1.477700 4.297200 0.393200
C 0.041200 3.485000 -1.150900
C -0.905300 5.561100 0.355900
C 0.669300 4.719900 -1.246200
C 0.188600 5.775400 -0.477600
H -2.336000 4.070200 1.016800
H 0.385800 2.626400 -1.721000
H -1.312100 6.355500 0.970000
H 1.516800 4.842100 -1.910100
H 0.659000 6.751400 -0.527300

Structure **5_TS2b**
N -1.630900 -2.964100 -0.445800
N 3.265300 0.736500 0.427000
C -3.387300 -1.502800 -0.399300
C -2.996400 -2.837000 -0.174300
C -3.883000 -3.812700 0.257100
C -5.204800 -3.426500 0.467700
C -5.613900 -2.106300 0.251800
C -4.715900 -1.133600 -0.179300
C -1.132000 -1.766600 -0.826900
C -2.183600 -0.788700 -0.789700
C -1.969100 0.625800 -1.259300
C -1.957500 0.896800 0.177100
C -0.867500 -4.192200 -0.338500
C -0.795900 0.503000 1.001900
C -0.979900 0.219200 2.425600

C 0.123900 0.359200 3.279100
C -2.211600 -0.220200 2.934700
C -0.011200 0.088700 4.632400
C -2.332000 -0.510600 4.286500
C -1.237100 -0.347700 5.134500
C -3.108200 1.253900 -2.027100
C -3.973700 1.633400 -4.168400
C 4.515400 0.312700 0.679400
C 2.921800 1.976900 0.814600
C 5.456800 1.104100 1.320100
C 3.799800 2.826700 1.467000
C 5.093400 2.383700 1.724400
O 0.085200 -1.594200 -1.163300
O 0.338300 0.485400 0.487600
O -4.061400 1.794500 -1.509200
O -2.928700 1.115100 -3.331400
Sc 1.767100 -0.640900 -0.610900
Br 1.593300 1.284200 -2.526600
Br 3.485400 -2.049100 -2.015300
Br 2.019200 -2.235600 1.528800
H -3.561400 -4.834500 0.426000
H -5.925800 -4.163400 0.805000
H -6.650200 -1.834200 0.422300
H -5.042600 -0.112900 -0.345700
H 0.167200 -3.975800 -0.599300
H -0.906800 -4.565600 0.686900
H -1.273100 -4.943400 -1.020200
H 1.071700 0.689600 2.869700
H -3.061800 -0.379300 2.279000
H 0.837800 0.209800 5.295800
H -3.277700 -0.867200 4.679000
H -1.338700 -0.567100 6.192300
H -1.012100 0.733500 -1.772500
H -4.088400 2.706300 -4.006100
H -3.656600 1.431900 -5.188900
H -4.915300 1.126500 -3.950700
H -2.929200 0.960300 0.650400
H 4.755800 -0.693200 0.353100
H 1.907800 2.286400 0.590300
H 6.452100 0.715500 1.496900
H 3.470400 3.815800 1.761200
H 5.806100 3.025700 2.230600
N -1.661800 2.912100 0.459500
C -0.685600 3.506500 -0.233000
C -2.446700 3.626800 1.268700
C -0.452700 4.871000 -0.133600
C -2.277700 4.995500 1.423500
C -1.261900 5.626200 0.710600
H -0.082900 2.869000 -0.877700
H -3.223700 3.085600 1.799800
H 0.346000 5.324400 -0.707900
H -2.929800 5.548900 2.088100
H -1.104200 6.694400 0.810800

Structure **5_TS2c**

N 4.777700 -0.410400 -0.853800
N -1.974100 -1.600000 1.607000
C 2.686400 -1.315800 -1.106400
C 4.015000 -1.568400 -0.698900
C 4.418200 -2.807800 -0.236400
C 3.458300 -3.825400 -0.184400
C 2.147900 -3.598900 -0.598200
C 1.752900 -2.342600 -1.066600
C 4.006500 0.613100 -1.353800
C 2.627200 0.090400 -1.447700
C 1.561300 0.955900 -2.063300
C 1.540100 1.577400 -0.718700
C 6.174200 -0.277500 -0.510600
C 1.255100 0.676700 0.402000
C 1.986500 0.790200 1.675600
C 1.408400 0.221400 2.818400
C 3.239200 1.416800 1.776300
C 2.066600 0.278200 4.039300
C 3.895500 1.464200 2.998400
C 3.312000 0.896000 4.130400
C 0.340500 0.278100 -2.622100
C -0.527200 -0.913200 -4.457300
C -3.158900 -1.653100 2.235000
C -0.978500 -2.395200 2.032100
C -3.391300 -2.497000 3.309900
C -1.125900 -3.253700 3.109400
C -2.354800 -3.307800 3.759900
O 4.398300 1.755600 -1.596500
O 0.305400 -0.161500 0.325900
O -0.779500 0.286200 -2.103100
O 0.579700 -0.276200 -3.771500
Sc -1.691300 -0.263500 -0.191100
Br -2.046200 1.656500 1.654900
Br -1.862700 -2.727800 -1.351500
Br -4.029600 0.386900 -1.218100
H 5.441500 -2.987400 0.075400
H 3.747700 -4.807400 0.175400
H 1.419500 -4.402600 -0.563700
H 0.731400 -2.196700 -1.393900
H 6.475300 0.744100 -0.741700
H 6.325600 -0.468000 0.555900
H 6.784300 -0.975000 -1.091100
H 0.432100 -0.240600 2.740400
H 3.723000 1.850800 0.907600
H 1.606900 -0.157200 4.919900
H 4.867200 1.941000 3.066900
H 3.828800 0.937300 5.083600
H 1.969400 1.621800 -2.826900
H -0.989000 -1.650400 -3.800400
H -1.253100 -0.151800 -4.744200
H -0.082500 -1.377600 -5.332900
H 2.370400 2.259100 -0.590800
H -3.934900 -0.995300 1.857200
H -0.044100 -2.330300 1.483800
H -4.365900 -2.509000 3.782100
H -0.291800 -3.869700 3.422500
H -2.503000 -3.974000 4.602800
N 0.303100 3.106100 -0.572400
C 0.546700 3.912600 0.464800
C -0.708500 3.375700 -1.402800
C -0.220200 5.039500 0.713500

C -1.522500 4.484400 -1.222700
C -1.274800 5.329600 -0.146400
H 1.375700 3.637800 1.110300
H -0.874600 2.684900 -2.219600
H 0.007000 5.669100 1.564900
H -2.335600 4.670300 -1.913500
H -1.896700 6.201700 0.021900

Structure **5_Int2a**
N 1.132900 2.235700 -1.567600
N -0.927500 -2.124100 -0.167500
C 2.678600 2.232400 0.129600
C 2.329300 2.819600 -1.088100
C 3.080800 3.818100 -1.678900
C 4.228200 4.231300 -0.997600
C 4.587400 3.663300 0.223400
C 3.811000 2.657800 0.804900
C 0.687500 1.304000 -0.724900
C 1.632600 1.222200 0.438300
C 1.854500 -0.141500 1.068200
C 1.207600 0.925300 1.878800
C 0.485400 2.587800 -2.819400
C -0.216100 0.810700 2.271500
C -1.869900 1.432200 3.815400
C 3.258400 -0.576700 1.418700
C 3.929800 -1.512600 0.485100
C 5.096300 -2.158700 0.911500
C 3.441800 -1.741500 -0.805800
C 5.755400 -3.036400 0.062200
C 4.111800 -2.610500 -1.659600
C 5.263200 -3.262100 -1.223900
C -0.257400 -2.529400 -1.261000
C -0.757800 -2.811600 0.975800
C 0.605400 -3.614200 -1.248100
C 0.094600 -3.900800 1.073200
C 0.793700 -4.308500 -0.057800
O -0.309700 0.583500 -0.944500
O -1.055200 0.132200 1.671400
O -0.498400 1.477600 3.349700
O 3.794900 -0.158800 2.429100
Sc -2.103200 -0.144800 -0.177100
Br -2.920200 2.366200 0.058100
Br -4.035900 -1.304500 1.155600
Br -2.975900 -0.603800 -2.596400
H 2.796300 4.264000 -2.625000
H 4.845600 5.011800 -1.428500
H 5.481100 4.006400 0.732300
H 4.094000 2.219800 1.755400
H 1.171200 2.407200 -3.649400
H -0.403000 1.968000 -2.931300
H 0.198300 3.640600 -2.797900
H 1.195900 -0.911100 0.676500
H 1.834200 1.414200 2.617100
H -2.525800 1.833700 3.042400
H -2.141600 0.401900 4.045500
H -1.882500 2.050900 4.708100
H 5.467000 -1.966400 1.912400
H 2.548600 -1.237400 -1.159400
H 6.653000 -3.544400 0.397900
H 3.735900 -2.778400 -2.663100
H 5.780800 -3.946100 -1.888600
H -0.424700 -1.955900 -2.165600
H -1.321600 -2.462500 1.833400
H 1.124600 -3.897100 -2.155400
H 0.203100 -4.413300 2.021100
H 1.473300 -5.152200 -0.013300

Structure **5_Int2b**
N -2.415100 -1.279400 1.937100
N 3.332600 0.192600 -0.554100
C -3.811800 -0.056400 0.585800
C -3.720700 -0.756100 1.791200
C -4.779500 -0.873700 2.672100
C -5.974300 -0.247400 2.310700
C -6.081300 0.466500 1.118600
C -4.997600 0.575000 0.243700
C -1.651400 -0.945300 0.898100
C -2.472300 -0.126800 -0.056900
C -2.135800 -0.262200 -1.530800
C -1.911700 1.053500 -0.865800
C -1.971800 -2.087800 3.059700
C -3.246800 -0.363600 -2.531300
C -4.210600 -1.748200 -4.154600
C -0.541900 1.581300 -0.644200
C -0.335500 2.998500 -0.350600
C 0.982700 3.484900 -0.335500
C -1.399300 3.862700 -0.044300
C 1.229900 4.814700 -0.037200
C -1.143100 5.190500 0.267300
C 0.167100 5.666900 0.264700
C 4.454500 0.189400 0.186800
C 3.342300 0.848500 -1.728600
C 5.613400 0.835800 -0.213700
C 4.457300 1.525400 -2.195300
C 5.615700 1.519300 -1.424500
O -0.471200 -1.333700 0.751000
O -4.083100 0.492900 -2.720900
O -3.186800 -1.520700 -3.173900
O 0.432200 0.813600 -0.717400
Sc 1.442800 -0.817400 0.192500
Br 1.232500 -2.061000 -2.164200
Br 2.663700 -2.816000 1.359300
Br 1.564400 0.760600 2.346400
H -4.689600 -1.422500 3.602600
H -6.829100 -0.316600 2.974500
H -7.019300 0.947400 0.864100
H -5.085400 1.126300 -0.684700
H -2.573600 -2.996800 3.116100
H -0.925900 -2.346600 2.904300
H -2.075700 -1.515900 3.983600
H -4.165000 -0.982400 -4.930600
H -4.000500 -2.730000 -4.572200
H -5.193900 -1.733200 -3.681600
H -1.259900 -0.874500 -1.734000
H 1.798400 2.808900 -0.562500
H -2.422900 3.507500 -0.019300

H 2.247500 5.188900 -0.033500
H -1.964100 5.854200 0.513200
H 0.361400 6.707500 0.502800
H -2.681500 1.786700 -1.075700
H 4.408200 -0.350300 1.126000
H 2.421800 0.821900 -2.300800
H 6.491700 0.801800 0.419000
H 4.412400 2.041900 -3.146200
H 6.505900 2.037900 -1.763200

Structure **5_Int2c**
N 4.771100 -1.197300 -0.844100
N -1.503200 -0.011500 1.946900
C 2.865800 -0.849300 0.400900
C 4.248100 -1.074200 0.452500
C 4.930300 -1.149800 1.655300
C 4.187300 -0.994100 2.828800
C 2.813400 -0.770800 2.792200
C 2.139300 -0.695400 1.570400
C 3.789800 -1.071600 -1.788700
C 2.499600 -0.823400 -1.036900
C 1.210500 -1.348500 -1.637100
C 1.479900 0.117300 -1.666700
C 6.162600 -1.448400 -1.151700
C 0.861100 1.181100 -0.822100
C 1.628700 2.395900 -0.544200
C 0.962300 3.486000 0.039200
C 3.000400 2.484700 -0.825800
C 1.661600 4.643800 0.341100
C 3.695900 3.643800 -0.513600
C 3.028400 4.719900 0.069700
C 0.282700 -2.087000 -0.735900
C -0.119200 -4.138100 0.331500
C -1.011200 1.096100 2.529300
C -1.498800 -1.157200 2.651300
C -0.491900 1.097900 3.814500
C -0.998000 -1.239600 3.941100
C -0.483300 -0.091700 4.535200
O 3.915600 -1.130700 -2.998600
O -0.283700 1.069300 -0.364100
O -0.742600 -1.649000 -0.212500
O 0.685100 -3.308700 -0.536700
Sc -2.121800 0.001800 -0.275800
Br -4.045400 -1.703100 0.172900
Br -3.358100 2.276700 0.048100
Br -2.097400 -0.119100 -2.891300
H 5.999900 -1.323700 1.689500
H 4.695600 -1.048900 3.785600
H 2.258600 -0.652200 3.716000
H 1.068900 -0.518000 1.551500
H 6.484800 -2.396300 -0.712800
H 6.259200 -1.499400 -2.235600
H 6.789800 -0.639400 -0.768500
H 1.782100 0.485300 -2.645800
H -0.104200 3.417700 0.226300
H 3.530700 1.654500 -1.277300
H 1.145700 5.488400 0.783600
H 4.757400 3.708900 -0.723600
H 3.575600 5.625400 0.310300
H 1.346000 -1.852300 -2.591200
H -0.067200 -3.746100 1.348000
H -1.151500 -4.152300 -0.018100
H 0.329400 -5.125800 0.274800
H -1.037900 2.001600 1.935400
H -1.913200 -2.026400 2.153900
H -0.103400 2.017500 4.234200
H -1.016100 -2.188100 4.463600
H -0.083100 -0.123700 5.542800

Structure **5a**
N 1.332100 1.747000 1.258200
C 0.340900 1.243500 -0.752600
C 0.685600 2.275300 0.131100
C 0.404400 3.602800 -0.144900
C -0.241000 3.886100 -1.352000
C -0.581300 2.872600 -2.243800
C -0.287800 1.537200 -1.950500
C 1.445800 0.385400 1.171300
C 0.792800 -0.022900 -0.126400
C 0.017100 -1.346600 -0.107700
C 1.280800 -1.246200 -0.867100
C 1.830700 2.530000 2.365900
C 2.496300 -1.981200 -0.374700
C 4.830200 -1.888200 -0.212300
C -1.293500 -1.393800 -0.840900
C -2.532200 -1.153700 -0.052800
C -3.769300 -1.373900 -0.668300
C -2.488400 -0.682000 1.263500
C -4.947100 -1.137700 0.026700
C -3.669800 -0.437300 1.955000
C -4.897400 -0.668500 1.339300
O 1.958800 -0.354000 1.996800
O 2.480300 -3.066200 0.158300
O 3.603000 -1.288600 -0.643800
O -1.326500 -1.587800 -2.045100
H 0.675000 4.394700 0.544700
H -0.475100 4.917100 -1.596000
H -1.077000 3.119000 -3.176400
H -0.552200 0.748100 -2.646000
H 1.014000 3.071400 2.850900
H 2.284100 1.843200 3.079900
H 2.582800 3.245400 2.022100
H 0.065100 -1.863400 0.847200
H 1.199400 -1.182100 -1.949400
H 4.822500 -2.020600 0.871200
H 4.974600 -2.855900 -0.696200
H 5.615800 -1.195100 -0.505800
H -3.787000 -1.731600 -1.691900
H -1.538100 -0.489600 1.750100
H -5.904300 -1.315800 -0.451500
H -3.632000 -0.065700 2.973300
H -5.818200 -0.481000 1.882200

Structure **5b**
N -0.006600 1.702400 1.572600
C -1.149200 1.122800 -0.336900

C -0.738700 2.186600 0.479800
C -1.036300 3.504700 0.176000
C -1.763600 3.747500 -0.992300
C -2.164700 2.703600 -1.821600
C -1.853900 1.378300 -1.501900
C 0.109800 0.339700 1.525800
C -0.616700 -0.117800 0.280900
C -1.252000 -1.510000 0.368400
C -0.059500 -1.293200 -0.486200
C 0.576000 2.526500 2.607000
C -2.575800 -1.752000 -0.272300
C -4.784200 -2.429300 0.122000
C 1.271100 -1.906800 -0.110400
C 2.497400 -1.123900 -0.434800
C 3.742400 -1.668500 -0.103200
C 2.434100 0.133300 -1.045800
C 4.908200 -0.966900 -0.376800
C 3.603500 0.834400 -1.320000
C 4.839200 0.286200 -0.985500
O 0.691000 -0.365800 2.334600
O -2.821600 -1.590200 -1.449400
O -3.466400 -2.172400 0.624100
O 1.322300 -3.008100 0.402900
H -0.714600 4.319400 0.815200
H -2.012500 4.770100 -1.256400
H -2.722100 2.918200 -2.726900
H -2.164700 0.562500 -2.143800
H -0.200600 3.088100 3.133000
H 1.084500 1.866500 3.308900
H 1.298700 3.225700 2.177600
H -4.754600 -3.209200 -0.640900
H -5.365900 -2.757800 0.980500
H -5.211300 -1.519800 -0.304300
H -1.091600 -1.998600 1.324000
H 3.775000 -2.642800 0.372100
H 1.477800 0.572700 -1.311200
H 5.871400 -1.392600 -0.116100
H 3.549400 1.808900 -1.793300
H 5.750400 0.835700 -1.198600
H -0.248400 -1.240600 -1.557100

Structure **5c**
N -3.256300 -1.139600 -0.655700
C -1.833200 0.185600 0.580800
C -3.166100 -0.208700 0.389300
C -4.203400 0.284000 1.164000
C -3.879900 1.203900 2.163800
C -2.562300 1.601900 2.372000
C -1.527100 1.092400 1.583000
C -2.026100 -1.398200 -1.190600
C -1.029000 -0.542000 -0.439200
C 0.074300 0.037900 -1.340000
C 0.370700 -1.071200 -0.375200
C -4.482100 -1.757800 -1.106600
C 1.252300 -0.905500 0.842500
C 2.723000 -0.796100 0.614100
C 3.557100 -0.658000 1.728300
C 3.283900 -0.835700 -0.666000
C 4.931900 -0.556400 1.565400
C 4.661900 -0.738100 -0.827700
C 5.485700 -0.596800 0.286200
C 0.578000 1.412400 -1.071800
C 0.968800 3.514000 -2.029800
O -1.777100 -2.165800 -2.106900
O 0.781200 -0.945100 1.963200
O 1.003000 1.807700 -0.006000
O 0.507400 2.163700 -2.169100
H -5.228000 -0.032700 1.003100
H -4.670400 1.608100 2.787600
H -2.332000 2.312400 3.158600
H -0.502600 1.396100 1.750400
H -5.185100 -0.999800 -1.462700
H -4.232300 -2.432500 -1.924800
H -4.946300 -2.326000 -0.295900
H 0.494700 -2.040200 -0.857200
H 3.108200 -0.630200 2.715100
H 2.652200 -0.944200 -1.541200
H 5.573900 -0.446200 2.433000
H 5.092100 -0.771100 -1.823000
H 6.560500 -0.518000 0.158100
H -0.014900 -0.227600 -2.387900
H 0.371000 4.044000 -1.286300
H 2.017900 3.526900 -1.729000
H 0.847300 3.967600 -3.011000

Structure **$ScBr_3Py_3$**
Sc -0.000000 -0.392100 -0.000000
C 0.851200 2.609700 0.778600
C -0.851200 2.609600 -0.778700
C 0.887500 3.994500 0.803000
H 1.515500 2.024100 1.403300
C -0.887600 3.994500 -0.803100
H -1.515500 2.024000 -1.403400
C -0.000100 4.702700 -0.000000
H 1.597300 4.500100 1.446100
H -1.597400 4.500000 -1.446100
H -0.000100 5.787200 -0.000000
C -3.044500 -0.975200 -0.671200
C -2.869600 0.666800 0.945500
C -4.428500 -0.912900 -0.644200
H -2.527500 -1.660700 -1.333000
C -4.246300 0.794100 1.026400
H -2.214500 1.265600 1.568100
C -5.042400 -0.010400 0.217400
H -5.006200 -1.561600 -1.290900
H -4.679100 1.509100 1.715100
H -6.123500 0.063900 0.259300
N 0.000000 1.916900 -0.000000
N -2.268900 -0.199200 0.108100
Br -0.128600 -0.247600 -2.645700
Br 0.000000 -3.010000 0.000000
C 2.869600 0.666900 -0.945400
C 3.044500 -0.975300 0.671000
C 4.246300 0.794300 -1.026200
H 2.214500 1.265800 -1.567900
C 4.428600 -0.913000 0.644100

H 2.527500 -1.661000 1.332800
C 5.042400 -0.010400 -0.217400
H 4.679100 1.509300 -1.714800
H 5.006300 -1.561800 1.290700
H 6.123500 0.064000 -0.259300
Br 0.128600 -0.247500 2.645700
N 2.268900 -0.199200 -0.108100

Structure **Py**
C -1.195500 0.671000 0.000000
C -1.140300 -0.719800 0.000100
C 1.140400 -0.719700 -0.000000
C 1.195400 0.671100 -0.000000
C -0.000100 1.381700 -0.000000
H -2.153300 1.179200 -0.000000
H -2.058700 -1.302300 -0.000000
H 2.058900 -1.302100 -0.000100
H 2.153100 1.179400 -0.000000
H -0.000100 2.466800 -0.000000
N 0.000100 -1.418200 -0.000000

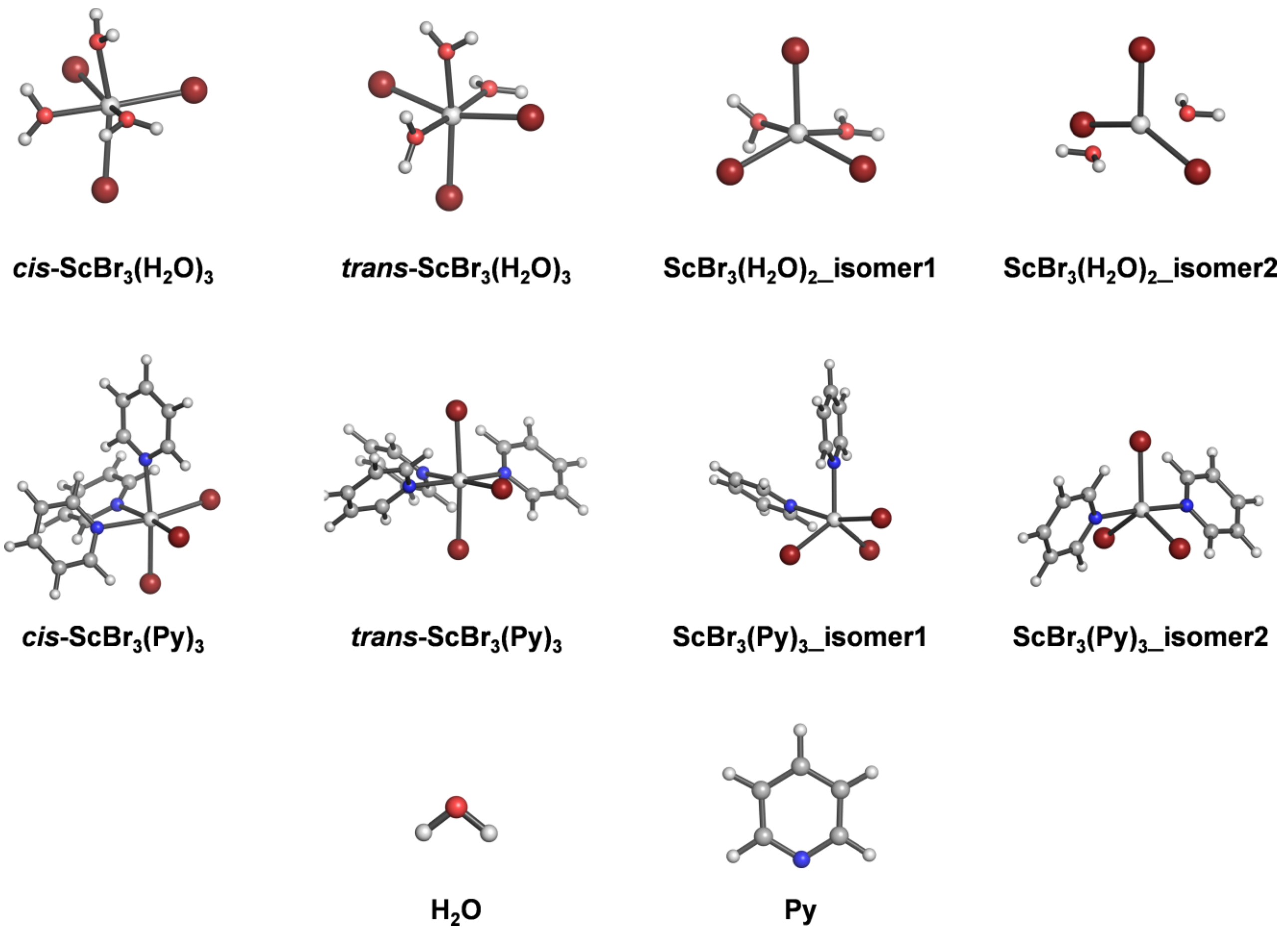

Figure S2. Lowest energy geometries for Sc complexes featuring bromide, water and pyridine ligands calculated with PCM(pyridine)/ωB97X-D/6-31G(d,p)+LANL2DZ(Sc,Br). The calculated reaction energy of the isodesmic reaction $ScBr_3(H_2O)_3$ + 3 Py → $ScBr_3(Py)_3$ + 3 H_2O, which accounts for the substitution of coordinated water molecules by pyridines, is ΔG ≈ +6 kcal mol^{-1}, which reflects its endergonic character and the irreversible binding of water to Sc. Of note, the calculated reaction enthalpy is negative (ΔH ≈ –7 kcal mol^{-1}), due to the much tighter binding of the water molecules compared to pyridine. Hence, such ligand exchange is forbidden by entropy despite the same number of bonds being cleaved and formed. These results strongly suggests that not only the strength of the Sc···O bond (enthalpy) but, much more importantly, its tightness (entropy) determines the thermodynamics of the reaction.

Table S3. Energies, entropies, and lowest frequencies of the lowest energy calculated structures reported in **Figure S2**.[a]

Structure	E_{elec} (Hartree)	E_{elec} + ZPE (Hartree)	H (Hartree)	S (cal mol^{-1} K^{-1})	G (Hartree)	Lowest freq. (cm^{-1})	# imag. freq.
***cis*–$ScBr_3(H_2O)_3$**	–315.567457	–315.488024	–315.468826	132.4223	–315.539524	45.83	0
***trans*–$ScBr_3(H_2O)_3$**	–315.567872	–315.490055	–315.469954	136.7563	–315.543067	56.95	0
**$ScBr_3(H_2O)_2$_isomer1**	–239.126834	–239.073220	–239.057294	120.9400	–239.121560	48.51	0
**$ScBr_3(H_2O)_2$_isomer2**	–239.130711	–239.077761	–239.061589	119.1266	–239.126313	75.66	0
***cis*–$ScBr_3(Py)_3$**	–830.979031	–830.699556	–830.668653	187.5637	–830.763857	23.56	0
***trans*–$ScBr_3(Py)_3$**	–830.986690	–830.707168	–830.676367	185.7094	–830.771558	31.54	0
**$ScBr_3(Py)_3$_isomer1**	–582.740271	–582.552203	–582.529154	153.4016	–582.608464	30.17	0
**$ScBr_3(Py)_3$_isomer2**	–582.741120	–582.553673	–582.530291	156.2338	–582.610389	22.32	0
**H_2O**	–76.404530	–76.382735	–76.378381	46.2169	–76.403655	1669.65	0
**Py**	–248.207339	–248.117411	–248.110844	71.2444	–248.149804	391.32	0

[a]Energy values calculated at the PCM(pyridine)/ωB97X-D/6-31G(d,p) level. 1 Hartree = 627.51 kcal mol^{-1}. Thermal corrections at 343.15 K.

Table S4. Cartesian coordinates of the lowest energy structures of **Figure S2** calculated with PCM(pyridine)/ ωB97X-D /6-31G(d,p)+LANL2DZ(Sc,Br)

***cis*-ScBr$_3$(H$_2$O)$_3$**

Sc	-0.00289	-0.00308	0.46465
O	-128.341	0.89167	200.294
H	-218.735	116.769	180.781
O	-0.13369	-158.753	197.260
H	-0.88558	-158.953	257.833
O	143.017	0.64875	198.958
H	180.544	-0.01179	258.524
H	0.07538	-250.498	175.786
H	211.909	129.435	178.829
H	-0.89953	153.818	260.839
Br	-221.502	-0.71191	-0.67391
Br	173.124	-154.179	-0.67824
Br	0.48174	226.935	-0.66512

***trans*-ScBr$_3$(H$_2$O)$_3$**

Sc	-0.00012	-0.24374	0.07013
O	0.00014	-0.08939	220.317
O	0.00001	-0.56390	-205.476
H	0.78581	-0.48133	-260.935
O	-0.00059	-236.149	0.58686
H	0.78450	-291.758	0.50881
H	-0.78641	-291.650	0.50843
H	-0.78521	-0.48249	-261.033
H	0.00256	0.72416	272.106
H	0.00163	-0.84462	280.320
Br	0.00044	233.298	-0.00995
Br	-259.065	-0.64965	-0.11892
Br	259.031	-0.65032	-0.11904

ScBr$_3$(H$_2$O)$_2$_isomer1

Sc	-0.05333	-0.03635	0.49022
Br	-169.002	-141.026	-0.85955
Br	-0.23589	239.109	-0.30415
Br	230.199	-0.63124	-0.33948
O	0.40032	-144.626	206.211
H	111.364	-209.422	201.073
O	-153.750	0.32028	206.235
H	-245.419	0.49811	181.308
H	-133.999	0.89423	281.317
H	-0.26472	-176.282	268.424

ScBr$_3$(H$_2$O)$_2$_isomer2

Sc	0.00000	0.00000	-0.17733
Br	243.608	-0.00033	-0.98318
Br	-243.608	0.00033	-0.98318
O	0.00000	211.977	-0.49286
H	0.78397	268.265	-0.46770
H	-0.78370	268.296	-0.46671
Br	0.00000	0.00000	235.146
O	0.00000	-211.977	-0.49286
H	0.78370	-268.296	-0.46671
H	-0.78397	-268.265	-0.46770

***cis*-ScBr$_3$(Py)$_3$**

Sc	-0.02880	-0.27961	-0.47218
N	166.720	-0.38075	112.948
C	205.789	0.64719	190.678
C	237.572	-152.349	121.166
C	313.225	0.57545	277.798
H	148.750	156.428	182.675
C	346.399	-167.760	205.661
H	205.095	-233.718	0.57468
C	385.291	-0.61104	285.760
H	339.272	143.949	337.676
H	399.138	-262.332	207.680
H	470.020	-0.70080	352.831
N	0.03669	199.780	-0.12827
C	113.046	272.493	-0.42821
C	-105.898	265.419	0.29788
C	117.294	410.402	-0.29963
H	199.179	217.030	-0.78260
C	-109.614	402.955	0.45649
H	-193.402	205.079	0.50515
C	0.04095	477.134	0.15439
H	208.079	463.632	-0.55502
H	-200.521	450.311	0.80598
H	0.04264	584.993	0.26622
N	-153.643	-0.30865	128.918
C	-277.368	-0.79295	106.416
C	-123.897	0.08890	253.882
C	-373.577	-0.88616	205.672
H	-298.875	-110.810	0.05023
C	-214.235	0.02748	358.748
H	-0.24072	0.47007	270.500
C	-341.805	-0.46854	334.425
H	-471.459	-128.131	181.447
H	-184.211	0.36382	457.221
H	-414.961	-0.52958	414.238
Br	194.519	-0.23389	-220.450
Br	-0.38989	-289.251	-0.51652
Br	-192.700	0.27685	-220.818

***trans*-ScBr$_3$(Py)$_3$**

Sc	0.00071	-0.39276	0.00009
N	-226.820	-0.19985	-0.10713
C	-286.927	0.66535	-0.94507
C	-304.355	-0.97606	0.67228
C	-424.604	0.79173	-102.637
H	-221.434	126.421	-156.779
C	-442.763	-0.91464	0.64490
H	-252.630	-166.082	133.472
C	-504.182	-0.01293	-0.21726
H	-467.901	150.615	-171.555
H	-500.509	-156.336	129.179
H	-612.297	0.06067	-0.25958
N	226.910	-0.19643	0.10771
C	286.951	0.67017	0.94464
C	304.505	-0.97255	-0.67125
C	424.617	0.79810	102.537
H	221.416	126.899	156.695
C	442.906	-0.90964	-0.64436
H	252.834	-165.854	-133.283
C	504.256	-0.00647	0.21678
H	467.858	151.364	171.373
H	500.702	-155.835	-129.082
H	612.364	0.06831	0.25870
N	-0.00224	191.647	0.00054
C	0.84777	261.010	-0.77860
C	-0.85384	260.825	0.77955
C	0.88242	399.498	-0.80318
H	151.243	202.522	-140.355

C	-0.89182	399.304	0.80379
H	-151.706	202.188	140.463
C	-0.00557	470.222	0.00020
H	159.129	450.132	-144.665
H	-160.189	449.783	144.715
H	-0.00691	578.669	0.00003
Br	0.00195	-301.032	-0.00046
Br	0.12841	-0.24740	-264.592
Br	-0.12708	-0.24848	264.583

$ScBr_3(Py)_3$_isomer1

Sc	0.54828	-0.28257	-0.00822
Br	107.104	-174.029	-207.558
Br	282.920	0.97658	0.05480
Br	0.68680	-136.237	233.503
C	-229.124	-160.942	-0.88284
C	-245.094	-0.28061	0.99698
C	-364.732	-190.196	-0.83913
H	-165.542	-202.022	-165.782
C	-380.735	-0.52594	111.163
H	-195.311	0.36279	171.458
C	-442.090	-135.307	0.17495
H	-407.839	-255.045	-159.179
H	-436.663	-0.07473	192.186
H	-548.279	-156.390	0.23736
C	-0.24896	267.118	0.71519
C	-115.880	204.298	-131.894
C	-0.85486	391.481	0.65040
H	0.37712	240.173	155.895
C	-178.479	327.004	-146.286
H	-126.955	127.245	-207.656
C	-163.420	422.219	-0.45962
H	-0.70827	462.424	145.513
H	-238.240	346.660	-234.417
H	-211.769	518.922	-0.54142
N	-0.39785	174.490	-0.24861
N	-169.172	-0.81091	0.01762

$ScBr_3(Py)_3$_isomer2

Sc	-0.00277	0.06555	-0.02556
Br	-0.23285	-0.98722	232.334
Br	0.28221	-140.517	-213.182
Br	-0.03812	262.932	-0.26496
C	306.832	0.97909	-0.39152
C	288.952	-103.940	0.71305
C	445.238	0.90577	-0.38536
H	255.857	182.481	-0.83705
C	426.562	-118.815	0.75620
H	223.925	-179.097	114.538
C	506.573	-0.19600	0.19981
H	503.096	170.481	-0.83206
H	469.331	-206.735	122.200
H	614.659	-0.28062	0.22239
C	-304.876	0.91877	0.43938
C	-292.375	-110.731	-0.65931
C	-443.171	0.85862	0.48227
H	-251.771	176.167	0.86607
C	-430.315	-124.031	-0.65843
H	-229.352	-186.738	-110.563
C	-507.464	-0.23832	-0.08134
H	-498.692	166.325	0.94823
H	-475.512	-211.560	-110.830
H	-615.654	-0.31081	-0.06994
N	228.619	0.02690	0.15238
N	-229.355	-0.04222	-0.12844

Py

C	-114.057	0.71964	-0.00000
C	-119.572	-0.67099	0.00000
C	-0.00000	-138.146	-0.00001
C	119.572	-0.67100	-0.00000
C	114.057	0.71964	0.00000
H	-205.930	130.195	0.00001
H	-215.328	-117.926	0.00001
H	-0.00001	-246.667	0.00000
H	215.327	-117.926	0.00000
H	205.931	130.194	0.00000
N	0.00000	141.804	-0.00000

H_2O

O	0.00000	0.00000	0.11888
H	0.00000	0.75516	-0.47550
H	0.00000	-0.75516	-0.47550

6. ^{1}H and ^{13}C NMR spectra of compound 25

NMR characterization has been performed over compound **25** to ensure that only the (*E*)-isomer was formed with such an approach. In this regard, the same procedure illustrated in paragraph 3 of SI has been followed, without adding compound **3**. ^{1}H and ^{13}C spectra confirmed the formation of only the (*E*)-isomer, in agreement with literature characterization.[15]

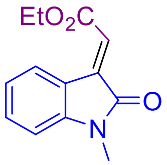

25
300 MHz
DMSO-*d*$_6$

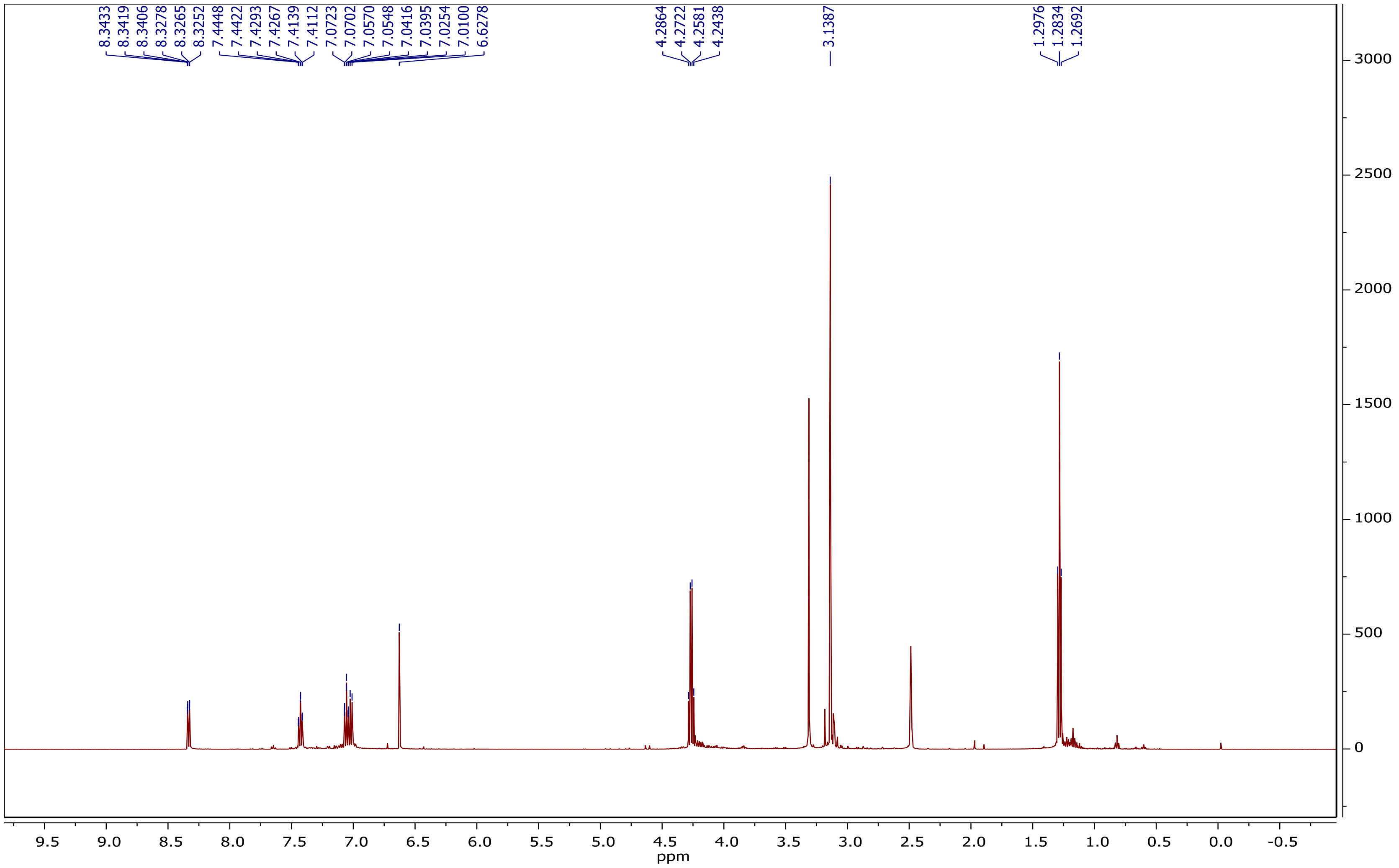

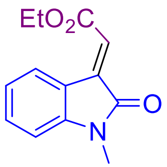

25
126 Mhz
DMSO-d_6

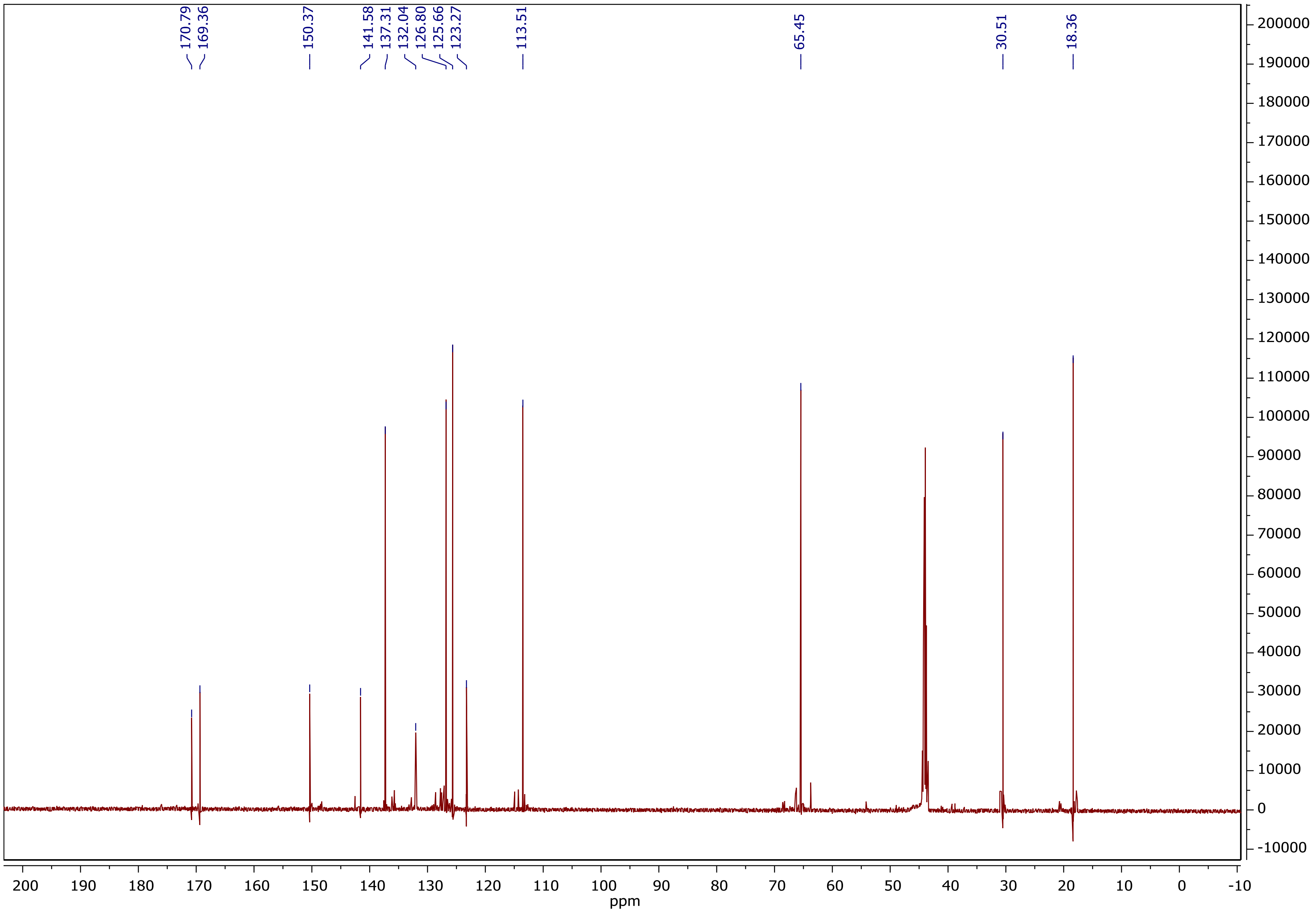

7. GC chromatograms and MS spectra of model reaction crude using different REM triflate salts

Sc(OTf)$_3$ (92:8 *dr*)

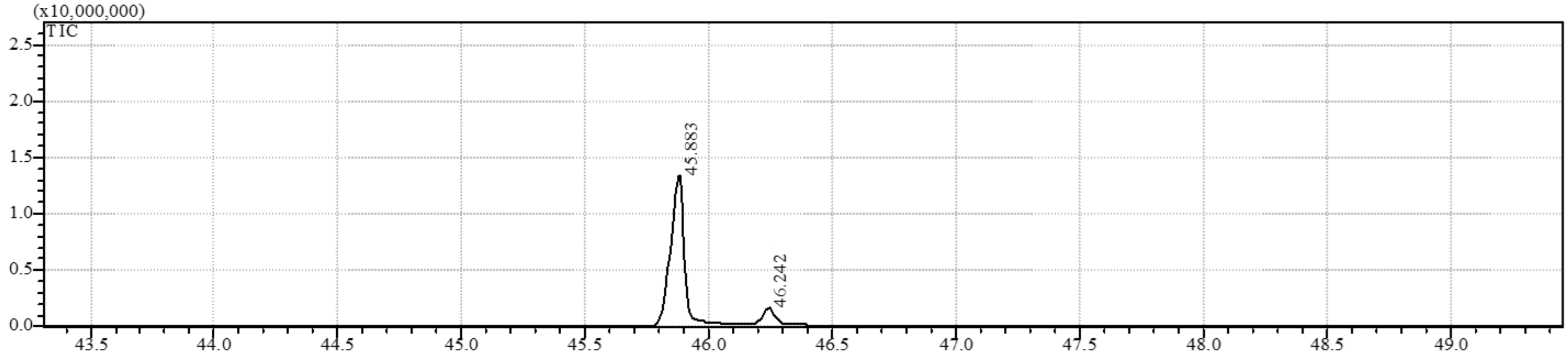

La(OTf)$_3$ (91:9 *dr*)

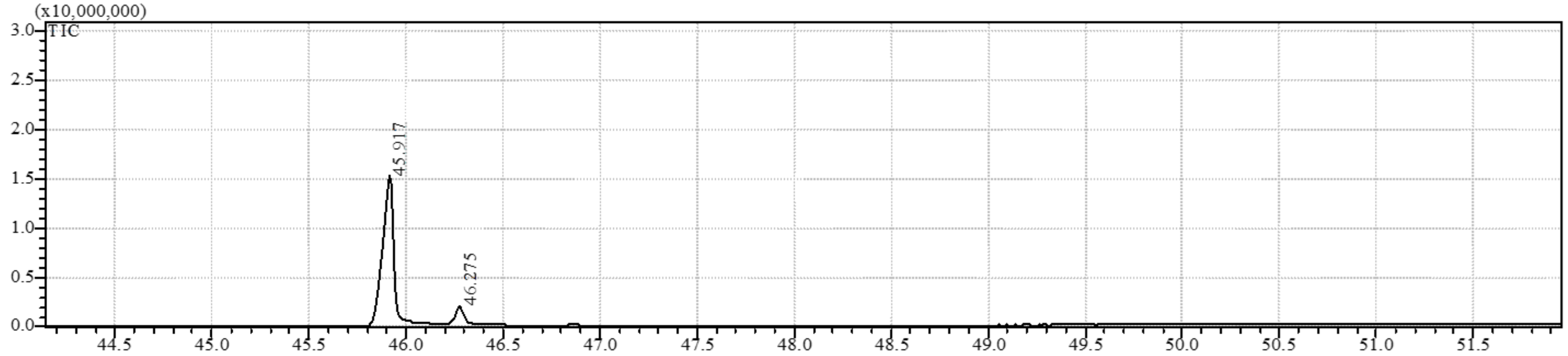

Ce(OTf)$_3$ (90:10 *dr*)

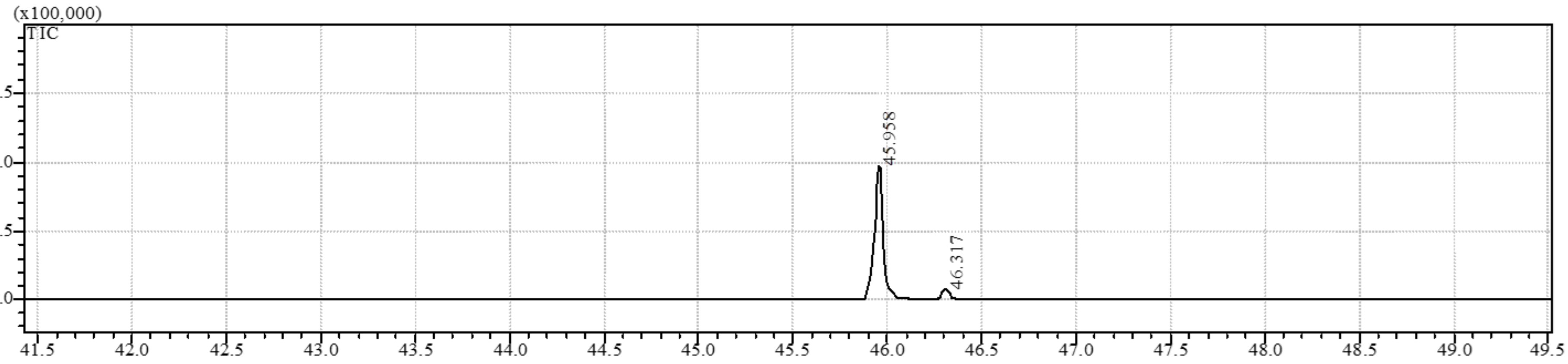

Ho(OTf)$_3$ (88:12 *dr*)

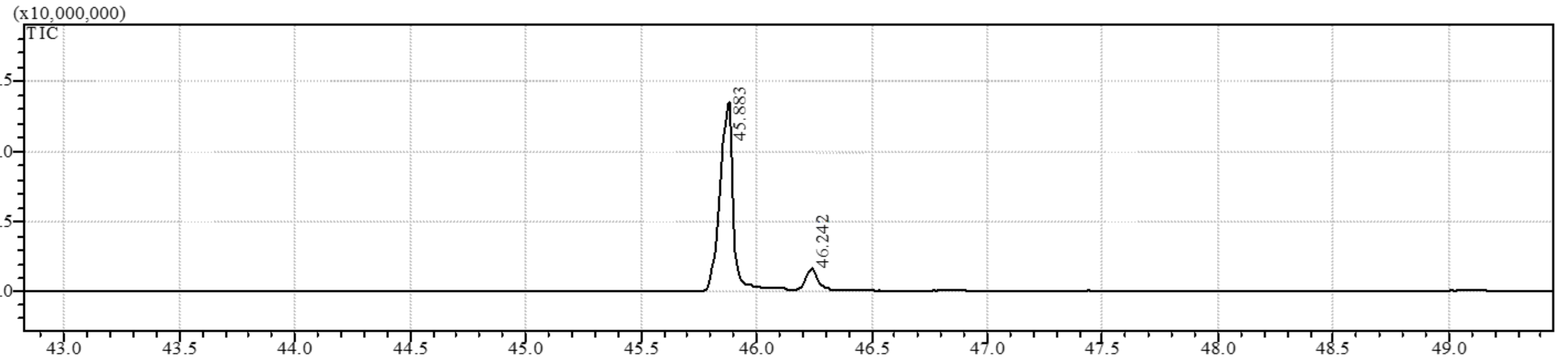

Er(OTf)$_3$ (89:11 *dr*)

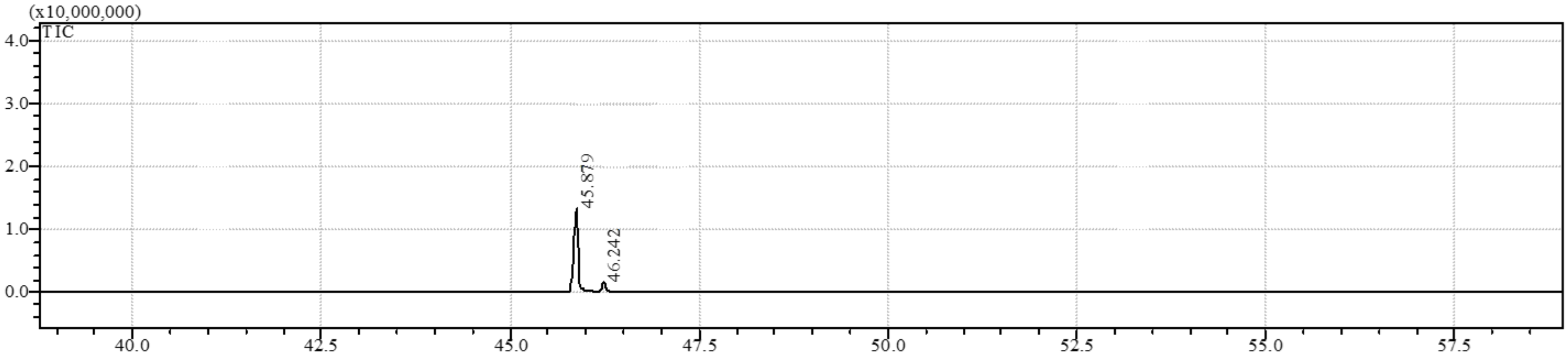

Yb(OTf)$_3$ (90:10 *dr*)

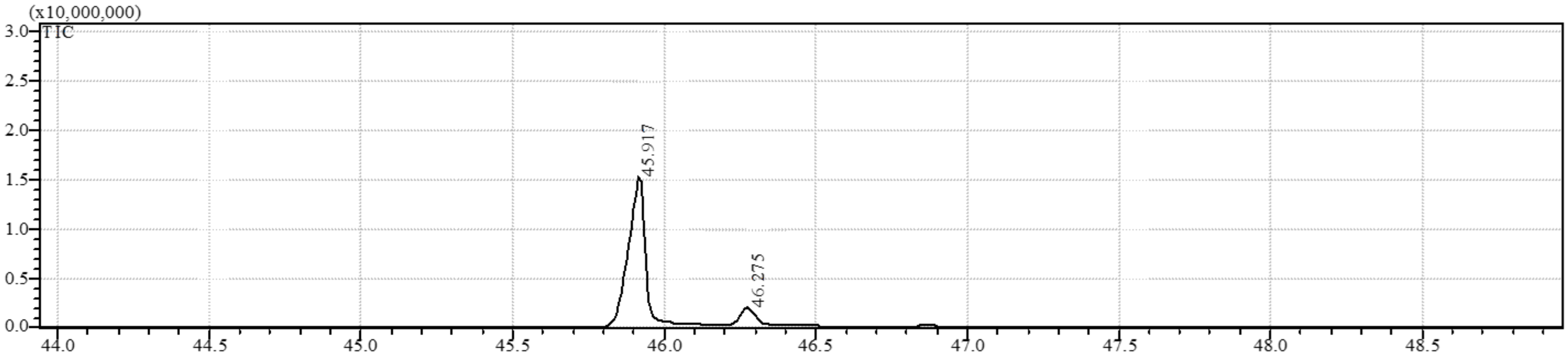

8. HR-MS spectra of the isolated products

5a

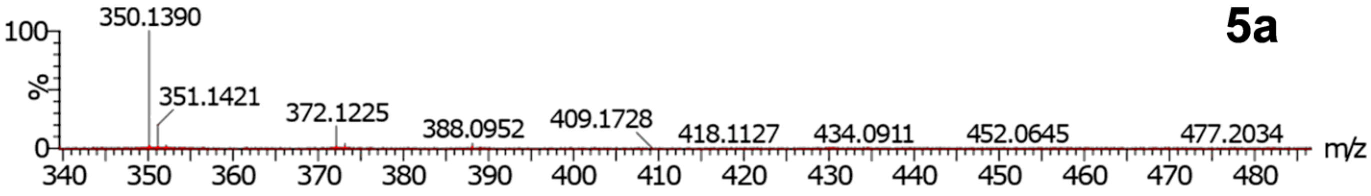

5b

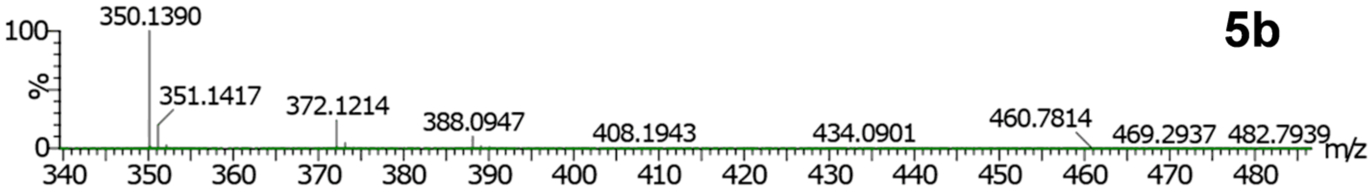

6

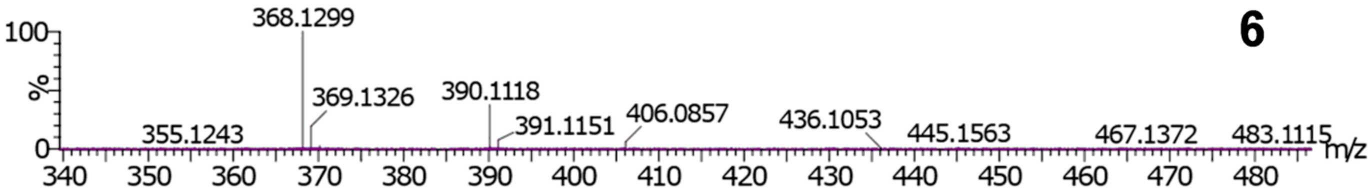

7

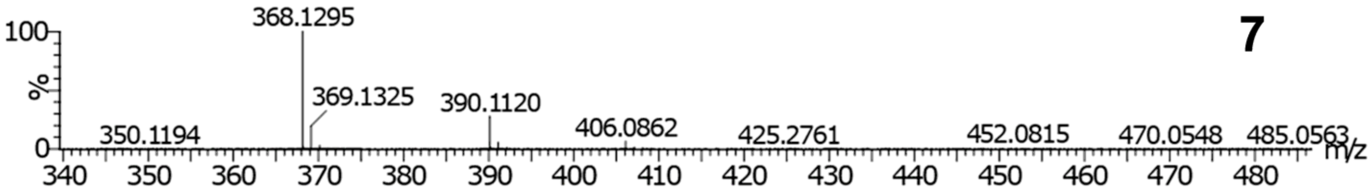

8

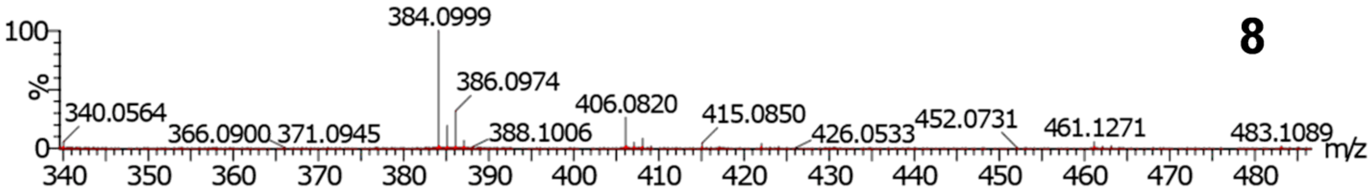

9

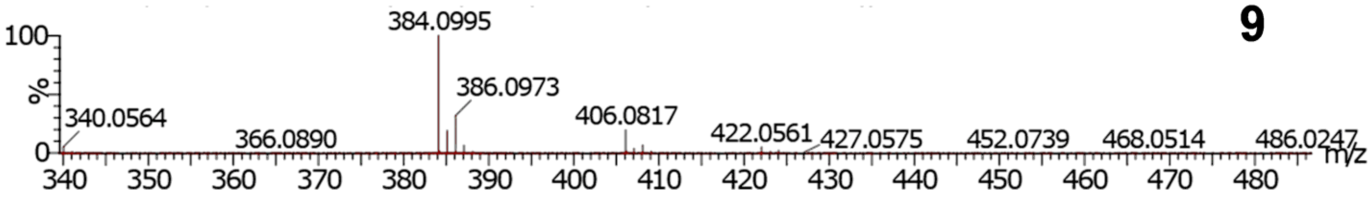

10

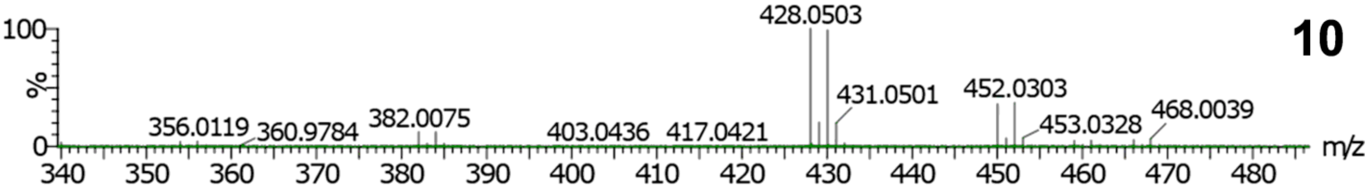
428.0503
431.0501
452.0303
453.0328
468.0039
356.0119
360.9784
382.0075
403.0436
417.0421
100
%
0
m/z
340 350 360 370 380 390 400 410 420 430 440 450 460 470 480

11

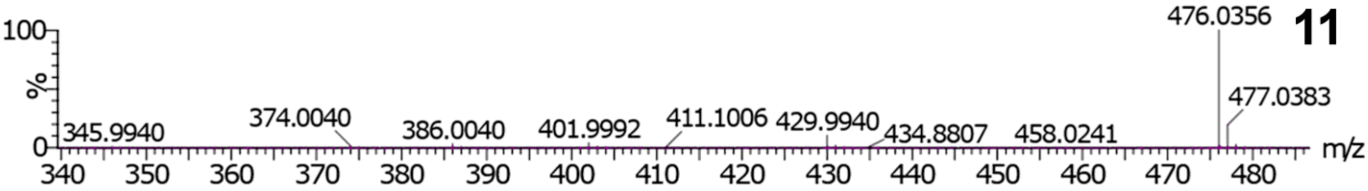
476.0356
477.0383
345.9940
374.0040
386.0040
401.9992
411.1006
429.9940
434.8807
458.0241
100
%
0
m/z
340 350 360 370 380 390 400 410 420 430 440 450 460 470 480

12

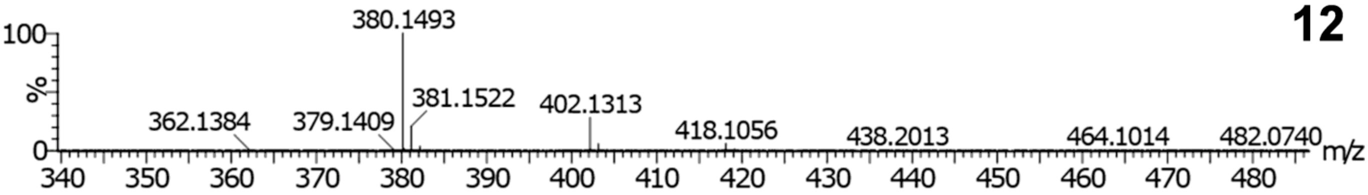
380.1493
381.1522
362.1384
379.1409
402.1313
418.1056
438.2013
464.1014
482.0740
100
%
0
m/z
340 350 360 370 380 390 400 410 420 430 440 450 460 470 480

13

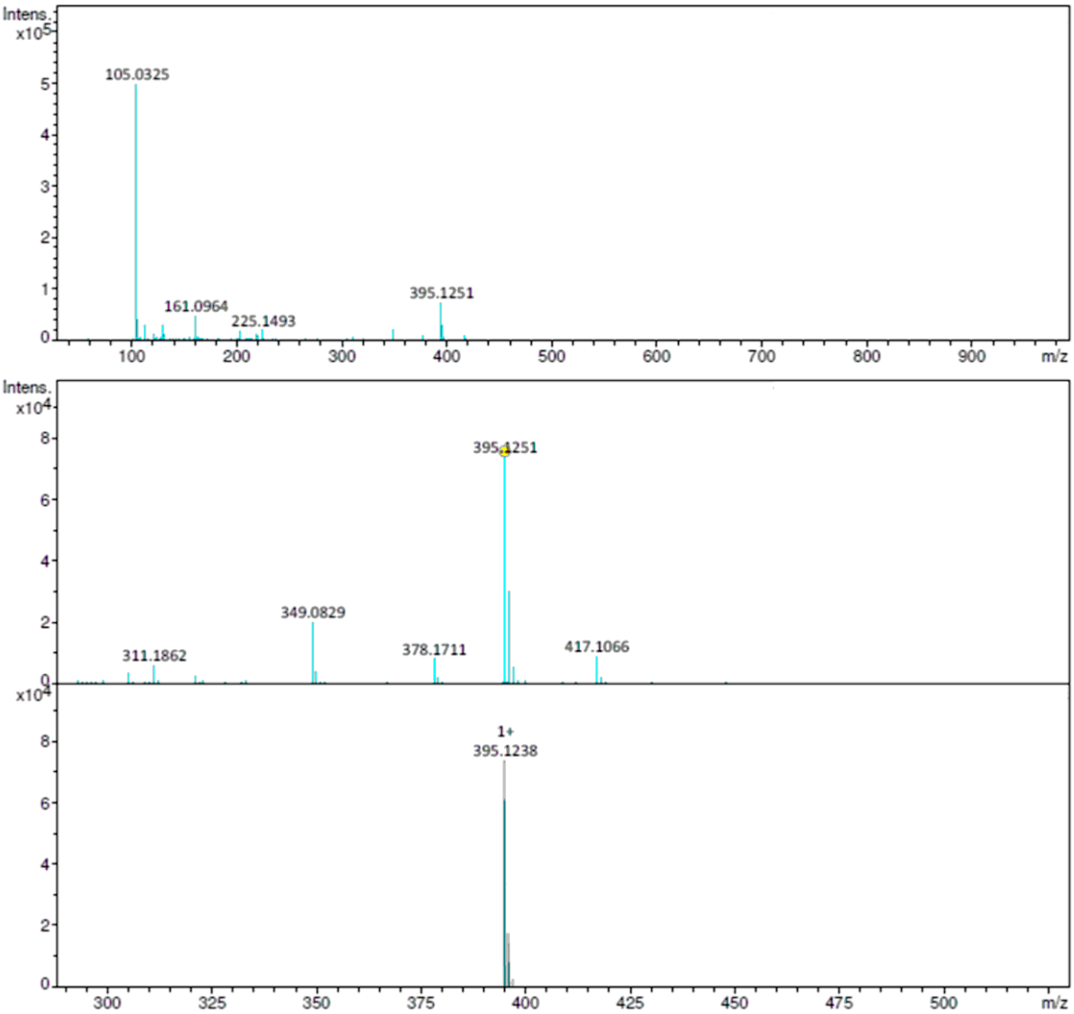
Intens.
x10^5
105.0325
161.0964
225.1493
395.1251
100 200 300 400 500 600 700 800 900 m/z
Intens.
x10^4
395.1251
311.1862
349.0829
378.1711
417.1066
x10^4
1+
395.1238
300 325 350 375 400 425 450 475 500 m/z

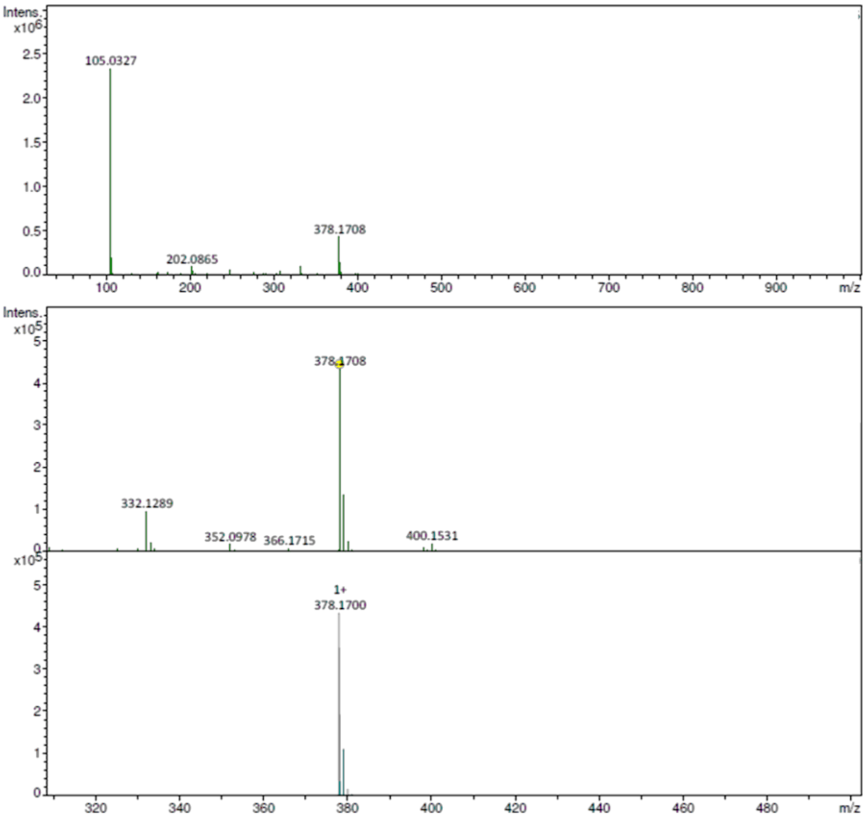
Intens.
x10^6
105.0327
202.0865
378.1708
m/z
x10^5
378.1708
332.1289
352.0978
366.1715
400.1531
1+
378.1700

15

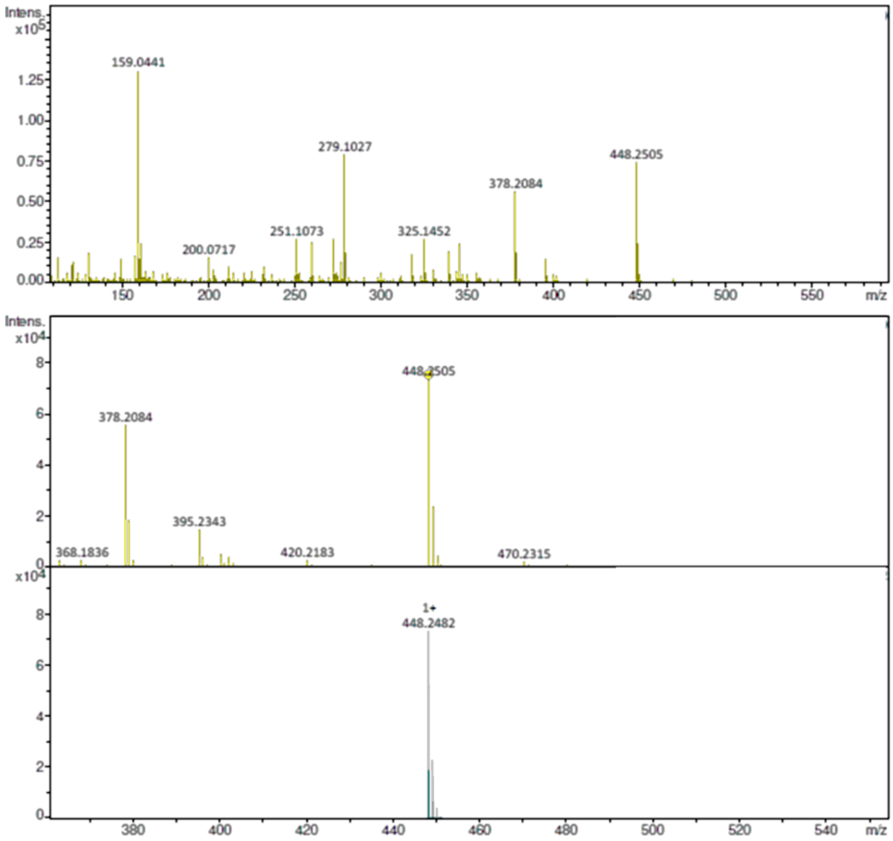
Intens.
x10^5
159.0441
279.1027
448.2505
378.2084
251.1073
325.1452
200.0717
m/z
x10^4
448.2505
378.2084
395.2343
368.1836
420.2183
470.2315
1+
448.2482

16

17

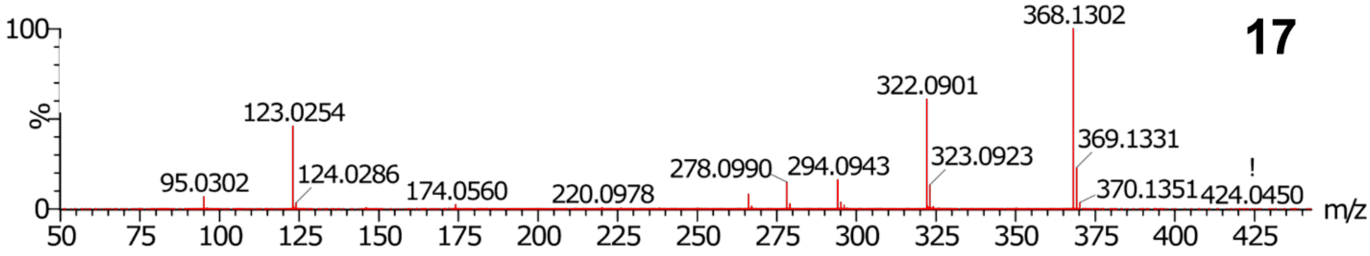

368.1302
322.0901
123.0254
369.1331
95.0302
124.0286
174.0560
220.0978
278.0990
294.0943
323.0923
370.1351
424.0450
100
%
0
m/z
50 75 100 125 150 175 200 225 250 275 300 325 350 375 400 425

18

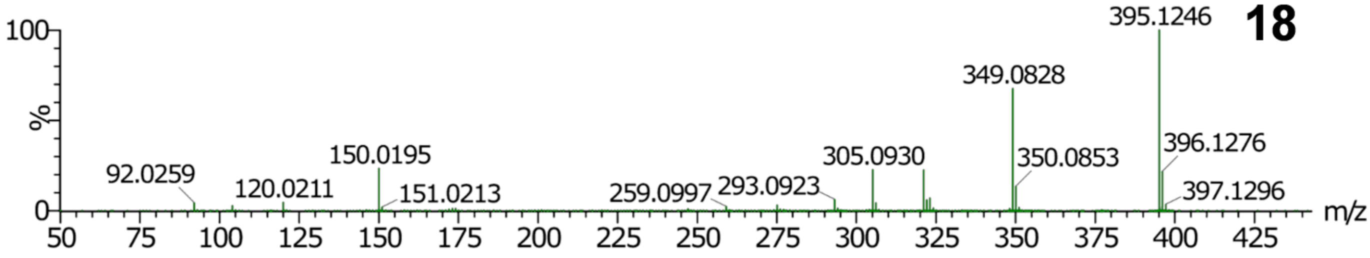

395.1246
349.0828
150.0195
92.0259
120.0211
151.0213
259.0997
293.0923
305.0930
350.0853
396.1276
397.1296
100
%
0
m/z
50 75 100 125 150 175 200 225 250 275 300 325 350 375 400 425

19

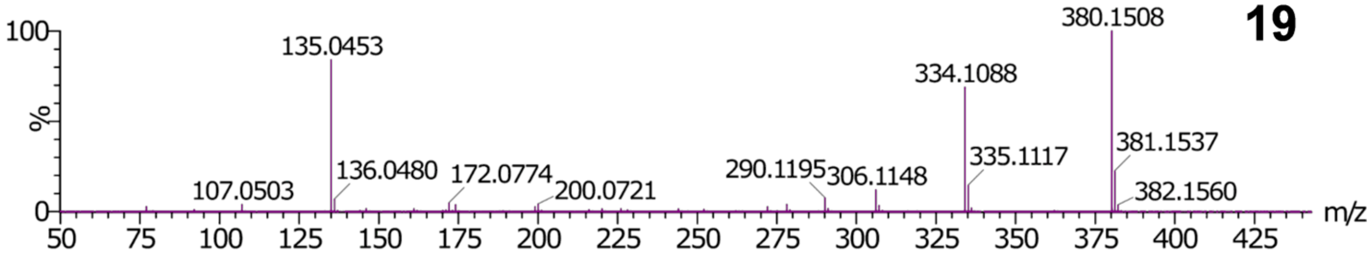

380.1508
135.0453
334.1088
107.0503
136.0480
172.0774
200.0721
290.1195
306.1148
335.1117
381.1537
382.1560
100
%
0
m/z
50 75 100 125 150 175 200 225 250 275 300 325 350 375 400 425

20

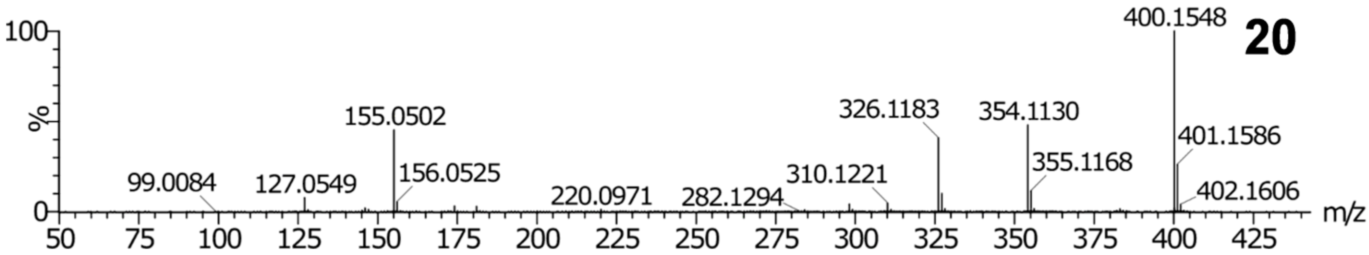

400.1548
155.0502
326.1183
354.1130
99.0084
127.0549
156.0525
220.0971
282.1294
310.1221
355.1168
401.1586
402.1606
100
%
0
m/z
50 75 100 125 150 175 200 225 250 275 300 325 350 375 400 425

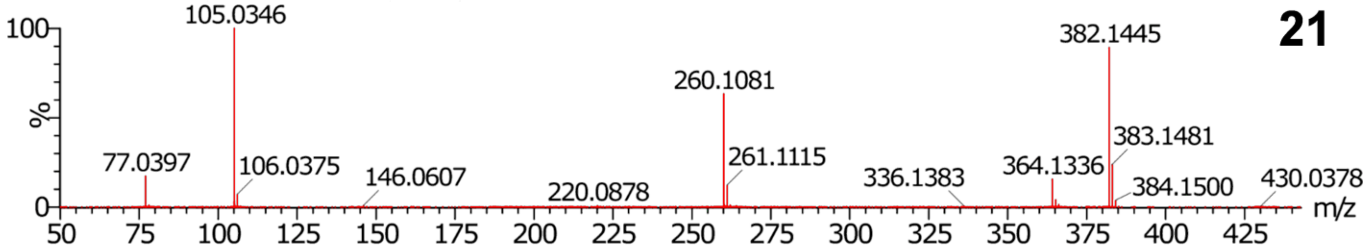
21
100
%
0
105.0346
382.1445
260.1081
77.0397
106.0375
146.0607
220.0878
261.1115
336.1383
364.1336
383.1481
384.1500
430.0378
m/z
50
75
100
125
150
175
200
225
250
275
300
325
350
375
400
425

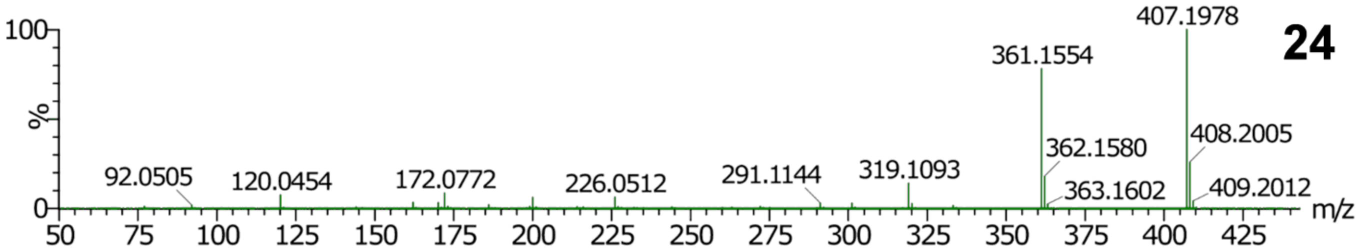
24
100
%
0
407.1978
361.1554
362.1580
408.2005
92.0505
120.0454
172.0772
226.0512
291.1144
319.1093
363.1602
409.2012
m/z
50
75
100
125
150
175
200
225
250
275
300
325
350
375
400
425

9. ^{1}H NMR spectra of the reaction crudes (optimized conditions)

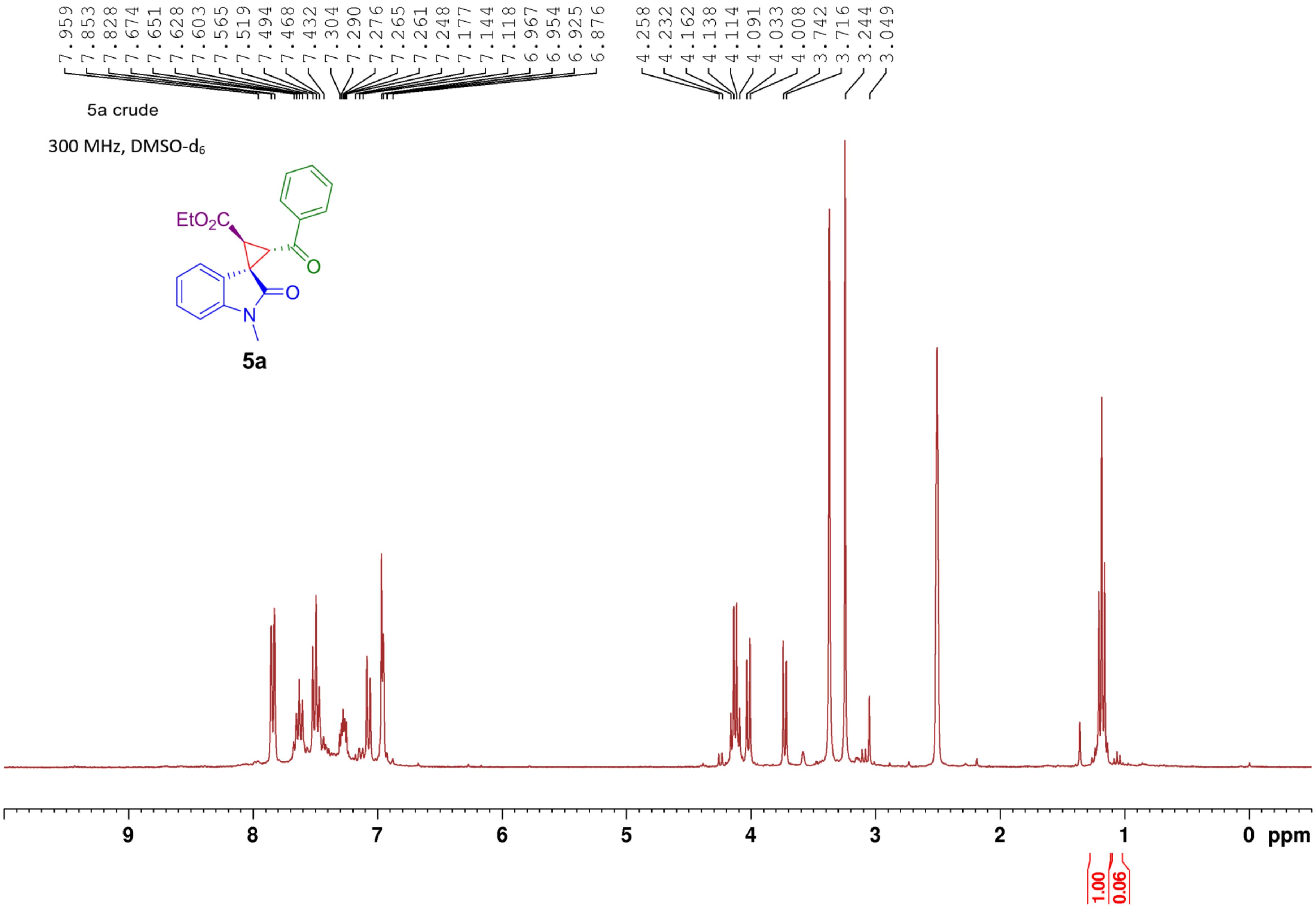

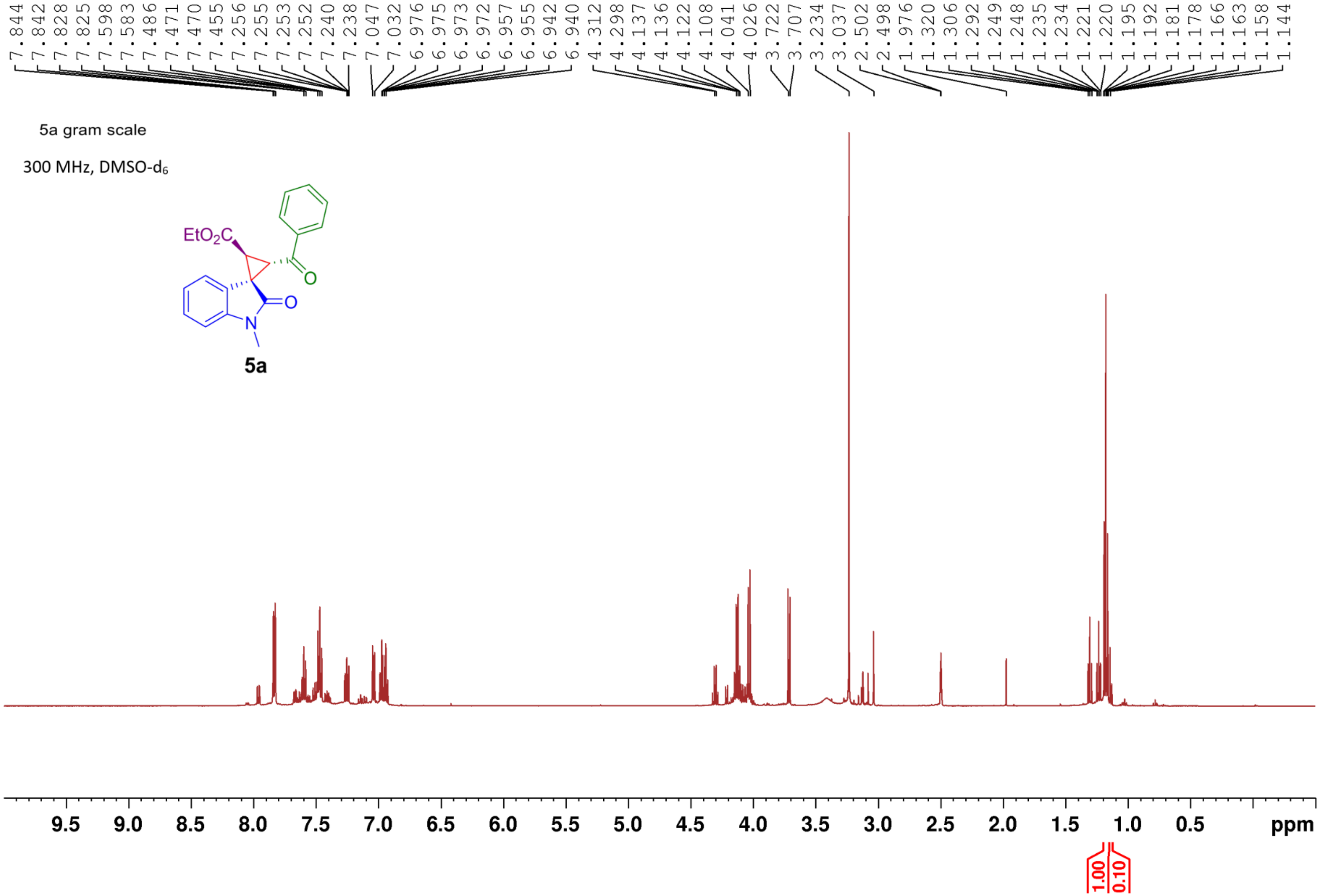

5a gram scale
300 MHz, DMSO-d6
EtO2C
O
N
O
5a
ppm

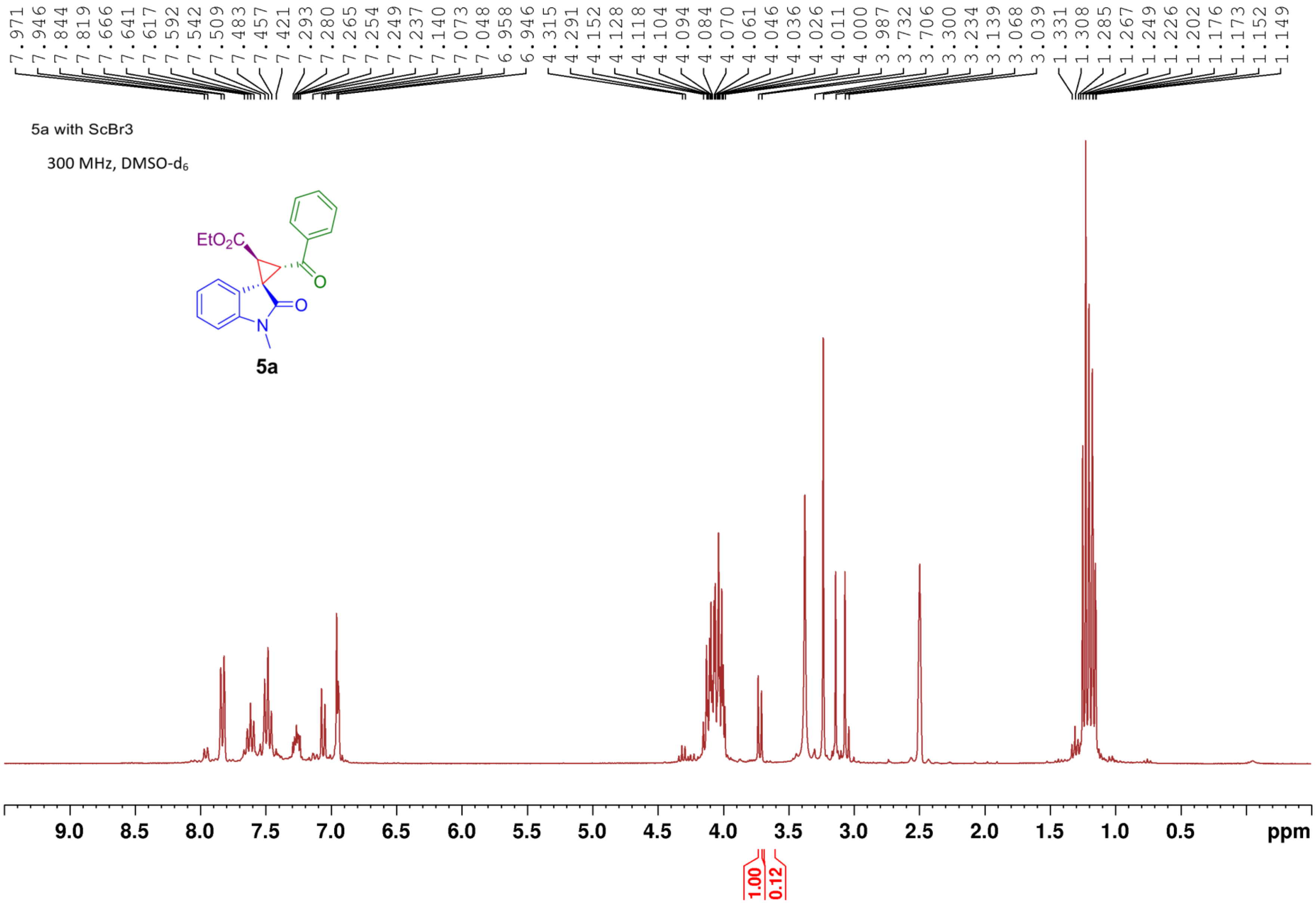
7.971
7.946
7.844
7.819
7.666
7.641
7.617
7.592
7.542
7.509
7.483
7.457
7.421
7.293
7.280
7.265
7.254
7.249
7.237
7.140
7.073
7.048
6.958
6.946
4.315
4.291
4.152
4.128
4.118
4.104
4.094
4.084
4.070
4.061
4.046
4.036
4.026
4.011
4.000
3.987
3.732
3.706
3.300
3.234
3.139
3.068
3.039
1.331
1.308
1.285
1.267
1.249
1.226
1.202
1.176
1.173
1.152
1.149
5a with ScBr3
300 MHz, DMSO-d6
EtO2C
O
O
N
5a
9.0 8.5 8.0 7.5 7.0 6.5 6.0 5.5 5.0 4.5 4.0 3.5 3.0 2.5 2.0 1.5 1.0 0.5 ppm
1.00
0.12

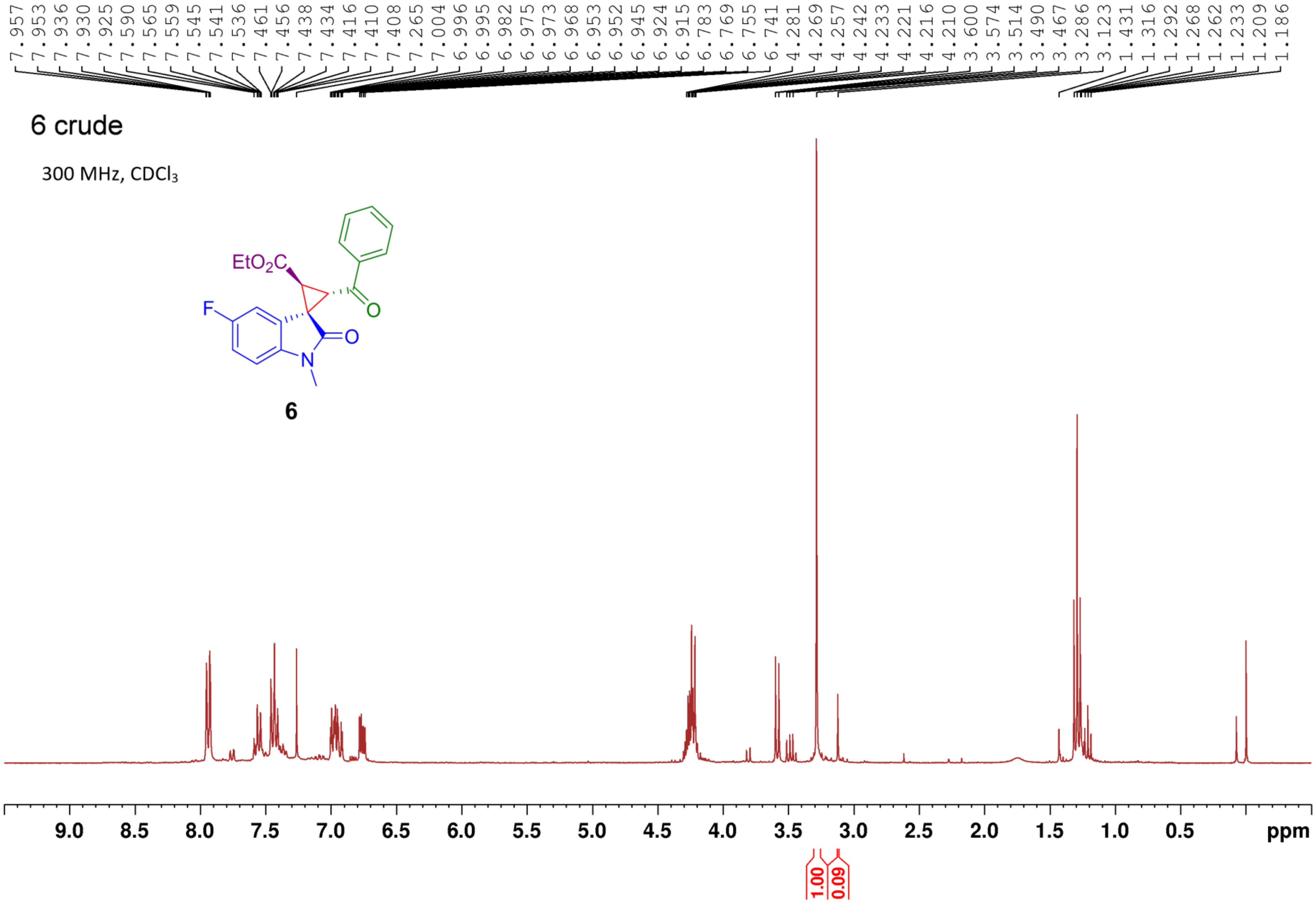
7.957 7.953 7.936 7.930 7.925 7.590 7.565 7.559 7.545 7.541 7.536 7.461 7.456 7.438 7.434 7.416 7.410 7.408 7.265 7.004 6.996 6.995 6.982 6.975 6.973 6.968 6.953 6.952 6.945 6.924 6.915 6.783 6.769 6.755 6.741 4.281 4.269 4.257 4.242 4.233 4.221 4.216 4.210 3.600 3.574 3.514 3.490 3.467 3.286 3.123 1.431 1.316 1.292 1.268 1.262 1.233 1.209 1.186
6 crude
300 MHz, CDCl3
EtO2C
F
O
N
O
6
9.0 8.5 8.0 7.5 7.0 6.5 6.0 5.5 5.0 4.5 4.0 3.5 3.0 2.5 2.0 1.5 1.0 0.5 ppm
1.00
0.09

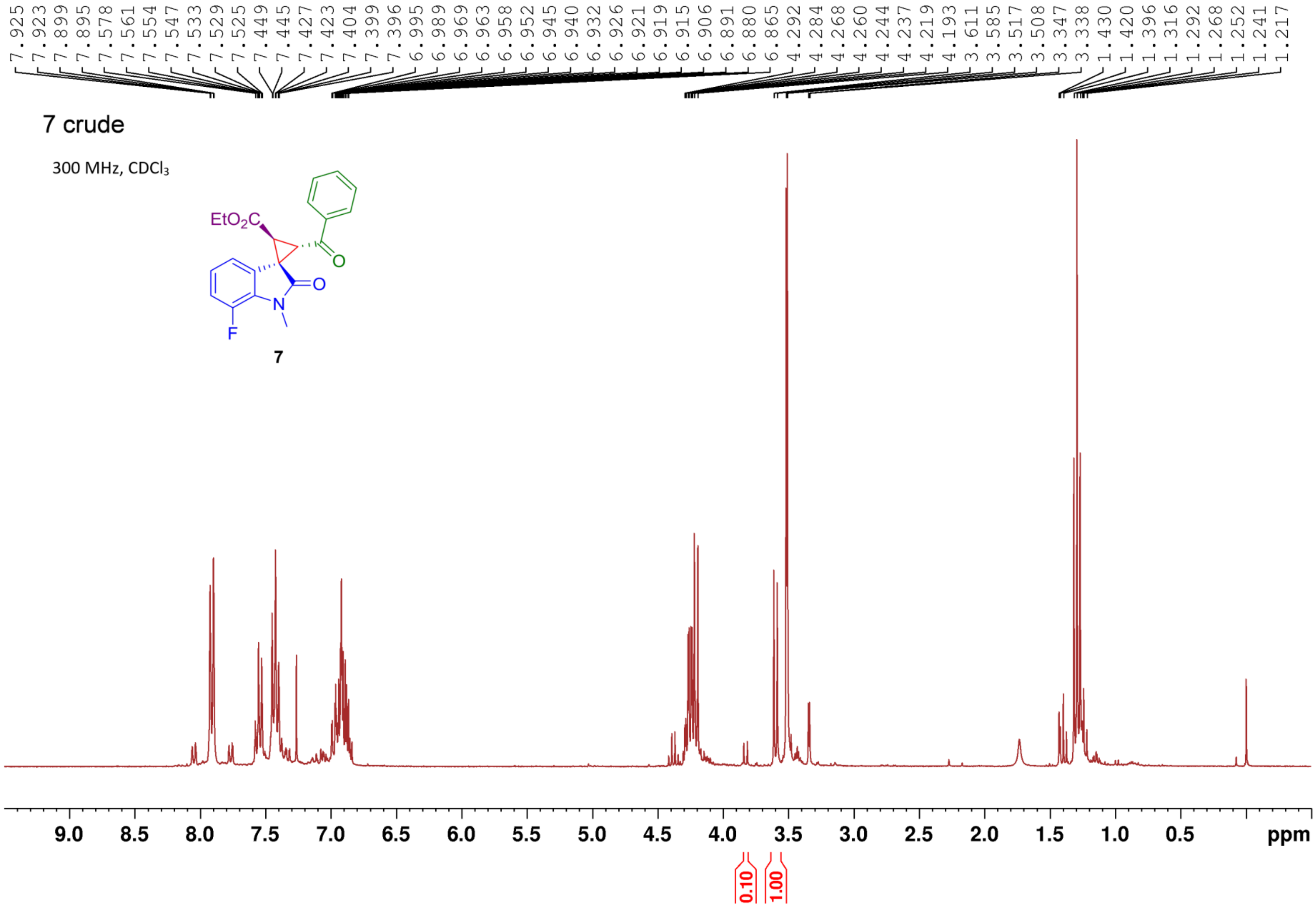
7 crude
300 MHz, $CDCl_3$
EtO_2C
O
O
N
F
7
7.925 7.923 7.899 7.895 7.578 7.561 7.554 7.547 7.533 7.529 7.525 7.449 7.445 7.427 7.423 7.404 7.399 7.396 6.995 6.989 6.969 6.963 6.958 6.952 6.945 6.940 6.932 6.926 6.921 6.919 6.915 6.906 6.891 6.880 6.865 4.292 4.284 4.268 4.260 4.244 4.237 4.219 4.193 3.611 3.585 3.517 3.508 3.347 3.338 1.430 1.420 1.396 1.316 1.292 1.268 1.252 1.241 1.217
9.0 8.5 8.0 7.5 7.0 6.5 6.0 5.5 5.0 4.5 4.0 3.5 3.0 2.5 2.0 1.5 1.0 0.5 ppm
0.10
1.00

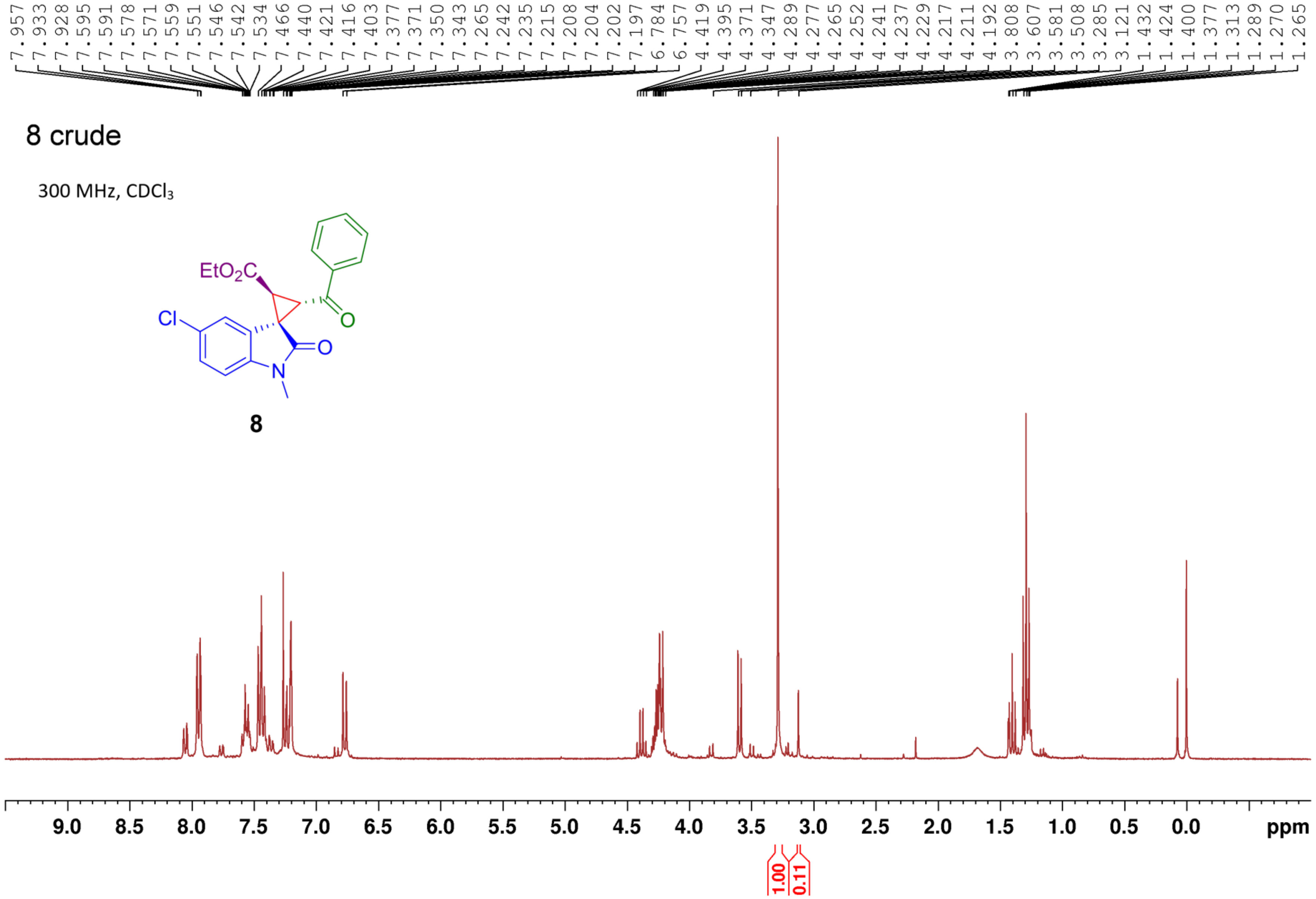
7.957
7.933
7.928
7.595
7.591
7.578
7.571
7.559
7.551
7.546
7.542
7.534
7.466
7.440
7.421
7.416
7.403
7.377
7.371
7.350
7.343
7.265
7.242
7.235
7.215
7.208
7.204
7.202
7.197
6.784
6.757
4.419
4.395
4.371
4.347
4.289
4.277
4.265
4.252
4.241
4.237
4.229
4.217
4.211
4.192
3.808
3.607
3.581
3.508
3.285
3.121
1.432
1.424
1.400
1.377
1.313
1.289
1.270
1.265
8 crude
300 MHz, CDCl3
EtO2C
Cl
O
O
N
8
9.0
8.5
8.0
7.5
7.0
6.5
6.0
5.5
5.0
4.5
4.0
3.5
3.0
2.5
2.0
1.5
1.0
0.5
0.0
ppm
1.00
0.11

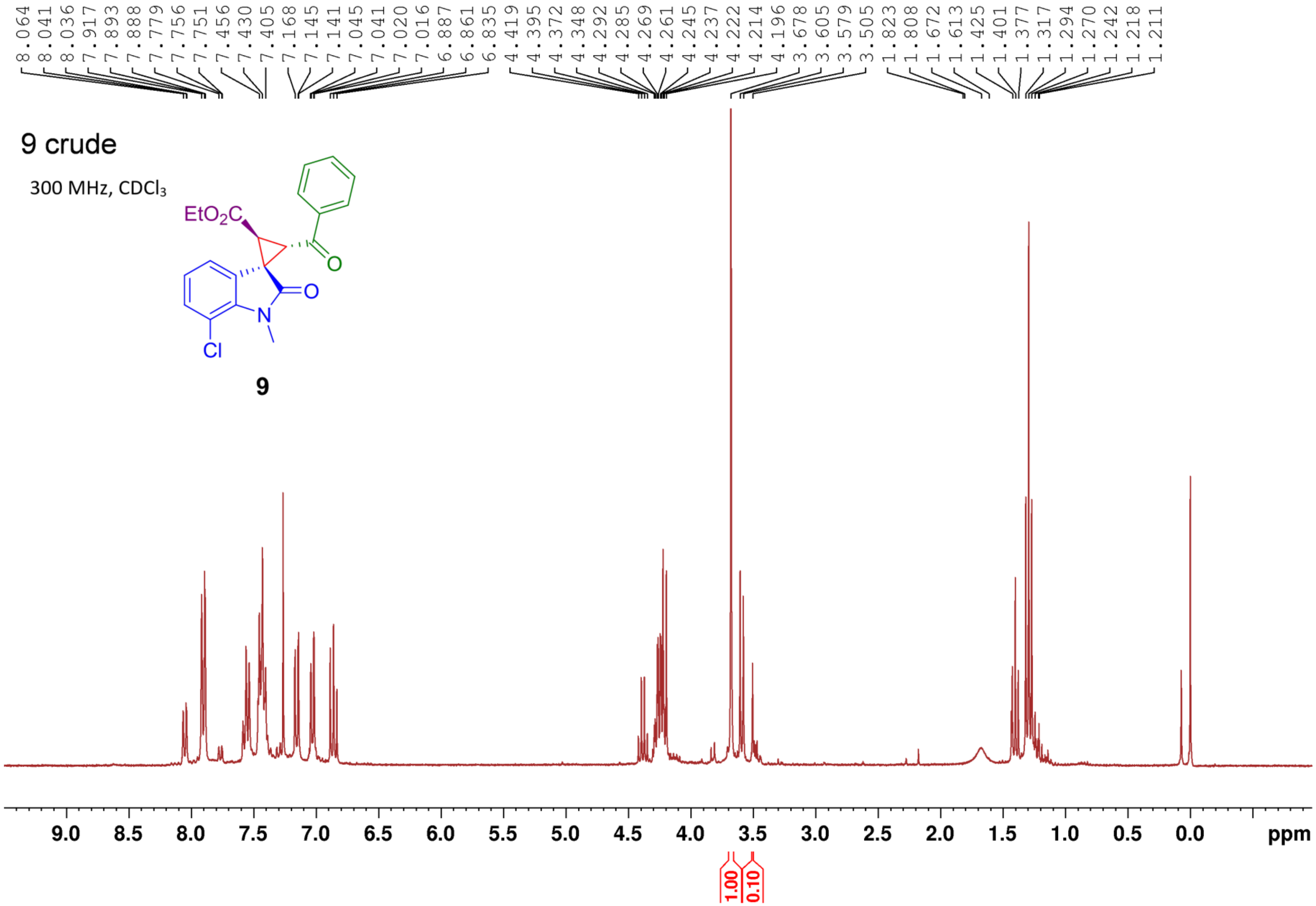
9 crude
300 MHz, CDCl3
EtO2C
O
O
N
Cl
9
8.064
8.041
8.036
7.917
7.893
7.888
7.779
7.756
7.751
7.456
7.430
7.405
7.168
7.145
7.141
7.045
7.041
7.020
7.016
6.887
6.861
6.835
4.419
4.395
4.372
4.348
4.292
4.285
4.269
4.261
4.245
4.237
4.222
4.214
4.196
3.678
3.605
3.579
3.505
1.823
1.808
1.672
1.613
1.425
1.401
1.377
1.317
1.294
1.270
1.242
1.218
1.211
9.0
8.5
8.0
7.5
7.0
6.5
6.0
5.5
5.0
4.5
4.0
3.5
3.0
2.5
2.0
1.5
1.0
0.5
0.0
ppm
1.00
0.10

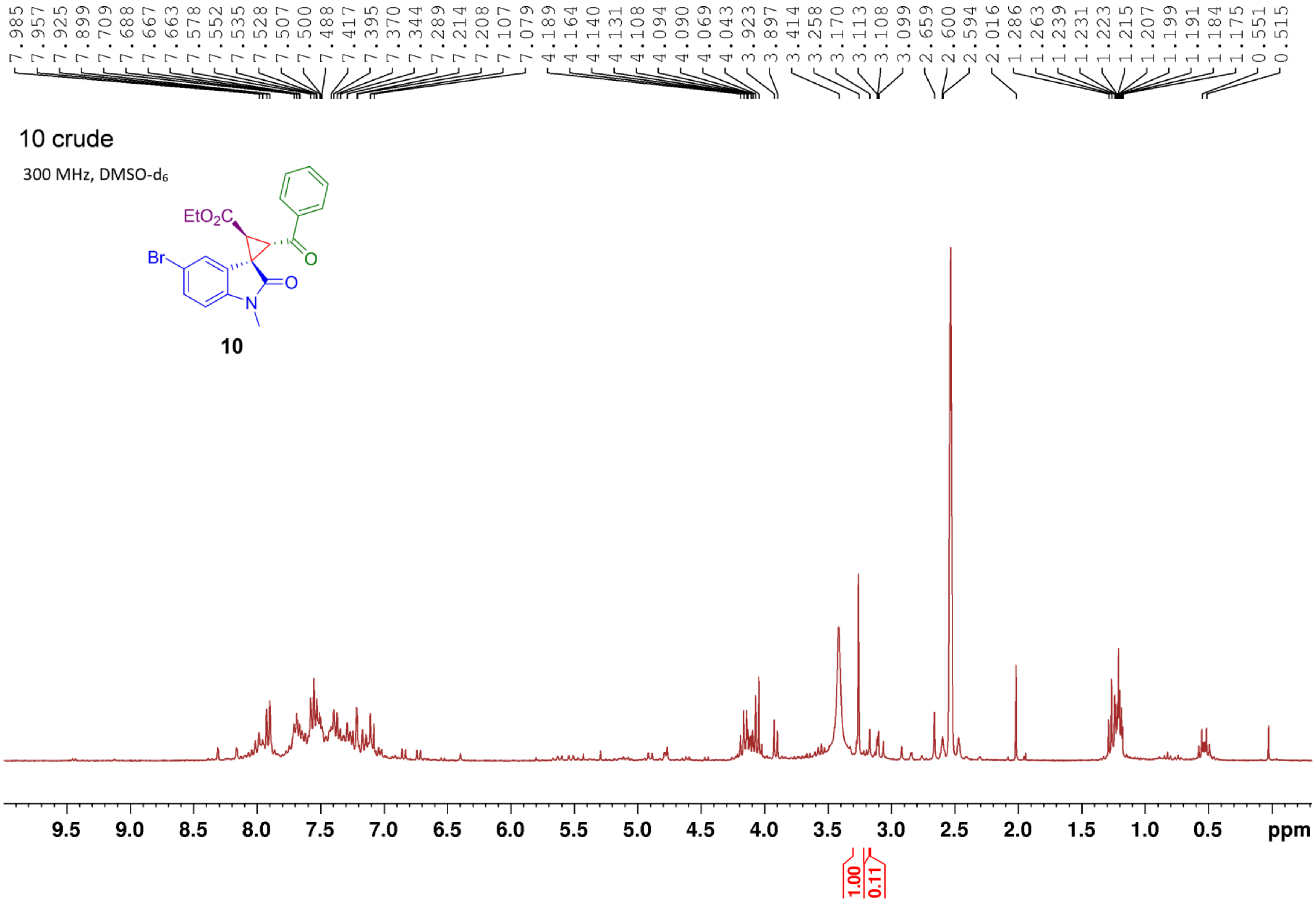

7.985
7.957
7.925
7.899
7.709
7.688
7.667
7.663
7.578
7.552
7.535
7.528
7.507
7.500
7.488
7.417
7.395
7.370
7.344
7.289
7.214
7.208
7.107
7.079
4.189
4.164
4.140
4.131
4.108
4.094
4.090
4.069
4.043
3.923
3.897
3.414
3.258
3.170
3.113
3.108
3.099
2.659
2.600
2.594
2.016
1.286
1.263
1.239
1.231
1.223
1.215
1.207
1.199
1.191
1.184
1.175
0.551
0.515
10 crude
300 MHz, DMSO-d6
EtO2C
Br
O
N
O
10
9.5
9.0
8.5
8.0
7.5
7.0
6.5
6.0
5.5
5.0
4.5
4.0
3.5
3.0
2.5
2.0
1.5
1.0
0.5
ppm
1.00
0.11

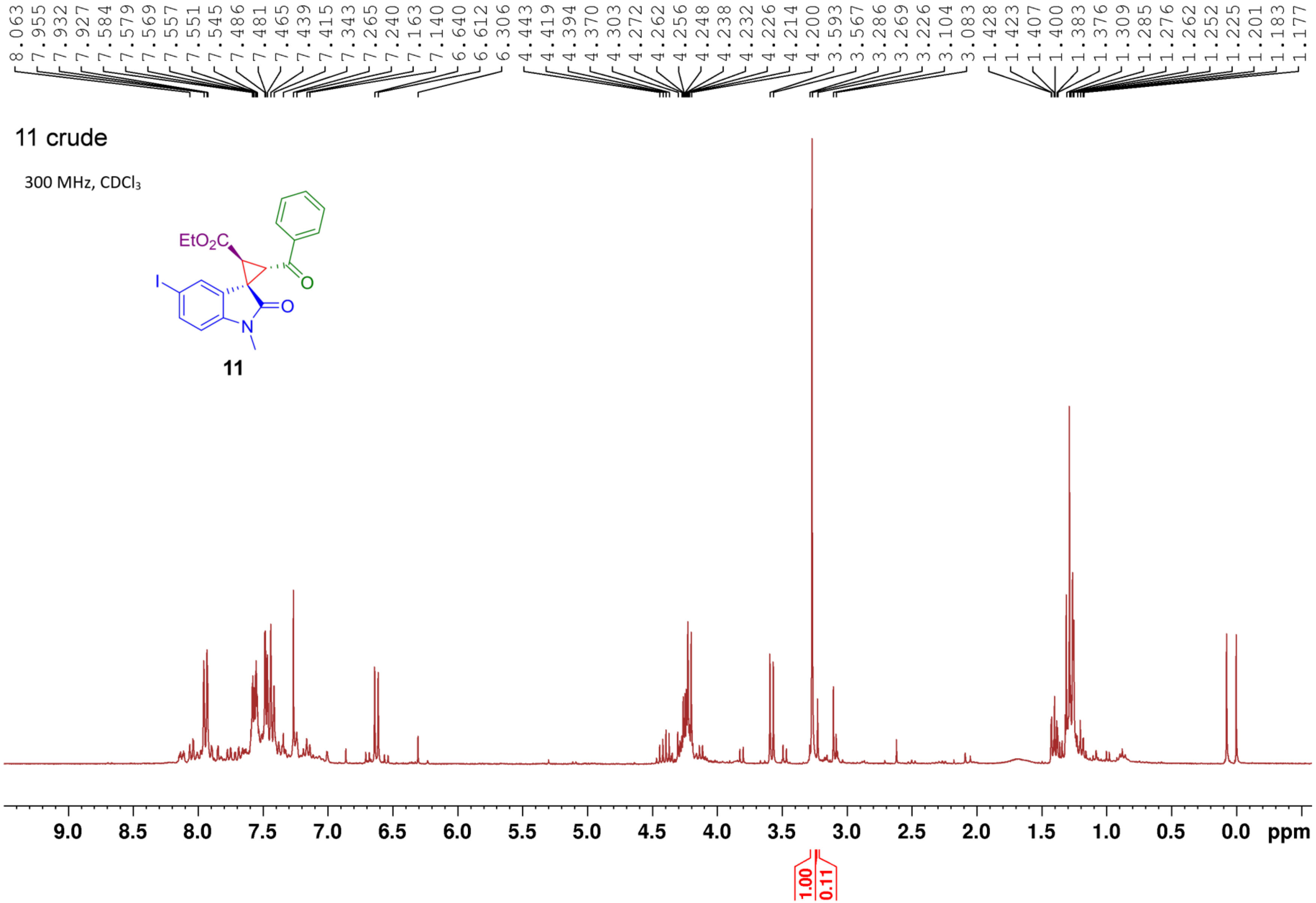

11 crude
300 MHz, CDCl3
EtO2C
I
O
N
11
8.063 7.955 7.932 7.927 7.584 7.579 7.569 7.557 7.551 7.545 7.486 7.481 7.465 7.439 7.415 7.343 7.265 7.240 7.163 7.140 6.640 6.612 6.306 4.443 4.419 4.394 4.370 4.303 4.272 4.262 4.256 4.248 4.238 4.232 4.226 4.214 4.200 3.593 3.567 3.286 3.269 3.226 3.104 3.083 1.428 1.423 1.407 1.400 1.383 1.376 1.309 1.285 1.276 1.262 1.252 1.225 1.201 1.183 1.177
9.0 8.5 8.0 7.5 7.0 6.5 6.0 5.5 5.0 4.5 4.0 3.5 3.0 2.5 2.0 1.5 1.0 0.5 0.0 ppm
1.00
0.11

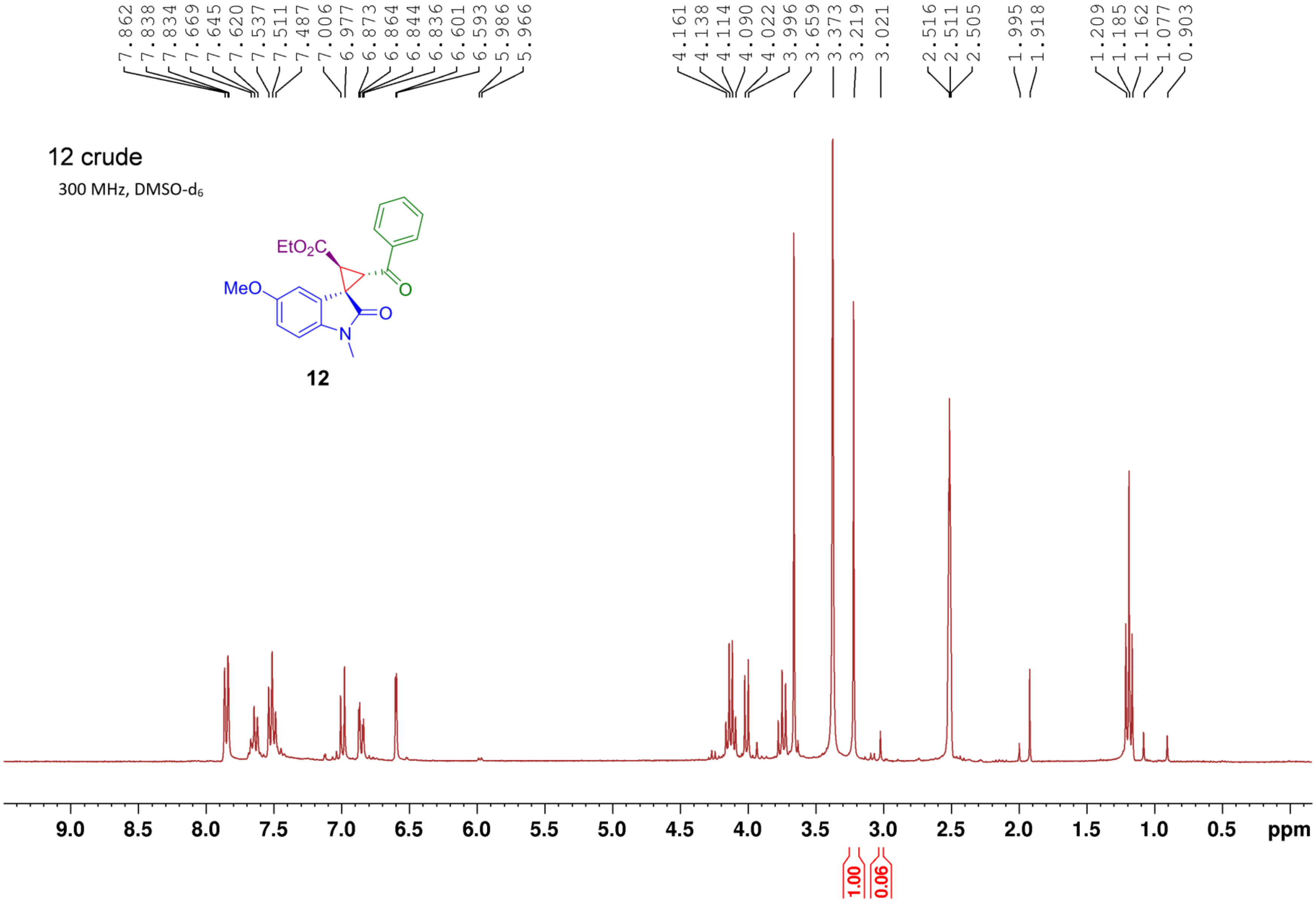

12 crude
300 MHz, DMSO-d6
EtO2C
MeO
O
O
N
12
7.862
7.838
7.834
7.669
7.645
7.620
7.537
7.511
7.487
7.006
6.977
6.873
6.864
6.844
6.836
6.601
6.593
5.986
5.966
4.161
4.138
4.114
4.090
4.022
3.996
3.659
3.373
3.219
3.021
2.516
2.511
2.505
1.995
1.918
1.209
1.185
1.162
1.077
0.903
9.0 8.5 8.0 7.5 7.0 6.5 6.0 5.5 5.0 4.5 4.0 3.5 3.0 2.5 2.0 1.5 1.0 0.5 ppm
1.00
0.06

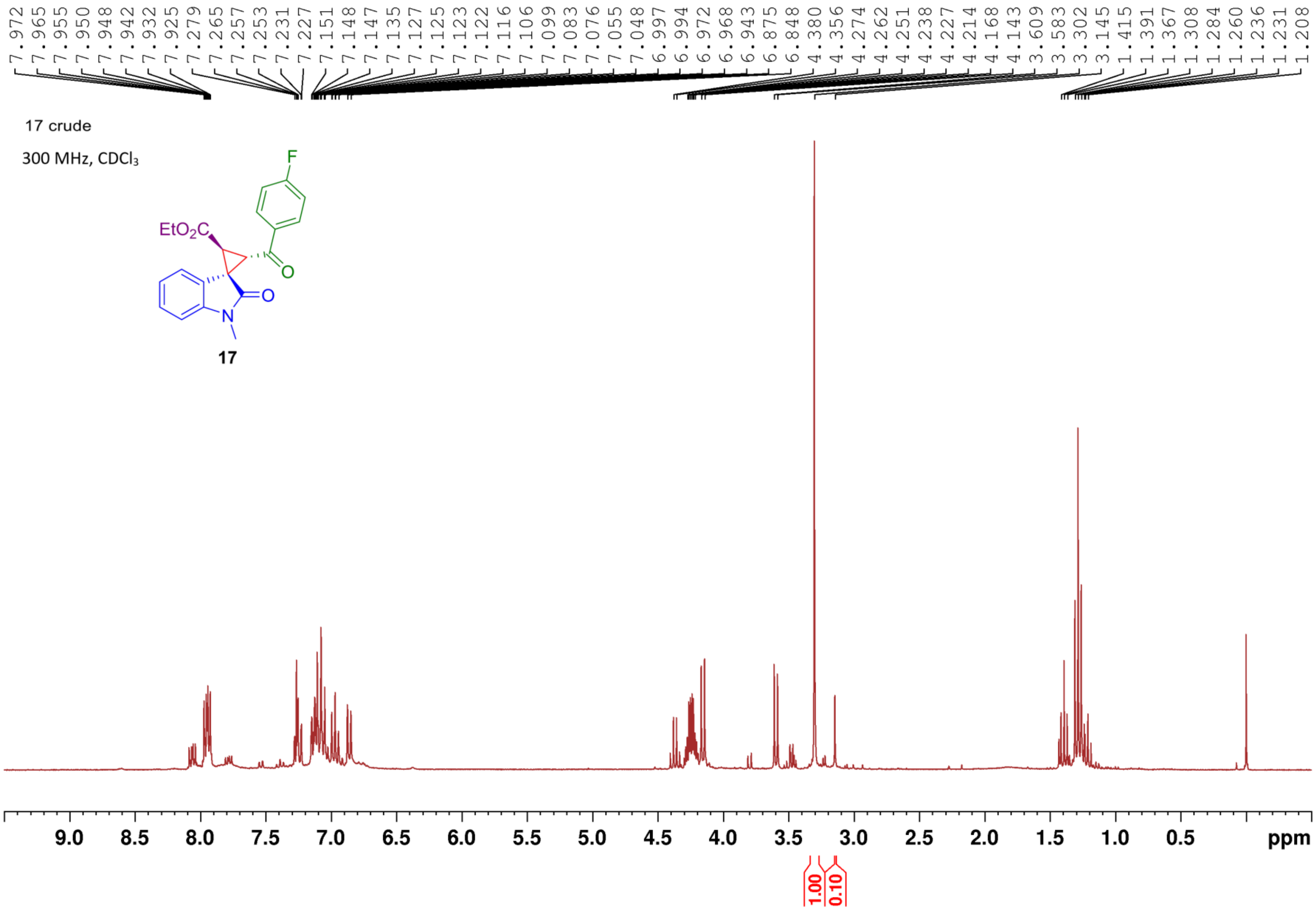
17 crude
300 MHz, CDCl3
EtO2C
F
O
O
N
17
7.972 7.965 7.955 7.950 7.948 7.942 7.932 7.925 7.279 7.265 7.257 7.253 7.231 7.227 7.151 7.148 7.147 7.135 7.127 7.125 7.123 7.122 7.116 7.106 7.099 7.083 7.076 7.055 7.048 6.997 6.994 6.972 6.968 6.943 6.875 6.848 4.380 4.356 4.274 4.262 4.251 4.238 4.227 4.214 4.168 4.143 3.609 3.583 3.302 3.145 1.415 1.391 1.367 1.308 1.284 1.260 1.236 1.231 1.208
9.0 8.5 8.0 7.5 7.0 6.5 6.0 5.5 5.0 4.5 4.0 3.5 3.0 2.5 2.0 1.5 1.0 0.5 ppm
1.00
0.10

18 crude

300 MHz, DMSO-d_6

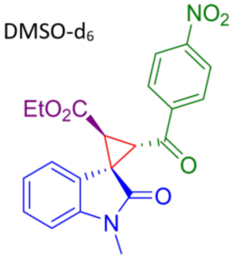

18

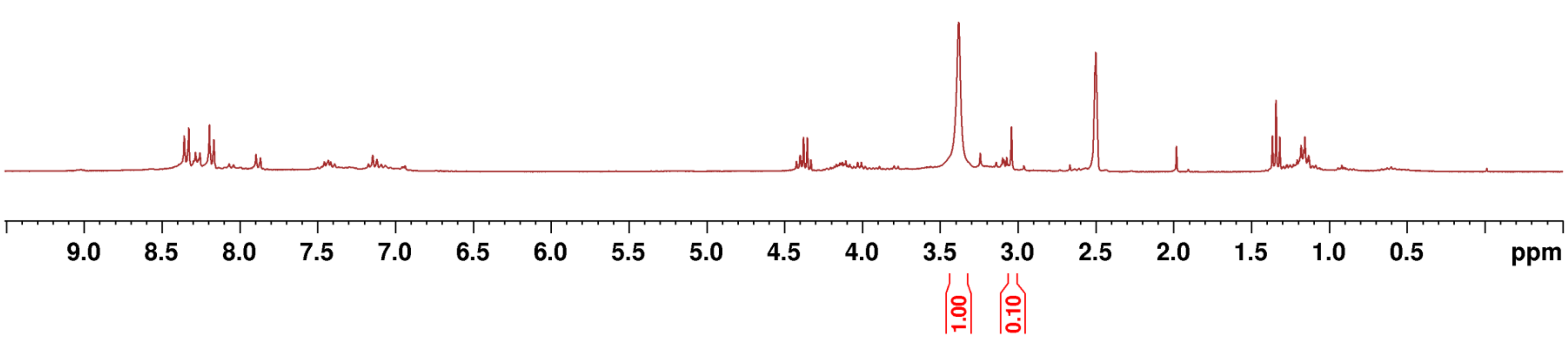

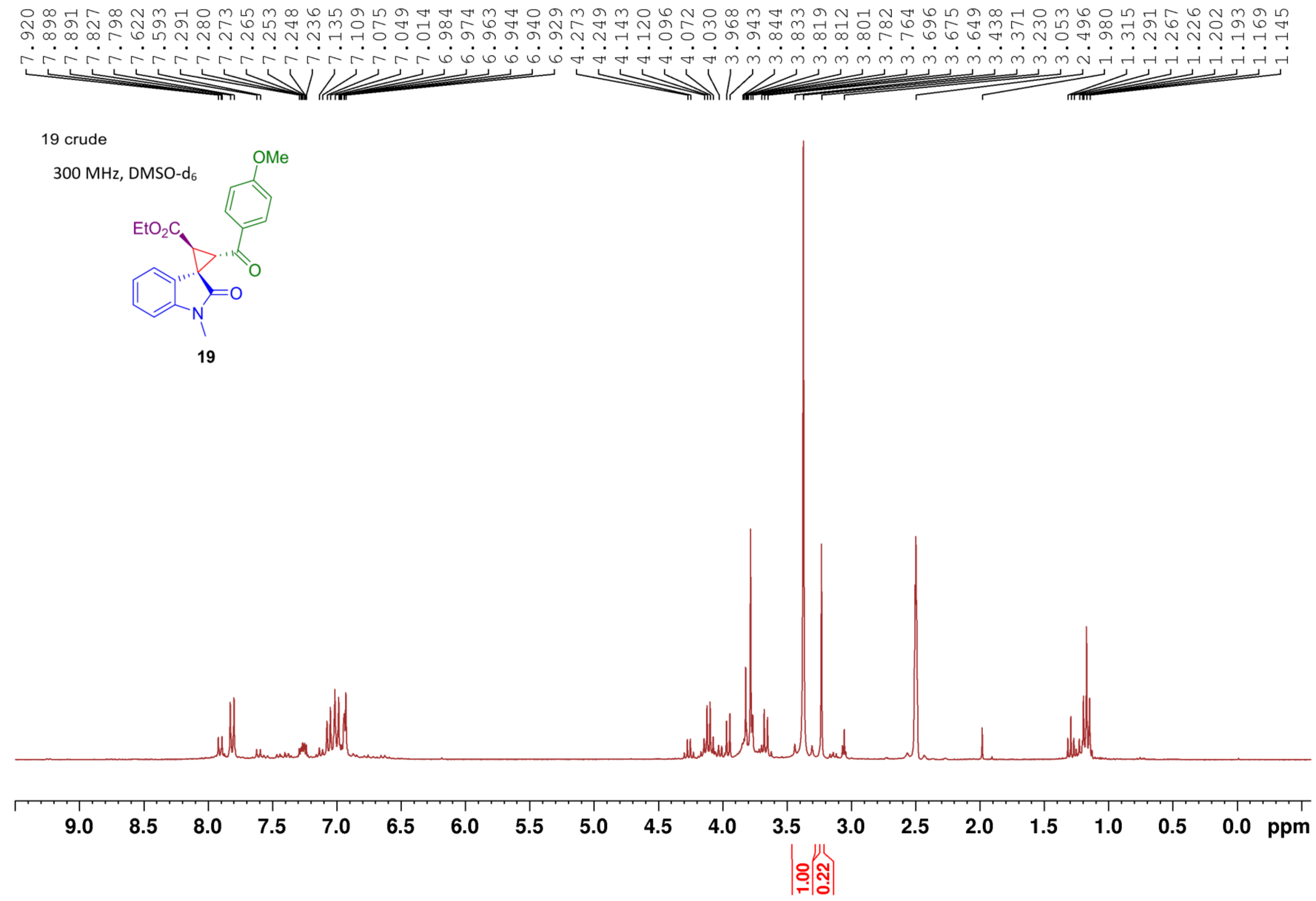
7.920 7.898 7.891 7.827 7.798 7.622 7.593 7.291 7.280 7.273 7.265 7.253 7.248 7.236 7.135 7.109 7.075 7.049 7.014 6.984 6.974 6.963 6.944 6.940 6.929 4.273 4.249 4.143 4.120 4.096 4.072 4.030 3.968 3.943 3.844 3.833 3.819 3.812 3.801 3.782 3.764 3.696 3.675 3.649 3.438 3.371 3.230 3.053 2.496 1.980 1.315 1.291 1.267 1.226 1.202 1.193 1.169 1.145
19 crude
300 MHz, DMSO-d6
OMe
EtO2C
O
O
N
19
1.00
0.22
9.0 8.5 8.0 7.5 7.0 6.5 6.0 5.5 5.0 4.5 4.0 3.5 3.0 2.5 2.0 1.5 1.0 0.5 0.0 ppm

20 crude

300 MHz, DMSO-d$_6$

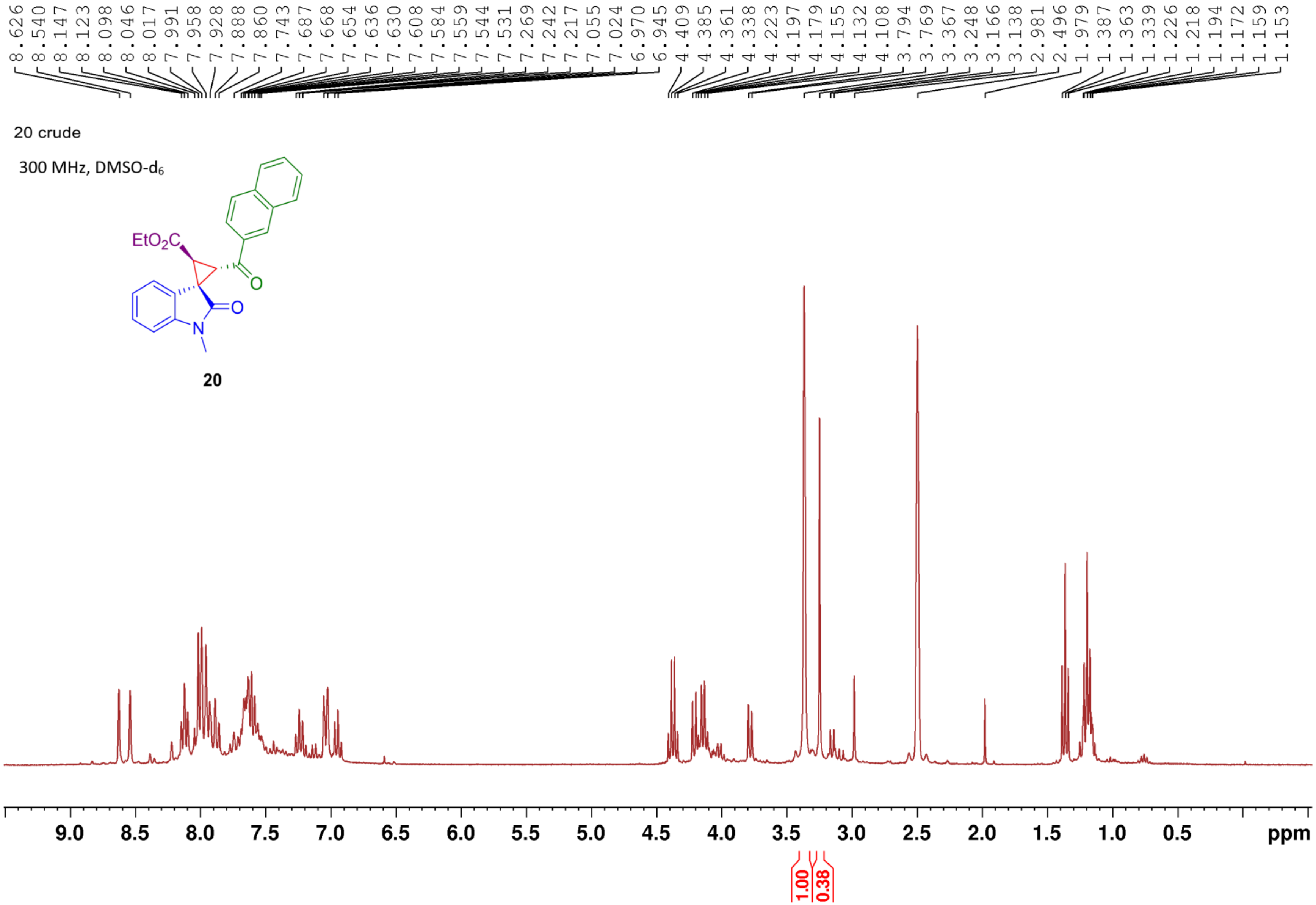

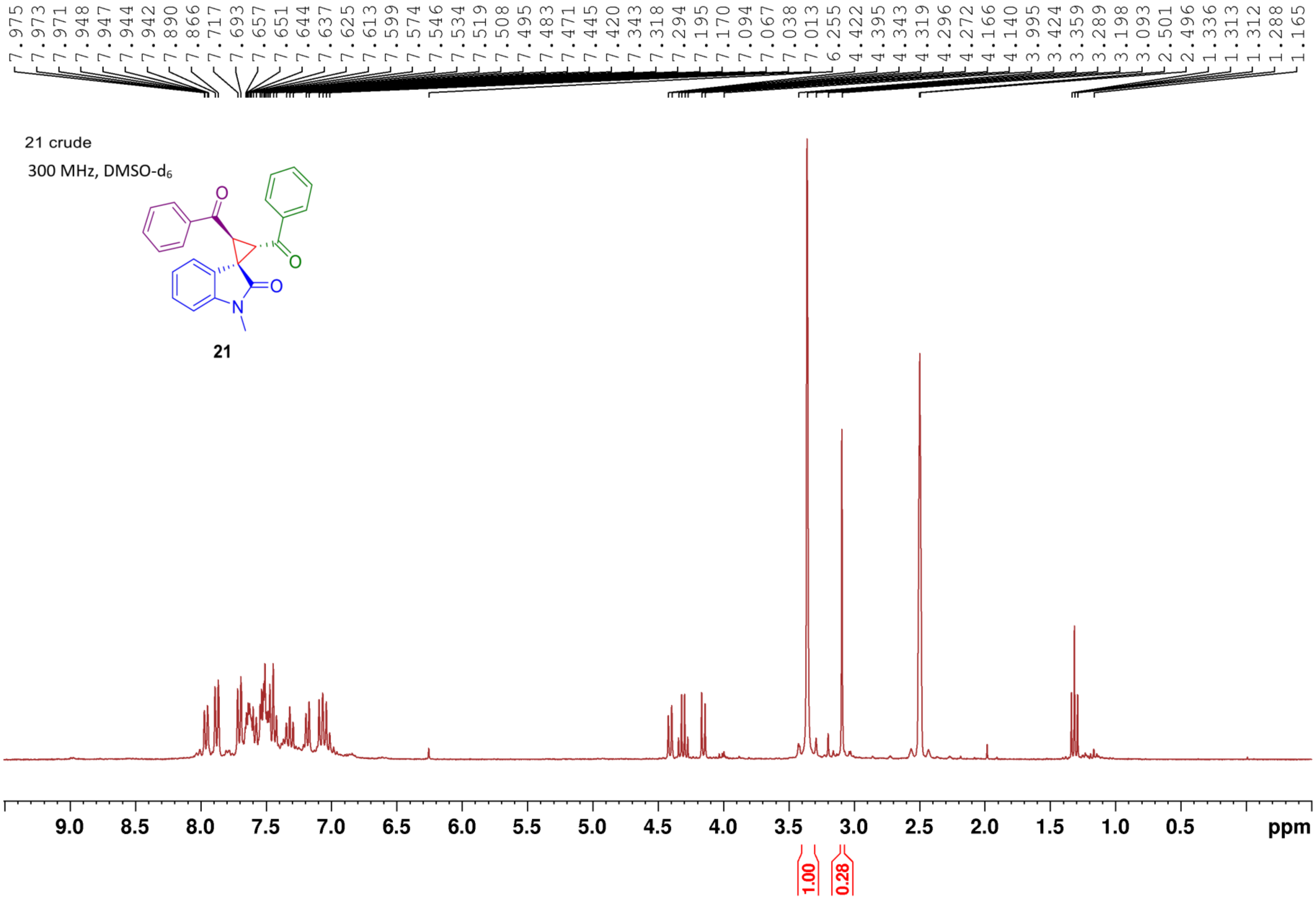
7.975
7.973
7.971
7.948
7.947
7.944
7.942
7.890
7.866
7.717
7.693
7.657
7.651
7.644
7.637
7.625
7.613
7.599
7.574
7.546
7.534
7.519
7.508
7.495
7.483
7.471
7.445
7.420
7.343
7.318
7.294
7.195
7.170
7.094
7.067
7.038
7.013
6.255
4.422
4.395
4.343
4.319
4.296
4.272
4.166
4.140
3.995
3.424
3.359
3.289
3.198
3.093
2.501
2.496
1.336
1.313
1.312
1.288
1.165
21 crude
300 MHz, DMSO-d6
O
O
N
O
21
9.0
8.5
8.0
7.5
7.0
6.5
6.0
5.5
5.0
4.5
4.0
3.5
3.0
2.5
2.0
1.5
1.0
0.5
ppm
1.00
0.28

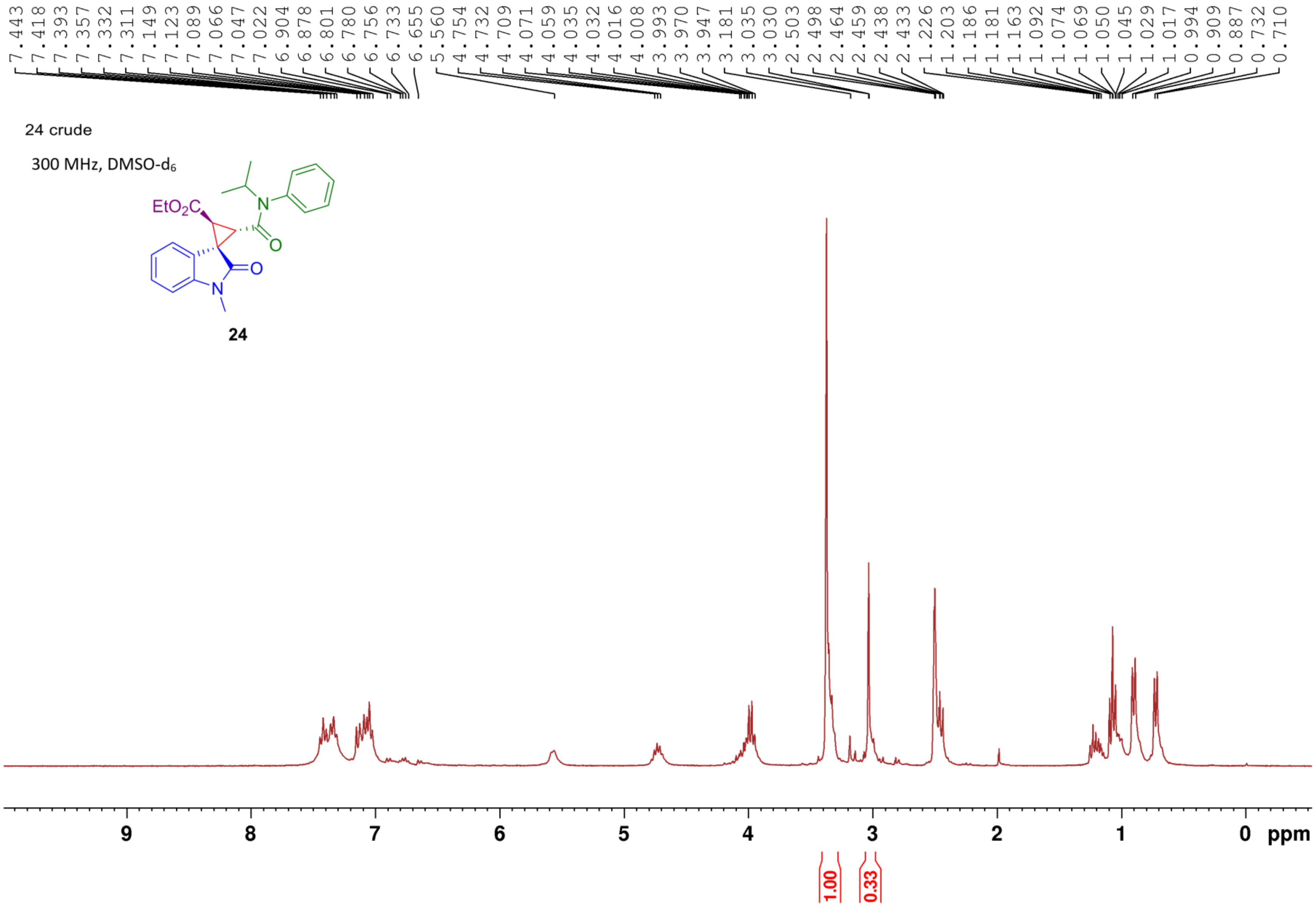
7.443 7.418 7.393 7.357 7.332 7.311 7.149 7.123 7.089 7.066 7.047 7.022 6.904 6.878 6.801 6.780 6.756 6.733 6.655 5.560 4.754 4.732 4.709 4.071 4.059 4.035 4.032 4.016 4.008 3.993 3.970 3.947 3.181 3.035 3.030 2.503 2.498 2.464 2.459 2.438 2.433 1.226 1.203 1.186 1.181 1.163 1.092 1.074 1.069 1.050 1.045 1.029 1.017 0.994 0.909 0.887 0.732 0.710
24 crude
300 MHz, DMSO-d6
EtO2C
N
O
O
N
24
9 8 7 6 5 4 3 2 1 0 ppm
1.00
0.33

10. ^{1}H, ^{13}C, NOE, and NOESY NMR spectra of the isolated products

The relative disposition of the substituents at the positions 2 and 3 of the cyclopropane ring was determined by NOE based NMR experiments (2D-NOESY or 1D-Selective NOE). All the samples displayed a relative *trans* orientation of the substituents in positions 2 and 3 of the cyclopropyl ring, which are always on opposite faces. The $^{3}J_{H2-H3}$ vicinal coupling constants were in the range of 7.7-7.8 Hz for all the compounds. It could be established that there are 2 types of *trans* compounds depending on the relative orientation of the protons H3 and H2 with respect to the aromatic proton H4' of the indole: A type, with H4' and H3 on the same face and B type, with H4' and H2 on the same face.

The configuration of the spiranic carbon cannot be deduced by NMR. An arbitrary (*R*) absolute configuration has been used throughout the text.

Compounds exhibited highly homologous ^{1}H-NMR spectra and the key protons H3, H2, Me (1') and H2/6 of COPh were easily identified. The H7' protons were identified by the NOE Me(1')-H7', except when the 7' position is substituted.

All the samples, except **5b**, have the A type orientation, with H4' and H3 on the same face. Compound **5b** has the opposite B type orientation.

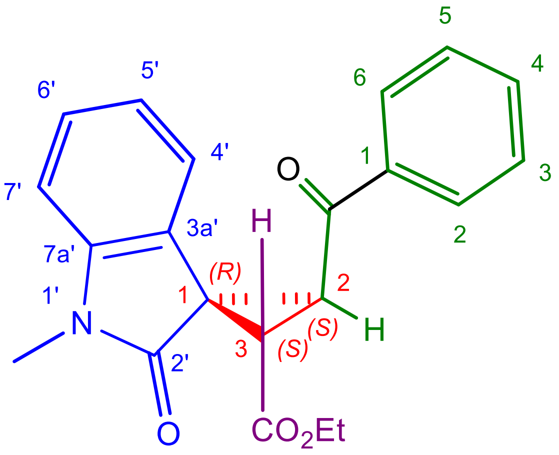

5a - 1H NMR, 400 MHz

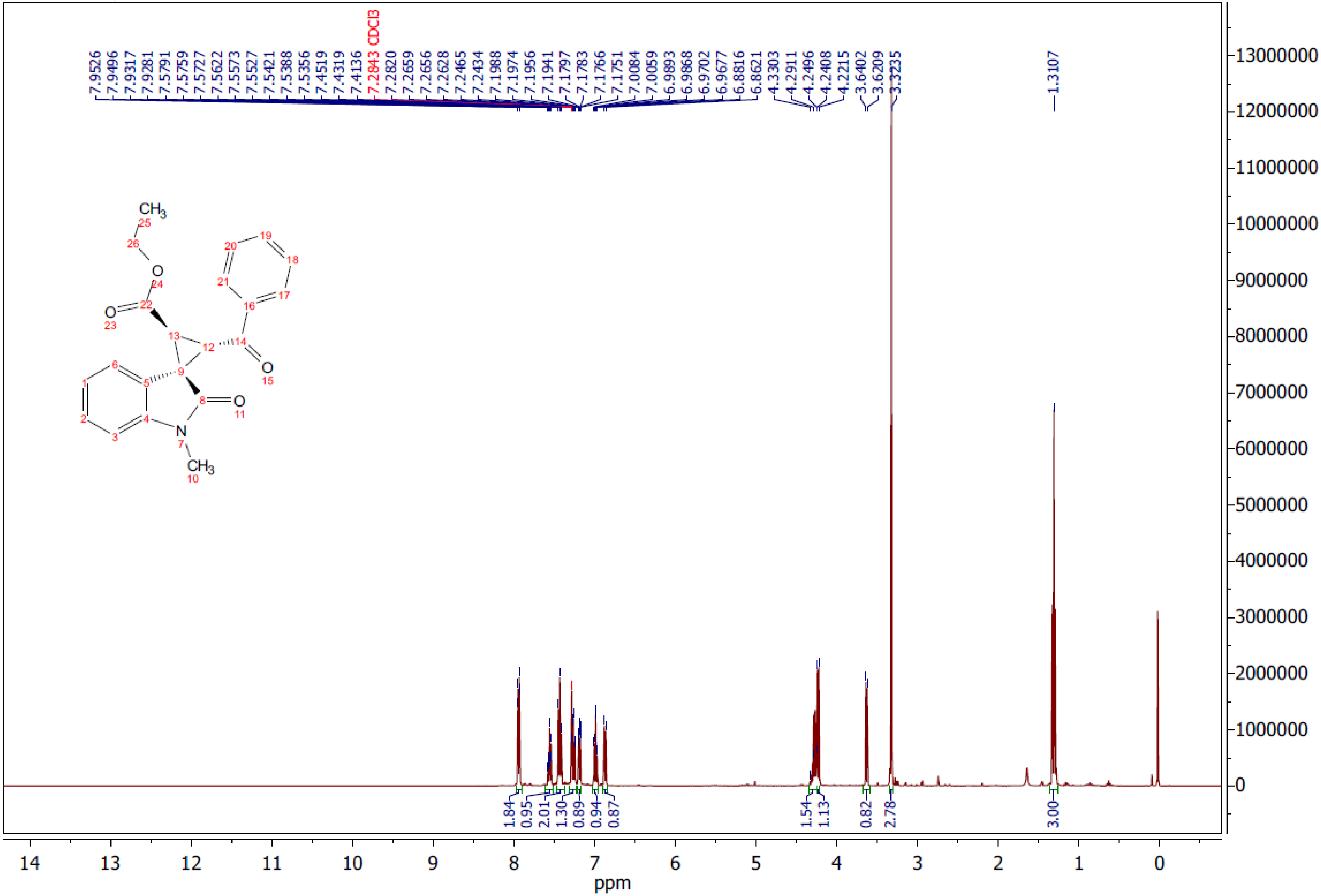

5a - 13C NMR, 126 MHz

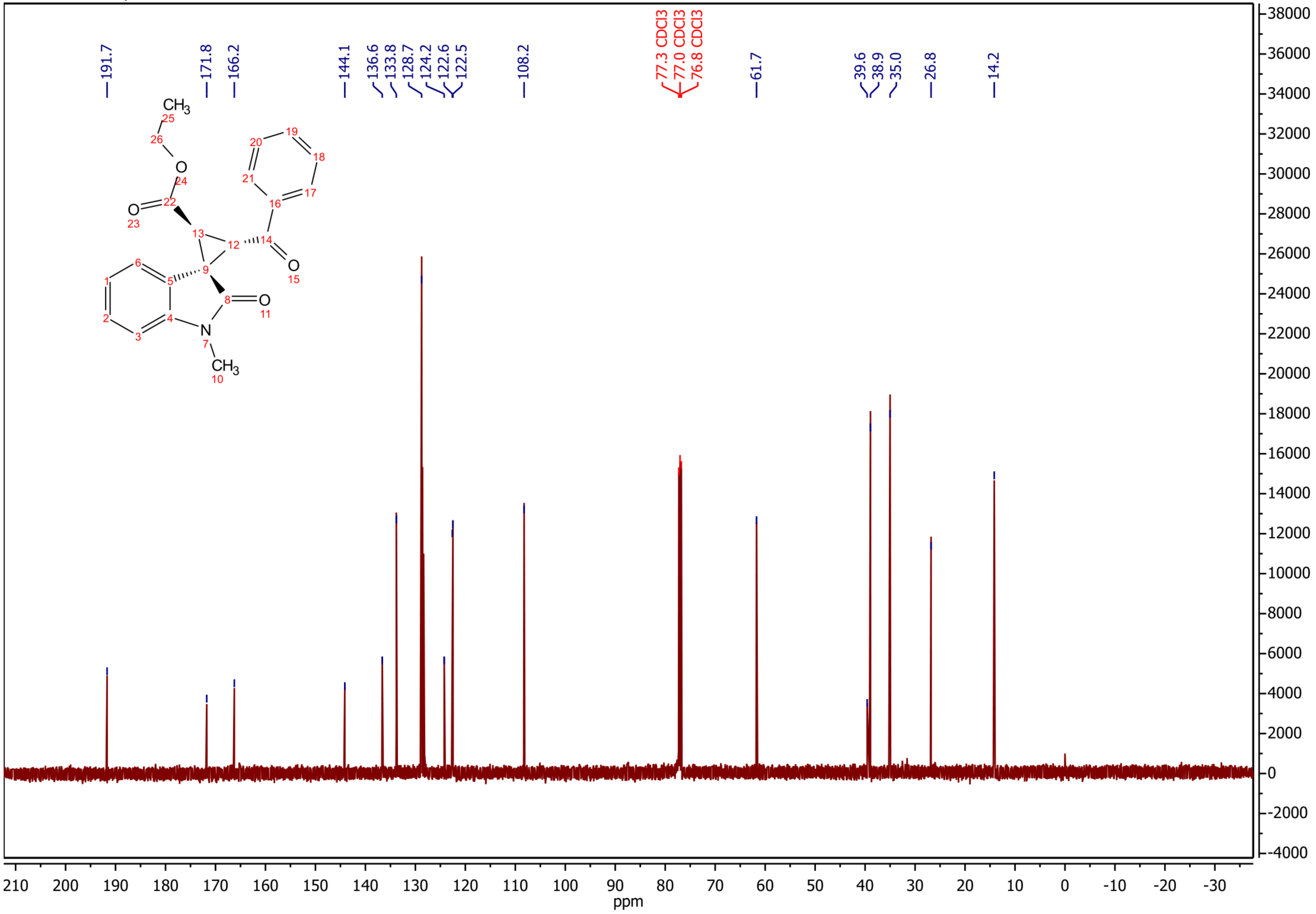

2D-NOESY

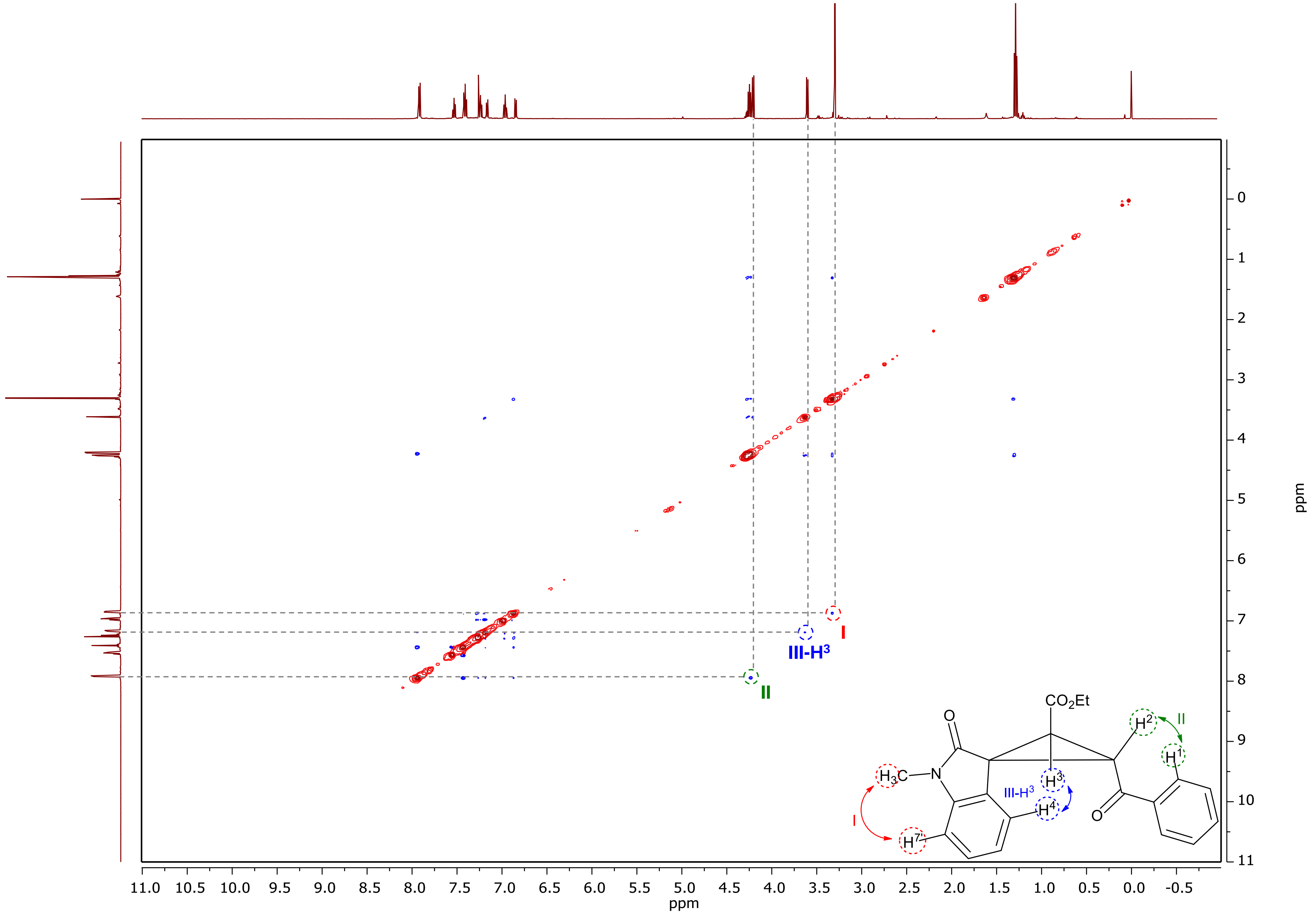

ppm
0
1
2
3
4
5
6
7
8
9
10
11
11.0 10.5 10.0 9.5 9.0 8.5 8.0 7.5 7.0 6.5 6.0 5.5 5.0 4.5 4.0 3.5 3.0 2.5 2.0 1.5 1.0 0.5 0.0 -0.5
ppm
I
III-H3
II
CO2Et
O
H3C
N
H2
H1
H3
III-H3
H4
O
I
H7
II

1D-Selective NOE experiments

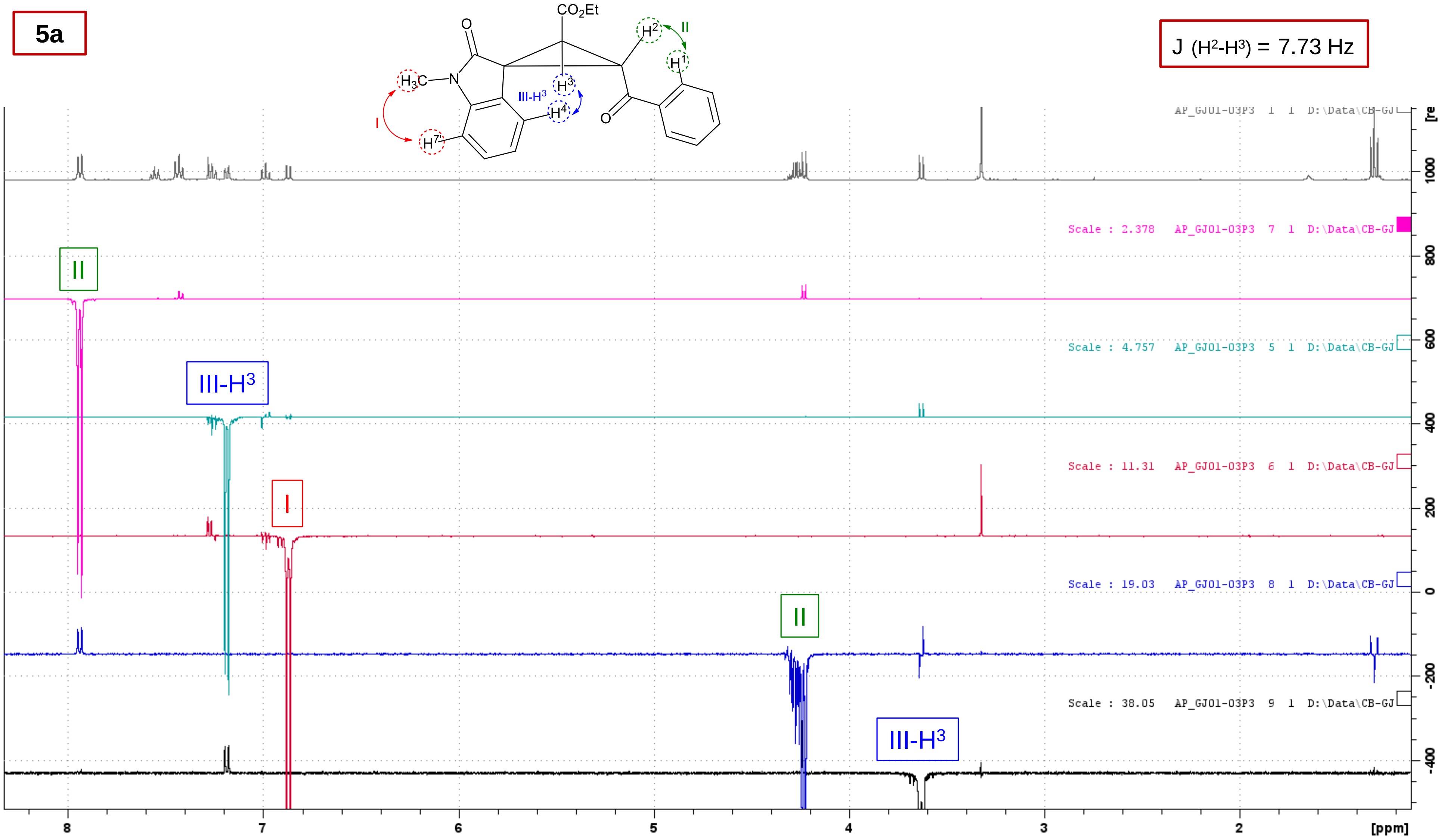

5b - 1H NMR, 400 MHz

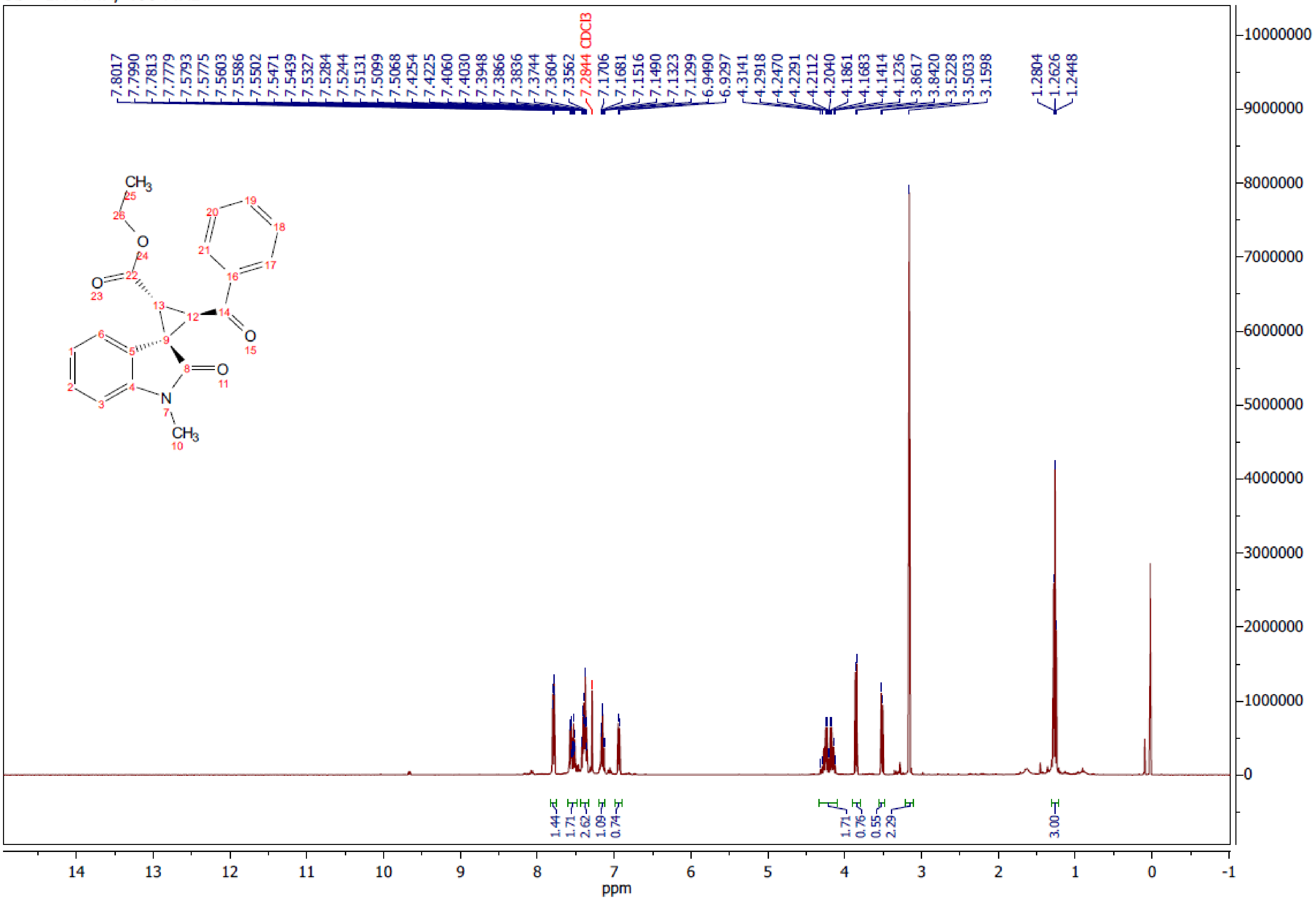

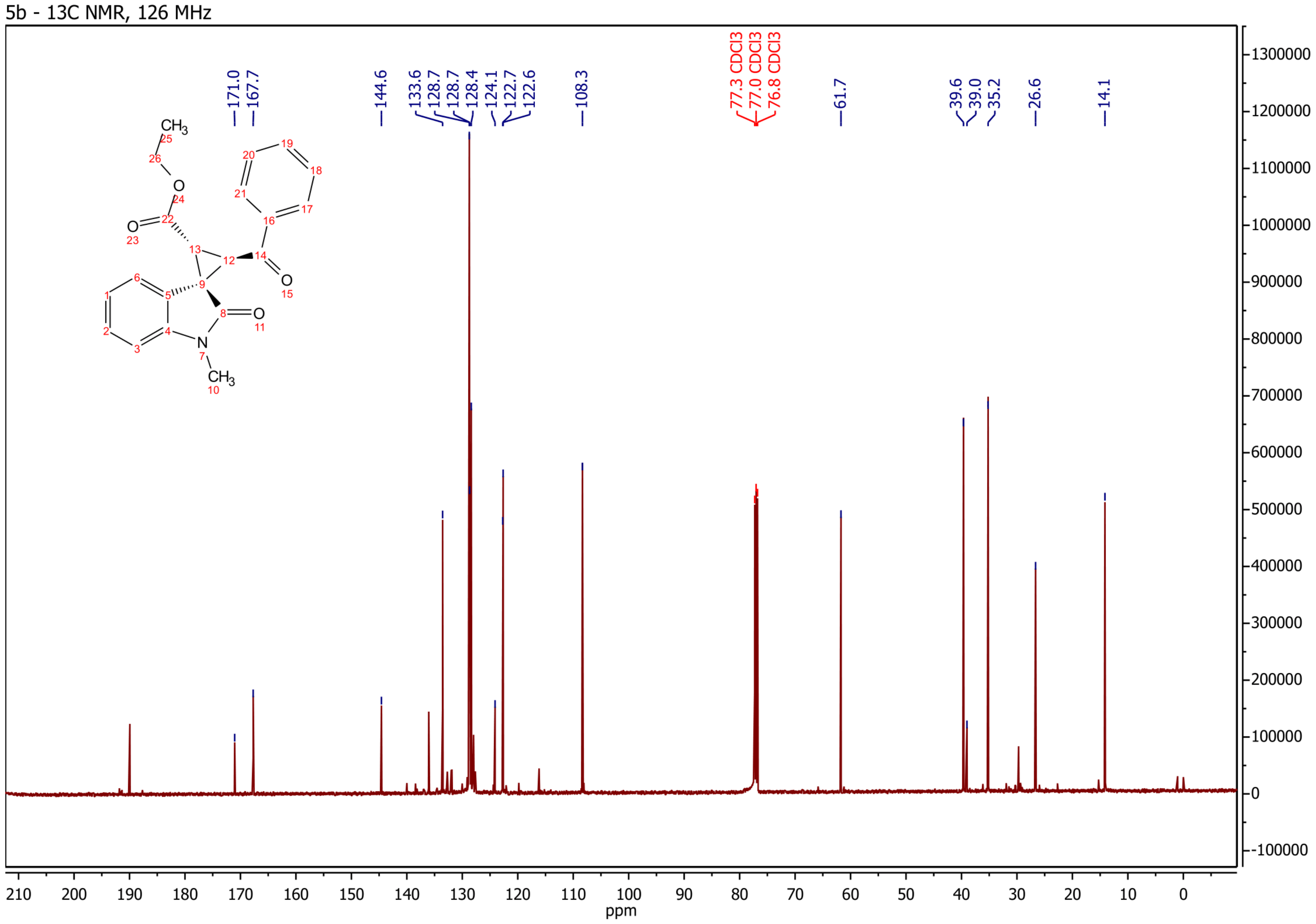
5b - 13C NMR, 126 MHz
171.0
167.7
144.6
133.6
128.7
128.7
128.4
124.1
122.7
122.6
108.3
77.3 CDCl3
77.0 CDCl3
76.8 CDCl3
61.7
39.6
39.0
35.2
26.6
14.1
ppm

2D-NOESY

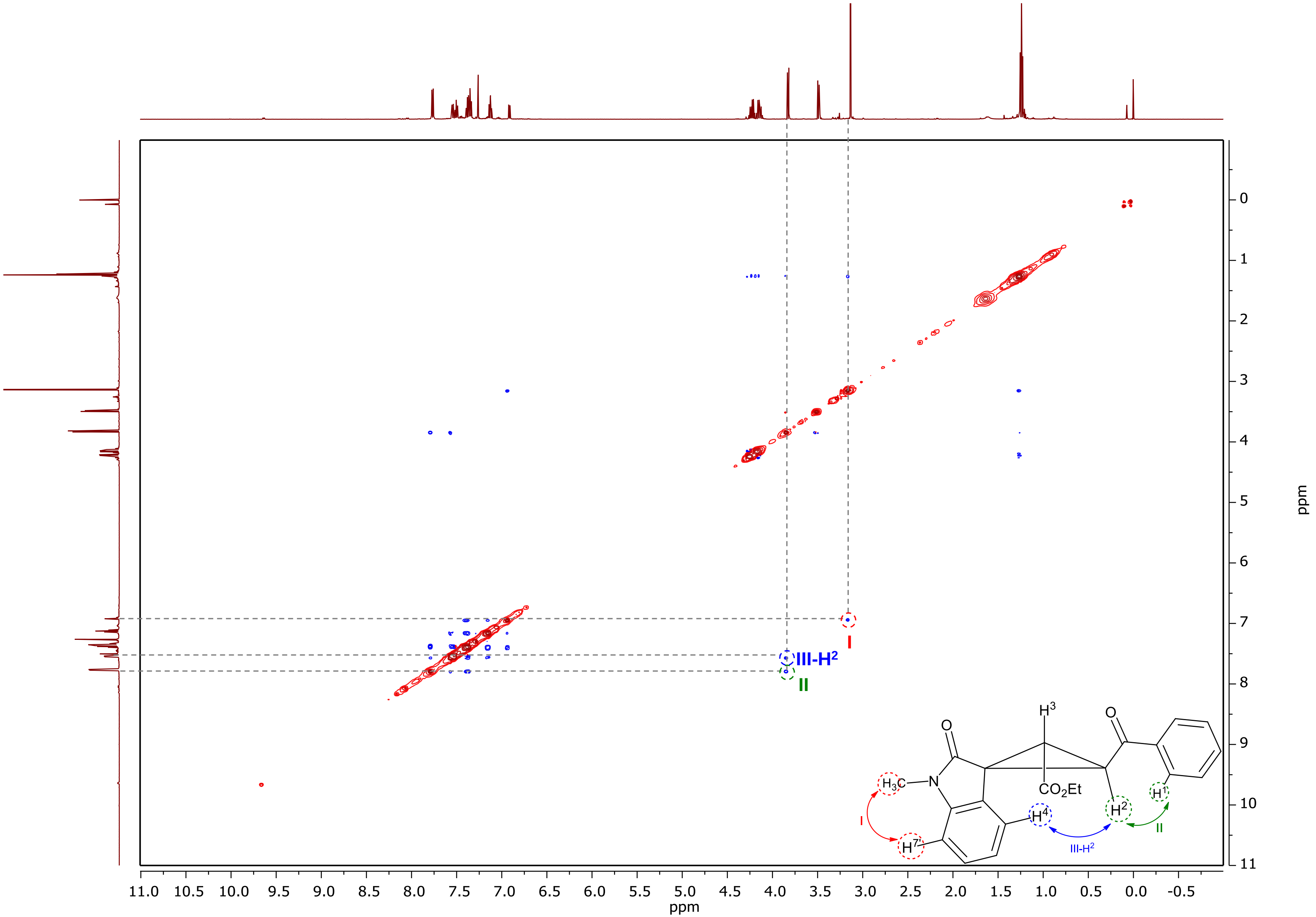

I
III-H2
II
H3
O
O
H3C
N
CO2Et
H1
H2
H4
H7
I
III-H2
II
ppm
11.0 10.5 10.0 9.5 9.0 8.5 8.0 7.5 7.0 6.5 6.0 5.5 5.0 4.5 4.0 3.5 3.0 2.5 2.0 1.5 1.0 0.5 0.0 -0.5
ppm
0 1 2 3 4 5 6 7 8 9 10 11

1D-Selective NOE experiments

5b

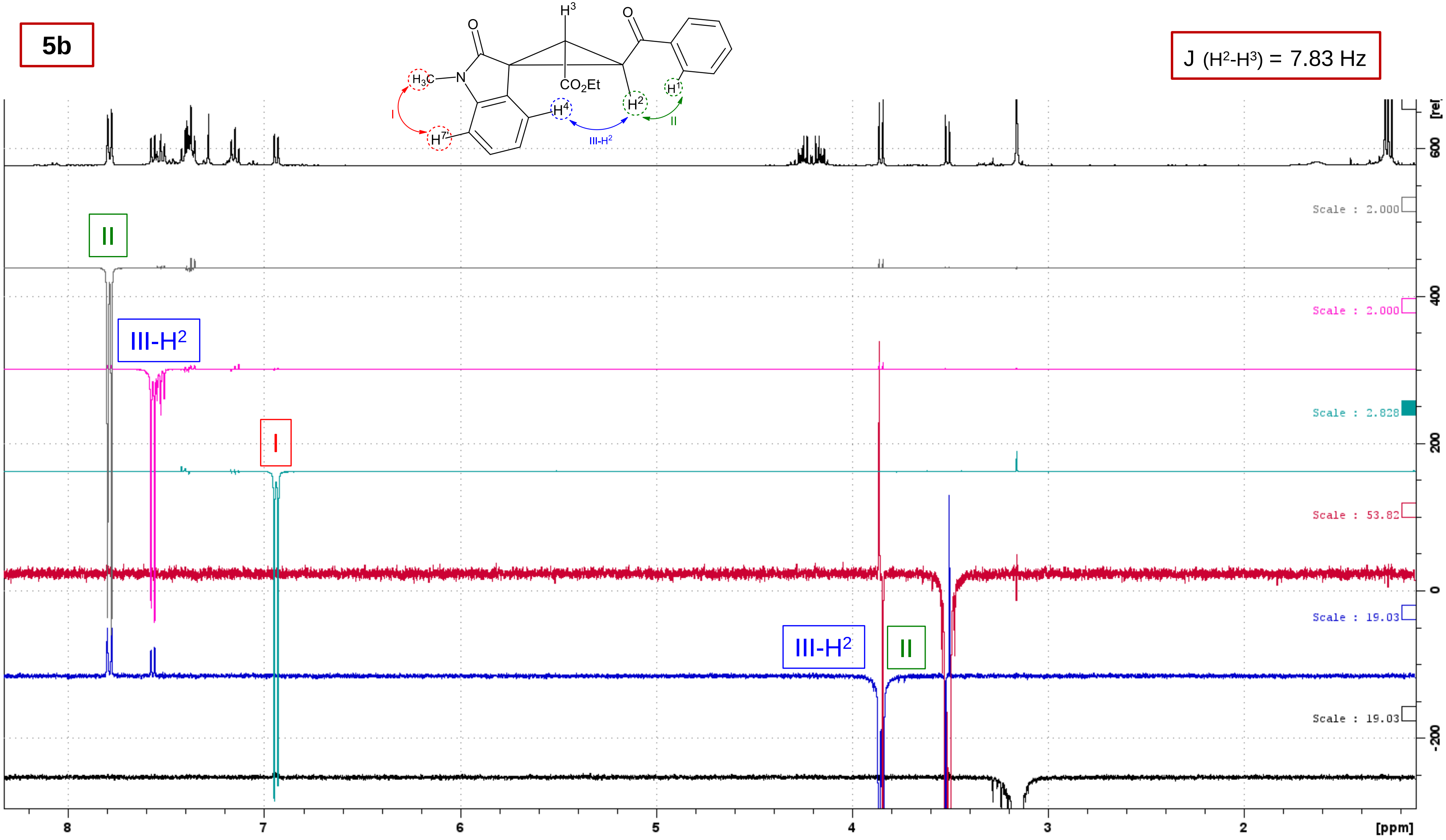

6 - 1H NMR, 400 MHz

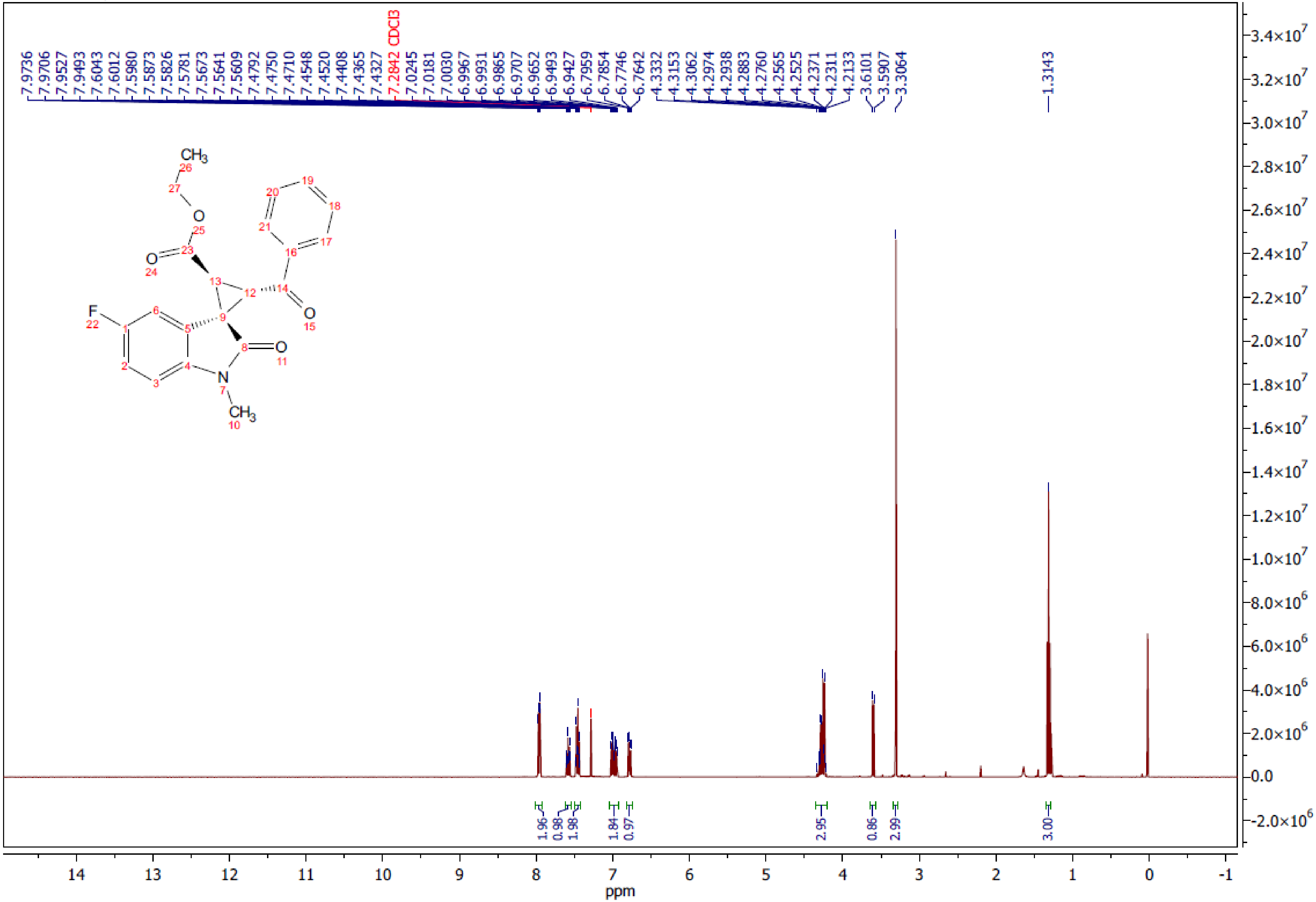

6 - 13C NMR, 126 MHz

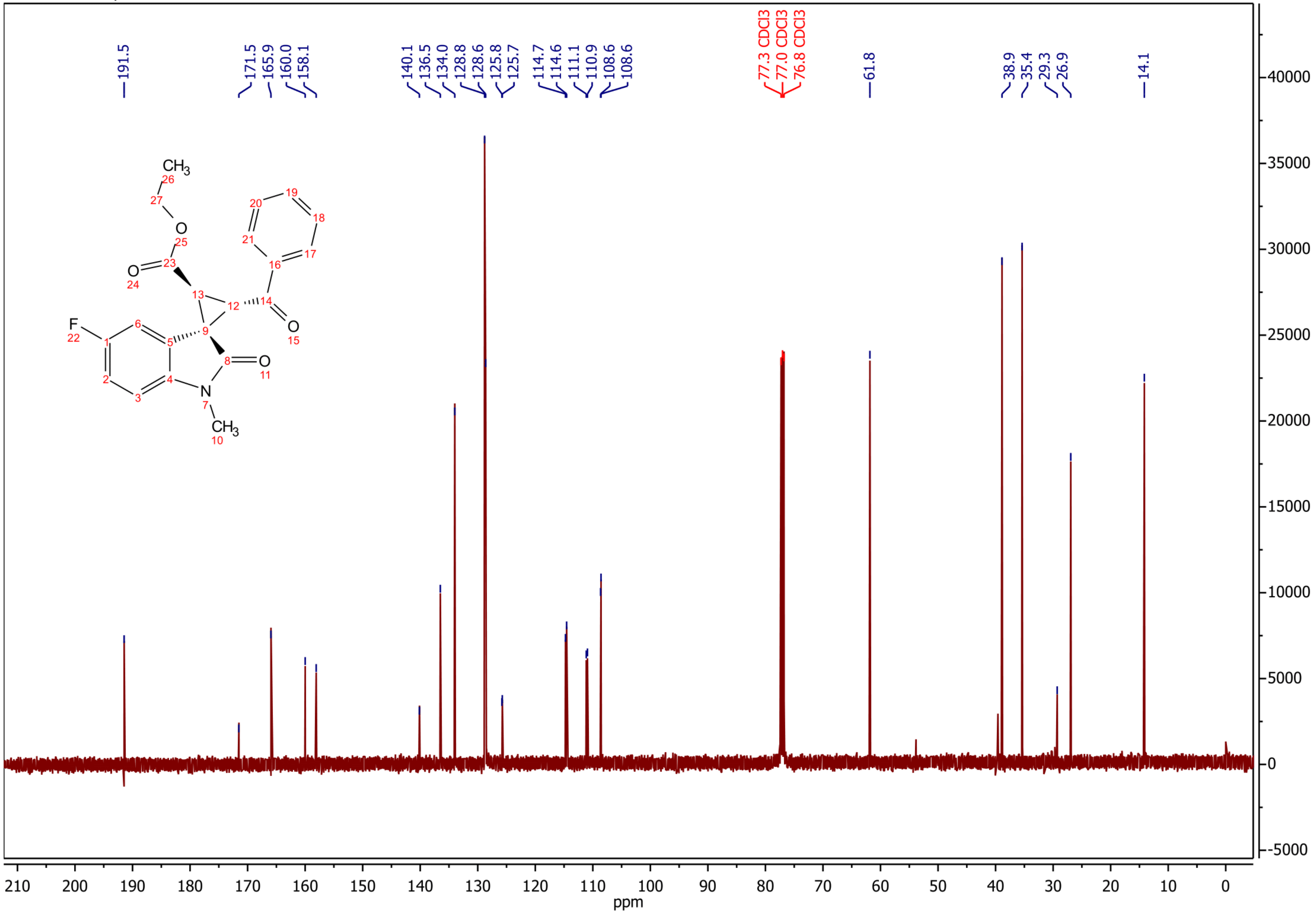

1D-Selective NOE experiments

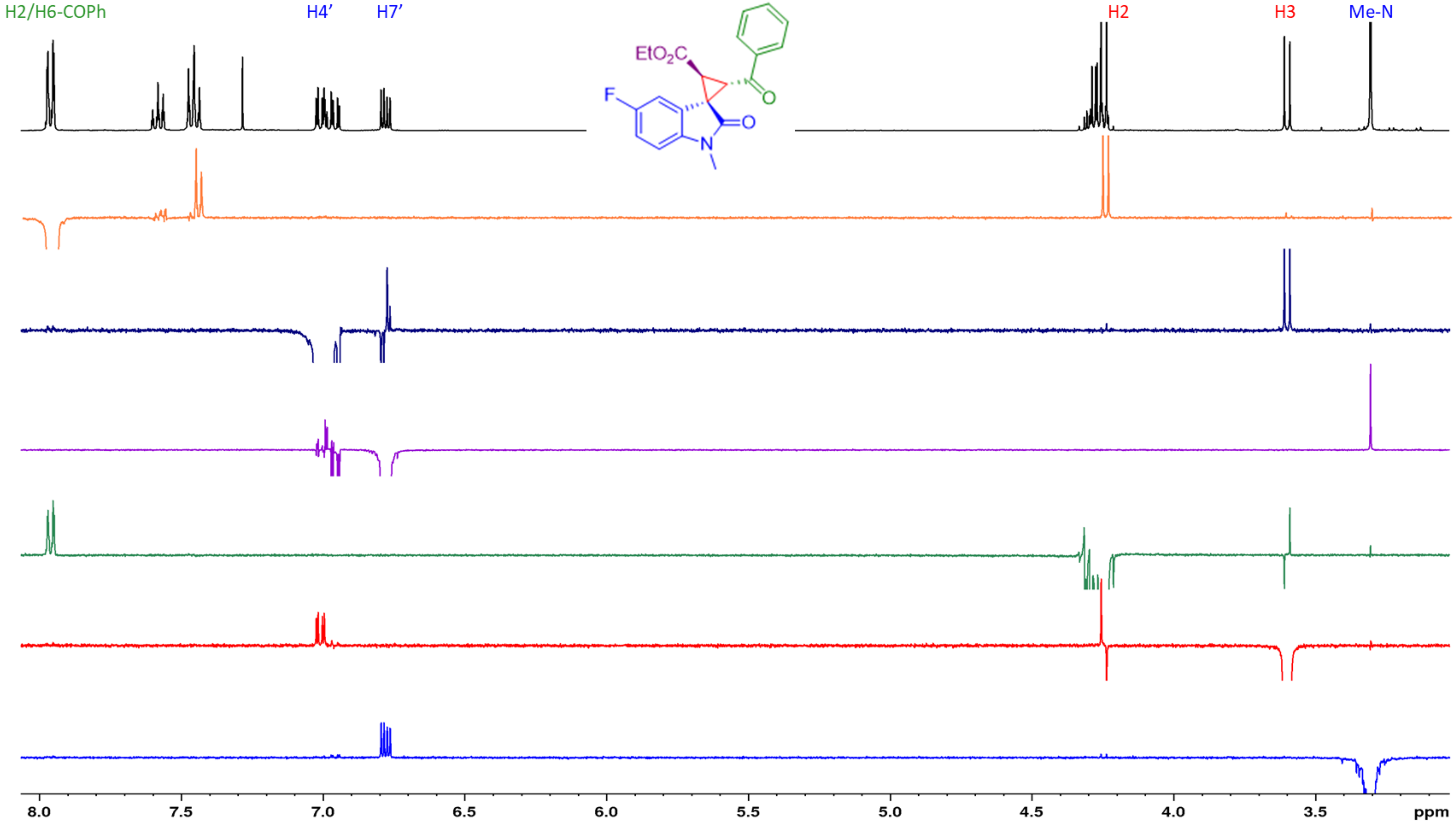

7 - 1H NMR, 400 MHz

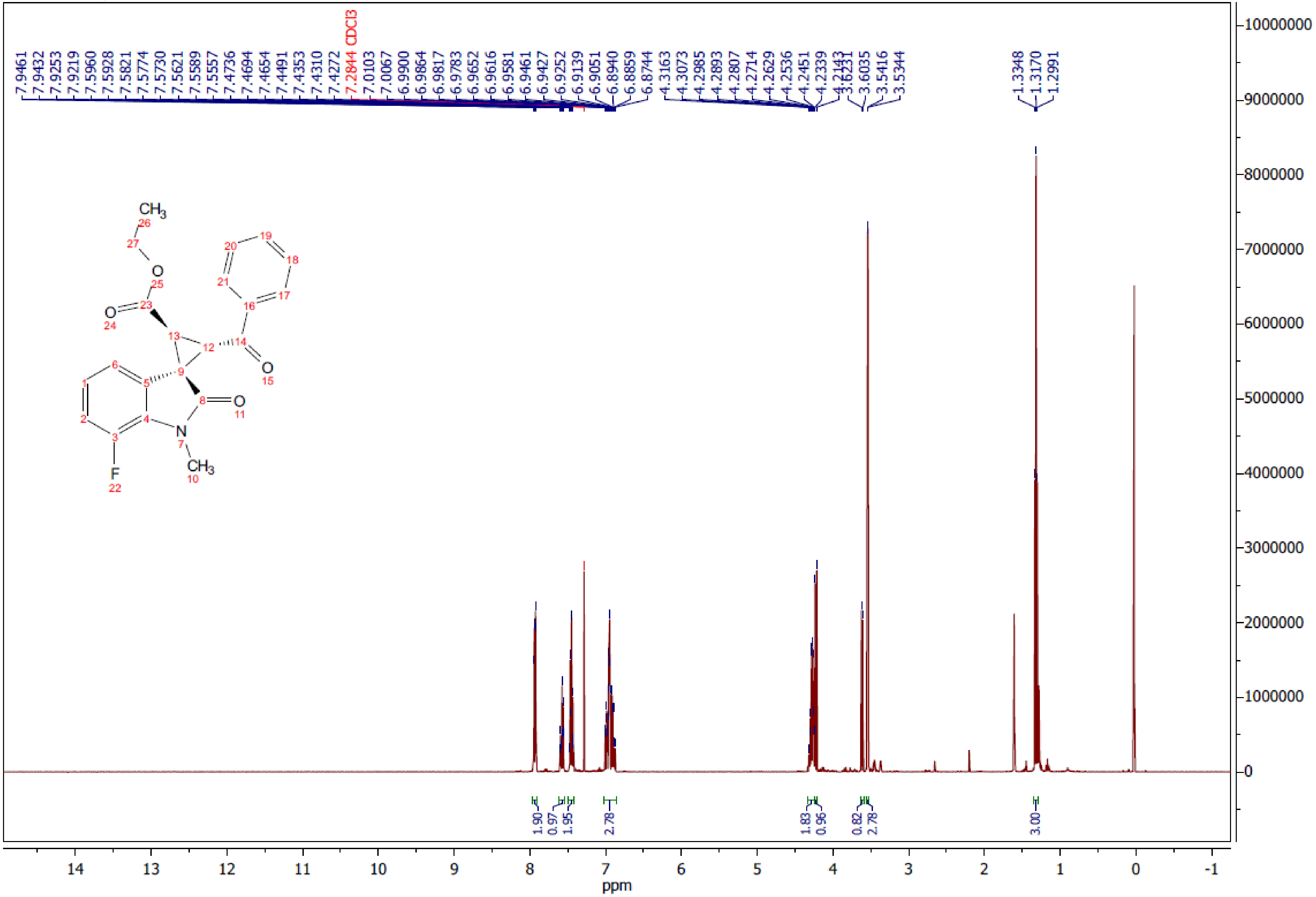

7 - 13C NMR, 126 MHz

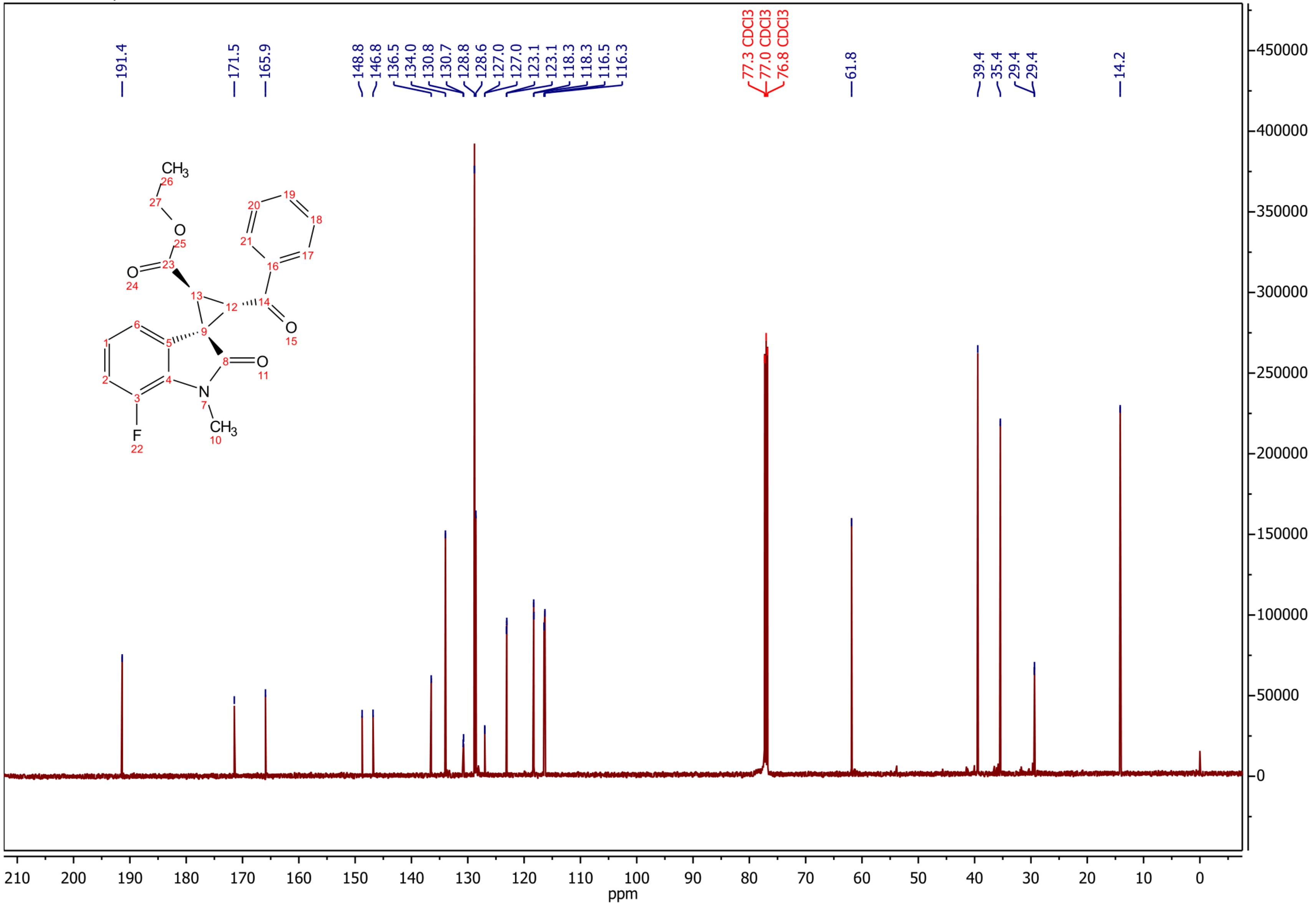

1D-Selective NOE experiments

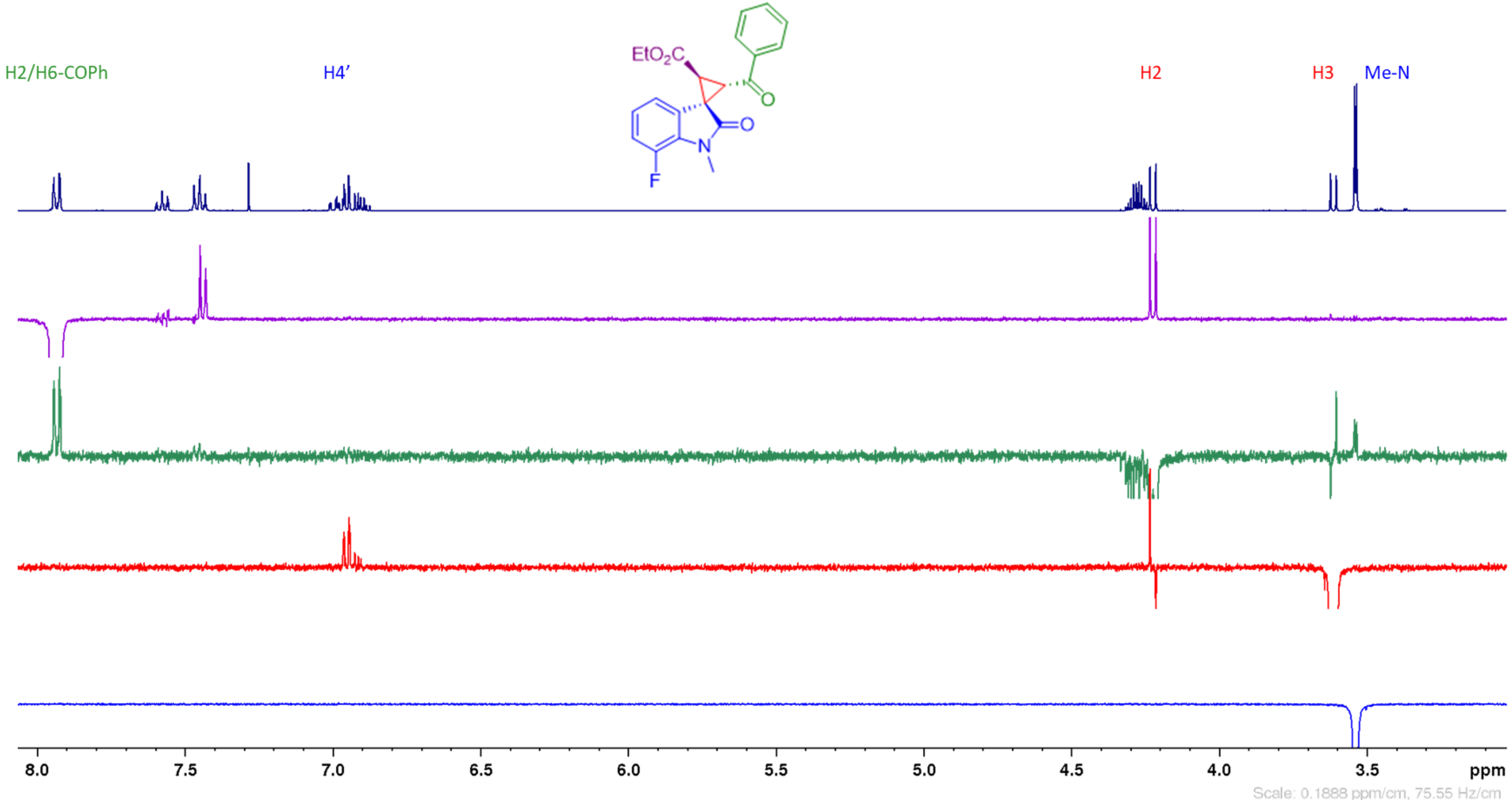

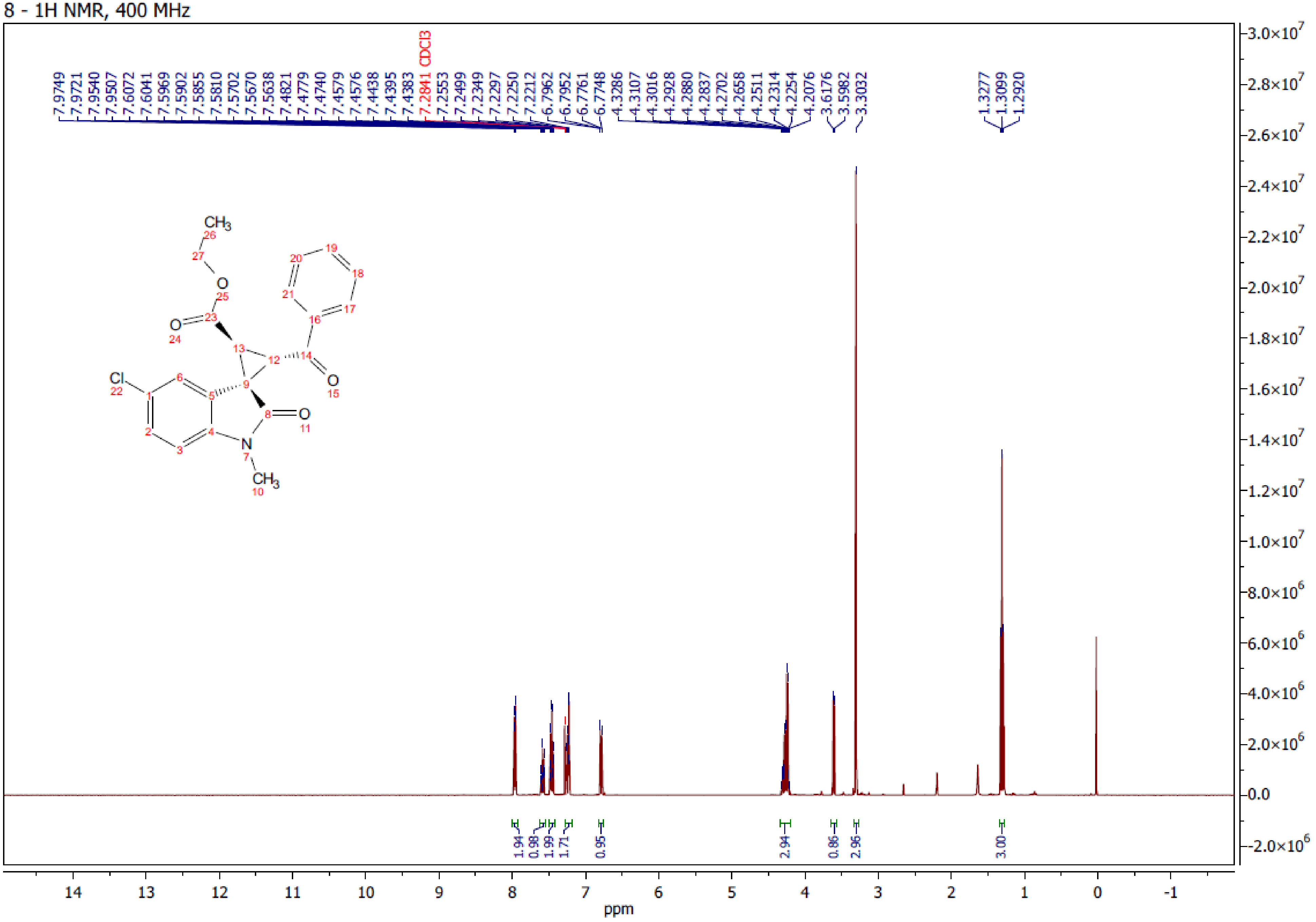
8 - 1H NMR, 400 MHz
7.9749
7.9721
7.9540
7.9507
7.6072
7.6041
7.5969
7.5902
7.5855
7.5810
7.5702
7.5670
7.5638
7.4821
7.4779
7.4740
7.4579
7.4576
7.4438
7.4395
7.4383
7.2841 CDCl3
7.2553
7.2499
7.2349
7.2297
7.2250
7.2212
6.7962
6.7952
6.7761
6.7748
4.3286
4.3107
4.3016
4.2928
4.2880
4.2837
4.2702
4.2658
4.2511
4.2314
4.2254
4.2076
3.6176
3.5982
3.3032
1.3277
1.3099
1.2920
1.94
0.98
1.99
1.71
0.95
2.94
0.86
2.96
3.00
ppm

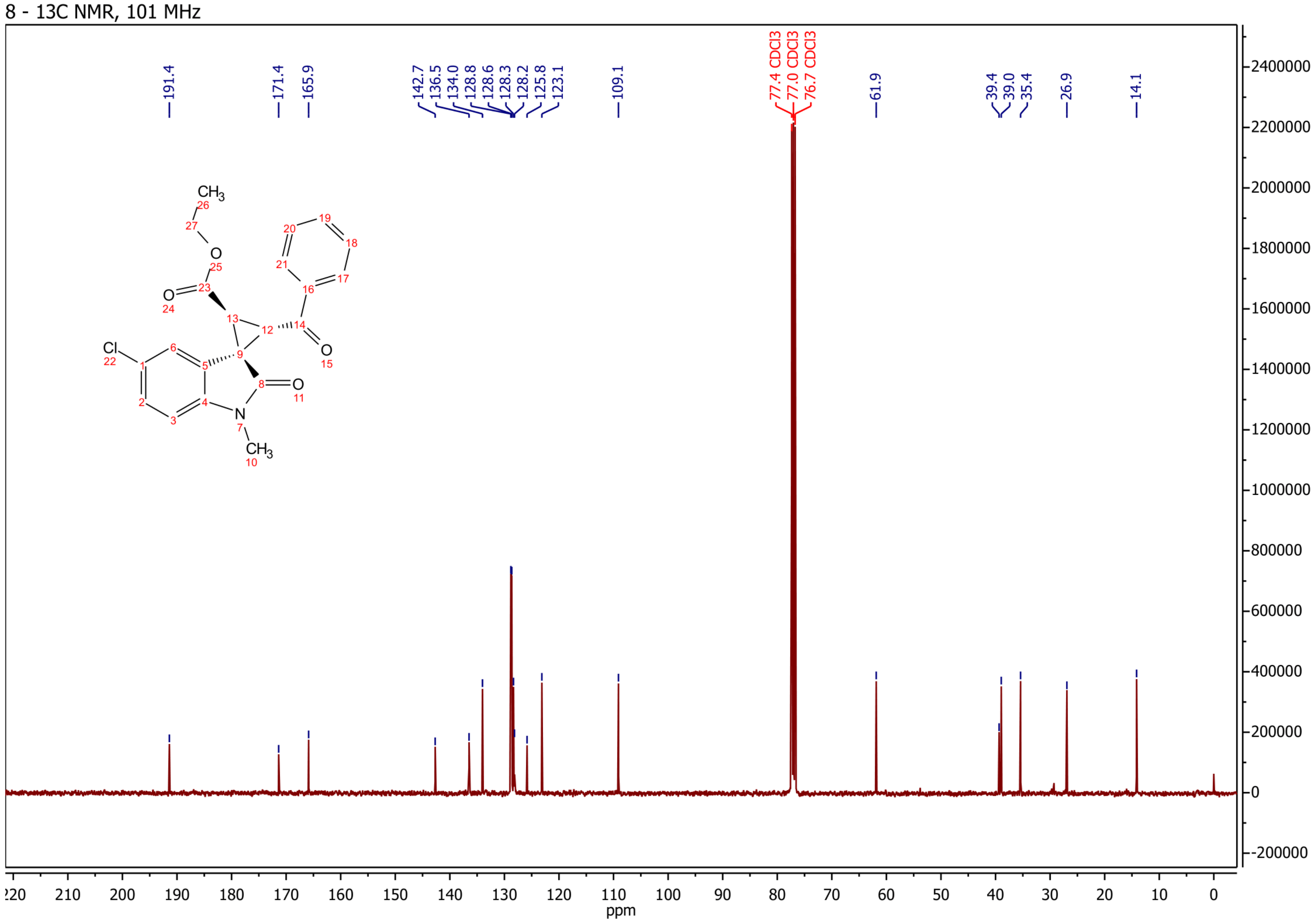

8 - 13C NMR, 101 MHz
191.4
171.4
165.9
142.7
136.5
134.0
128.8
128.6
128.3
128.2
125.8
123.1
109.1
77.4 CDCl3
77.0 CDCl3
76.7 CDCl3
61.9
39.4
39.0
35.4
26.9
14.1
ppm

1D-Selective NOE experiments

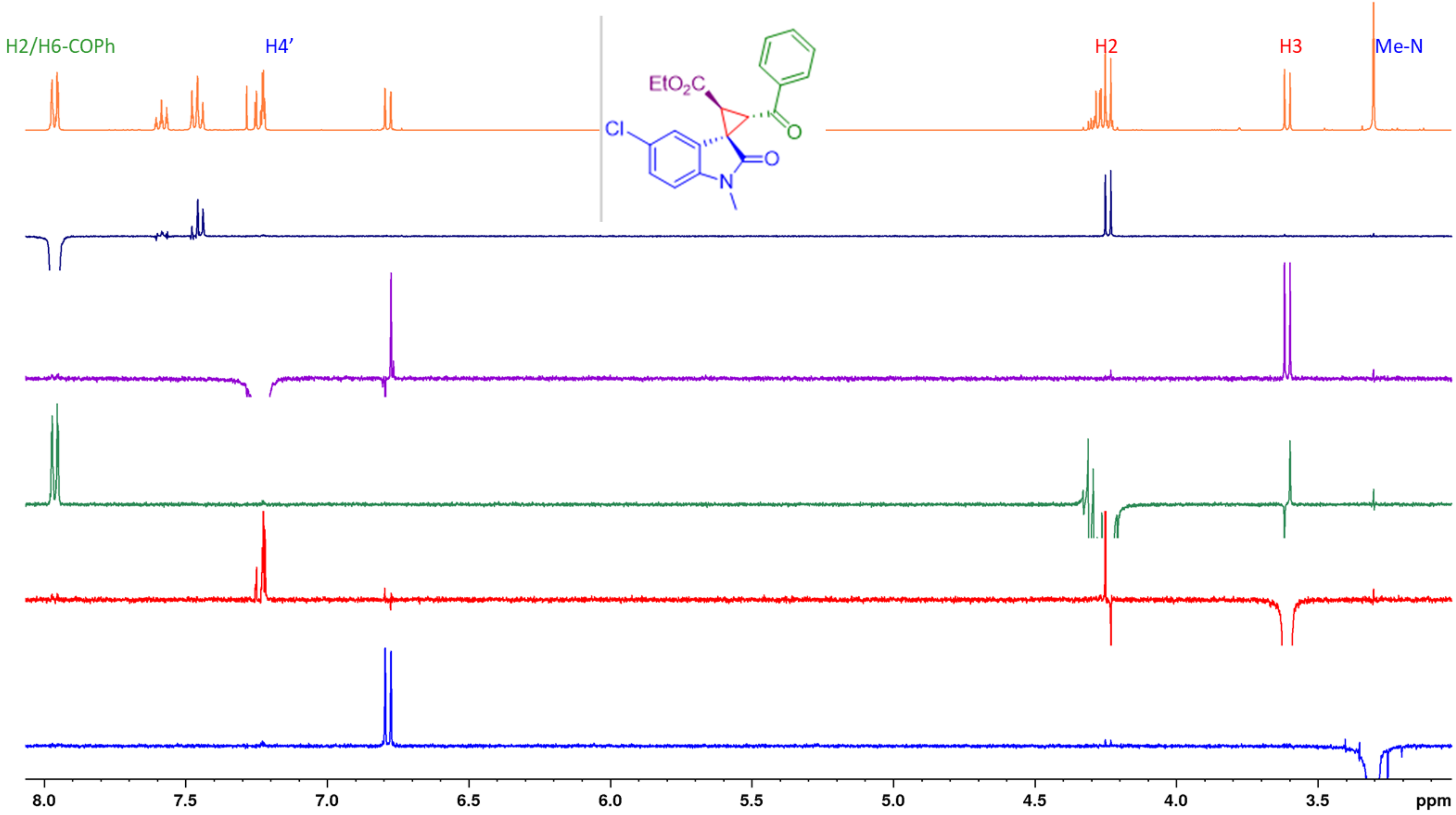

9 - 1H NMR, 400 MHz

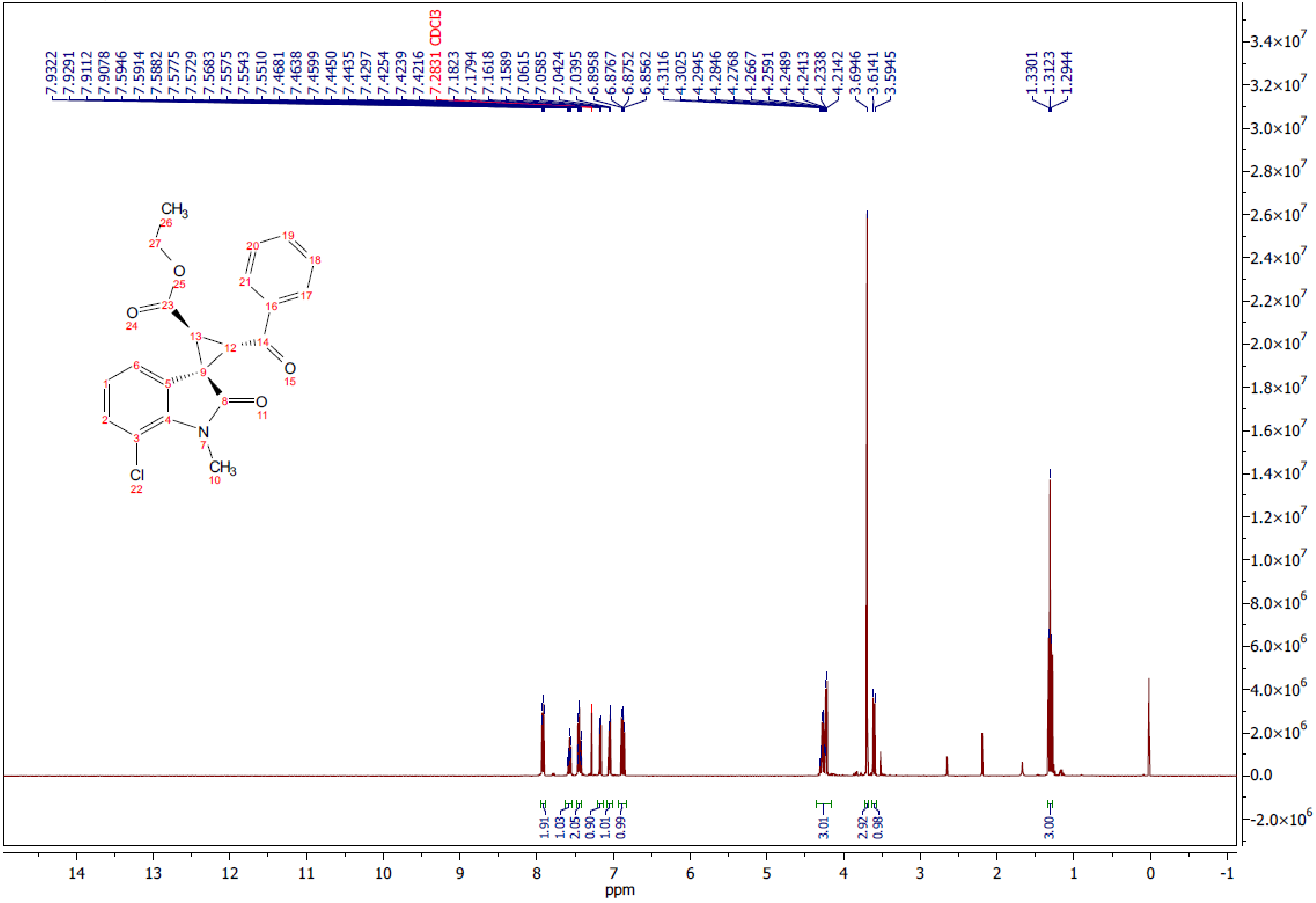

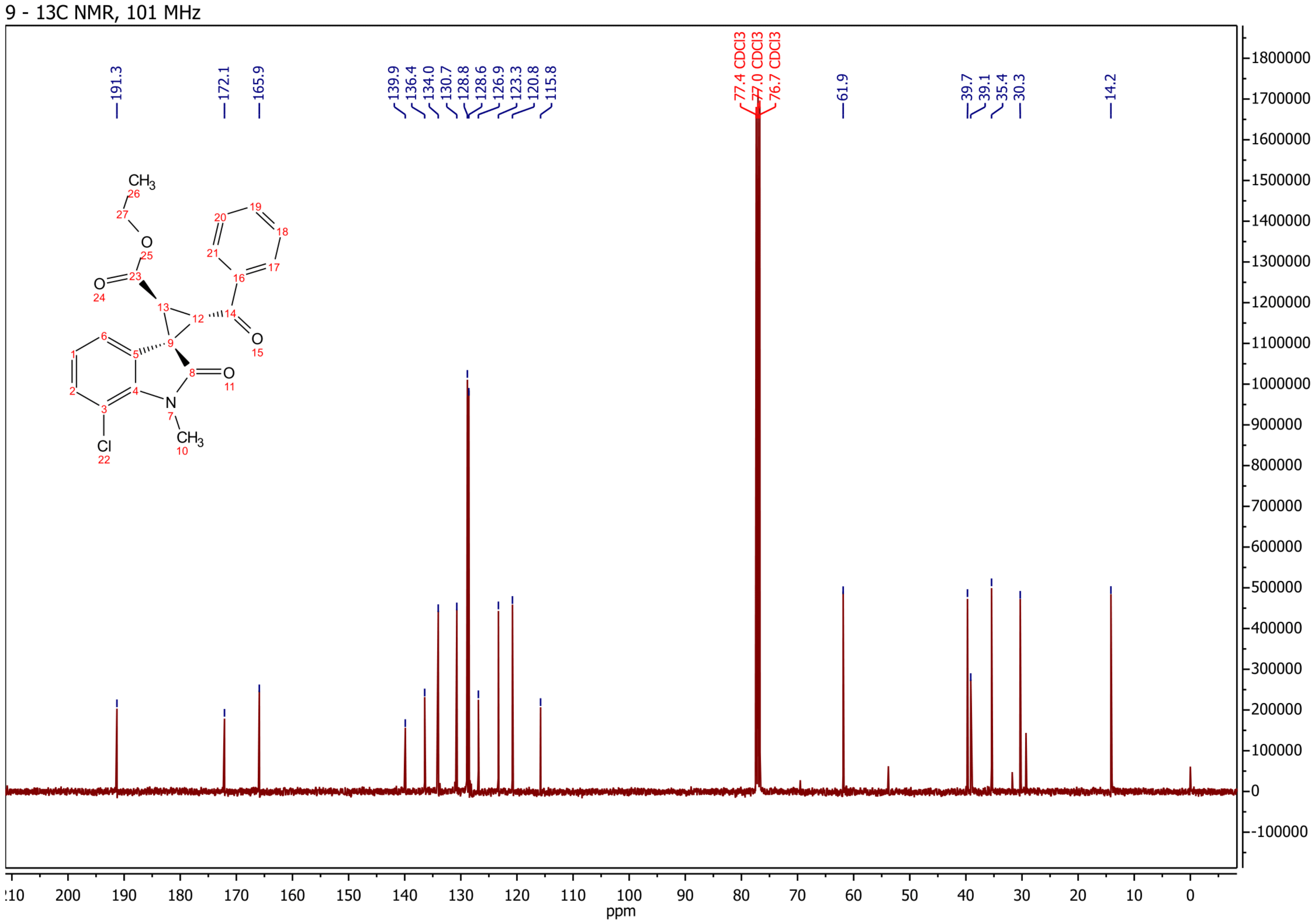
9 - 13C NMR, 101 MHz
191.3
172.1
165.9
139.9
136.4
134.0
130.7
128.8
128.6
126.9
123.3
120.8
115.8
77.4 CDCl3
77.0 CDCl3
76.7 CDCl3
61.9
39.7
39.1
35.4
30.3
14.2
ppm
CH3
O
Cl
N
CH3
O
O
O
O

1D-Selective NOE experiments

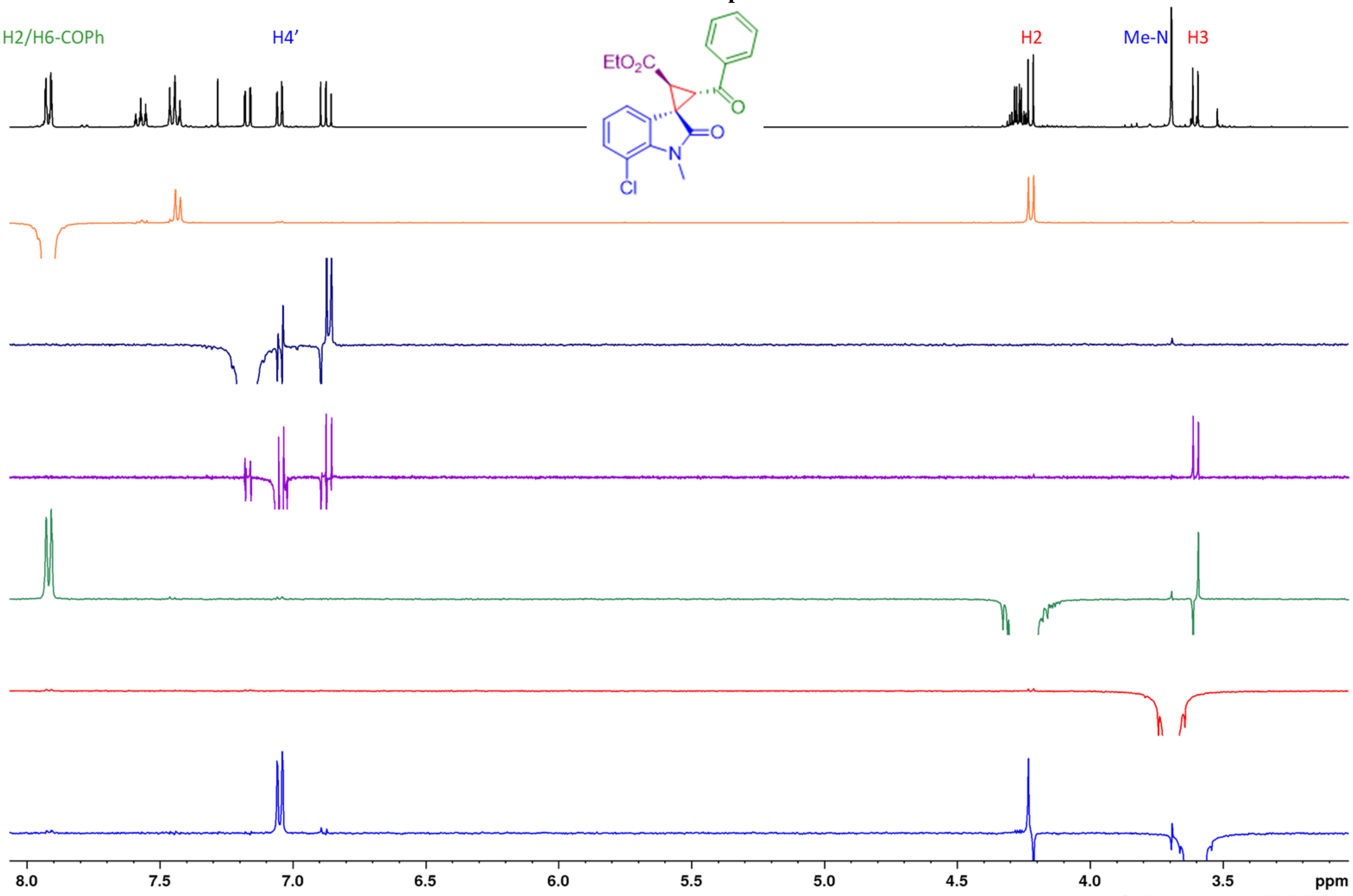

10 - 1H NMR, 400 MHz

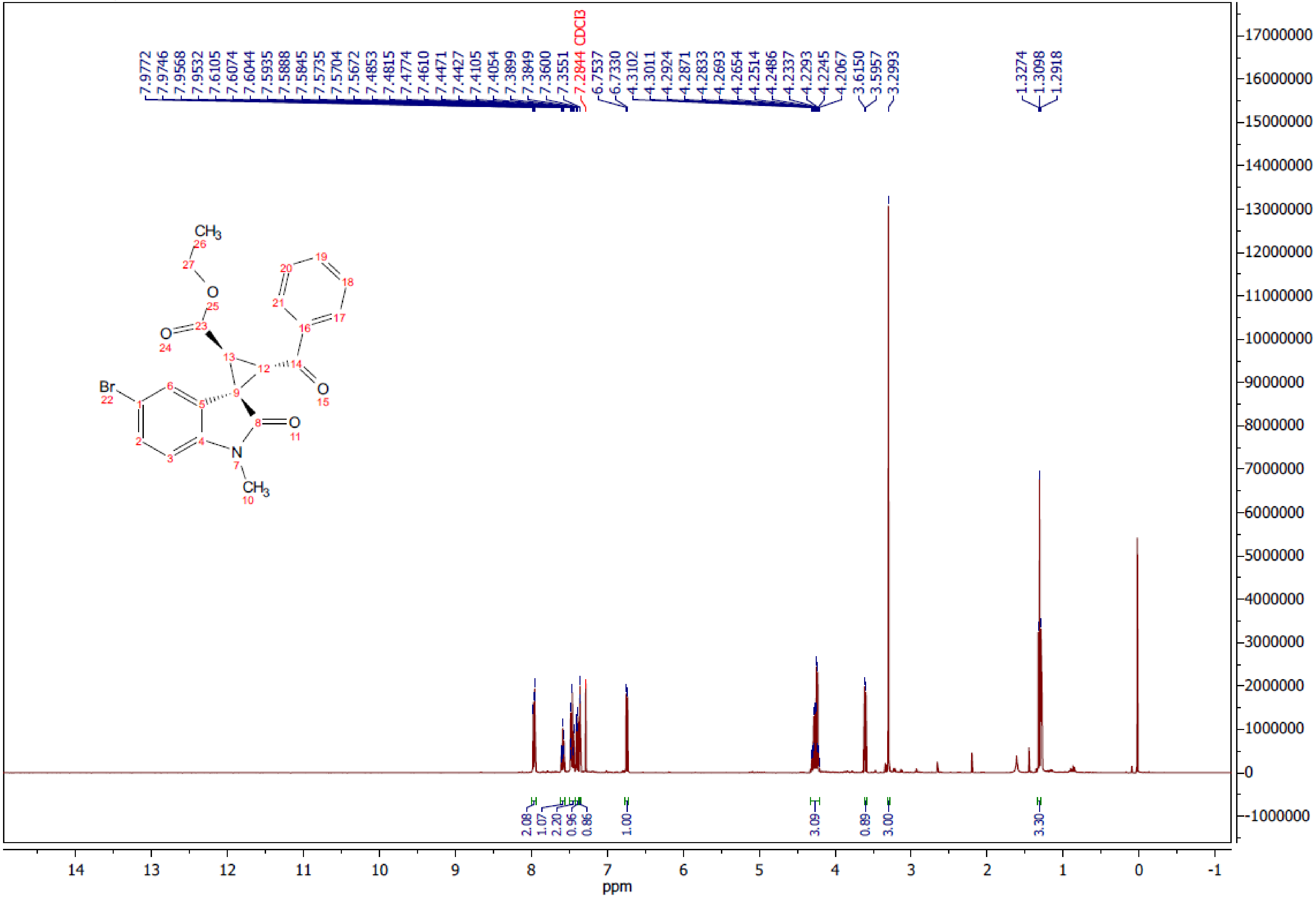

10 - 13C NMR, 126 MHz

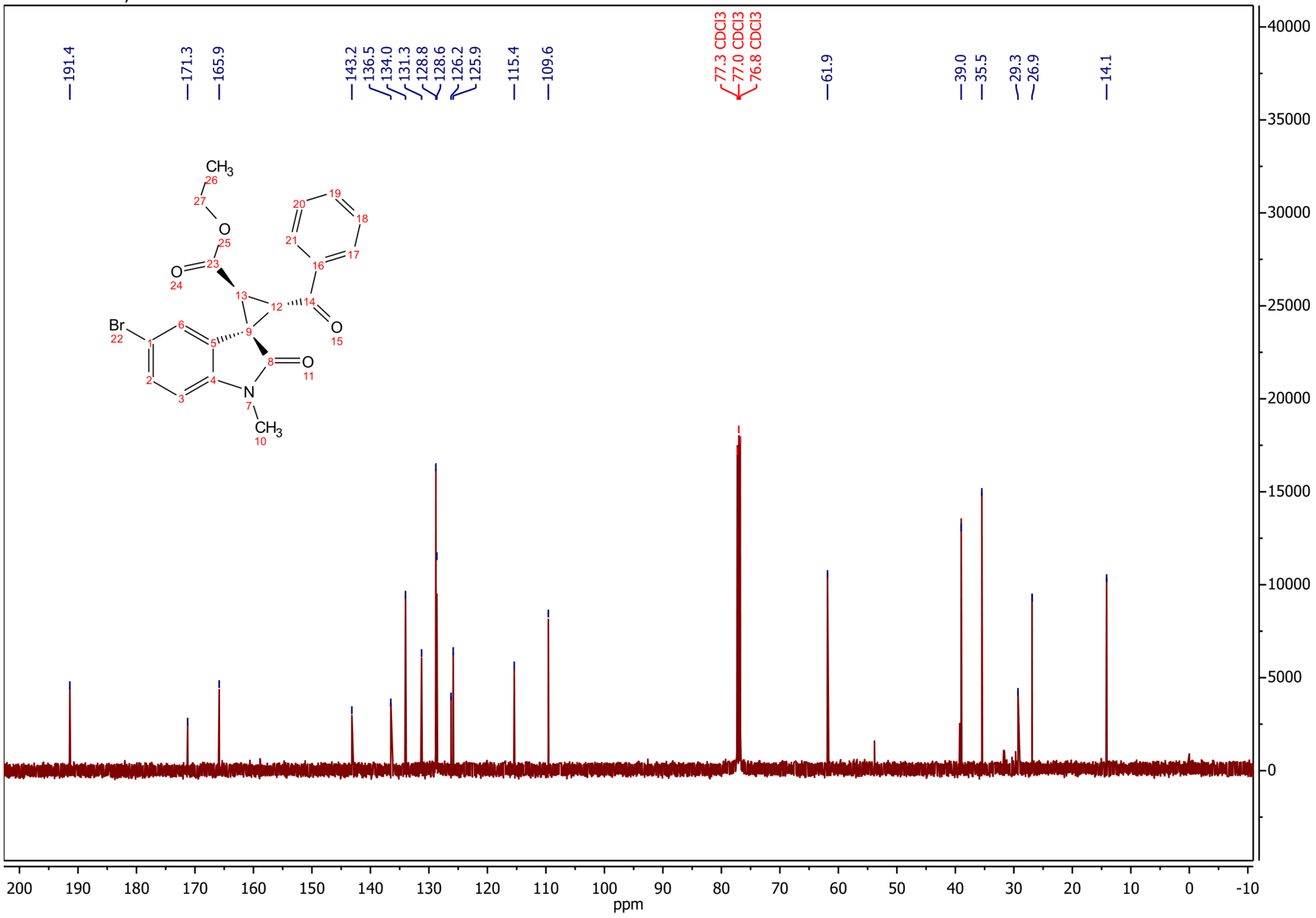

1D-Selective NOE experiments

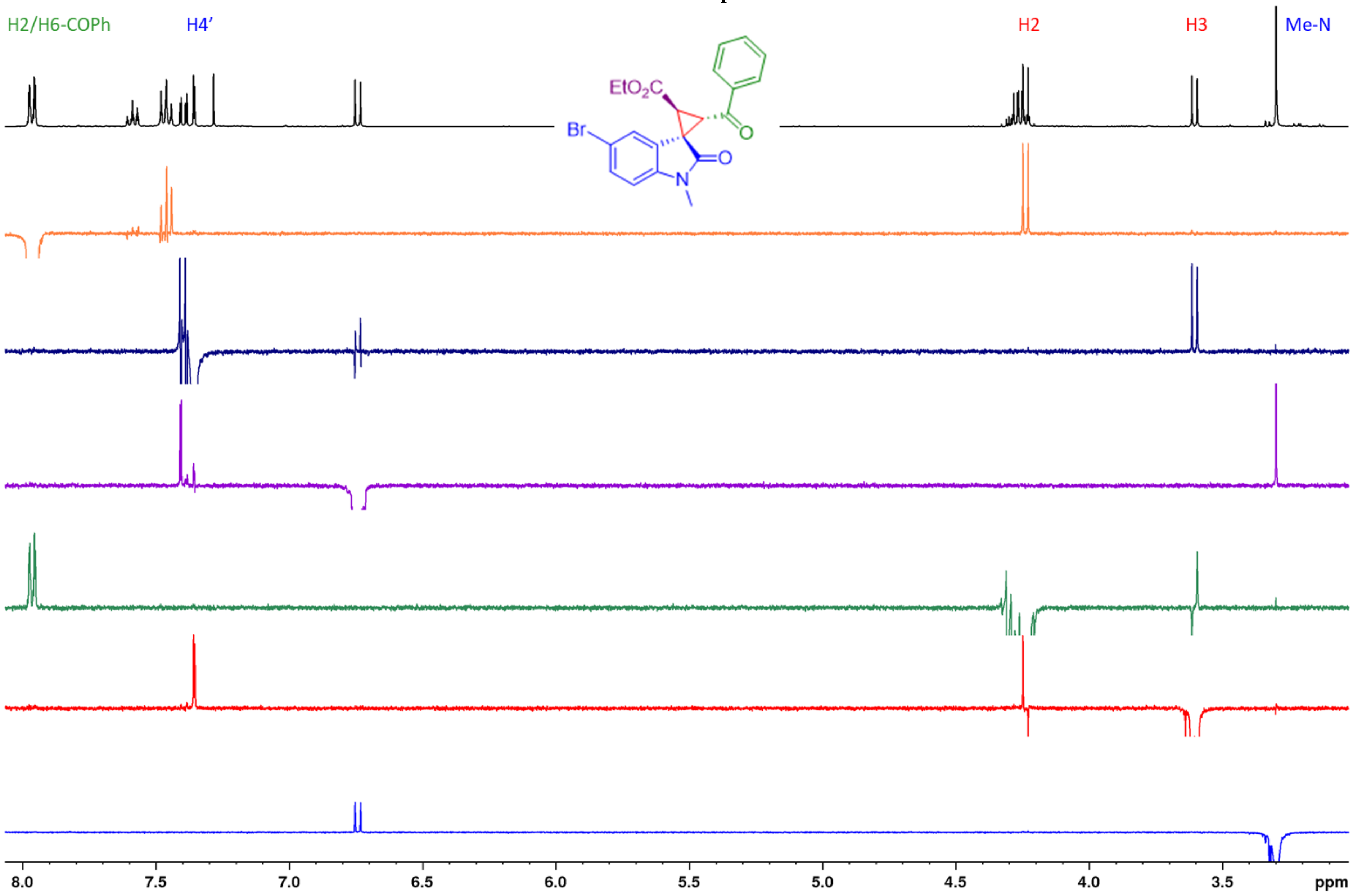

11 - 1H NMR, 400 MHz

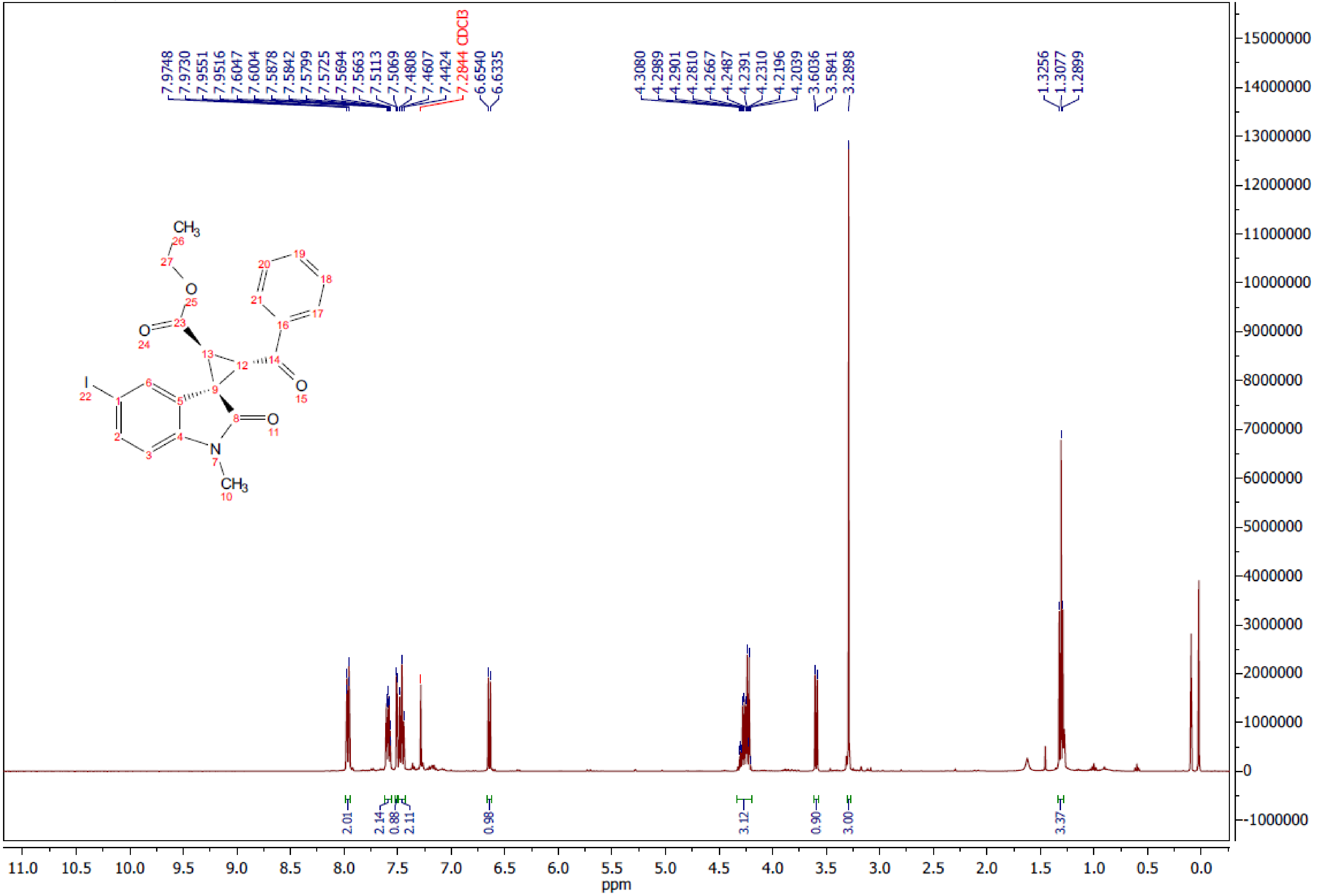

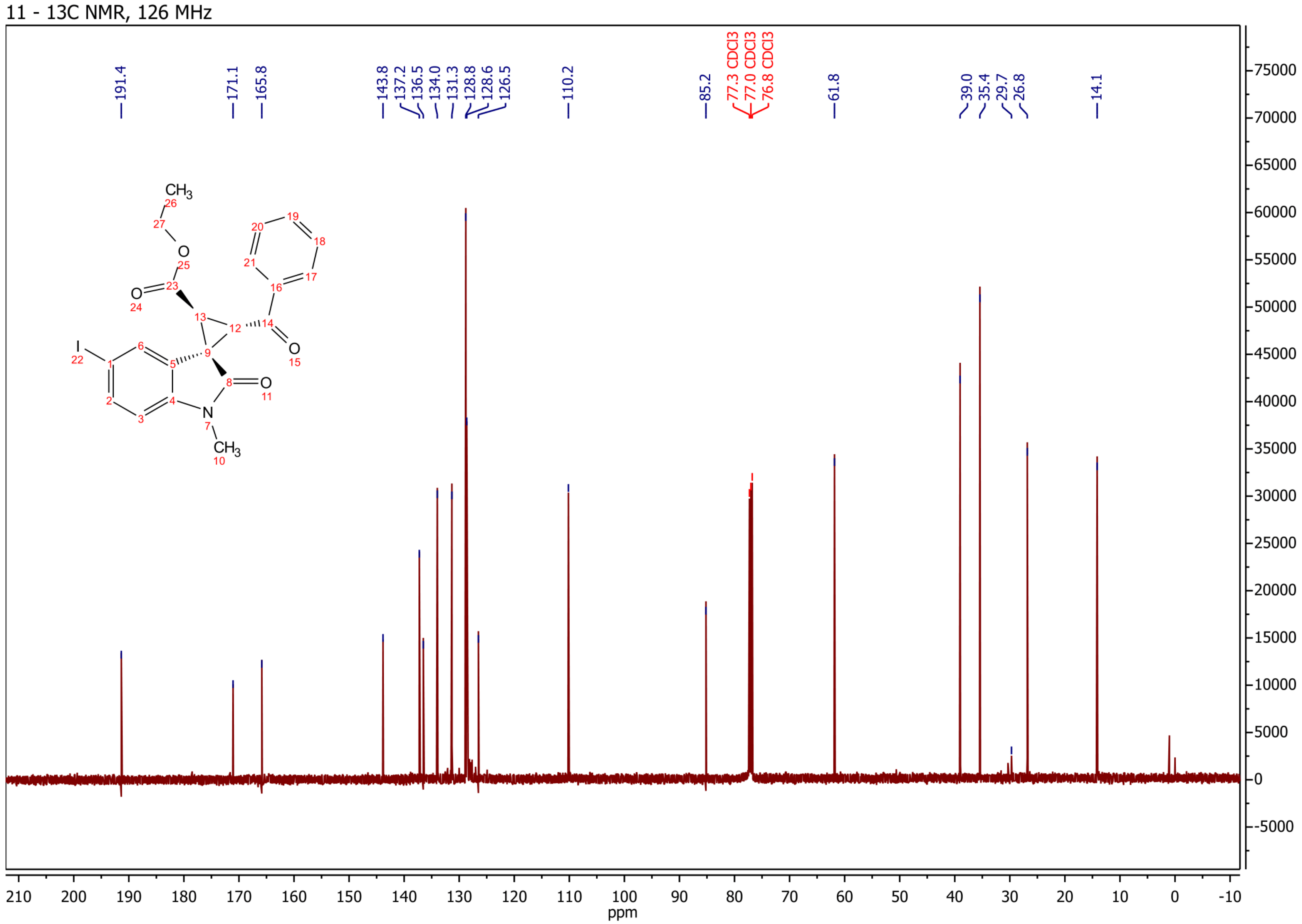

11 - 13C NMR, 126 MHz
191.4
171.1
165.8
143.8
137.2
136.5
134.0
131.3
128.8
128.6
126.5
110.2
85.2
77.3 CDCl3
77.0 CDCl3
76.8 CDCl3
61.8
39.0
35.4
29.7
26.8
14.1
ppm

1D-Selective NOE experiments

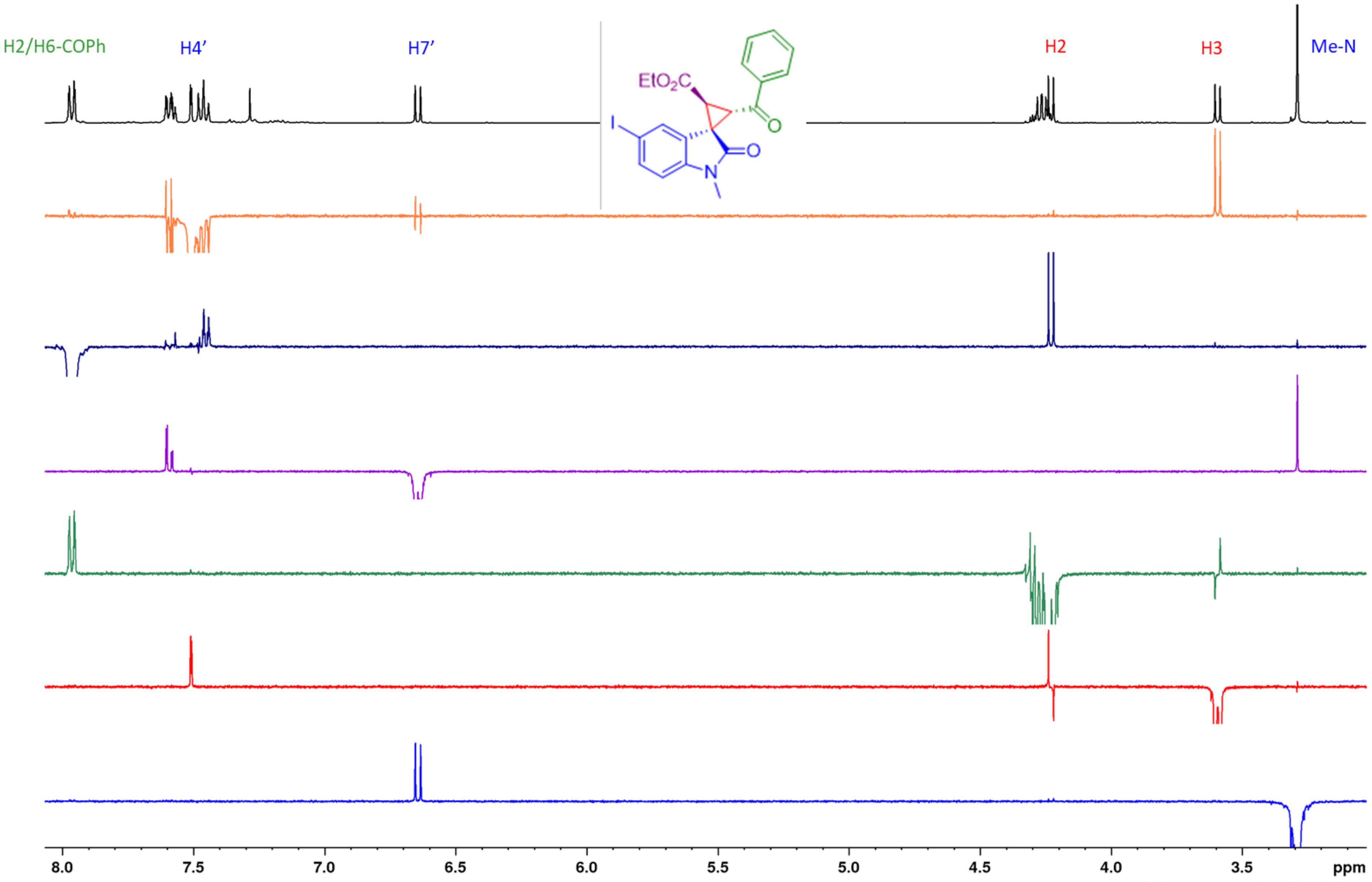

12 - 1H NMR, 400 MHz

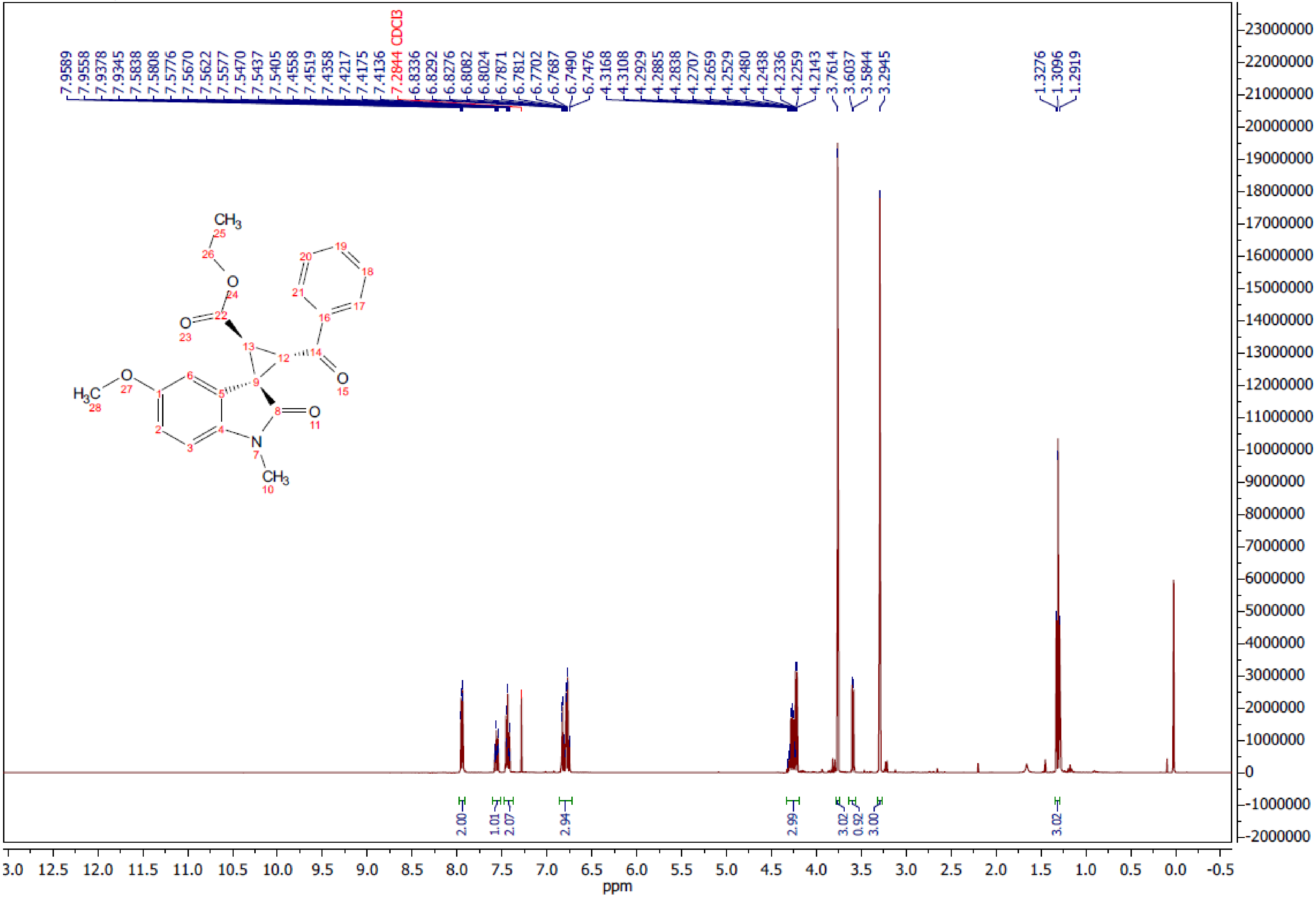

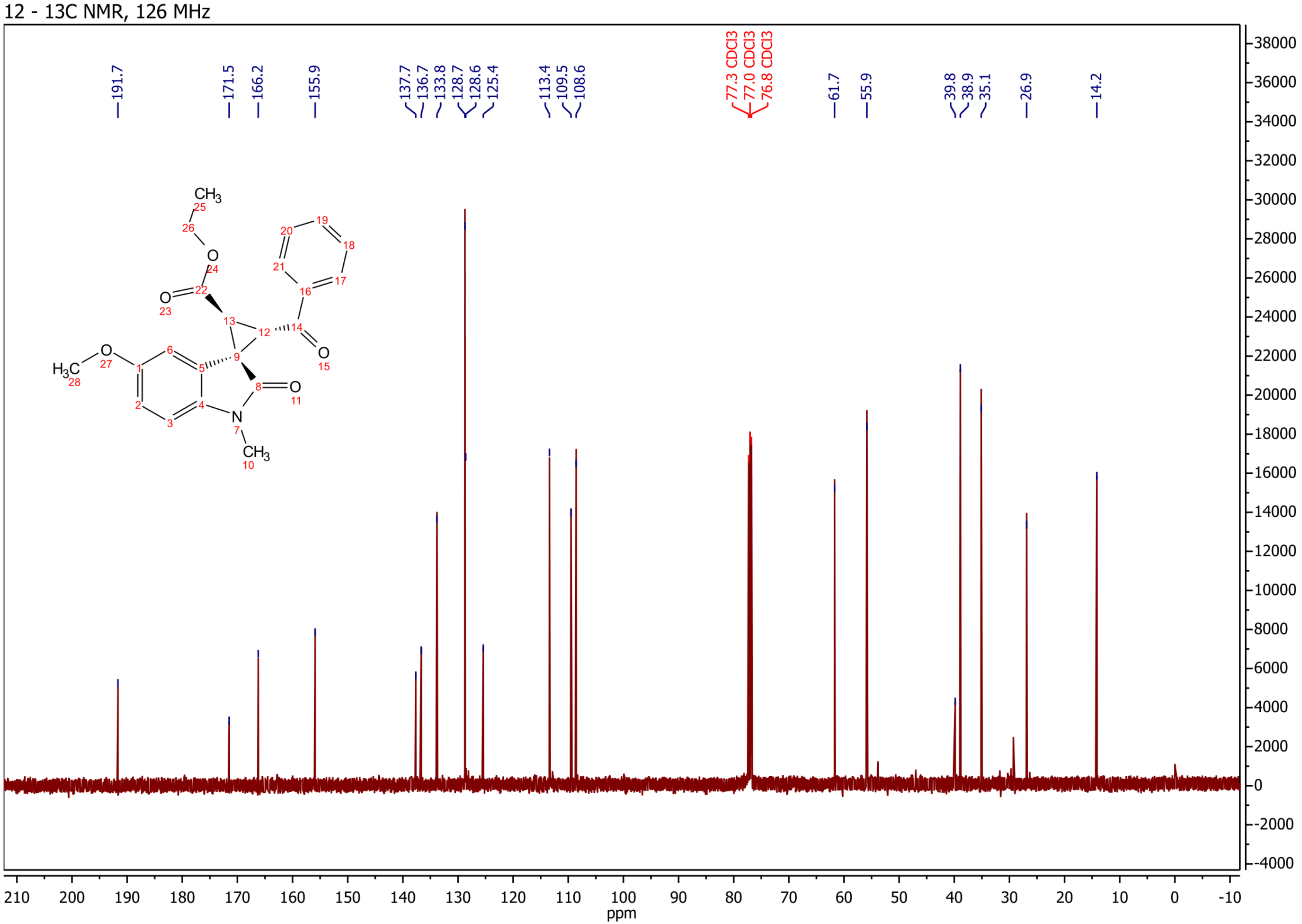
12 - 13C NMR, 126 MHz
191.7
171.5
166.2
155.9
137.7
136.7
133.8
128.7
128.6
125.4
113.4
109.5
108.6
77.3 CDCl3
77.0 CDCl3
76.8 CDCl3
61.7
55.9
39.8
38.9
35.1
26.9
14.2
ppm

1D-Selective NOE experiments

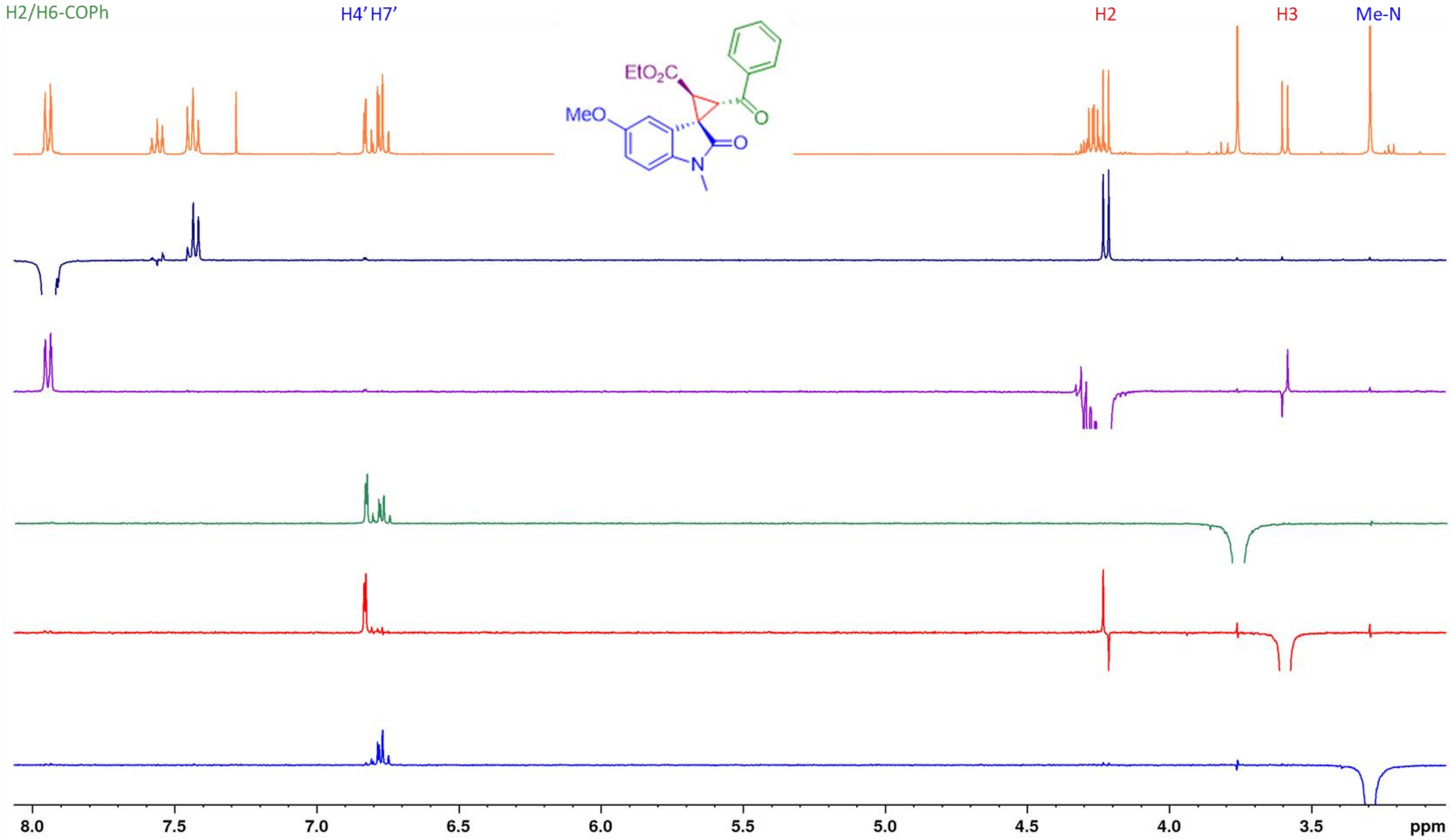

13 - 1H NMR, 500 MHz

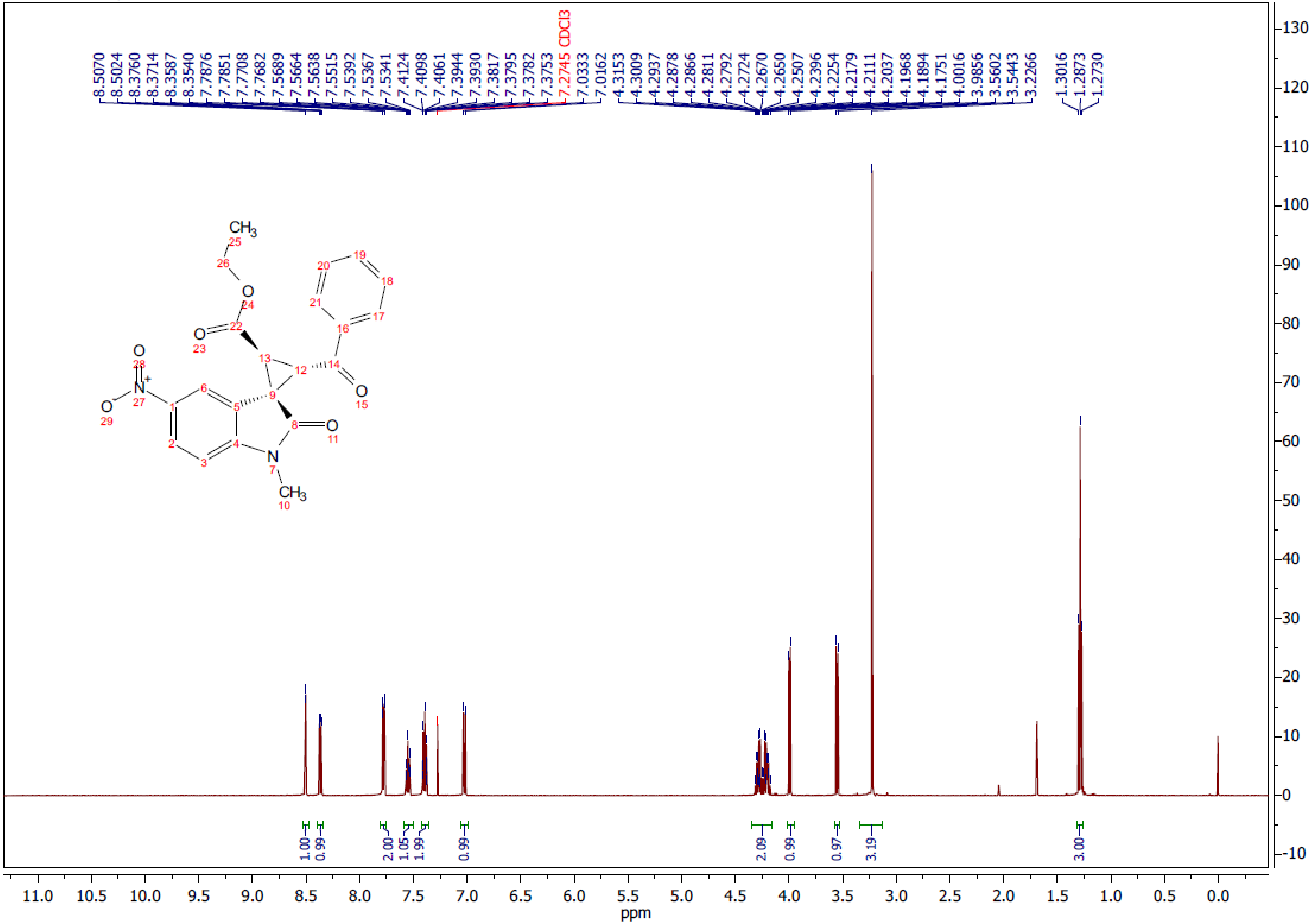

13 - 13C NMR, 126 MHz

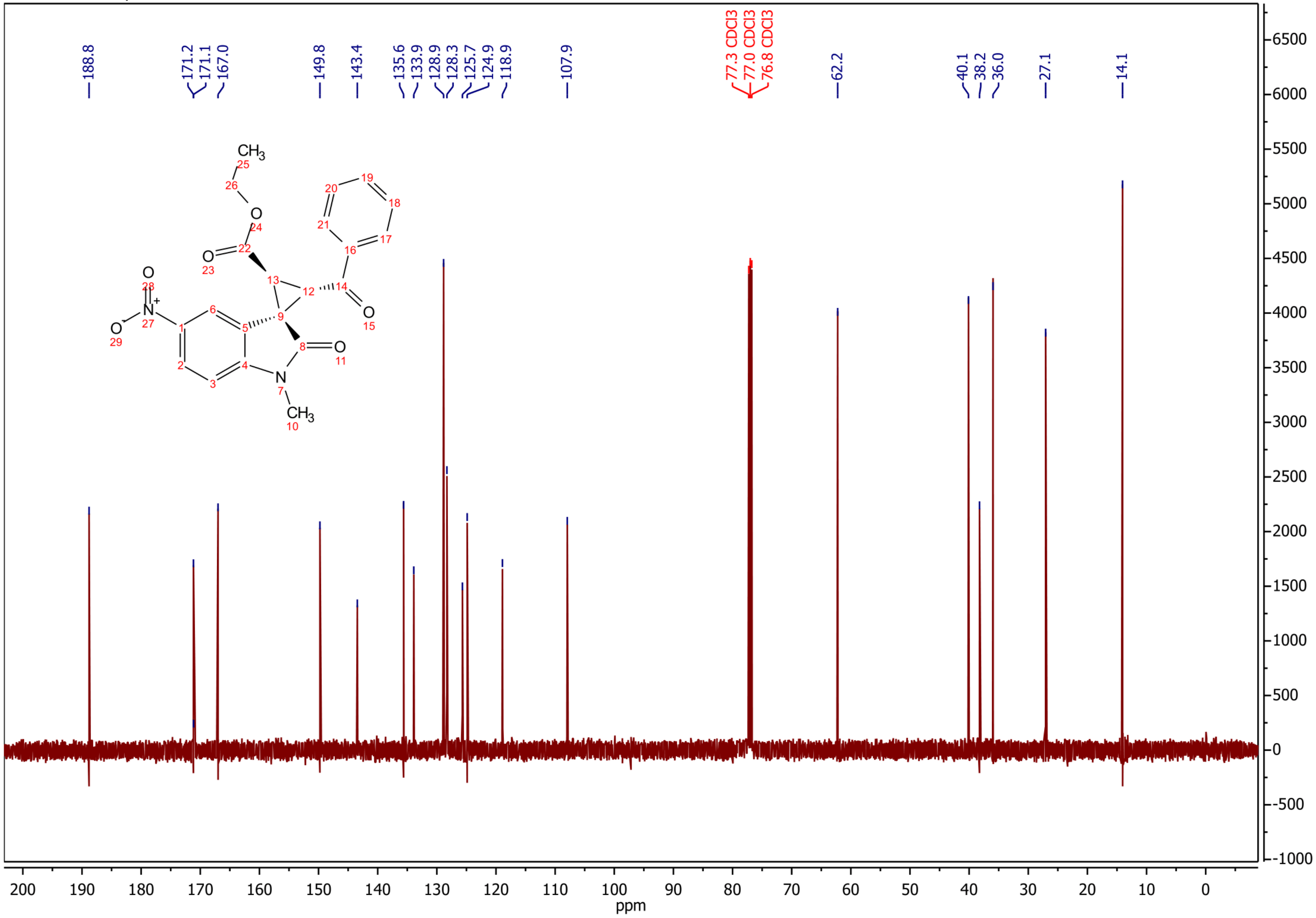

15 - 1H NMR, 500 MHz

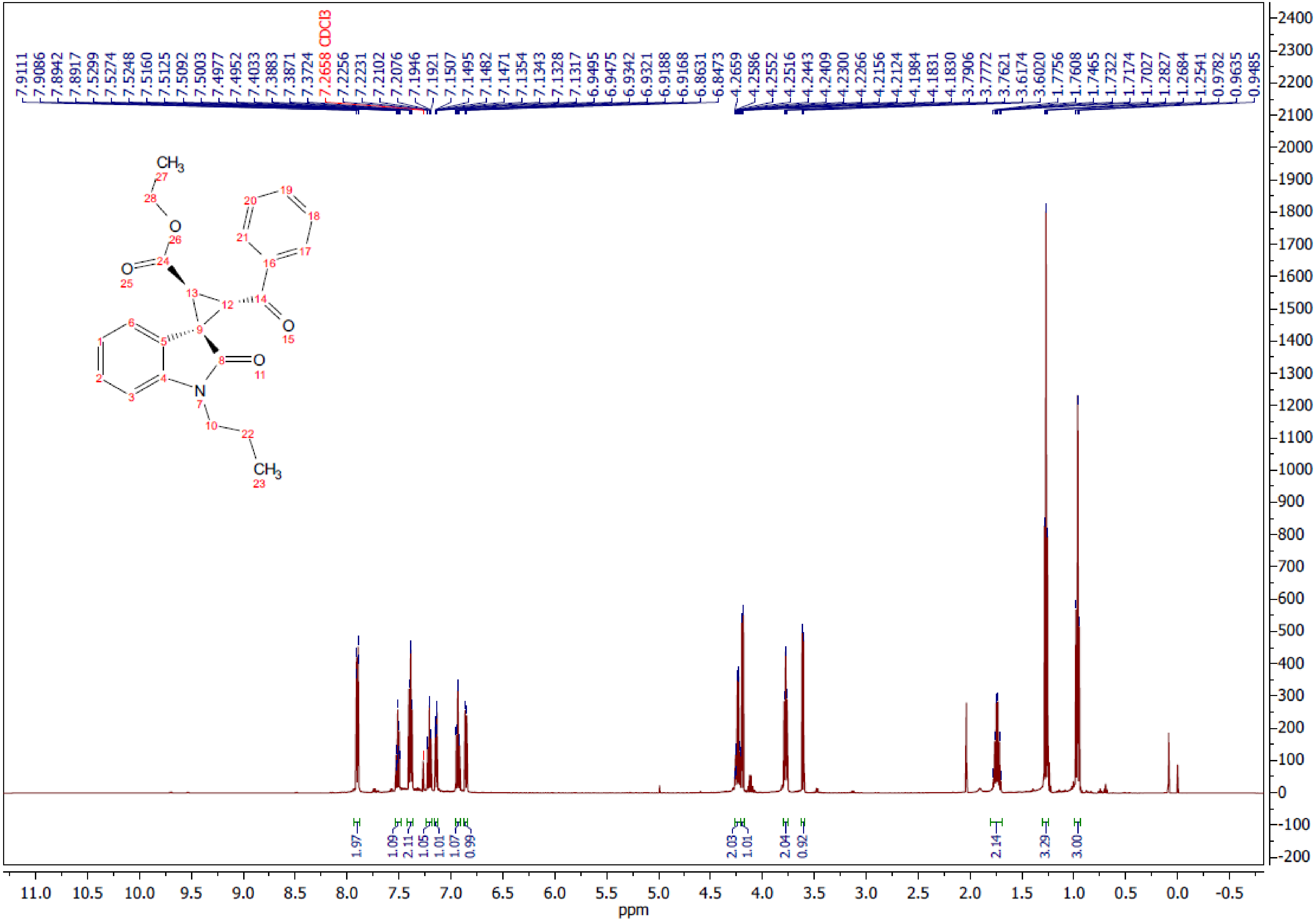

15 - 13C NMR, 126 MHz

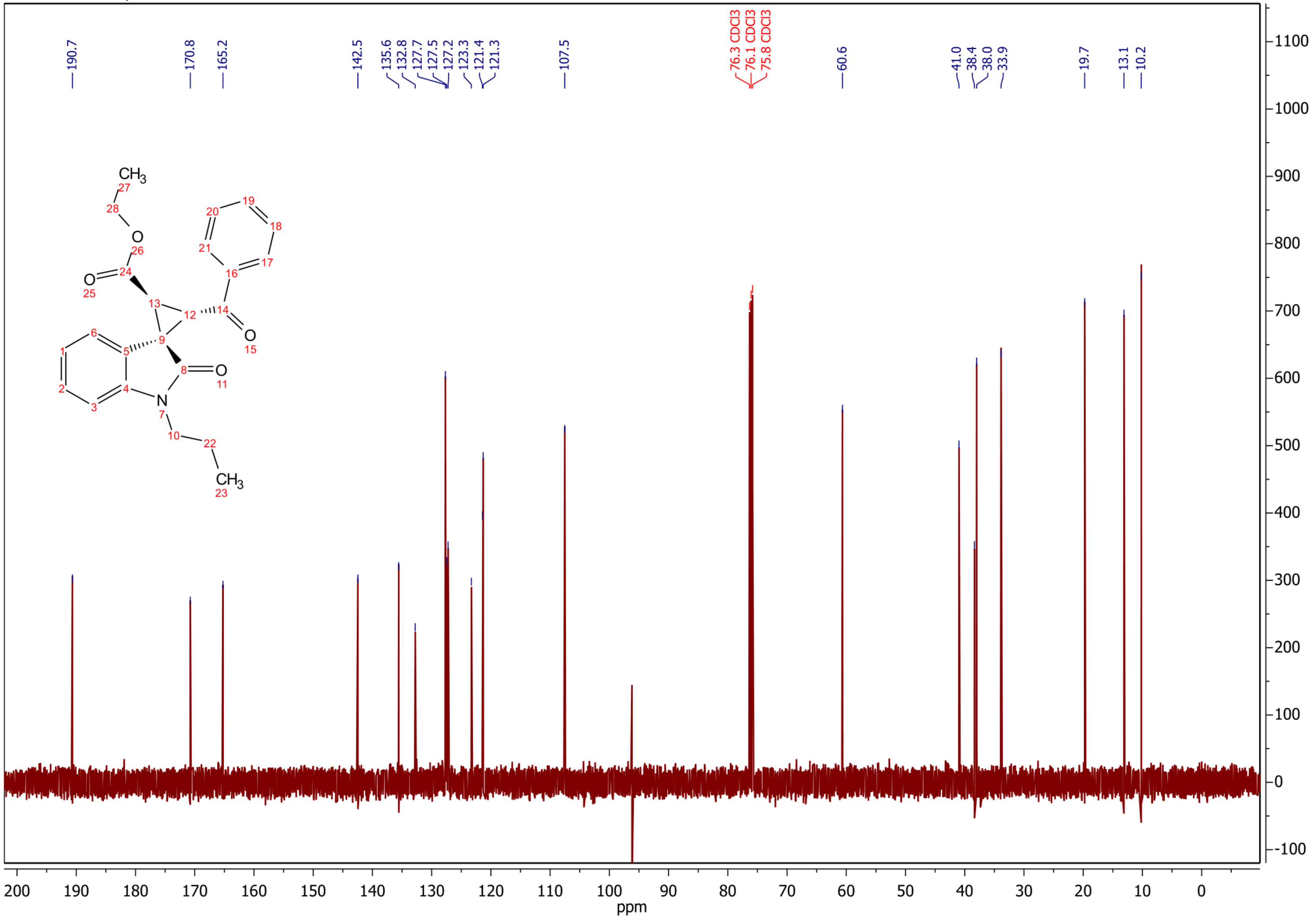

16 - 1H NMR, 500 MHz

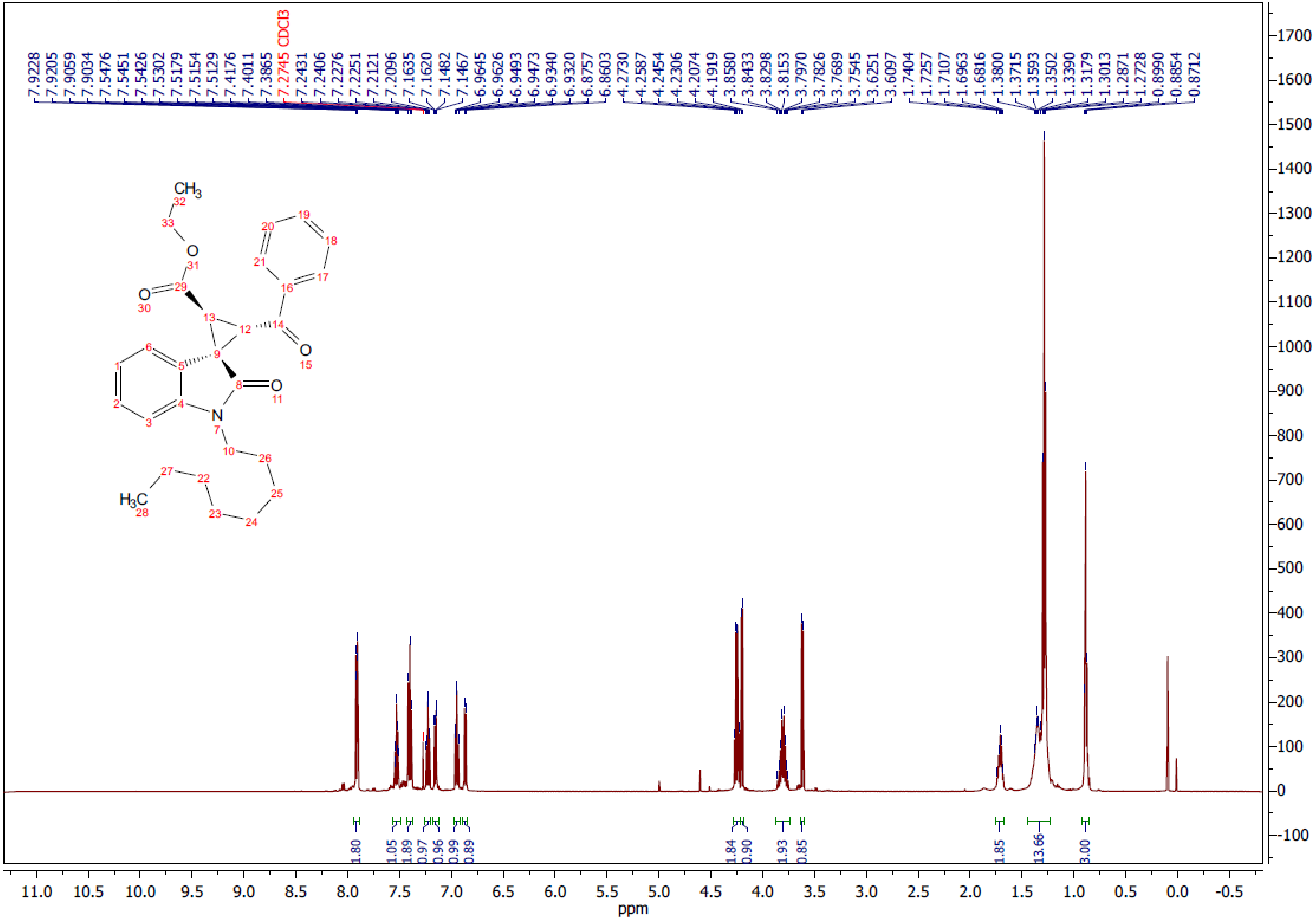

16 - 13C NMR, 126 MHz

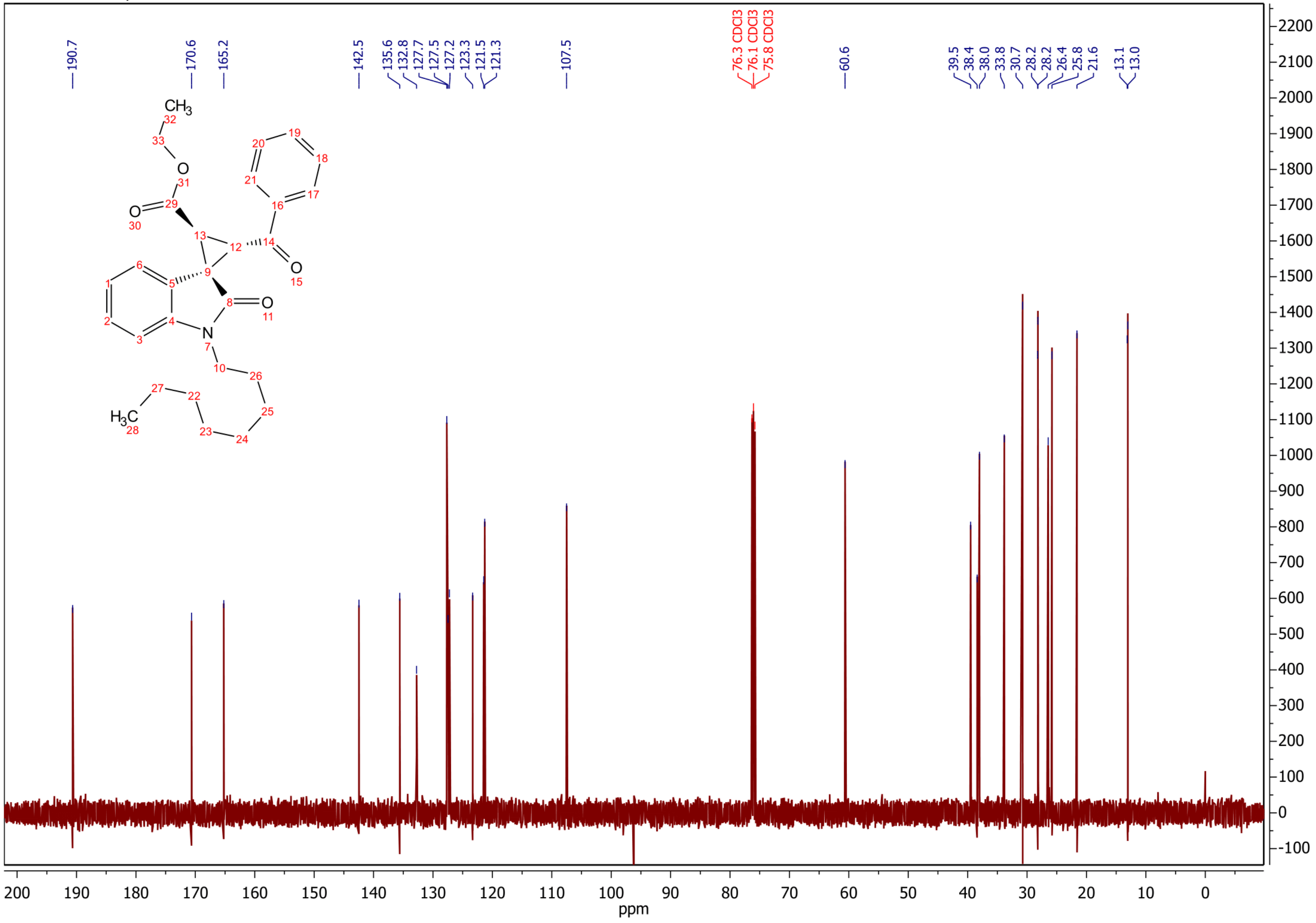

17 - 1H NMR, 400 MHz

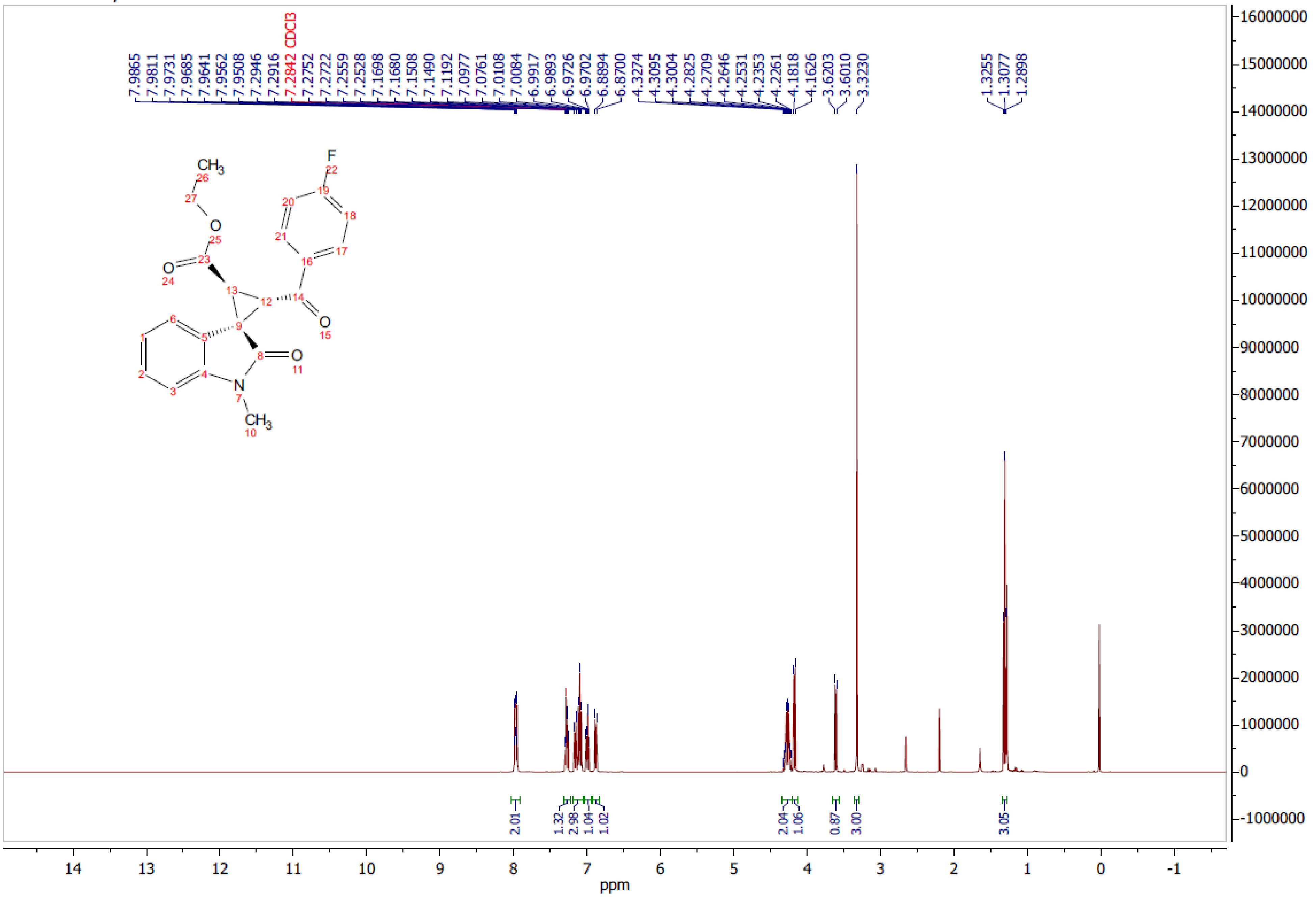

17 - 13C NMR, 101 MHz

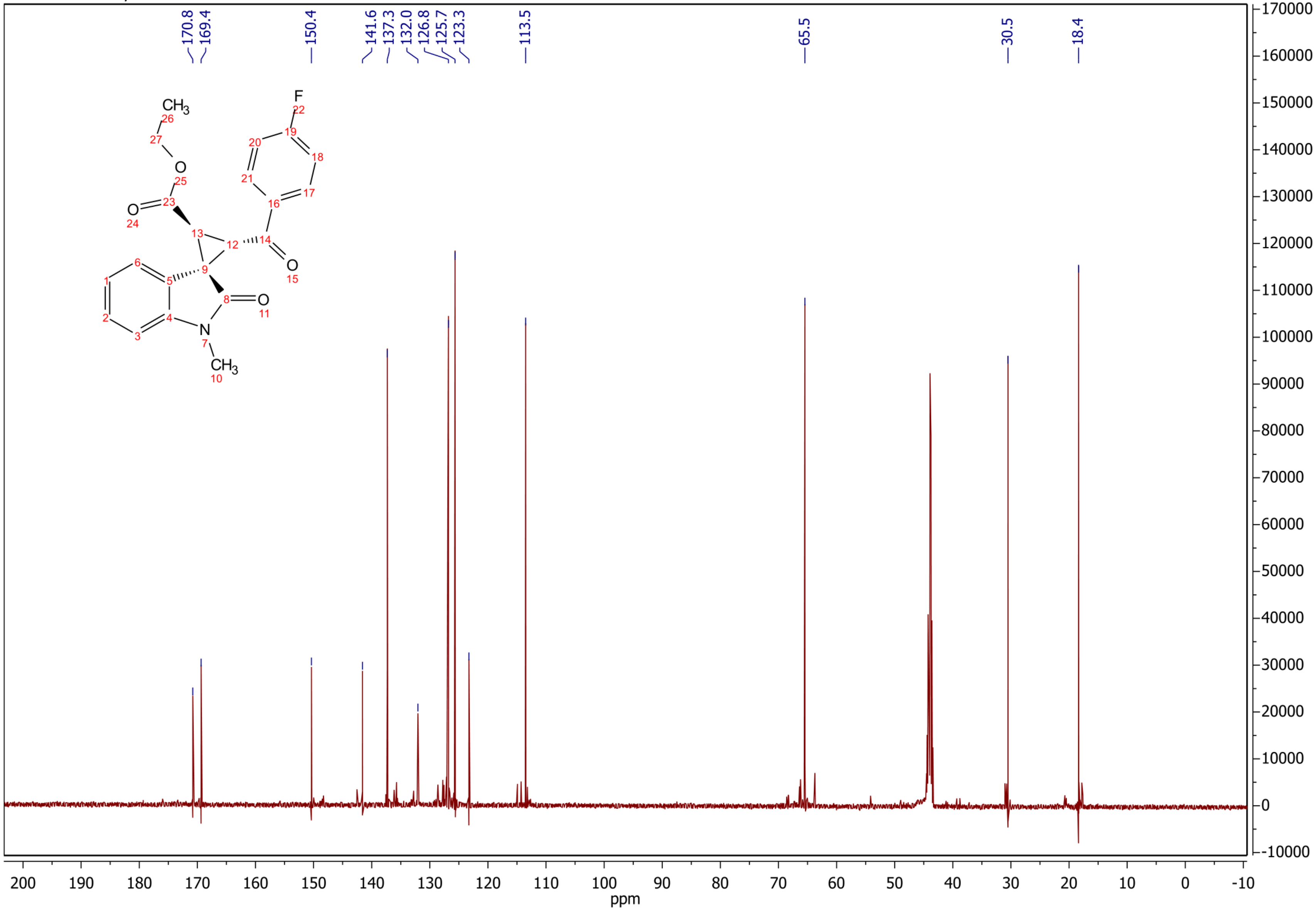

18 - 1H NMR, 500 MHz

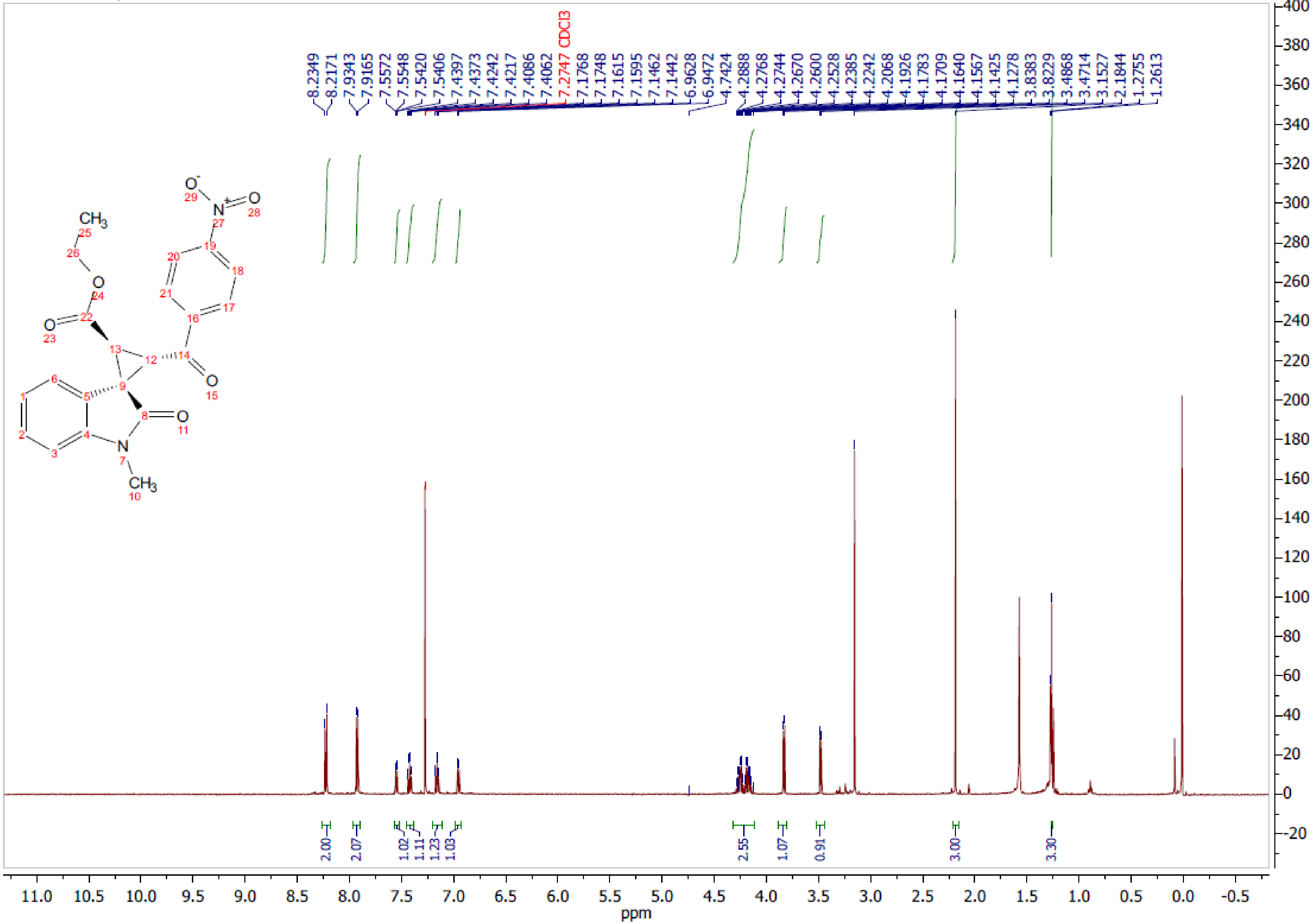

18 - 13C NMR, 126 MHz

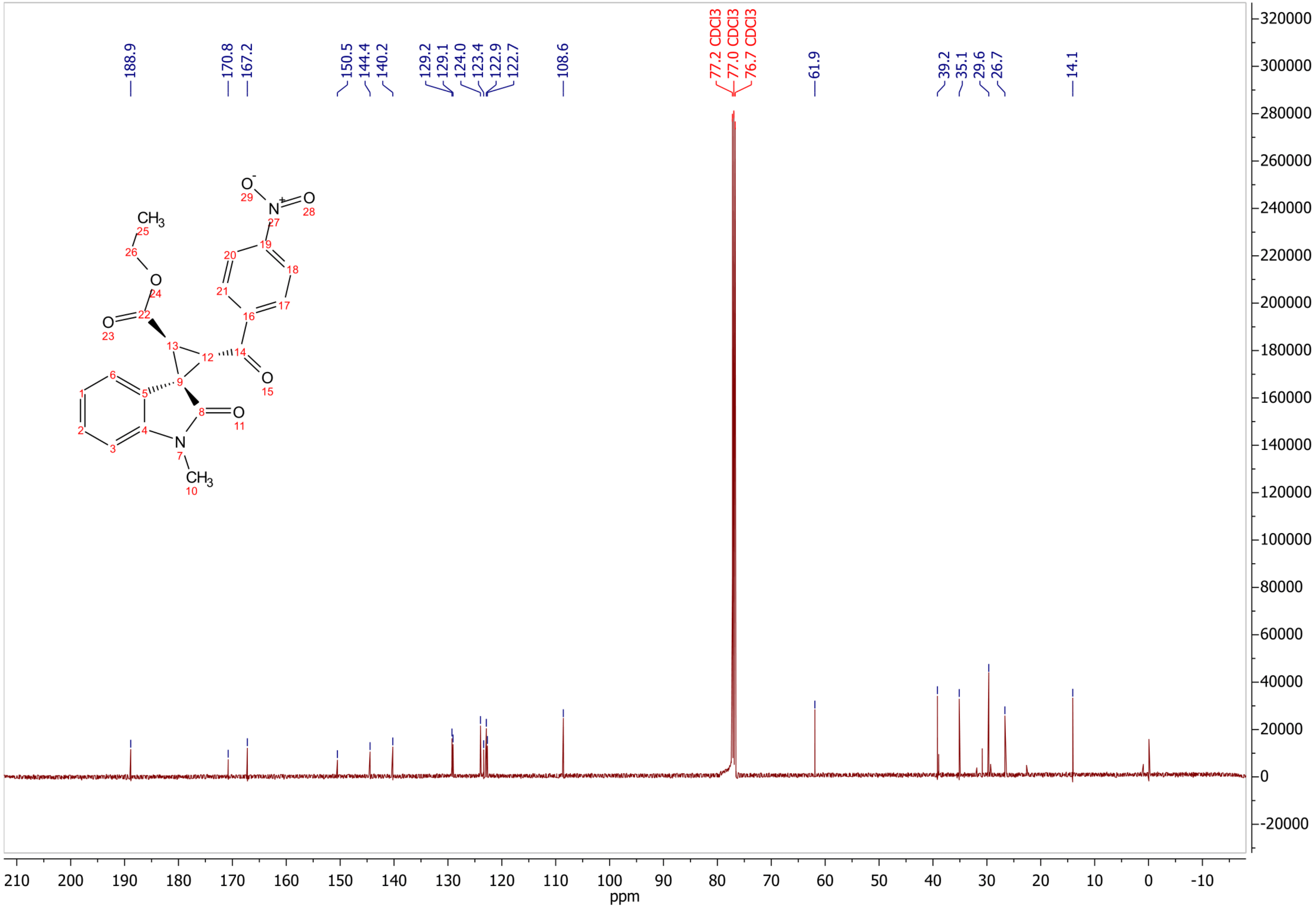

19 - 1H NMR, 500 MHz

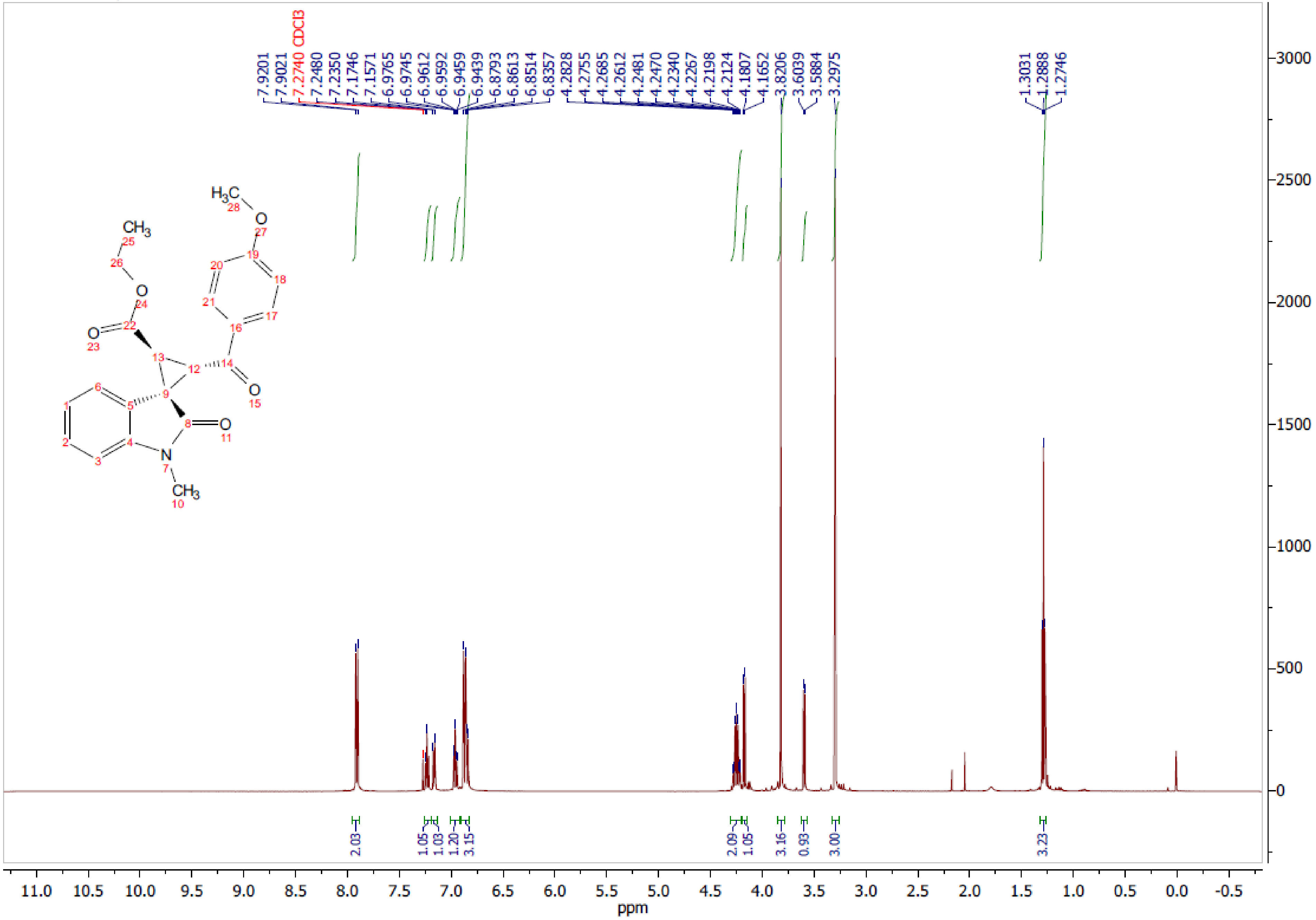

19 - 13C NMR, 126 MHz

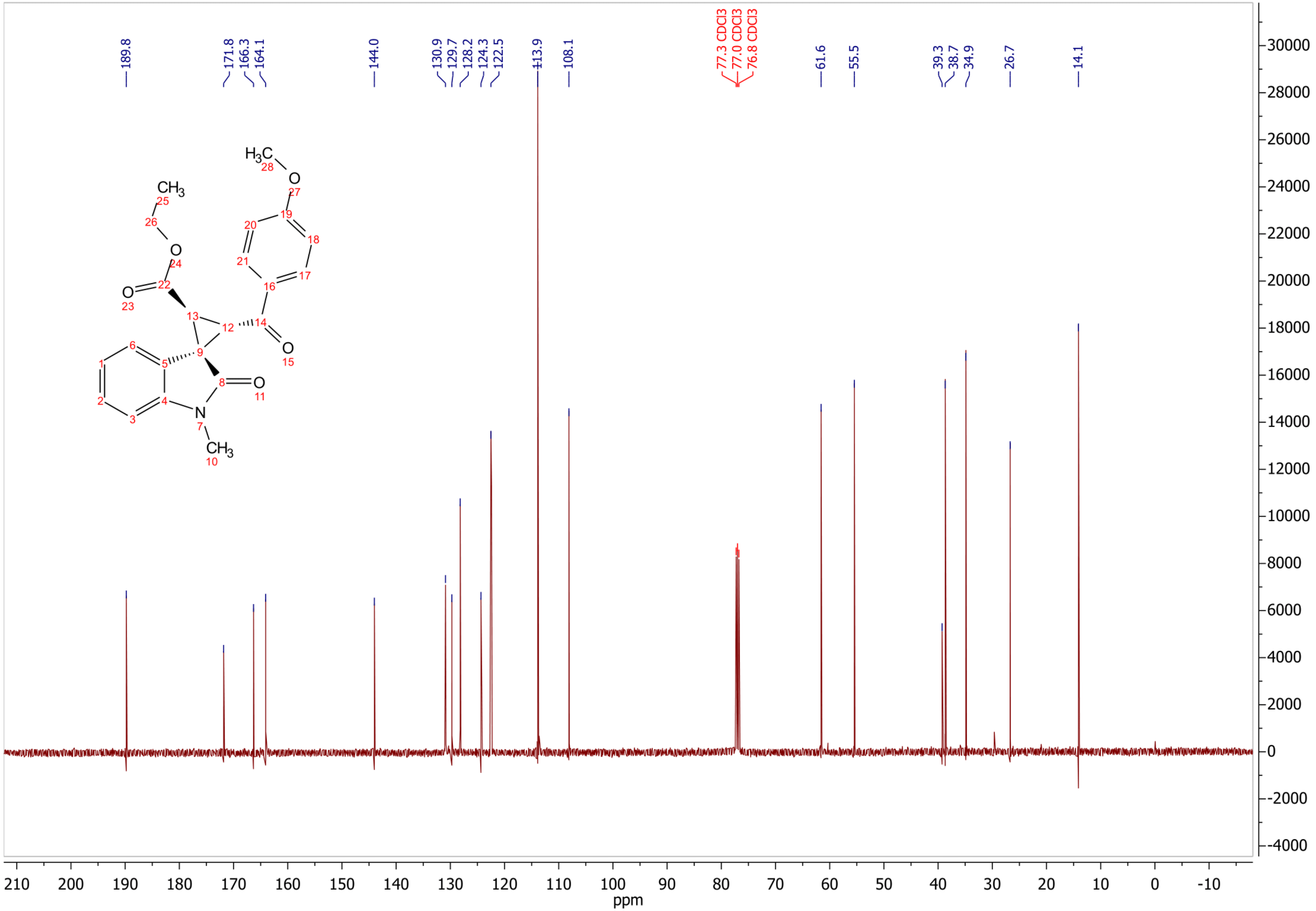

20 - 1H NMR, 500 MHz

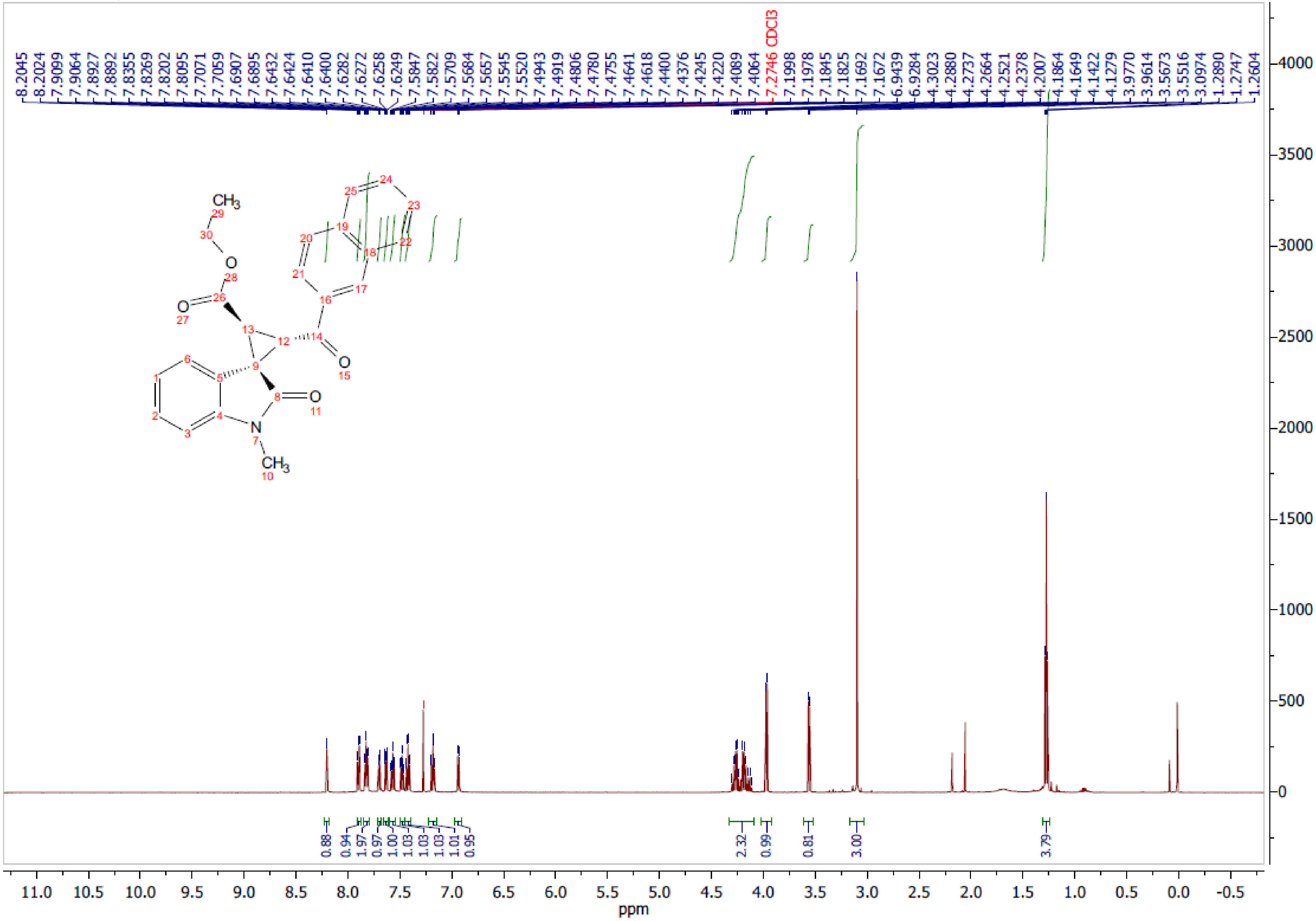

20 - 13C NMR, 126 MHz

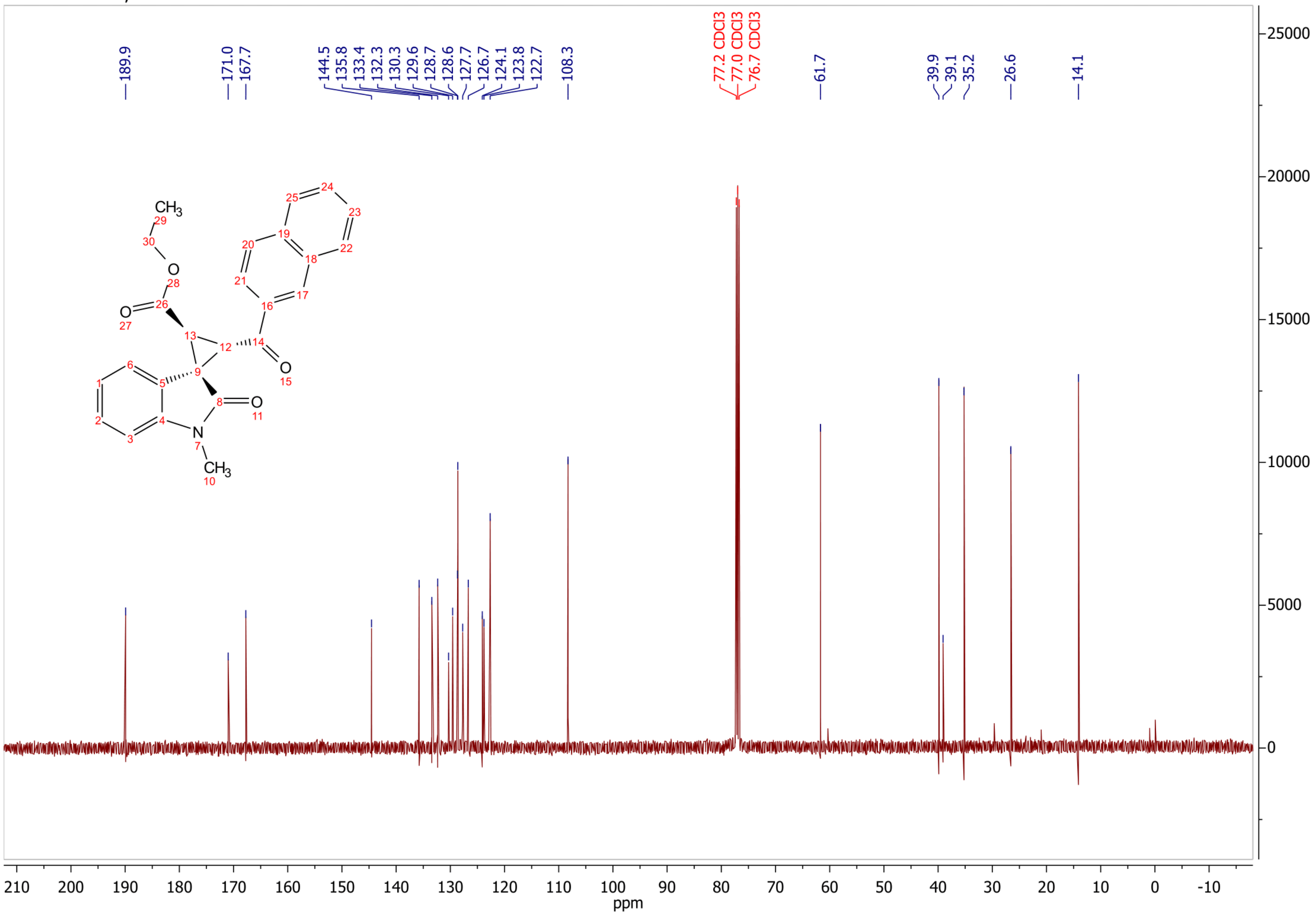

21 - 1H NMR, 500 MHz

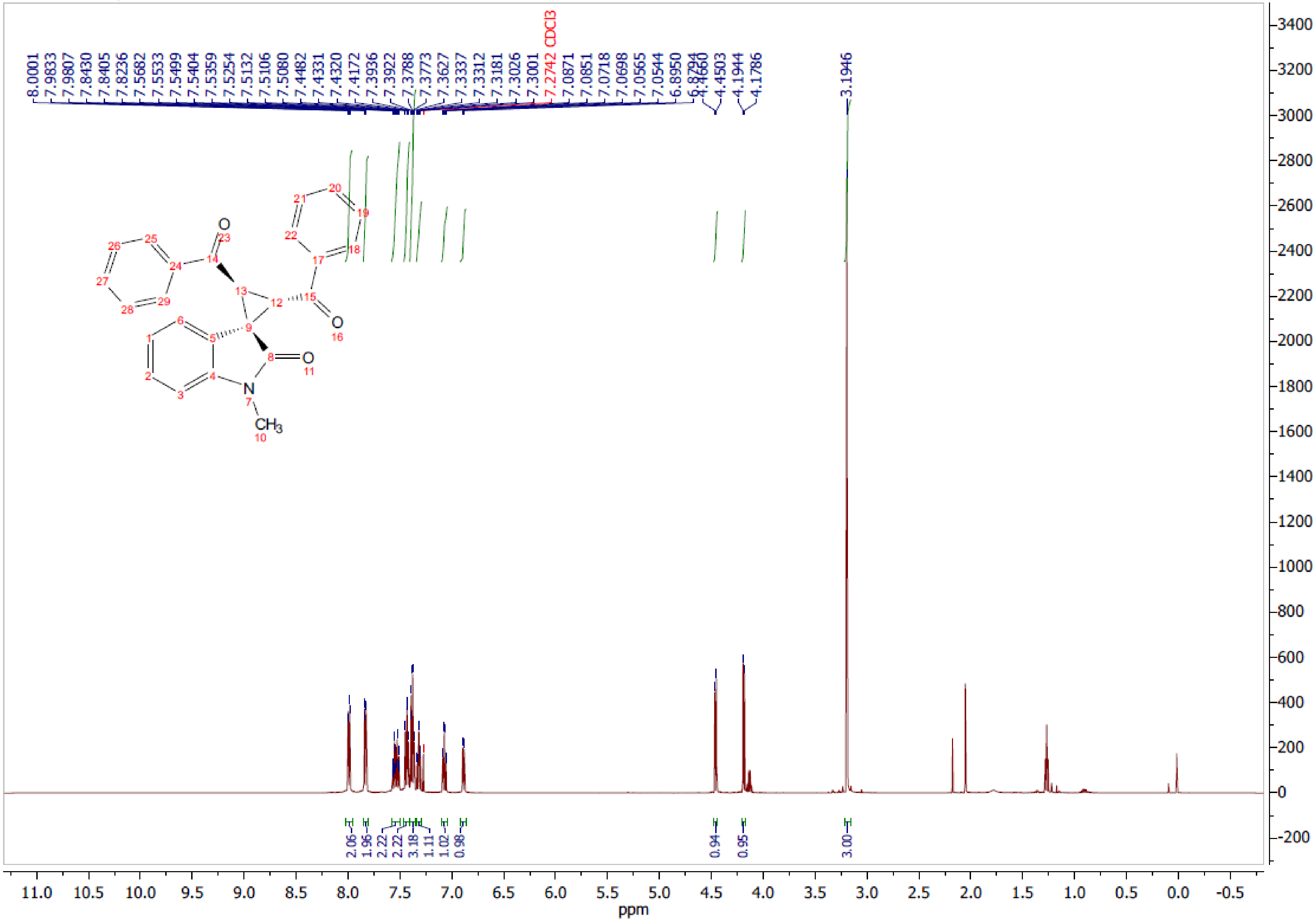

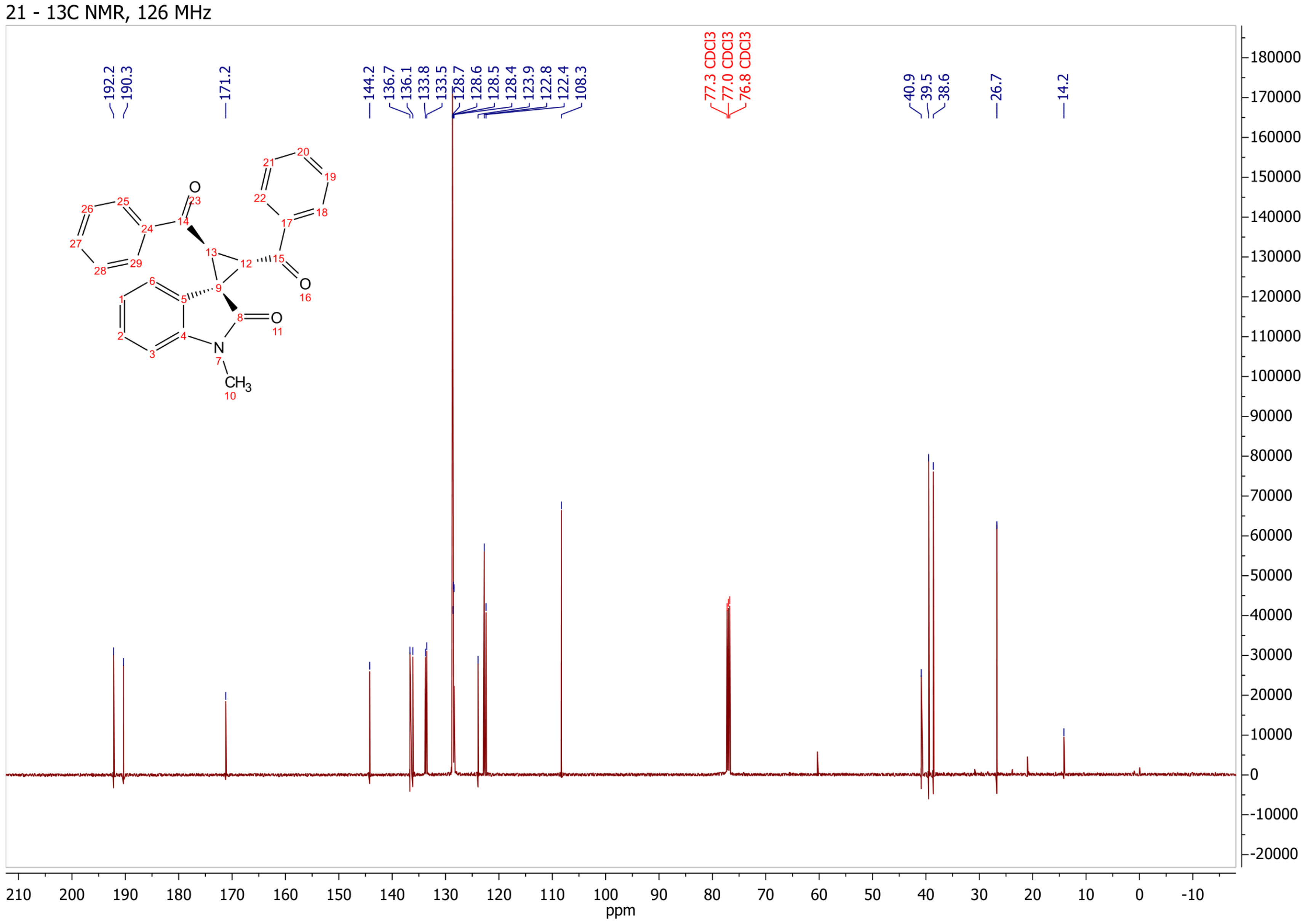
21 - 13C NMR, 126 MHz
192.2
190.3
171.2
144.2
136.7
136.1
133.8
133.5
128.7
128.6
128.5
128.4
123.9
122.8
122.4
108.3
77.3 CDCl3
77.0 CDCl3
76.8 CDCl3
40.9
39.5
38.6
26.7
14.2
ppm

24 - 1H NMR, 500 MHz

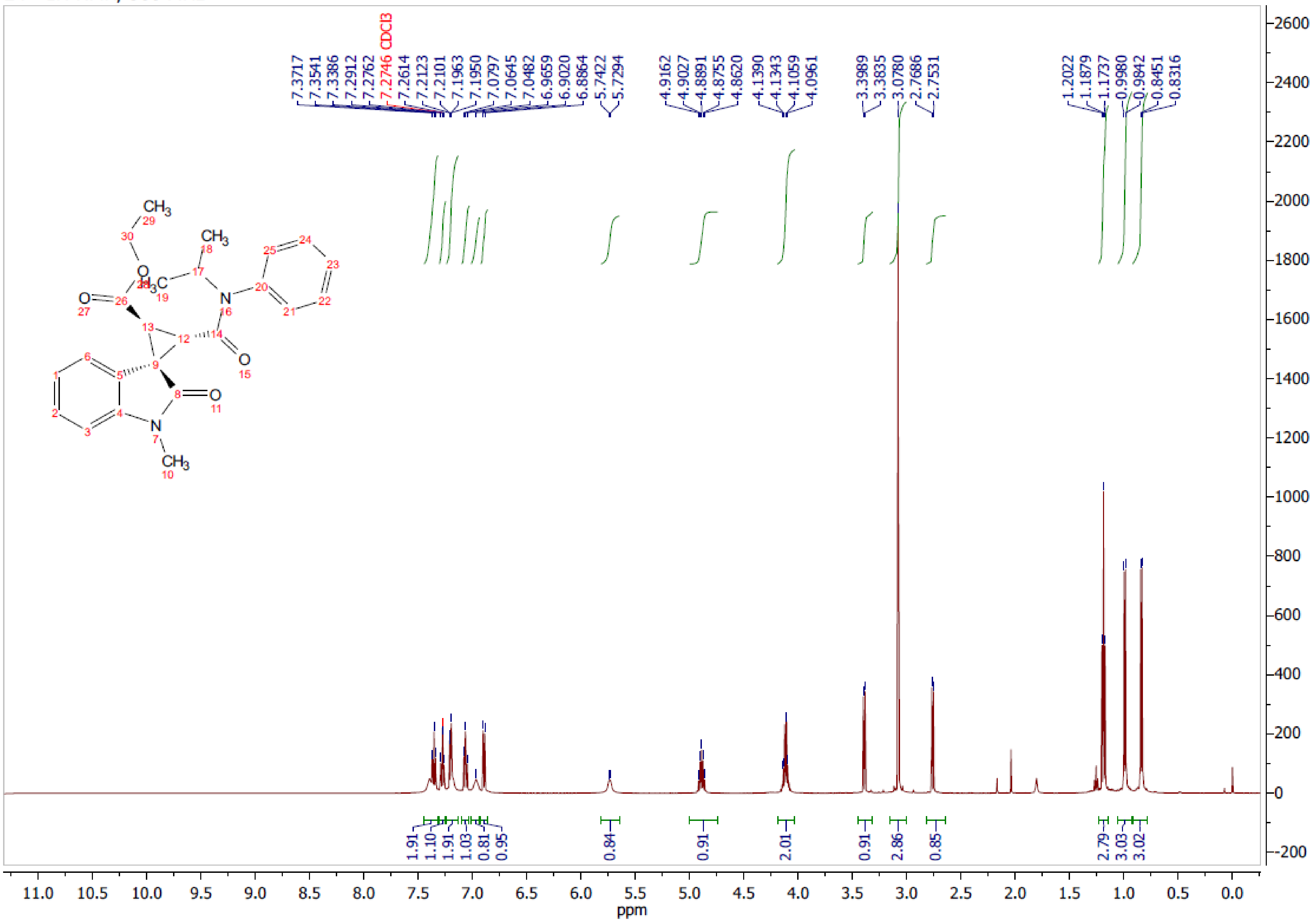

24 - 13C NMR, 126 MHz

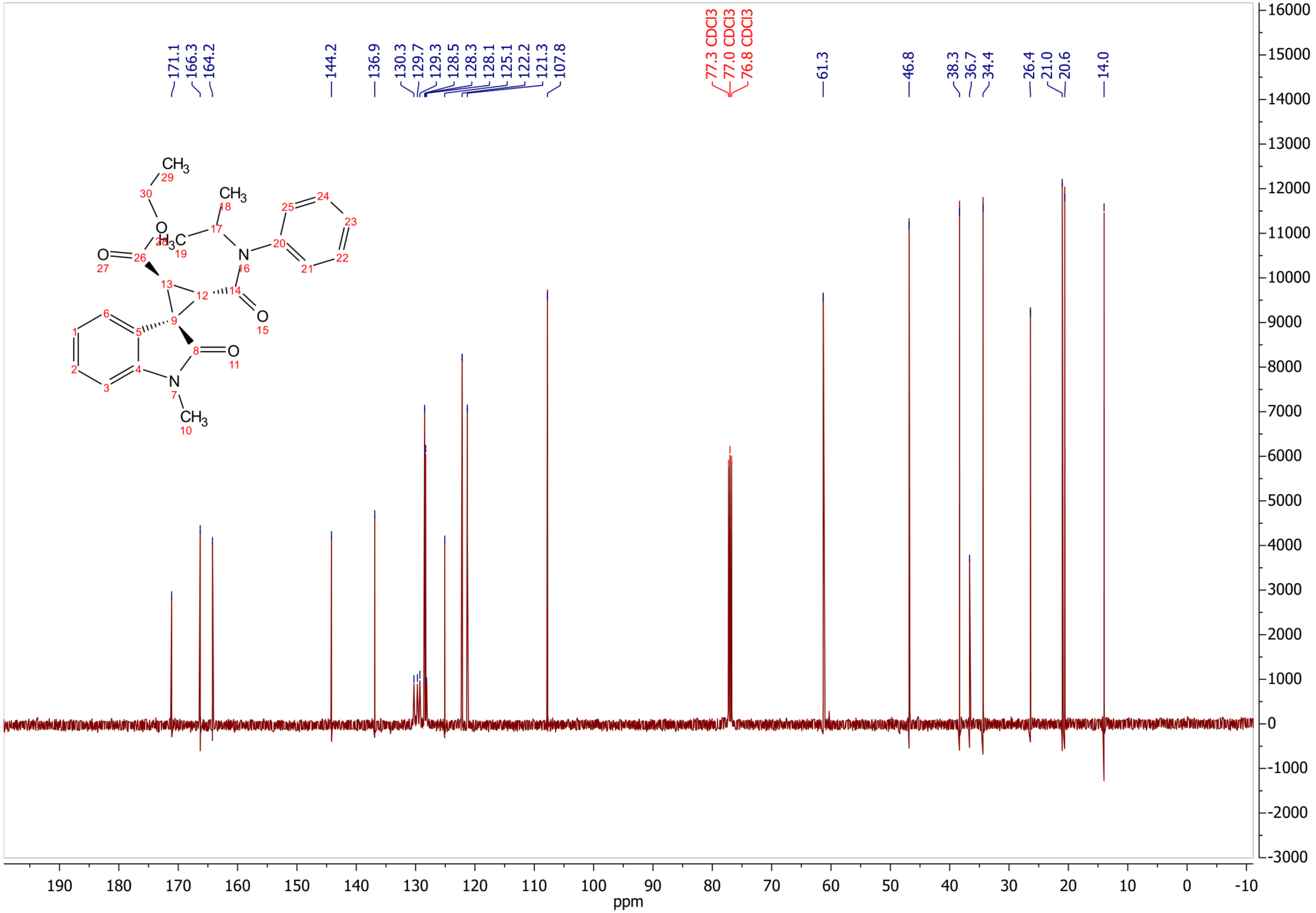

11. References

(1) Ilangovan, A.; Satish, G. Direct Amidation of 2'-Aminoacetophenones Using I2-TBHP: A Unimolecular Domino Approach toward Isatin and Iodoisatin. *J. Org. Chem.* **2014**, *79* (11), 4984–4991. https://doi.org/10.1021/jo500550d.

(2) Mamuye, A. D.; Monticelli, S.; Castoldi, L.; Holzer, W.; Pace, V. Eco-Friendly Chemoselective N-Functionalization of Isatins Mediated by Supported KF in 2-MeTHF. *Green Chem.* **2015**, *17* (8), 4194–4197. https://doi.org/10.1039/c5gc01002k.

(3) Buxton, C. S.; Blakemore, D. C.; Bower, J. F. Reductive Coupling of Acrylates with Ketones and Ketimines by a Nickel-Catalyzed Transfer-Hydrogenative Strategy. *Angew. Chemie - Int. Ed.* **2017**, *56* (44), 13824–13828. https://doi.org/10.1002/anie.201707531.

(4) Sun, Z.; Xiang, K.; Tao, H.; Guo, L.; Li, Y. Synthesis of 2-Substituted 3-Chlorobenzofurans via TMSCl-Mediated Nucleophilic Annulation of Isatin-Derived Propargylic Alcohols. *Org. Biomol. Chem.* **2018**, *16* (33), 6133–6139. https://doi.org/10.1039/c8ob01731j.

(5) Ryu, H.; Seo, J.; Ko, H. M. Synthesis of Spiro[Oxindole-3,2′-Pyrrolidine] Derivatives from Benzynes and Azomethine Ylides through 1,3-Dipolar Cycloaddition Reactions. *J. Org. Chem.* **2018**, *83* (22), 14102–14109. https://doi.org/10.1021/acs.joc.8b02117.

(6) Zhang, Y.; Luo, L.; Ge, J.; Yan, S. Q.; Peng, Y. X.; Liu, Y. R.; Liu, J. X.; Liu, C.; Ma, T.; Luo, H. Q. "on Water" Direct Organocatalytic Cyanoarylmethylation of Isatins for the Diastereoselective Synthesis of 3-Hydroxy-3-Cyanomethyl Oxindoles. *J. Org. Chem.* **2019**, *84* (7), 4000–4008. https://doi.org/10.1021/acs.joc.8b03194.

(7) Bojack, G.; Baltz, R.; Dittgen, J.; Fischer, C.; Freigang, J.; Getachew, R.; Grill, E.; Helmke, H.; Hohmann, S.; Lange, G.; Lehr, S.; Porée, F.; Schmidt, J.; Schmutzler, D.; Yang, Z.; Frackenpohl, J. Synthesis and Exploration of Abscisic Acid Receptor Agonists Against Dought Stress by Adding Constraint to a Tetrahydroquinoline-Based Lead Structure. *European J. Org. Chem.* **2021**, *2021* (23), 3442–3457. https://doi.org/10.1002/ejoc.202100415.

(8) Frisch, M. J. .; Trucks, G. W. .; Schlegel, H. B. .; Scuseria, G. E. .; Robb, M. A. .; Cheeseman, J. R. .; Scalmani, G. .; Barone, V. .; Petersson, G. A. .; Nakatsuji, H. .; Li, X. .; Caricato, M. .; Marenich, A. V. .; Bloino, J. .; Janesko, B. G. .; Gomperts, R. .; Mennucci, B. .; Hratchian, H. P. .; Ortiz, J. V. .; Izmaylov, A. F.; Sonnenberg, J. L. .; Williams-Young, D. .; Ding, F. .; Lipparini, F. .; Egidi, F. .; Goings, J. .; Peng, B. .; Petrone, A. .; Henderson, T. .; Ranasinghe, D.; Zakrzewski, V. G. .; Gao, J. .; Rega, N. .; Zheng, G. .; Liang, W. .; Hada, M. .; Ehara, M. .; Toyota, K. .; Fukuda, R. .; Hasegawa, J. .; Ishida, M. .; Nakajima, T. .; Honda, Y. .; Kitao, O. .; Nakai, H. .; Vreven, T. .; Throssell, K. .; Montgomery, J. A., J. .; Peralta, J. E. .; Ogliaro, F. .; Bearpark, M. J. .; Heyd, J. J. .; Brothers, E. N. .; Kudin, K. N. .; Staroverov, V. N. .; Keith, T. A. .; Kobayashi, R. .; Normand, J. .; Raghavachari, K. .; Rendell, A. P. .; Burant, J. C. .; Iyengar, S. S. .; Tomasi, J. .; Cossi, M. .; Millam, J. M. .; Klene, M. .; Adamo, C. .; Cammi, R. .; Ochterski, J. W. .; Martin, R. L. .; Morokuma, K. .; Farkas, O. .; Foresman, J. B. .; Fox, D. J. Gaussian 16, Revision C.01. Wallingford CT 2016.

(9) Chai, J. Da; Head-Gordon, M. Long-Range Corrected Hybrid Density Functionals with Damped Atom-Atom Dispersion Corrections. *Phys. Chem. Chem. Phys.* **2008**, *10* (44), 6615–6620. https://doi.org/10.1039/b810189b.

(10) Hay, P. J.; Wadt, W. R. Ab Initio Effective Core Potentials for Molecular Calculations. Potentials for K to Au Including the Outermost Core Orbitale. *J. Chem. Phys.* **1985**, *82* (1), 299–310. https://doi.org/10.1063/1.448975.

(11) Scalmani, G.; Frisch, M. J. Continuous Surface Charge Polarizable Continuum Models of Solvation. I. General Formalism. *J. Chem. Phys.* **2010**, *132* (11). https://doi.org/10.1063/1.3359469.

(12) Ribeiro, R. F.; Marenich, A. V.; Cramer, C. J.; Truhlar, D. G. Use of Solution-Phase Vibrational Frequencies in Continuum Models for the Free Energy of Solvation. *J. Phys. Chem. B* **2011**, *115* (49), 14556–14562. https://doi.org/10.1021/jp205508z.

(13) Li, Y. P.; Gomes, J.; Sharada, S. M.; Bell, A. T.; Head-Gordon, M. Improved Force-Field Parameters for QM/MM Simulations of the Energies of Adsorption for Molecules in Zeolites and a Free Rotor Correction to the Rigid Rotor Harmonic Oscillator Model for Adsorption Enthalpies. *J. Phys. Chem. C* **2015**, *119* (4), 1840–

1850. https://doi.org/10.1021/jp509921r.

(14) Hratchian, H. P.; Schlegel, H. B. Following Reaction Pathways Using a Damped Classical Trajectory Algorithm. *J. Phys. Chem. A* **2002**, *106* (1), 165–169. https://doi.org/10.1021/jp012125b.

(15) Satham, L.; Sankara, C. S.; Namboothiri, I. N. N. A Morita–Baylis–Hillman Pathway to Wittig Products: One-Pot Transformation of Nitroalkylideneoxindoles to Oxindolylidene-Carboxylates. *European J. Org. Chem.* **2020**, *2020* (44), 6903–6908. https://doi.org/10.1002/ejoc.202000852.
